# Manipulating the Rate and Overpotential for Electrochemical Water Oxidation: Mechanistic Insights for Cobalt Catalysts Bearing Noninnocent Bis(benzimidazole)pyrazolide Ligands

**DOI:** 10.1021/acsorginorgau.3c00061

**Published:** 2024-02-14

**Authors:** Yu-Ting Wu, Sharad V. Kumbhar, Ruei-Feng Tsai, Yung-Ching Yang, Wan-Qin Zeng, Yu-Han Wang, Wan-Chi Hsu, Yun-Wei Chiang, Tzuhsiung Yang, I-Chung Lu, Yu-Heng Wang

**Affiliations:** † Department of Chemistry, National Tsing Hua University, Hsinchu 30013, Taiwan; ‡ Department of Chemistry, National Chung Hsing University, Taichung 40227, Taiwan

**Keywords:** water oxidation, molecular electrocatalyst, homogeneous catalysis, noninnocent ligand, rate–overpotential correlation

## Abstract

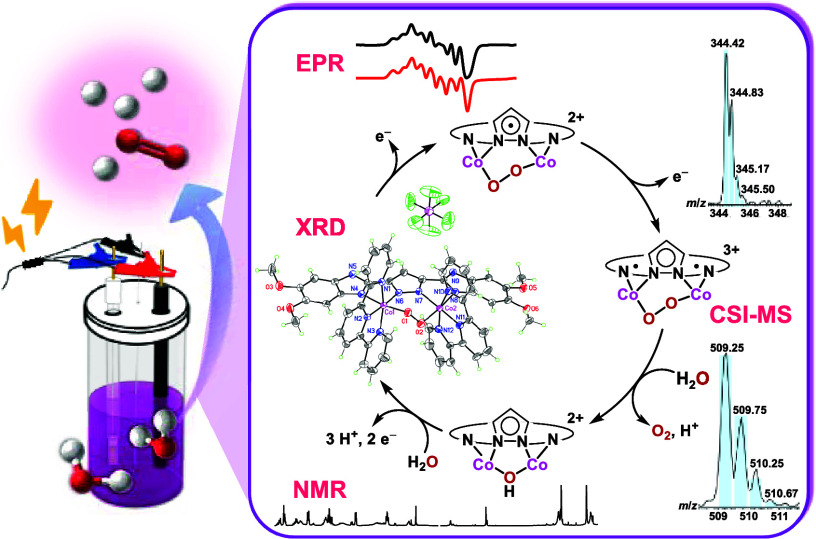

Electrochemical water oxidation is known as the anodic reaction of water splitting. Efficient design and earth-abundant electrocatalysts are crucial to this process. Herein, we report a family of catalysts (**1**–**3**) bearing bis(benzimidazole)pyrazolide ligands (**H**_**2**_**L1**–**H**_**2**_**L3**). **H**_**2**_**L3** contains electron-donating substituents and noninnocent components, resulting in catalyst **3** exhibiting unique performance. Kinetic studies show first-order kinetic dependence on [**3**] and [H_2_O] under neutral and alkaline conditions. In contrast to previously reported catalyst **1**, catalyst **3** exhibits an insignificant kinetic isotope effect of 1.25 and zero-order dependence on [NaOH]. Based on various spectroscopic methods and computational findings, the **L3**Co_2_^III^(μ-OH) species is proposed to be the catalyst resting state and the nucleophilic attack of water on this species is identified as the turnover-limiting step of the catalytic reaction. Computational studies provided insights into how the interplay between the electronic effect and ligand noninnocence results in catalyst **3** acting via a different reaction mechanism. The variation in the turnover-limiting step and catalytic potentials of species **1**–**3** leads to their catalytic rates being independent of the overpotential, as evidenced by Eyring analysis. Overall, we demonstrate how ligand design may be utilized to retain good water oxidation activity at low overpotentials.

## Introduction

Energy security and the mitigation of climate change are both known to be at the core of energy policies worldwide.^1−3^ Due to the ongoing population growth and industrialization of the developing world, the use of renewable energy sources to reduce our dependence on fossil fuels is critical to address these energy-related issues.^4,5^ In addition to renewable energy sources, such as solar power, hydropower, and biomass, H_2_ has gradually gained global attention as an alternative to conventional fossil fuels.^6,7^ Due to its cleanliness and high energy density, H_2_ is considered a promising energy carrier to store energy in the form of chemical H–H bonds.^8−12^ Although H_2_ can be generated via water splitting, efficient hydrogen evolution can only be achieved using efficient, inexpensive, and robust (electro)catalysts for the water oxidation reaction (WOR, eq 1).^13−18^ However, the involvement of multielectron/multiproton reactions along with both bond-forming and bond-breaking processes results in sluggish reaction kinetics and limits the overall performance of the WOR.^19−22^
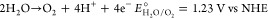
1

With the above considerations in mind, exploring low-cost and earth-abundant catalysts to promote practical application of the WOR in energy conversion is imperative. Therefore, precious metal catalysts composed of noble elements,^23^ such as Ru and Ir, must be replaced. To date, various first-row transition metal catalysts,^24−27^ such as Co-based complexes, have been considered suitable candidates for use as molecular water oxidation catalysts (MWOCs) because of the similarity between Co^III^ and Ru^II^ (i.e., a low-spin electronic structure and an octahedral geometry).^28−32^ Although Ru-based WOCs have been extensively studied, systematic investigations have yet to be carried out on Co-based WOCs.

Homogeneous molecular (electro)catalysts are of particular interest due to the ease of probing their reaction mechanisms by spectroscopic methods, and the fact that their reaction sites can be adjusted using alternating ligand scaffolds.^15,33^ With a well-defined active site that can be characterized spectroscopically, the rational design of molecular catalysts toward efficient (electro)catalysis becomes more feasible. For example, in the case of metal-based catalysts, the reaction rate and overpotential (η) could be improved by modifying the ligand primary and secondary coordination spheres to optimize the electronic and steric effects, proton relay, hemilability, and noninnocent characteristics.^34−41^ One vital feature of the noninnocent ligand motif is that the ligand can participate in both proton- and electron-transfer (PT and ET) steps (i.e., (de)protonation and redox events), which could lead to improved catalytic efficiencies for energy-related multielectron, multiproton reactions by facilitating the PT and ET rates.^42−46^

**Figure 1:**
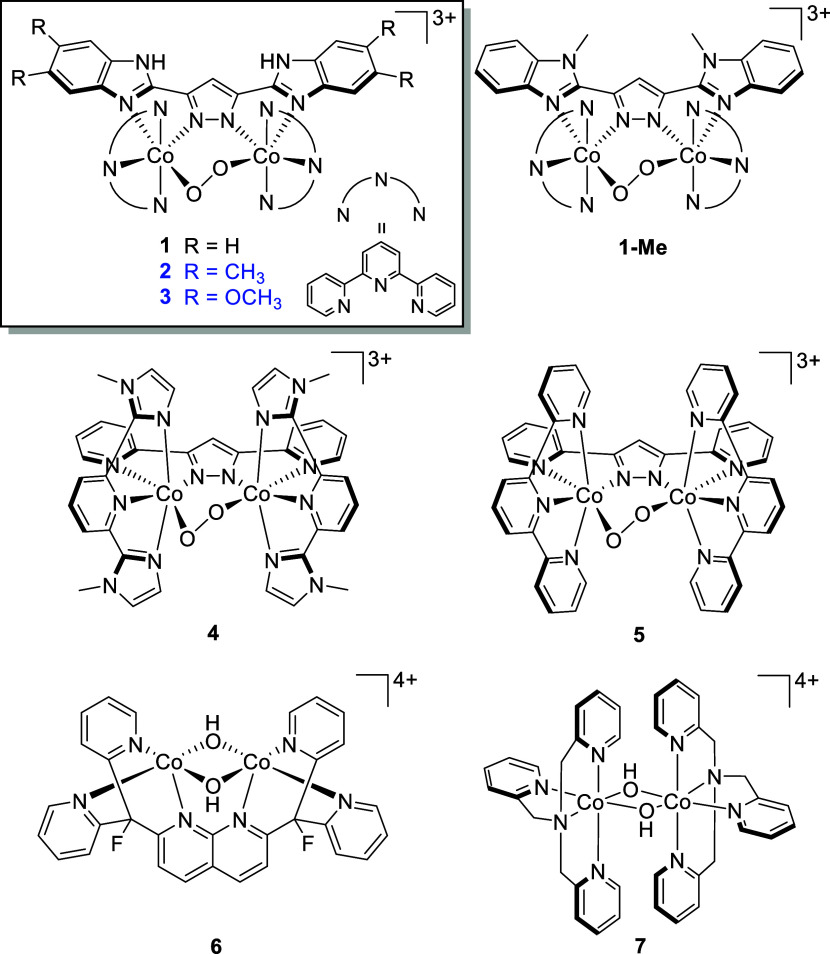
Structures of **1** and **1-Me** dimeric Co-based WOCs previously synthesized by our group. Structures of catalysts **2** and **3** are the new dimeric Co-based WOCs described herein. The structures of catalysts **4**–**7** are also shown; these are previously reported dimeric Co-based WOCs.^47−49^

Recently, our group investigated dimeric cobalt complexes, **1** and **1-Me**, bearing bis(benzimidazole)pyrazolide (bbp) ligands, as homogeneous catalysts for the electrocatalytic oxidation of water (Figure 1).^30^ Preliminary mechanistic studies of the WOR catalyzed by **1** indicated that the rate exhibited a first-order dependence on [**1**] and [H_2_O], and a kinetic isotope effect (KIE) of 2.67 was observed when the reaction was conducted under alkaline conditions. Notably, in the case of catalyst **1**, which bears a noninnocent ligand that exhibited higher rates at lower overpotentials, a reverse trend in the rate–overpotential correlation was observed relative to **1-Me** and the majority of previously reported dimeric Ru-based WOCs.^30^ This encouraging result suggested that both the kinetic and thermodynamic performances of an MWOC can be improved by introducing noninnocent ligands, thereby paving a route to developing efficient nonprecious metal catalysts for application in the WOR.

Therefore, in the current study, we focus on **3** having a similar framework to **1** but incorporating electron-donating substituents on the bbp ligand to examine the synergy between the electronic effect and the noninnocent behavior in terms of its effect on the catalyst performance (Figure 1). In addition, a comprehensive mechanistic study is carried out based on experimental and computational data, and the turnover-limiting steps (TLSs) and catalysis-initiating potentials (*E*_cat_) of the prepared catalysts are determined. The results of this study are expected to provide valuable insights into developing competent MWOCs.

## Results and Discussion

### Preparation and Characterization of the Dimeric Cobalt Complexes

**Figure 2:**
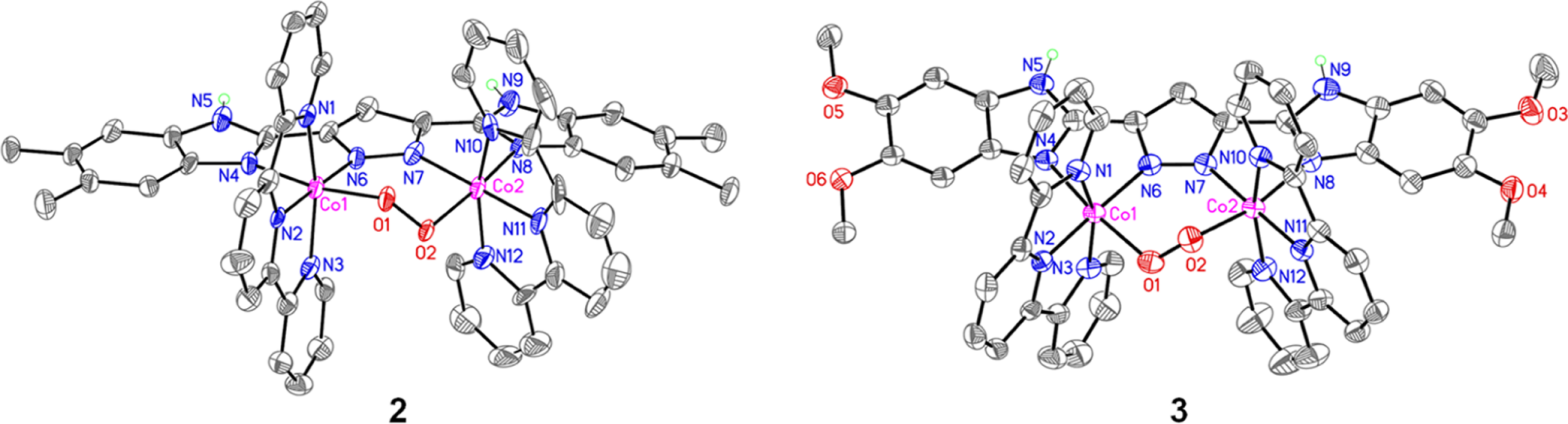
Oak ridge thermal ellipsoid plot (ORTEP) diagrams of complexes **2** and **3** with 30% probability ellipsoids. The hydrogen atoms and counterions (PF_6_^–^) have been omitted for clarity.

The cooperativity of (redox) noninnocent ligands on molecular catalysts for application in small molecule catalysis (e.g., water oxidation and carbon dioxide reduction) has manifested in kinetics and thermodynamics.^34−36^ Thus, to further explore the synergy between the electronic effect and the (redox) noninnocent nature of MWOCs, a series of dimeric cobalt complexes bearing bbp ligands was prepared, wherein these ligands contained electron-donating substituents on their benzimidazolyl rings (Figure 2, –Me (**2**) and –OMe (**3**)). Complexes **2**–**3** were synthesized via a metalation reaction between Co(tpy)Cl_2_ (tpy: terpyridine) and the corresponding bbp ligands (**H**_**2**_**L2** and **H**_**2**_**L3**) under aerobic conditions, as described in Section S2b,c of the Supporting Information. The X-ray crystal structures of **2** and **3** revealed that the cobalt ions occupy a distorted octahedral geometry, with the μ-peroxo-bridged Co_2_^III^ unit and each Co^III^ center being supported by a tridentate tpy ligand and two N-donor sites through the backbone bpp scaffold. The sharp ^1^H and ^13^C NMR spectra recorded for **2** and **3** were indicative of their low-spin 3d^6^ electronic configurations (Section S17 of the Supporting Information).^30,47,50^

### Electrochemical Behavior of the Dimeric Cobalt Complexes

**Figure 3:**
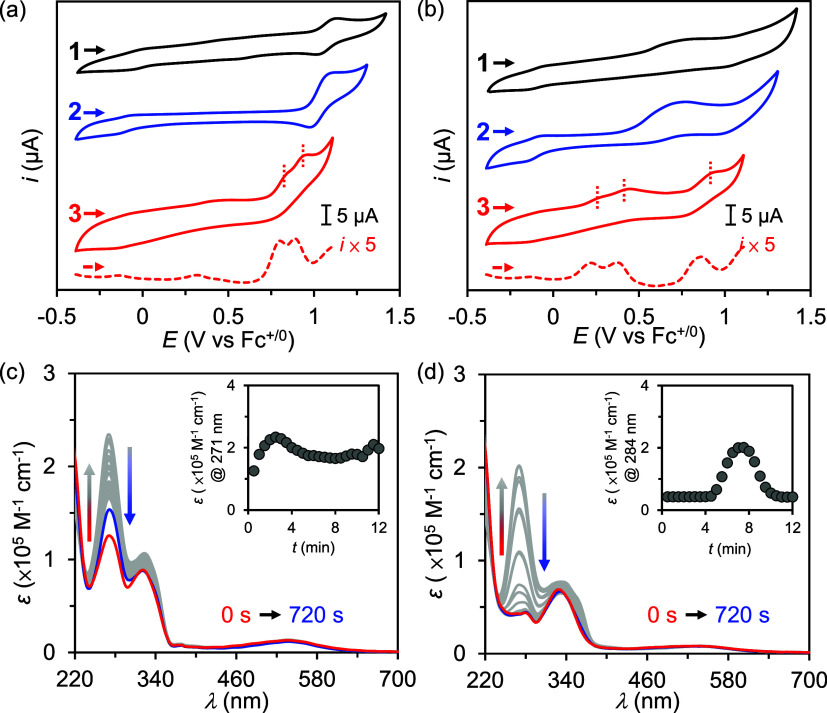
(a) Cyclic voltammograms of **1** (black),^30^**2** (blue), and **3** (red) in CH_3_CN. (b) Cyclic voltammograms of **1**, (black),^30^**2** (blue), and **3** (red) in CH_3_CN in the presence of 10 mM NaOH, 10 mM NaOH, and 10 mM NaOH containing 1 mM 15-crown-5 crown ether, respectively. 15-Crown-5 crown ether was added to increase the solubility of NaOH and the nucleophilicity of the hydroxide ion in anhydrous CH_3_CN.^51^ The redox potentials of **3** were further confirmed by DPV ([cat] 0.4 mM; scan rate: 100 mV/s; working electrode (WE): glassy carbon (GC) disk electrode). UV–vis SEC spectra were acquired during the potential sweep of (c) **2** and (d) **3** in MeCN under alkaline conditions ([**2** or **3**]: 0.04 mM, [NBu_4_OH]: 1 mM, WE: Pt gauze). Scan rate: 1 mV/s; initial and final potential (mV): 450 (**2**) and 100 (**3**); switching potential (mV): 850 (**2**) and 500 (**3**). CVs were plotted by using the IUPAC convention. See the Supporting Information for the general considerations for the cyclic voltammetric measurements.

Complexes **2** and **3** were subjected to cyclic voltammetry (CV) in a mixture of acetonitrile (CH_3_CN) and 0.1 M tetrabutylammonium hexafluorophosphate (TBAP, a supporting electrolyte) under anaerobic conditions. Note that all potentials reported in this work are relative to the ferrocenium/ferrocene couple ( Fc^+/0^). Thus, as shown in Figure 3, the CV of **2** contains a quasi-reversible oxidation wave at 1.01 V, representing a slight cathodic potential shift of ∼80 mV compared to that of **1**. Surprisingly, a significant difference in the redox behavior of **3**, featuring two oxidation processes at 0.82 and 0.91 V, was observed by both CV and differential pulse voltammetry (DPV), indicating that the more electron-donating substituents (σ_p_: –OCH_3_ = −0.27; –CH_3_ = −0.17) influence the redox reservoir properties of these Co-based complexes. In addition, the striking shifts in the redox potentials of **2** and **3** under alkaline conditions were attributed to the increased electron densities resulting from deprotonation of the acidic –NH protons on their bpp ligands (Figure 3b, Table 2, and Section S4b of the Supporting Information). Computational studies further validated the redox couple assignments of **2** and **3**, as shown by the quasi-reversible/irreversible CV behaviors (Table 2, *vide infra*). The chemical reversibility of **2** and **3** under alkaline conditions was further investigated using ultraviolet–visible (UV–vis) spectroelectrochemical (SEC) experiments (Figure 3c,d), wherein the potential windows were selected based on the redox couples obtained from their cyclic voltammograms (Figure 3a,b). The appearance of a new ligand-to-metal charge transfer band centered at the UV region (270–290 nm) indicates that the oxidized forms of **2** and **3** were afforded. Although the recorded cyclic voltammograms show quasi-reversible/irreversible electrochemical processes of **2** and **3**, the resulting UV–vis SEC spectra demonstrated the good chemical reversibility of **2** and **3** under electrochemical conditions.

### Kinetic Studies and Stability Determination

**Figure 4:**
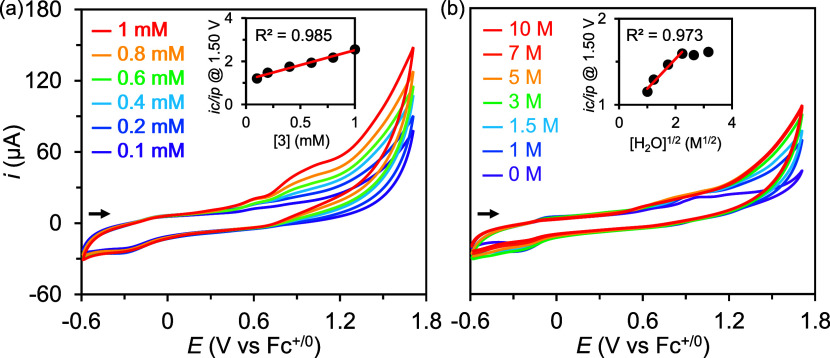
(a) Cyclic voltammograms of **3** (0.1–1 mM) in solutions containing 5 M H_2_O in CH_3_CN. Inset: plot of *i*_c_/*i*_p_ at 1.5 V vs [**3**]. (b) Cyclic voltammograms of **3** (0.4 mM) in solutions containing 0–10 M H_2_O in CH_3_CN. Inset: plot of *i*_c_/*i*_p_ at 1.5 V vs [H_2_O]^1/2^. General conditions: [TBAP]: 0.1 M, WE: GC disk electrode, RE: Ag^+^/Ag (0.01 M AgNO_3_/0.1 M TBAP in MeCN), CE: Pt wire, scan rate: 100 mV/s. CVs were plotted using the IUPAC convention.


Under the catalytic conditions employed herein, the CV results show an increase in the anodic wave when complexes **2** and **3** were present. In addition, the value of the catalytic current (*i*_c_) exhibited a linear, first-order dependence on [**3**] and a half-order dependence on [H_2_O] (Figure 4). These results may be rationalized using eq 2 as follows:^52,53^

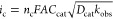
2where *n*_c_ is the number of electrons transferred in the catalytic wave, *F* is the Faraday constant (96,485 C/mol), *A* is the surface area of the WE (2.25 × 10^2^ π cm^2^), *C*_cat_ is the catalyst concentration (mol/cm^3^); *D*_cat_ is the diffusion coefficient of the catalyst (*D*_cat_, determined by diffusion ordered spectroscopy (DOSY), Section S4d of the Supporting Information), *k*_obs_ is defined as pseudo-first-order rate constant or the turnover frequency (TOF, s^–1^), *C*_substrate_ is the substrate concentration (mol/cm^3^), and *k*_cat_ is the catalytic rate constant (M^–1^ s^–1^). Equation 3 accounts for the first-order dependence of the catalytic current on [**3**] (*C*_cat_) and the half-order dependence on [H_2_O] (*C*_substrate_), which together yield the second-order rate law presented in eq 4.
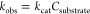
3
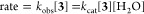
4

**Figure 5:**
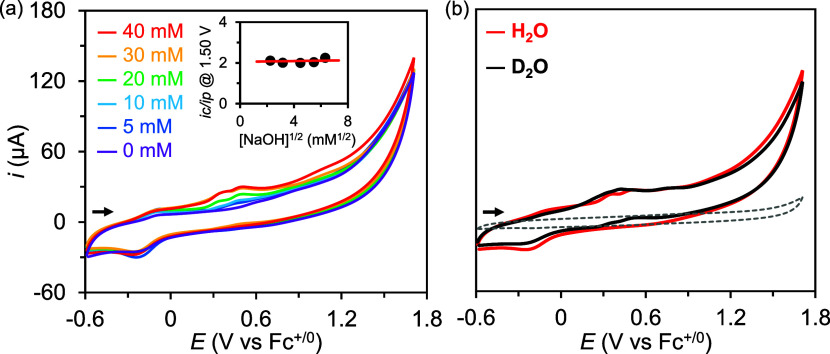
(a) Cyclic voltammograms of **3** (0.4 mM) in solutions containing 0–40 mM NaOH and 5 M H_2_O in CH_3_CN. Inset: plot of *i*_c_/*i*_p_ at 1.5 V vs [NaOH]^1/2^. (b) Cyclic voltammograms of **3** (0.4 mM) in solutions containing 5 M H_2_O and 10 mM NaOH (red) and 5 M D_2_O and 10 mM NaOD (black). General conditions: TBAP: 0.1 M, WE: GC disk electrode, RE: Ag^+^/Ag (0.01 M AgNO_3_/0.1 M TBAP in MeCN), CE: Pt wire, scan rate: 100 mV/s. CVs were plotted using the IUPAC convention.

The kinetic studies of **3** were performed under alkaline conditions to gain further mechanistic insight into the catalytic process. The WOR rates of **3** were observed to be invariable with respect to the NaOH concentration, suggesting a zero-order dependence on [NaOH] (Figure 5a). Upon CV investigation of the KIE for the WOR (Figure 5b), an insignificant KIE of 1.25 was derived using eq 5 (*k*_cat(H)_ and *k*_cat(D)_, rate constants under nondeuterated and deuterated conditions; *i*_c(H)_ and *i*_c(D)_, catalytic currents under nondeuterated and deuterated conditions), suggesting that PT is not the TLS.
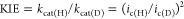
5

The results of these two kinetic experiments are contrary to those obtained for complex **1** under alkaline conditions (Table 1), indicating the possibility that the WOR follows a different reaction pathway due to modulation of the electronic effect and the noninnocence of the ligand scaffold. Subsequently, the electrochemical O_2_ evolution during controlled-potential water electrolysis by **3** was measured using a PreSens Microx 4 fiber-optic oxygen meter, and the Faraday efficiency was determined to be ≥90% over 3 h (Section S6b of the Supporting Information). Moreover, a Ti^IV^(O)SO_4_ colorimetric assay confirmed the negligible formation of H_2_O_2_ in the reaction mixture (1% H_2_O_2_, *n*_c_ ≈ 4),^54,55^ suggesting that O_2_ is the main product (Section S6c of the Supporting Information). The data presented above indicate that **3** is a competent WOC.

**Table 1:** Comparison of the Kinetic Parameters for the WOR Catalyzed by **1** and **3** under Neutral and Alkaline Conditions^a^

		**1**^b^	**3**
rate law		R ∝ [cat]^1^[H_2_O]^1^	R ∝ [cat]^1^[H_2_O]^1^
base assistance		yes	no
KIE	neutral	1.32(1)	1.26(2)
	alkaline	2.67(10)	1.25(6)

a General conditions: [cat]: 0.4 mM, [NaOH]: 10 mM, TBAP: 0.1 M, solvent: MeCN. [H_2_O]: 10 M (18%, v/v) for **1**; 5 M (9%, v/v).

b Obtained from ref (38).

The stability of **3** was then accessed using a range of techniques.^56^ First, the rinse test was performed according to a previously described procedure,^57^ and it was found that the current obtained using the rinsed WE was similar to the background current (Section S5a of the Supporting Information). In addition, successive CV scans of **3** were carried out for 3 h under catalytic conditions, giving stable currents with no significant increases or decreases observed (Section S5b of the Supporting Information). In addition, after subjecting complex **3** to CPE for 8 h at 1.5 V, energy-dispersive X-ray spectroscopy (EDXS) did not detect the formation of cobalt-containing nanoparticle deposits on the electrode surface above the detection limits (Section S5c of the Supporting Information). These results provide evidence of the homogeneity of the cobalt complexes employed herein and exclude the involvement of heterogeneous CoO_*x*_ in the catalytic process.

### Identify the Reaction Intermediates and the Catalyst Resting State

**Figure 6:**
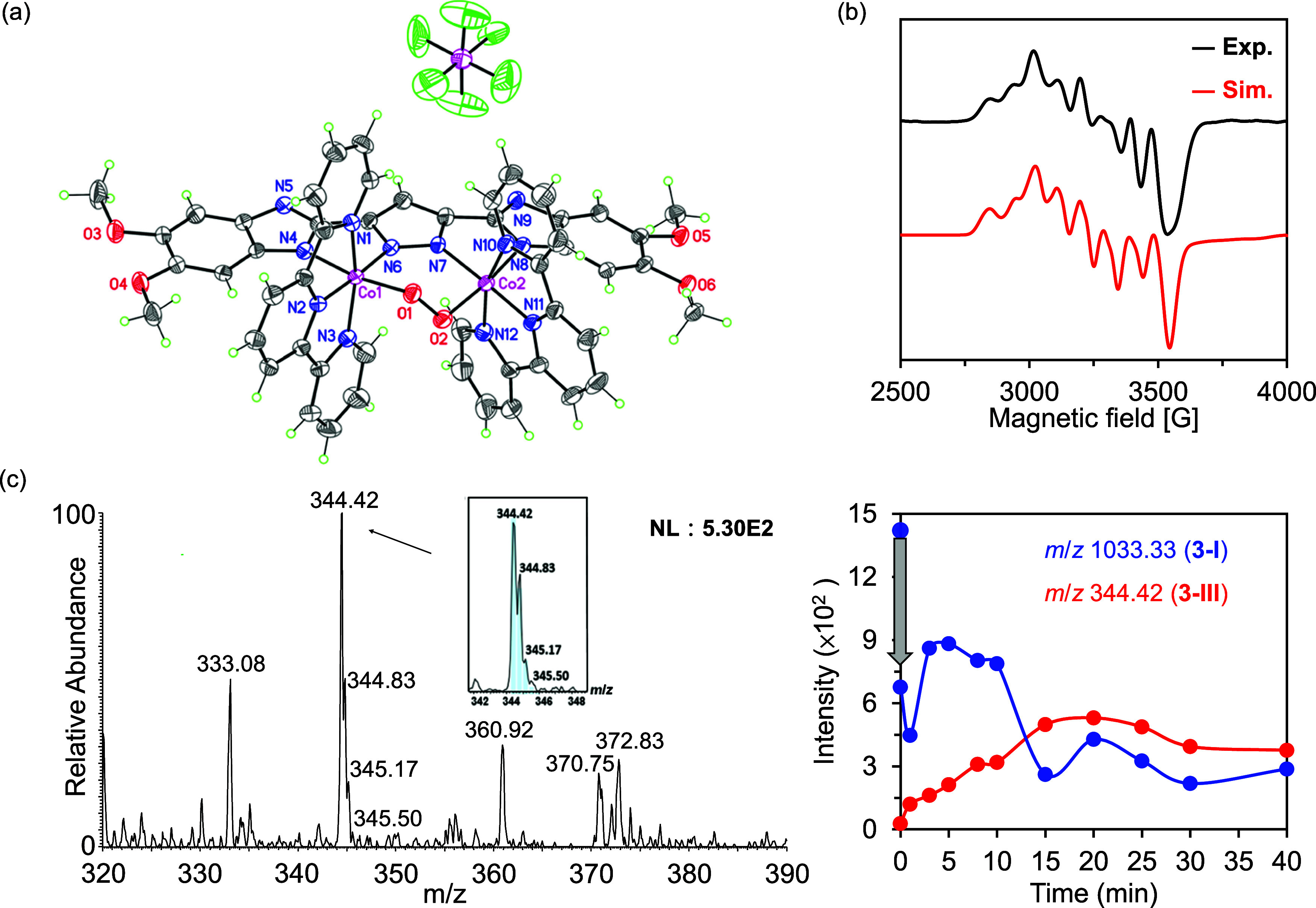
(a) ORTEP diagram of **3-I** with 50% probability ellipsoids. (b) Experimental (black) and simulated (red) EPR spectra of **3-II** in frozen CH_3_CN at 8 K. The EPR spectrum of **3-II** was simulated using EasySpin (version 5.2.35). (c) cold-spray ionization mass spectrometry (CSI-MS) spectrum of **3-III** (*m*/*z* 344.42) in MeCN at −5 °C. The theoretical isotopic distribution is shown in the blue bar. Inset: time-dependent CSI-MS ion intensities were obtained for **3-I** and **3-III**.

Additional experiments were subsequently performed to investigate the Co species involved in the reaction under alkaline conditions. It was found that upon the addition of 2 equiv of sodium hydride to a solution of **3** in anhydrous CH_3_CN, the doubly deprotonated cobalt species **3**^**+**^ (**3-I**) was formed, which was crystallographically characterized (Figure 6a). More specifically, the unit cell of **3-I** consists of a single molecule of the cobalt complex and a single molecule of the PF_6_^–^ anion, which differs from the neutral cobalt complexes **1**–**3**, which contain three molecules of PF_6_^–^ in their unit cells (Section S18 of the Supporting Information). Note that the Co_2_^III^O_2_ core unit of **3-I** was retained, indicating that neither Co^III^–O nor O–O bond cleavage takes place upon the deprotonation of **3**. The ^1^H NMR spectrum recorded for **3-I** confirms its diamagnetic nature, and the upfield shift of the ^1^H signals may be attributed to the shielding effect arising from the negatively charged bpp ligand (Section S12 of the Supporting Information). Subsequently, the one-electron oxidized cobalt species of **3-I** was characterized by in situ electron paramagnetic resonance (EPR) spectroscopy by the addition of 1 equiv of [NBu_4_][IO_4_] to a solution of **3** under alkaline conditions. The resulting EPR spectrum recorded at 8 K exhibited a distinctive hyperfine splitting that correlated best with the low spin (*S* = 1/2) μ-1,2-peroxo dimeric cobalt species, **3**^**2+**^ (**3-II**).^58^ In addition, the simulated EPR spectrum was in good agreement with the experimental data of **3-II**, yielding the principal *g* values of *g*_*x*_ = 2.168, *g*_*y*_ = 2.147, and *g*_*z*_ = 1.965, along with the *A*-tensors of *A*_*x*_^N^, *A*_*y*_^N^, *A*_*z*_^N^ = 26, 30, 35, and *A*_*x*_^Co^, *A*_*y*_^Co^, and *A*_*z*_^Co^ = 102, 95, and 109 in units of Gauss (Figure 6b and Section S13 of the Supporting Information).

The cobalt species participating in the redox process under alkaline conditions were then analyzed by cold-spray ionization mass spectrometry (CSI-MS). Note that the CSI-MS platform prepared in-house provides the capability of monitoring the time-dependent progression of the reaction intermediates. Therefore, a reaction mixture containing 0.1 mM **3**, 2.5 mM [NBu_4_][OH], and 1 mM [NBu_4_][IO_4_] in anhydrous CH_3_CN was introduced into the positive mode CSI source (Figure 6c and Section S14 of the Supporting Information). The time-dependent CSI-MS spectra indicated that the ion intensity at a mass–charge ratio (*m*/*z*) of 1033.33 (**3**-**I**, [**3**–2H–3PF_6_]^+^) decreased as a function of time. In addition, the ion signal at *m*/*z* 344.42 was attributed to the formation of a doubly oxidized species of **3-I** (**3**^**3+**^, **3-III**), as illustrated in Figure 6c.

**Figure 7:**
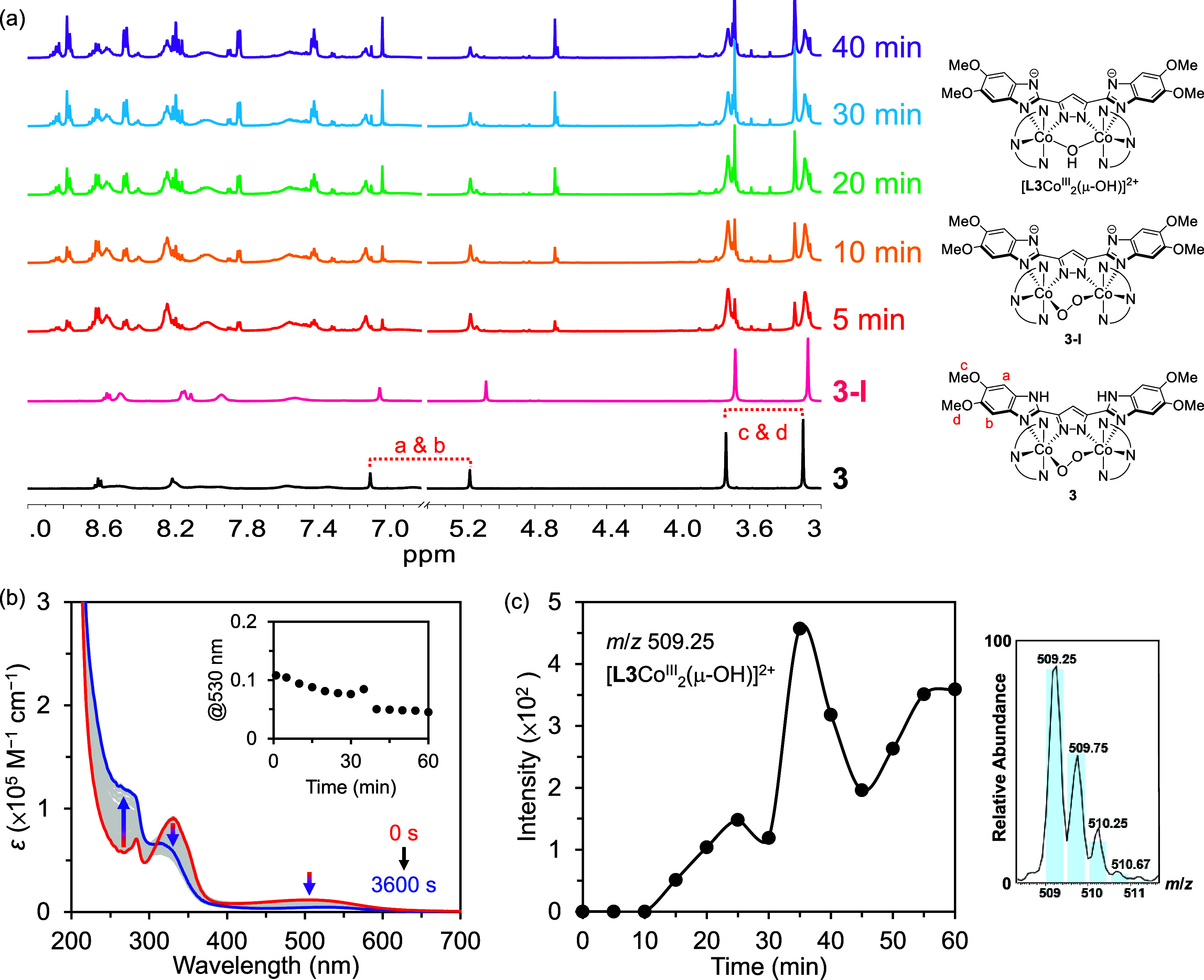
(a) Time-course ^1^H NMR spectra of WOR catalyzed by **3** under alkaline conditions in CD_3_CN and the corresponding ^1^H NMR spectra of **3** and **3-I**. See Figures S78–83 for the one-dimensional and two-dimensional NMR spectra of **3**. (b) Time-course UV–vis SEC spectra of the WOR catalyzed by **3** in CH_3_CN at 1.5 V ([H_2_O] = 5 M, [NBu_4_OH] = 1 mM). An *E*_app_ of 1.5 V was selected because the value of *i*_c_ at 1.5 V is independent of the scan rate (ν) when ν is ≥800 mV/s (Section S9 of the Supporting Information). (c) Time-dependent ion intensity of [**L3**Co_2_^III^(μ-OH)]^2+^ obtained by monitoring the WOR catalyzed by 0.1 mM **3** in CH_3_CN at −5 °C with [NBu_4_IO_4_] = 5 mM, [NBu_4_OH] = 2.5 mM, and [H_2_O] = 10 M. Inset: CSI-MS spectra and isotopic distribution fitting (blue bars) of [**L3**Co_2_^III^(μ-OH)]^2+^.

To probe the catalyst resting state of **3** under the catalytic conditions examined herein, the reaction mixture containing 5 mM **3**, 20 mM NaOH, 100 mM NBu_4_IO_4_, and 5 M H_2_O was monitored by ^1^H NMR spectroscopy. The ^1^H NMR time-course analysis revealed that a new diamagnetic cobalt species with a characteristic signal at δ 4.7 ppm began to form after ∼5 min, as shown in Figure 7a. This cobalt species exhibits distinct chemical shifts from those of **3** and **3-I**, and the integration of all protons suggests that this is a dimeric cobalt species that preserves the identical polypyridyl ligands as **3** (Section S12 of the Supporting Information). In addition, Figure 7b shows the time-dependent UV–vis SEC traces of the WOR catalyzed by **3** under alkaline conditions, wherein the disappearance of the broad transition centered at 500 nm indicates cleavage of the O–O bond from the Co^III^–O–O–Co^III^ core,^30,50,59,60^ which differs from those obtained under noncatalytic conditions (Figure 3c,d). Considered together, the NMR and UV–vis SEC findings indicate that the catalyst resting state is not **3-I**, **3-II**, or **3-III**. Therefore, to further evaluate the catalyst resting state, the catalytic reaction mixture was monitored in situ by CSI-MS for 40 min. The results showed an ion signal at *m*/*z* 509.25, which may be assigned to an [**L3**Co_2_^III^(μ-OH)]^2+^ species, as confirmed by isotopic distribution fitting (see Figure 7c).^48,49,61^ Importantly, the time-dependent analysis of this system demonstrates that the concentration of this intermediate increases during the steady-state reaction period, which suggests that the catalyst resting state may be attributed to a dimeric cobalt μ-hydroxide species.

### Computational Findings and Overall Catalytic Mechanism

Quantum chemical studies using density functional theory (DFT) were subsequently performed to further understand the experimentally observed redox potentials of **3**, the reaction intermediates, and the different KIE and [OH^–^]-dependent results obtained for the WORs catalyzed by **1** and **3**. Initially, the redox potentials of **3**^+^/**3**^2+^ and **3**^2+^/**3**^3+^ were calculated, and the results are summarized in Table 2. Excellent agreements can be observed between the calculated and experimental redox potentials under alkaline conditions (Figure 3b). The first two oxidations at ∼0.2 and 0.4 V were found to be centered on **L3**, differing from the observations made for complex **1**, in which only one accessible oxidation occurred on **L1**.^30^ This was attributed to tetrasubstitution of the bbp ligand by electron-donating methoxy groups.

**Table 2:**
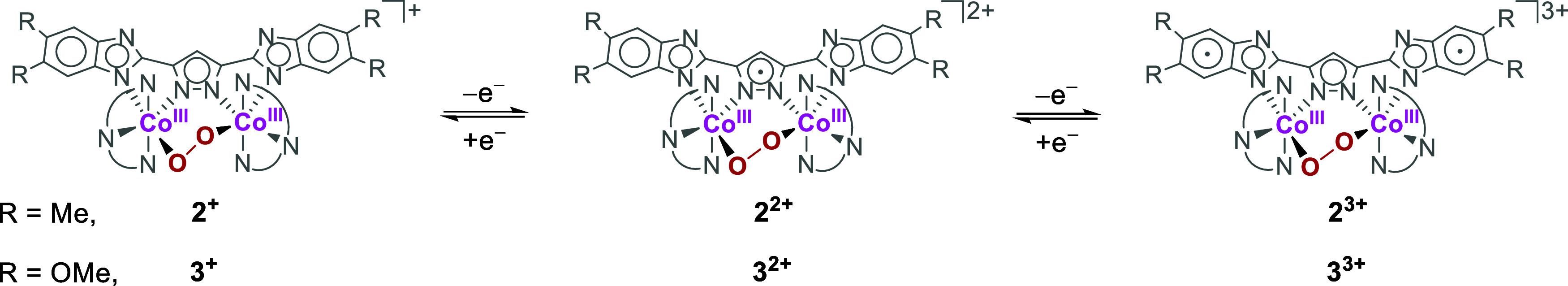
Calculated Redox Potentials of **2**^+^ and **3**^+^ under Alkaline Conditions (V vs. Fc/Fc^+^)

	**2**		**3**	
redox couples^a^	**2**^+^/**2**^2+^	**2**^2+^/**2**^3+^	**3**^+^/**3**^2+^	**3**^2+^/**3**^3+^
expt	0.59	0.73	0.22	0.37
calcd	0.68	0.80	0.27	0.29
assignment	**L2**	**L2**	**L3**	**L3**

a **3**^+^ = **3-I**, **3**^2+^ = **3-II**, **3**^3+^ = **3-III**.

**Figure 8:**
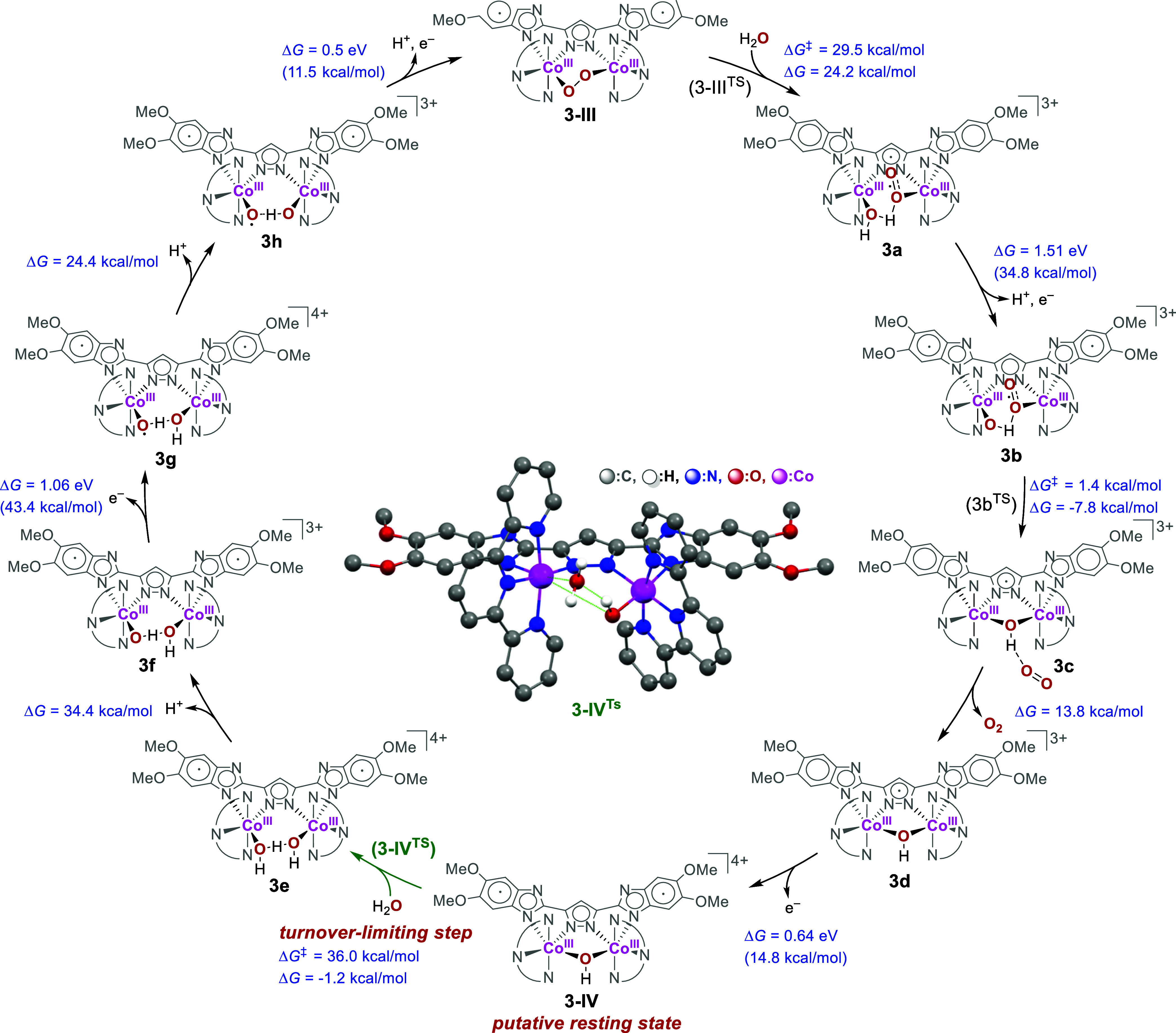
Calculated catalytic cycle for the WOR catalyzed by complex **3**.^62^ See Section S16 of the Supporting Information for the considerations of standard Gibbs free energy.

To investigate the WOR catalyzed by **3**, computational studies were performed, and the calculated catalytic cycle is summarized in Figure 8. The precatalyst was selected to be the doubly oxidized dideprotonated cobalt species (**3-III**) because the WOR is initiated at the potential of **3**^2+^/**3**^3+^ (*E*_cat_) according to the CV results (c.f., Figure 4a). Hydrolysis of **3-III** leads to the generation of the end-on superoxo Co^III^Co^III^ complex **3a** via the transition state **3-III**^**TS**^ featuring an intramolecular ET from the peroxo subunit to the attacked Co center. This elementary step has a Gibbs free energy of reaction (Δ*G*) computed as 24.2 kcal/mol. The resulting intermediate, **3a**, then undergoes proton-coupled electron transfer (PCET) to form a Co^III^Co^III^ hydroxide superoxide species bearing a diradical ligand (**3b**) with a Δ*G* of 34.8 kcal/mol. Subsequently, **3b** proceeds through an intramolecular S_N_2-like attack of the hydroxide ligand on the other Co center to release O_2_ via the facile transition state structure **3b**^**TS**^ to generate the Co^III^Co^III^ μ-hydroxide intermediate (**3c**, Δ*G*: −7.8 eV) that the bpp scaffold features radical character. The subsequent evolution of the O_2_ from **3c** to generate **3d** is exergonic by −13.8 kcal/mol, and **3-IV** with diradical character on **L3** is formed via the one-electron oxidation of **3d** at a potential of 0.64 V.

A turnover-limiting transition state occurs by the hydrolysis of **3-IV** via the attack of the H_2_O molecule on one of the Co^III^ centers (**3-IV**^**TS**^) to generate the corresponding Co^III^Co^III^ aqua hydroxide complex **3e** with a Δ*G* of −1.2 kcal/mol (**3-IV → 3e**).^62^ This finding is consistent with the experimental observation of **3-IV** in CSI-MS (Figure 7c) and the second-order rate law from CV (Table 1). The deprotonation of **3e** produces the dihydroxide species **3f** with a corresponding Δ*G* of 34.4 kcal/mol (equivalent to a p*K*_a_ of 24). **3f** then undergoes stepwise ET/PT to yield **3h** with Δ*G* values of 43.4 and 24.4 kcal/mol, respectively (p*K*_a_ of 17). Lastly, **3-III** is regenerated from **3h** via a PCET with a Δ*G* of 11.5 kcal/mol to complete the catalytic cycle.

As suggested by the KIE and [OH^–^] dependence results as well as the CSI-MS evidence (Section S14 of the Supporting Information), the reaction pathways of **1** and **3** were found to diverge from the [(**L**^**2•+**^)Co_2_^III^(μ-OH), **L** = **L3** or **L1**]^4+^ intermediate (**3-IV**: **L** = **L3**). For **3**, the second H_2_O molecule attacks one of the Co centers is proposed as the TLS (**3-IV** → **3e**), as abovementioned. In the case of **1**, conversely, the deprotonation of [(**L1**^**2•+**^)Co_2_^III^(μ-OH)]^4+^ (**1-IV**) occurs first to generate [(**L1**^**2•+**^)Co_2_^III^(μ-O)]^3+^ (**1-V**), followed by the attack of H_2_O to afford the corresponding dihydroxo intermediate, [(**L1**^**2•+**^)Co_2_^III^(OH)_2_]^3+^ (**1f**). The computational result suggests that **3-IV** possesses an unfavorable calculated p*K*_a_ of 33.0 (equivalent to the standard Gibbs free energy of deprotonation of 43.2 kcal/mol), higher than that of [(**L1**^**2•+**^)Co_2_^III^(μ-OH)]^4+^ (**1-IV**, p*K*_a_ = 27.3) by nearly 6 orders of magnitude (Section S16 of the Supporting Information). This difference in the p*K*_a_ values is expected due to the electron-donating methoxy substituents of **L3**, rendering the Co^III^ centers less Lewis acidic and, thus, the μ-hydroxy motif less acidic. The increased acidity of **1-IV** may lead to the formation of the deprotonated species **1-V**, indicated by CSI-MS ([**L1**Co_2_^III^(μ-O)]^+^, *m*/*z* 897.25; section S14 of the Supporting Information), leading to the divergent mechanism of **1** and **3**.

### Mechanistic Insights into the log(TOF_max_)–*η* Relationship

The TOF and overpotential (*η*) are regarded as normalized descriptors for representing the performance of a catalyst in terms of its kinetics and thermodynamics, respectively.^27,63−65^ To understand the effectiveness of the WOCs examined in this study, their TOF and *η* values were measured and assessed using the log(TOF_max_)–*η* relationship.^27,36^ For this purpose, the TOF_max_ values under different conditions were calculated using eq 2 (see Table 3), and the analysis was performed at a scan rate where the *i*_c_ exhibits a scan-rate independent regime (i.e., ≥800 mV/s, Section S9 of the Supporting Information).^52,53,66^ Within this regime, the *i*_c_ value represents the kinetics of the chemical steps instead of substrate diffusion to the electrode surface; therefore, the resulting TOF can indicate the intrinsic catalytic activity of the molecular (electro)catalyst.^27^ Additionally, the WOR overpotential was estimated by following the literature protocol (Section S10 of the Supporting Information).^67^ The open-circuit potential (OCP) of H^+^/H_2_ was first measured under the experimental conditions of interest, and subsequently, the corresponding thermodynamic potential of H_2_O/O_2_ (*E*_H_2_O/O_2__) was estimated by adding 1.23 V to the H^+^/H_2_ OCP.^68,69^ The *η* were then evaluated using eq 6, where *E*_cat_ is the catalytic potential initiating the WOR (i.e., **2**^2+^/**2**^3+^ and **3**^2+^/**3**^3+^):^53^
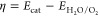
6

**Table 3:** Kinetic and Thermodynamic WOR Data for Complexes **1**–**3**

entry	catalytic condition	catalyst	log(TOF_max_, s^–1^)	*E*_cat_ (mV)^a^^,^^b^	*E*_H_2_O/O_2__ (mV)^a^	*η* (mV)^a^
a	5 M H_2_O, 0 mM NaOH	**1**^c^	1.31	1090	390	700
		**2**	1.11	1010	390	620
		**3**	1.23	890	390	500
b	5 M H_2_O, 10 mM NaOH	**1**^c^	1.54	820	20	800
		**2**	1.23	730	20	710
		**3**	1.33	370	20	350

a Potentials are given relative to Fc^+/0^.

b Redox couple: [cat]^3+^/[cat]^4+^.

c The *i*_c_ values used to calculate the TOF_max_ of **1** were retrieved from ref (30), but the value of *D*_cat_ was determined from DOSY experiments (Section S4d of the Supporting Information).

**Scheme 1:**
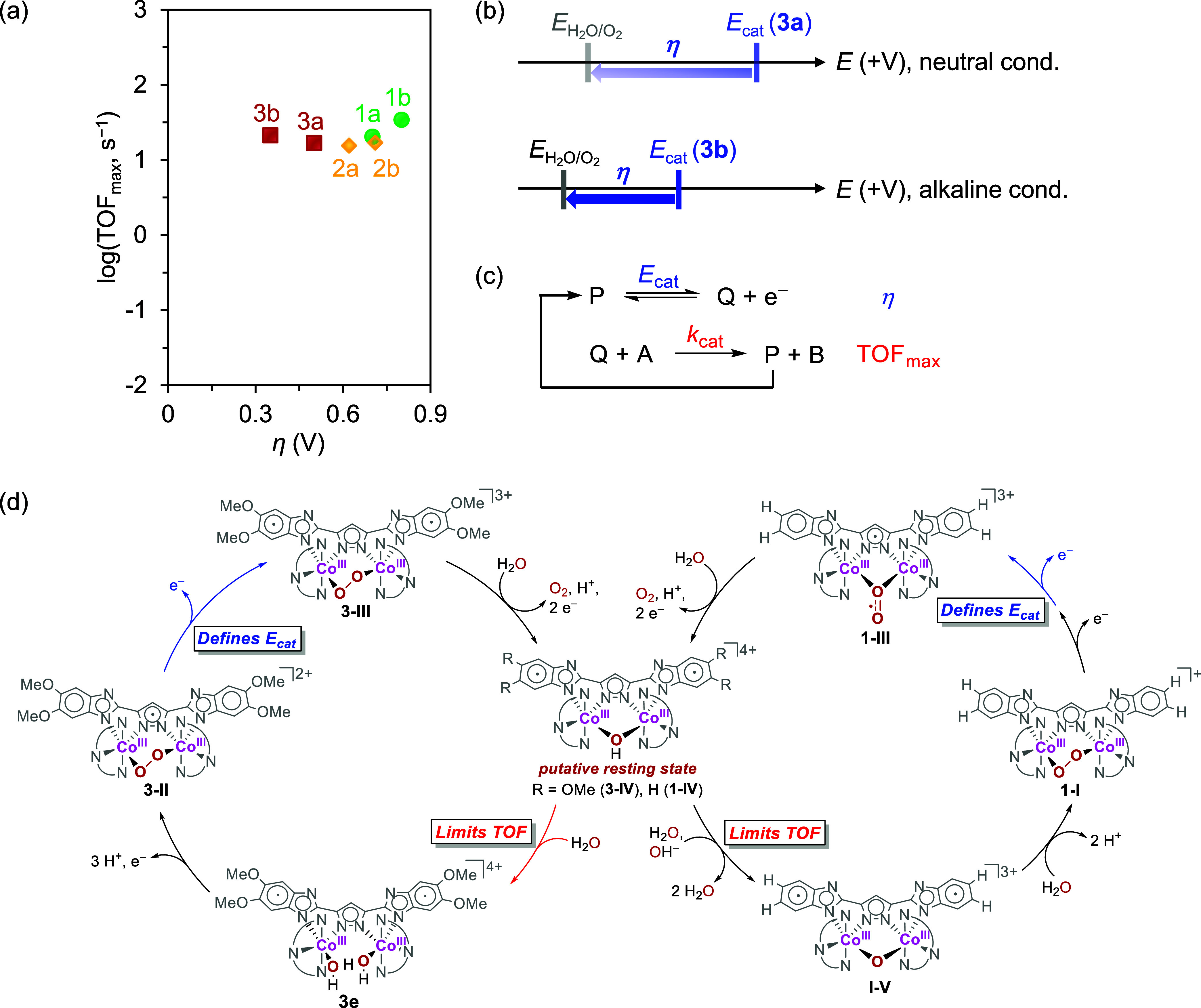
(a) Correlations between log(TOF_max_) versus *η* for the WOR catalyzed by **1**–**3**. (b) Under alkaline conditions, the cathodic shift in *E*_cat_ of **3** is more significant than that of *E*_H_2_O/O_2__, indicating that WOR can be conducted at a lower *η*. (c) Turnover-limiting and overpotential-determining steps in an EC′ mechanism are depicted. (d) Comparison of turnover-limiting and overpotential-determining steps of **1** and **3** The catalyst resting states, **1-IV** and **3-IV**, are proposed on the basis of CSI-MS evidence (Section S14 of the Supporting Information).

Correlating log(TOF_max_) with *η* for catalysts **1**–**3** in neutral and alkaline conditions shows a plateau of TOF_max_ values across the range of ∼400 mV overpotential (Scheme 1a). Although the TLS of complexes **1** and **3** differ under neutral and alkaline conditions,^30^ Eyring analysis revealed that **1**–**3** coincidentally features nearly identical Gibbs energies of activation (Δ*G*^‡^, 17.5–17.9 kcal/mol, Section S15 of the Supporting Information). Consequently, TOF_max_ becomes independent of the driving force under these catalytic conditions, allowing a low-overpotential WOR to take place (i.e., <400 mV) without compromising the catalytic turnover. Although the *η* of complexes **1** and **2** under alkaline conditions are higher than those under neutral conditions (**1b** vs **1a**, **2b** vs **2a**), intriguingly, complex **3** behaves oppositely (**3b** vs **3a**). This result is due to the cathodic shift in *E*_cat_ of **3** under alkaline conditions, which is greater than that of *E*_H_2_O/O_2__, making the low-overpotential WOR accessible (Scheme 1b).

A possible rationale for this log(TOF_max_)–*η* plot could be obtained based on the typical EC′ mechanism, as illustrated in Scheme 1c.^66,70^ In this mechanism, *E* represents a catalysis-initiating redox couple (*E*_cat_) related to the determination of *η*, and C′ represents an irreversible chemical step, namely, the TLS. For an MWOC containing electron-donating substituents in its primary coordination sphere, *E*_cat_ is expected to exhibit a cathodic shift due to the increased electron density on the MWOC. Although only a lower *η* is required to drive the WOR in this instance, an offsetting effect occurs on the successive C′ step. Catalyst regeneration then becomes more challenging because of the more reducing nature of the MWOC counterpart, leading to lower *k*_cat_ and TOF_max_ values. By altering the TLS and *E*_cat_ in the catalytic cycle (Scheme 1d), the present dimeric cobalt complexes are capable of retaining commendable activities at low overpotentials (Scheme 1a). Overall, it was demonstrated that the trade-off between the TOF and *η* can be circumvented through the use of noninnocent ligands, such as **H**_**2**_**L3**, with assistance from the electronic effect.

## Conclusions

A family of catalysts (**1**–**3**) bearing bis(benzimidazole)pyrazolide ligands (**H**_**2**_**L1**–**H**_**2**_**L3**) is reported for use in the electrochemical WOR and to demonstrate how ligand design can be utilized to retain good water oxidation activity at low overpotentials. Collectively, the obtained data suggest that the ligand scaffold incorporated into catalysts **1**–**3** exhibits a noninnocent behavior, in which ligand-centered oxidation is an essential component in the 4e^–^/4H^+^ oxidation of H_2_O. Under alkaline conditions, the kinetic studies reveal that **3** has a different reaction pathway, TLS, and overpotential-determining step compared with complex **1**. The reaction intermediates and catalyst resting states in the WOR catalyzed by **3** were evaluated using X-ray crystallography, electrochemical, and spectroscopic methods. The spectroscopic evidence indicated that multielectron/multiproton transfer occurs during the reaction, and the characterized cobalt species provided a vision of the reaction mechanism. Supplemented by density functional theory investigations, we deduced that the TLS involved the nucleophilic attack of water on [(**L3**^**2•+**^)Co_2_^III^(μ-OH)]^4+^ (**3-IV**), which differs from the base-assisted deprotonation of dimeric [(**L1**^**2•+**^)Co_2_^III^(OH)]^4+^ (**I–IV**), which was identified as the TLS of the WOR catalyzed by **1**.

The present results highlight that the mutual influence between the electronic effect and the noninnocent character of the ligand on the MWOCs can manipulate the PT and ET steps, leading to different reaction mechanisms. Furthermore, Eyring analysis demonstrates that complexes **1**–**3** exhibit similar Gibbs free energies of activation. Moreover, a plot of log(TOF_max_) against *η* showed a plateau region wherein the TOF_max_ is independent of *η* over 400 mV, which was attributed to the different overpotential-determining steps of **1**–**3**. These observations provide valuable insights into catalyst design and are expected to lead to catalysts suitable for operation at low overpotentials.

## Experimental Section

### General Methods and Materials

All commercially purchased reagents were utilized in their original state. The required precursors and metal salts were purchased from Sigma-Aldrich, Acros Organics, and TCI. ^1^H NMR and ^13^C NMR spectra were recorded on a Bruker Avance III spectrometer (400 and 500 MHz for ^1^H NMR, 100 and 125.7 MHz for ^13^C NMR). ^1^H diffusion-ordered spectroscopy (DOSY) NMR spectra were recorded on a JEOL ECZ500R/S1 (500 MHz). Crystal evaluation and data collection were carried out on a Bruker X8 APEX Quazar SMART APEXII diffractometer with Mo Kα (λ = 0.71073 Å) radiation and the diffractometer and Rigaku XtaLAB Synergy R, DW system, HyPix-Arc 150 with Cu Kα (λ = 1.54184 Å) radiation and the diffractometer. ESI and EI mass spectra were recorded on a VARIAN 901-MS and a JEOL JMS-700 mass spectrometer, respectively. SEM images and EDX analysis were obtained with a JEOL JSM-7000 FESEM instrument equipped with an EDX detector. EPR spectra were measured on a Bruker EPR-plus.

### Synthesis of Ligands and Cobalt Complexes

The required precursors, ligands (**H**_**2**_**L**_**1**_, **H**_**2**_**L**_**2**_, **H**_**2**_**L**_**3**_), and complexes (**1**, **2**, **3**) were synthesized according to previously reported literature procedures with necessary modification. The details are given in the Supporting Information with characterization data. Complex **1** was obtained as a purple solid, yielding 140 mg (55%). Complex **2** was synthesized according to the procedure employed to synthesize complex **1**. The crude was purified on an alumina column and eluted with 50 mM KPF_6_ in acetone to obtain a purple solid, yielding 48 mg (23%). Complex **3** was synthesized using the procedure employed to synthesize complex **1**, but precursors were added reversely. The crude product could be purified on an alumina column and eluted with 50 mM KPF_6_ in acetone to obtain the purple solid, yielding 46 mg (40%).

### Electrochemistry

Electrochemical experiments were conducted by using a PalmSens4 potentiostat connected to a computer with PSTrace software, employing a three-electrode setup. The working electrode was a 3.0 mm diameter glassy carbon disk with a platinum wire as the auxiliary electrode and a silver wire pseudoreference as the reference electrode. The pseudoreference for MeCN solvents included 100 mM [NBu_4_][PF_6_] as a supporting electrolyte and 10 mM AgNO_3_. The working electrode was polished with 0.05 μm alumina on a wetted Buehler felt pad between each CV experiment. All voltammograms were internally referenced to the redox potential of Fc^+/0^. The laboratory temperature was maintained at 25 ± 2 °C.

### Cold-Spray Ionization Mass Spectrometry (CSI-MS)

The catalytically relevant cobalt intermediates were detected by a CSI-MS system, combining a home-built cold-spray ionization source and a linear ion trap mass spectrometer (LTQ XL, Thermo Fisher Scientific) to detect the unstable intermediates. The entire sample transport line was constructed with a dual-layered structure to regulate the temperature of the sample.

## Data Availability

The data supporting this study can be accessed in the published article and its accompanying Supporting Information.

## Supporting Information Available

General instrumentation methods and experimental details, including characterization data such as electrochemical analysis of the turnover frequencies, H_2_O_2_ quantitation, OCP measurements for the determination of *E*_H/H_2__^+^, thermodynamic potentials for *E*_O_2_/H_2_O_2__, half-wave potentials of the cobalt catalysts, kinetic data, ^1^H and ^13^C NMR shift values, ESI-MS/HRMS, ^1^H and ^13^C NMR spectra of the synthesized compounds, UV–visible spectra and single-crystal XRD data for complexes (PDF)
