# Supplementary material for: Manipulating the Rate and Overpotential for Electrochemical Water Oxidation: Mechanistic Insights for Cobalt Catalysts Bearing Noninnocent Bis(benzimidazole)pyrazolide Ligands
Source: ACS Org Inorg Au. 2024 Feb 14;4(3):306–18. doi: 10.1021/acsorginorgau.3c00061 (PMC11157513; doi:10.1021/acsorginorgau.3c00061)
Supplement: Supplementary file 1 — gg3c00061_si_001.pdf [file gg3c00061_si_001.pdf]

Supporting Information

Manipulating the Rate and Overpotential for Electrochemical Water Oxidation: Mechanistic Insights for Cobalt Catalysts Bearing Non-innocent Bis(benzimidazole)pyrazolide Ligands

Yu-Ting Wu,[†] Sharad V. Kumbhar,[†] Ruei-Feng Tsai,[†] Yung-Ching Yang,[‡] Wan-Qin Zeng,[‡] Yu-Han Wang,[†] Wan-Chi Hsu,[†] Yun-Wei Chiang,*[†] Tzuhsiung Yang,*[†] I-Chung Lu,*[‡] and Yu-Heng Wang*[†]

[†]Department of Chemistry, National Tsing Hua University, Hsinchu 30013, Taiwan

[‡]Department of Chemistry, National Chung Hsing University, Taichung 40227, Taiwan

Table of Contents:

1. General Considerations 4

2. Synthesis of Precursors, Ligands, and Cobalt Complexes 6

2a. Synthesis of Precursors 6

2b. Synthesis of Ligands. 10

2c. Synthesis of Dinuclear Cobalt Complexes 12

3. UV-Vis Spectral Measurements 14

3a. General considerations 14

3b. UV-Vis absorption spectra of Co complexes 14

3c. UV-Vis absorption spectra of ligands 14

3d. Stability test 15

4. Electrochemical Experiments 16

4a. General considerations 16

4b. Differential pulse voltammograms of Co complexes in anhydrous MeCN 17
4c. Normalized CV curves of WOR catalyzed by Co complexes 18
4d. Diffusion coefficient of Co complexes 18
5. Supporting Evidence for a Homogeneous Electrocatalyst 20
5a. Rinse test 20
5b. Consecutive CV scans 21
5c. Characterization of electrodes after consecutive CV scans 21
5d. Stability test 22
6. Controlled Potential Electrolysis (CPE) Experiments 24
6a. General considerations 24
6b. Oxygen evolution 24
6c. Selectivity of water oxidation by cobalt complexes 26
7. Rate Law Analysis of H_2O Oxidation Catalyzed by Co Complexes 30
7a. General considerations 30
7b. [Co]-dependence 30
7c. [H_2O]-dependence 32
8. Kinetic Isotope Effects (KIE) 33
8a. General considerations 33
8b. Cyclic voltammograms analysis of Co complexes in H_2O or D_2O 33
9. Determining Turnover Frequency (TOF) through Cyclic Voltammetry Measurements 34
9a. General procedure 34
10. Estimation of the Thermodynamic Reduction Potential of O_2/H_2O at Non-standard State 38
10a. Open-circuit potential (OCP) measurement for E_{H^+/H_2} in MeCN 38
10b. Estimation of E_{O_2/H_2O} based on OCP measurements of E_{H^+/H_2} 39
10c. Derivation of overpotential (η) 39
11. Spectroelectrochemical Study 41
General considerations 41
12. NMR Spectroscopic Experiments 42
12a. General considerations for solvent suppression 42
13. EPR Spectroscopic Experiments and Simulations 43
13a. General considerations 43
13b. EPR spectrum of **3-II** 43
14. Cold-spray ionization mass spectrometry (CSI-MS) 44
14a. General considerations 44

15. Eyring Analysis 47
15a. General considerations 47
15b. Eyring plot of H_2O oxidation catalyzed by Co complexes 47
16. Computational Study 51
16a. Methods 51
16b. Energies 51
16c. Coordinates 54
17. Compound Spectra 106
18. Crystallographic Data 141
18a. Single crystal X-ray crystallography for **2**. 141
18b. Single crystal X-ray crystallography for **3**. 152
18c. Single crystal X-ray crystallography for **1-I**. 165
18d. Single crystal X-ray crystallography for **3-I**. 175
19. References 187

1. General Considerations

A. Materials and Instrumentations

All commercially available reagents, including solvents, were used as received, except where otherwise noted. 1*H*-pyrazole-3,5-dicarboxylic acid, 1,2-dimethoxybenzene, cobalt chloride hexahydrate, 2,2':6',2"-terpyridine, tetrabutylammonium bromide, sodium periodate, and amine substrates were purchased from Combi-Blocks, Acros, TCI, Matrix Scientific. ^{1}H-NMR and ^{13}C-NMR spectra were recorded on a Bruker Avance III spectrometer (400 MHz and 500 MHz for ^{1}H-NMR, 100 MHz and 125.7 MHz for ^{13}C-NMR). Chemical shifts are reported in parts per million (ppm), referenced to $CDCl_3$ at 7.26 ppm (^{1}H) and 77.16 ppm (^{13}C), CD_3CN at 1.94 ppm (^{1}H) and 1.32 ppm, 118.26 ppm (^{13}C), DMSO-d_6 at 2.50 ppm (^{1}H) and 39.52 ppm (^{13}C), and or DMF-d_7 at 2.75 ppm, 2.92 ppm, 8.03 ppm (^{1}H). Multiplicities are described using the following abbreviations: s = singlet, bs = broad singlet, d = doublet, dd = doublet of doublet, t = triplet, m = multiplet. NMR spectra were plotted with MestReNova v14.2.0-26256 (MestreLab Research S. L. 2020). ^{1}H Diffusion-Ordered Spectroscopy (DOSY) NMR spectra were recorded on a JEOL ECZ500R/S1 (500 MHz). UV-visible spectra were recorded on a Varian Cary 60 spectrometer. The crystal evaluation and data collection were performed on Bruker X8 APEX Quazar SMART APEXII diffractometer with Mo Kα (λ = 0.71073 Å) radiation and the diffractometer, and Rigaku XtaLAB Synergy R, DW system, HyPix-Arc 150 with Cu Kα (λ = 1.54184 Å) radiation and the diffractometer. ESI (electron spray ionization) mass spectra were recorded on a VARIAN 901-MS (FT-ICR Mass) mass spectrometer. EI (electron ionization) mass spectra were recorded on a JEOL JMS-700 mass spectrometer. The mass spectra were acquired by a linear ion trap mass spectrometer (Thermo Scientific LTQ XL) combined with a home-built cold spray ionization (CSI) source. The samples were delivered 3 µL/min to the CSI source, and each spectrum was acquired 60 scans in positive mode. All the chemical compositions in the mass spectra were confirmed with the theoretical estimation of isotopic distribution. Scanning electron microscopy (SEM) images and energy-dispersive X-ray spectroscopy (EDX) analysis were obtained with a JEOL JSM-7000 FESEM instrument equipped with an EDX detector. Electron Paramagnetic Resonance (EPR) spectra were measured on a Bruker EPR- plus.

B. Cyclic Voltammetry

All electrochemical experiments are performed using the PalmSens4 potentiostat interfaced to a computer with PSTrace software, employing a three-electrode setup. The working electrode consisted of a glassy carbon disk (3.0 mm diameter), with a platinum wire as the auxiliary electrode and a silver wire pseudoreference used as the reference electrode. The silver wire pseudoreference for MeCN solvents contains 100 mM [n-Bu_4N][PF_6] as a supporting electrolyte and 10 mM

$AgNO_3$. In all cyclic voltammetry (CV) experiments conducted in this work, the working electrode was carefully polished between each voltammogram using 0.05 micron alumina powder on a wetted Buehler felt pad. All voltammograms were internally referenced to the redox potential of ferrocene/ferrocenium ($Fc^{+/0}$). The laboratory temperature was typically maintained at 25 ± 2 °C.

2. Synthesis of Precursors, Ligands, and Cobalt Complexes

2a. Synthesis of Precursors

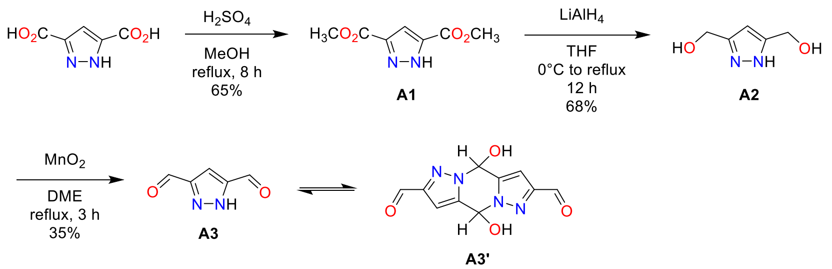

Scheme S1. Synthetic route to 1*H*-pyrazole-3,5-dicarbaldehyde (**A3**).

Synthesis of dimethyl 1*H*-pyrazole-3,5-dicarboxylate (A1)

Precursor **A1** was synthesized according to the literature protocol.[1, 2] 1*H*-pyrazole-3,5-dicarboxylic acid (10.11 g, 64.77 mmol) was placed into a round bottom flask. Then, 500 mL of methanol was added to the flask, and the mixture was cooled in an ice bath. Sulfuric acid (10 mL) was added dropwise using an addition funnel to adjust the pH to 1. The reaction mixture was refluxed in a metal beads bath for 8 hours. After refluxing, the reaction mixture was cooled to room temperature. The solution was concentrated to approximately 60 mL using rotary evaporation. A saturated sodium carbonate (Na_2CO_3) solution was added while stirring to adjust the pH to 8. Once a white solid precipitated, it was filtered and washed with hexanes at least three times using 100 mL of hexanes for each wash. The resulting white solid was then dried under a high vacuum. ^{1}H NMR ($CDCl_3$, 400 MHz): δ 7.34 (s, 1H), 3.95 (s, 6H). ^{13}C{^{1}H} NMR (101 MHz, $CDCl_3$): δ 160.9, 140.0, 111.5, 52.5.

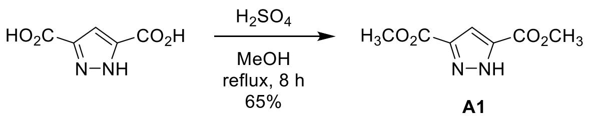

Synthesis of (1*H*-pyrazole-3,5-diyl) dimethanol (A2)

Precursor **A2** was synthesized according to the literature protocol.[2, 3] In a dry round-bottom flask, lithium aluminum hydride (0.36 g, 9.4246 mmol) was placed, then dry THF was added at 0 °C to make a slurry. Compound **A1** (1.0 g, 4.7123 mmol) was dissolved in THF (20 mL) and added dropwise to the slurry at 0 °C under a nitrogen atmosphere. The suspension mixture was heated under reflux in a metal beads bath for 12 hours. Completion of the reaction was confirmed by TLC analysis. The reaction mixture was cooled to 0 °C in an ice bath, and the minimum amount of water was added to quench the reaction mixture. Then, the mixture was concentrated on a

rotavapor. The white solid was suspended in hot methanol, filtered when still hot, and washed with hot methanol. The filtrate obtained was treated with CO_2 (g) for 15 min, resulting in the appearance of a white precipitate. The precipitate was filtered, and the filtrate was further concentrated to yield a white solid. Yield: 0.44 g, 73%. ^{1}H NMR (DMSO-d_6, 400 MHz): δ 6.42 (s, 1H), 4.55 (s, 4H); ^{13}C{^{1}H} NMR (DMSO-d_6, 101 MHz): δ 149.8, 103.0, 55.1. ESI-MS (*m*/*z*): [**A2** – H]$^-$ calculated for $C_5H_7N_2O_2$ 127.0507; found: 127.0510.

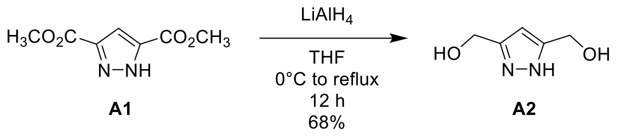

Synthesis of 1*H*-pyrazole-3,5-dicarbaldehyde (A3)

Precursor **A3** was synthesized according to the literature protocol.[2, 4] MnO_2 (3.50 g, 40.28 mmol) was added portion-wise to a refluxing solution of dimethoxymethane (DME) (20 mL) containing compound **A2** (0.43 g, 3.36 mmol). The reaction mixture was refluxed in a metal beads bath for 3 h, after which the hot mixture was filtered over celite and washed with hot MeOH (3 × 20 mL). Charcoal was added to the filtrate, filtered over celite, and rewashed with hot MeOH. The filtrate was evaporated to dryness and recrystallized from *i*-PrOH to afford the title compound as a white solid. NMR of **3A** at room temperature shows two sets of signals corresponding to a mixture of the dimeric species monomer and diastereomers (**3A'**). According to the literature report, the equilibrium between 3 and 3A' is affected by the temperature.[4, 5] Yield: 0.14 g, 34%. ^{1}H NMR (DMSO-d_6, 400 MHz): δ (ppm) = 9.94 (s, 2H), 7.48 (s, 1H); ^{13}C{^{1}H}NMR (DMSO-d_6, 101 MHz): δ 184.1, 147.2, 109.3. ESI-MS (*m*/*z*): [**A3** – H]$^-$ calculated for $C_5H_3N_2O_2$: 123.0194; found: 123.0197.

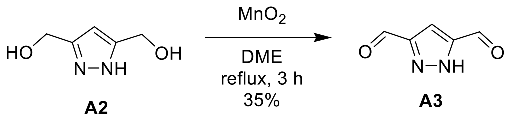

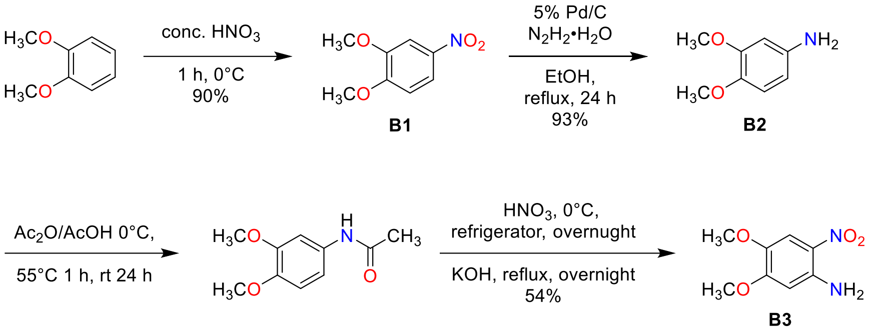

Scheme S2. Synthetic route to 4,5-dimethoxy-2-nitroaniline (**B3**).

Synthesis of 1,2-dimethoxy-4-nitrobenzene (B1)

Precursor **B1** was synthesized according to the literature protocol.[6] Veratrole (1,2-dimethoxybenzene) (1 g, 7.2 mmol) was added dropwisely to a stirred solution of HNO_3 (1 mL) and water at 0 °C. After the addition was complete, the mixture was stirred for 1 hr. Then, crushed ice was added to the reaction mixture, forming a yellow precipitate. The yellow precipitate was collected by filtration and dried under vacuum to obtain a yellow solid. Yield: 1.2 g, 90%. ^{1}H NMR ($CDCl_3$, 400 MHz): δ 7.92 (dd, *J* = 8.9, 2.7, 1H), 7.74 (d, *J* = 1.7 Hz, 1H), 6.91 (d, *J* = 8.9 Hz, 1H), 3.98 (s, 3H), 3.96 (s, 3H); ^{13}C{^{1}H} NMR ($CDCl_3$, 101 MHz): δ 154.5, 148.8, 141.4, 117.7, 109.8, 106.3, 56.4, 56.2.

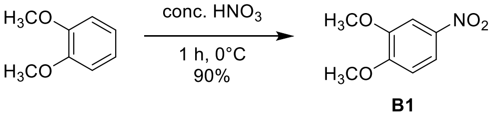

Synthesis of 3,4-dimethoxyaniline (B2)

Precursor **B2** was synthesized according to a patent procedure,[7] obtained as a brown solid and yielded 0.88 g, 93%. ^{1}H NMR ($CDCl_3$, 400 MHz) δ 6.69 (d, *J* = 8.4 Hz, 1H), 6.30 (d, *J* = 2.6 Hz, 1H), 6.22 (dd, *J* = 8.4, 2.7 Hz, 1H), 3.81 (s, 3H), 3.79 (s, 3H), 3.35 (s, 2H); ^{13}C{^{1}H} NMR ($CDCl_3$, 101 MHz) δ 149.9, 142.2, 140.7, 113.2, 106.4, 100.8, 56.7, 55.7.

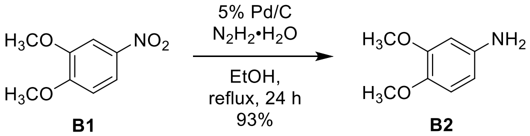

Synthesis of 4,5-dimethoxy-2-nitroaniline (B3)

Precursor **B3** was synthesized according to a patent procedure,[8] obtained as a yellow crystalline solid and yielded 0.7 g, 54 %. ^{1}H NMR ($CDCl_3$, 400 MHz): δ 7.54 (s, 1H), 6.16 (s, 3H), 3.91 (s, 3H), 3.86 (s, 3H); ^{13}C{^{1}H} NMR ($CDCl_3$, 101 MHz): δ 157.0, 142.8, 141.6, 124.5, 106.5, 99.1, 56.4, 56.3.

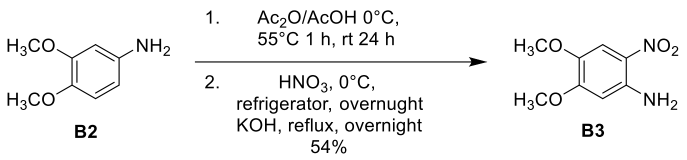

Synthesis of [Co(tpy)Cl$_2$]

[Co(tpy)Cl$_2$] was synthesized according to the literature protocol.[9] A solution of cobalt (II) chloride hexahydrate (1.13 g, 4.74 mmol) in boiling ethanol (25 mL) was added to a solution of 2,2';6',2"-terpyridine (1.15 g, 4.93 mmol) in boiling ethanol (25 mL). The mixture was refluxed in a metal beads bath for 15 min. After cooling at room temperature, the green crystalline compound was separated, washed with ethanol and ether, and dried under vacuum to obtain the desired green solid yielding 1.64 g, 95%.

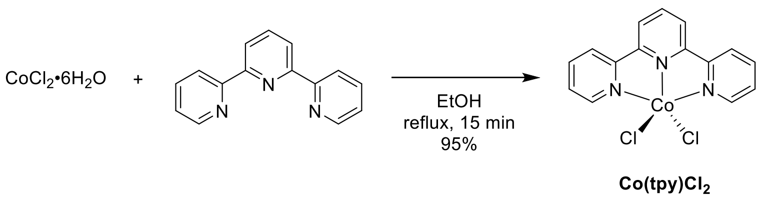

Synthesis of tetrabutylammonium hydroxide [NBu$_4$][OH]

[NBu$_4$][OH] was synthesized according to a literature procedure,[10] obtained as a white solid and yielded 0.72 g, 84%. ^{1}H NMR (500 MHz, CD_3CN): δ 3.14 – 3.06 (m, 8H), 1.61 (m, J = 7.7 Hz, 8H), 1.35 (m, J = 7.4 Hz, 8H), 0.97 (t, J = 7.4 Hz, 12H). ^{13}C{^{1}H} NMR (126 MHz, CD_3CN): δ 59.3, 24.3, 20.3, 13.7. ESI-MS (m/z): $[NBu_4]^+$ calculated for $C_{16}H_{36}N_1$: 242.2847; found: 242.2848.

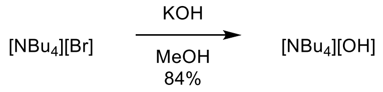

Synthesis of $[NBu_4][IO_4]$

$[NBu_4][IO_4]$ was synthesized according to a literature procedure,[11] obtained as a white crystalline solid and yielded 0.84 g, 80%. ^{1}H NMR (500 MHz, CD_3CN): δ 3.12 – 3.05 (m, 3H), 1.66 – 1.55 (m, 3H), 1.35 (m, *J* = 7.4 Hz, 3H), 0.97 (t, *J* = 7.4 Hz, 4H); $^{13}C\{^1H\}$ NMR (126 MHz, CD3CN): δ 59.3, 24.3, 20.3, 13.8. ESI-MS (*m*/*z*): $[NBu_4]^+$ calculated for $C_{16}H_{36}N_1$: 242.2848; found: 242.2791; $[IO4]^-$ calculated for I_1O_4: 190.8841; found: 190.8937.

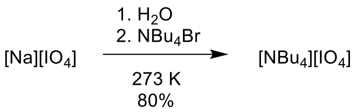

2b. Synthesis of Ligands.

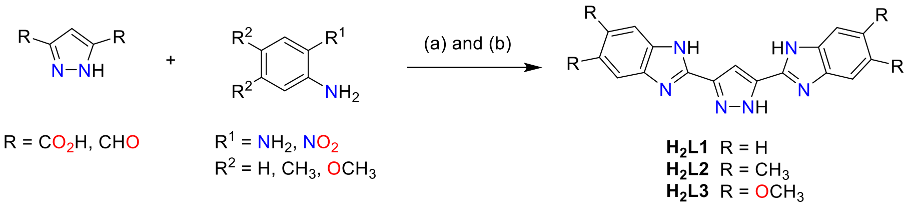

Scheme S3. Synthesis of ligands **H_2L1**–**H_2L3**. Reaction conditions: (a) H_3PO_4, 200 °C 2 h, 250 °C 2 h (**H_2L1** and **H_2L2**); (b) $Na_2S_2O_4$, EtOH/H_2O, 80 °C, 5 h (**H_2L3**).

Synthesis of H_2L1

Ligand **H_2L1** was synthesized according to a literature procedure,[12] obtained as an off-white solid and yielded 0.85 g, 43%. ^{1}H NMR (400 MHz, DMSO-d_6,): δ 14.40 (bs, 1H), 13.08 (bs, 2H), 7.61 – 7.60 (m, 4H), 7.54 (s, 1H), 7.23 (m, 4H); $^{13}C\{^1H\}$ NMR (126 MHz, DMSO-d_6): δ 162.2, 146.5, 144.2, 143.4, 142.7, 135.1, 134.3, 122.6, 121.7, 118.6, 111.4, 103.4. ESI-MS (*m*/*z*): [**H_2L1** – 2H + 2Na]$^+$ calculated for $C_{17}H_{11}N_6Na_2$: 345.0840; found: 345.0829. NMR spectral and ESI-MS data were consistent with those available in the literature.

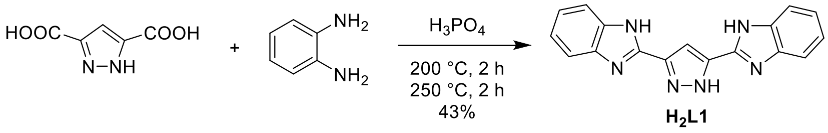

Synthesis of H_2L2

Ligand **H_2L2** was synthesized according to the procedure employed to synthesize **H_2L1**, obtained as a white solid and yielded 1.11 g, 44%. ^{1}H NMR (400 MHz, DMF-d_7): δ 7.45 (s, 1H), 7.44 – 7.42 (m, 4H), 2.38 – 2.36 (s, 12H); ^{13}C{^{1}H} NMR (126 MHz, DMF-d_7): δ 146.0, 145.6, 143.4, 141.7, 139.3, 132.0, 116.4, 107.9, 104.1, 20.5. EI-MS (*m*/*z*): [**H_2L2**]$^+$ calculated for $C_{21}H_{20}N_6$: 356.1749; found: 356.1748.

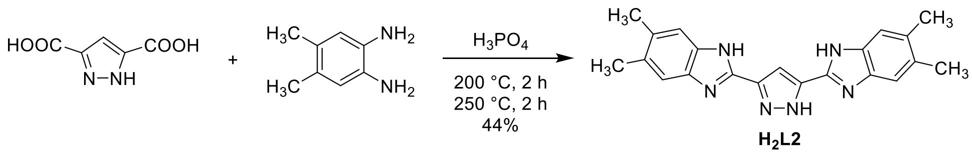

Synthesis of H_2L3

Compound **A3** (0.11 g, 0.84 mmol) and compound **B3** (0.34 g, 1.72 mmol) were suspended in EtOH (3.3 mL). An aqueous solution of disodium dithionite (0.89 g, 5.09 mmol, 5 mL) was added to the suspension, after which the reaction mixture was heated to 70 °C in a metal beads bath for 5 h and cooled to room temperature. The orange-yellow precipitate was filtered and washed with H_2O and cold EtOH to afford the pale yellow solid. Yield: 0.16 g, 46%. ^{1}H NMR (400 MHz, DMSO-d_6): δ 14.07 (bs, 1H), 12.77 (bs, 2H), 7.33 (s, 1H), 7.08 (m, 4H), 3.73 (s, 12H). ^{13}C{^{1}H} NMR (126 MHz, DMSO-d_6): δ 147.2, 142.3, 136.1, 130.4, 102.4, 97.4, 55.9. ESI-MS (*m*/*z*): [**H_2L3** – H]$^-$ calculated for $C_{21}H_{19}N_6O_4$: 419.1467; found: 419.1466.

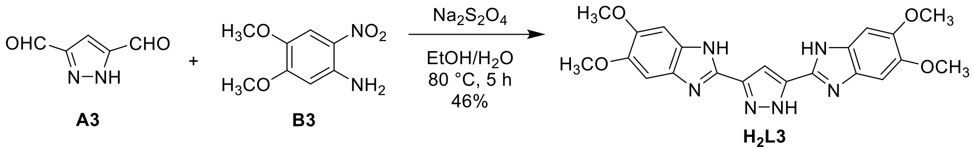

2c. Synthesis of Dinuclear Cobalt Complexes

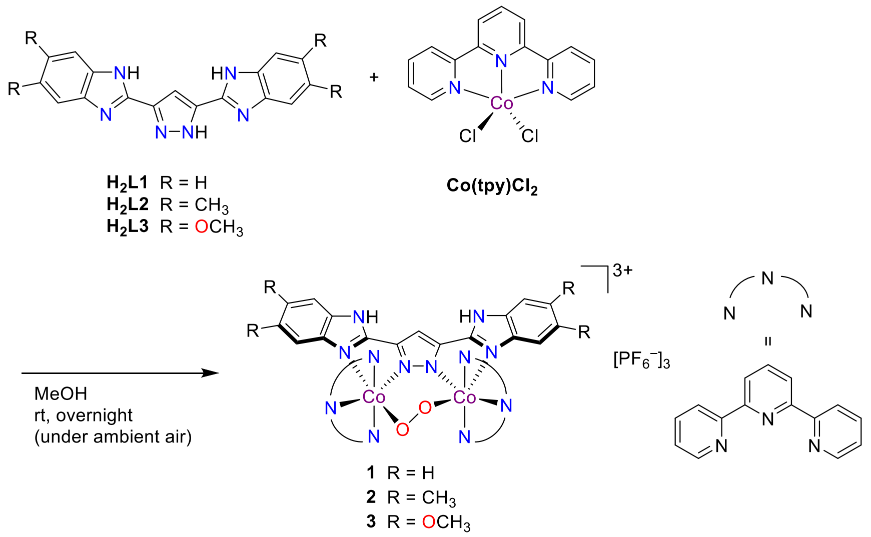

Scheme S4. Synthesis of dinuclear cobalt complexes **1**–**3**.

Synthesis of cobalt complex 1

Complex **1** was synthesized according to a literature procedure,[13] obtained as a purple solid and yielded 140 mg, 55%. ^{1}H NMR (400 MHz, CD_3CN): 8.65 (t, J = 8.0 Hz, 2H), 8.58 – 8.52 (m, 5H), 8.47 (s, 2H), 8.20 (s, 4H), 8.10 (s, 2H), 7.94 (s, 2H), 7.67 (d, J = 8.2 Hz, 2H), 7.58 (s, 2H), 7.51 (s, 2H), 7.31 (s, 2H), 7.24 (t, J = 7.8 Hz, 2H), 6.96 (t, J = 7.8 Hz, 2H), 6.87 (s, 2H), 6.03 (d, J = 8.3 Hz, 2H). NMR spectral data were consistent with those available in the literature.

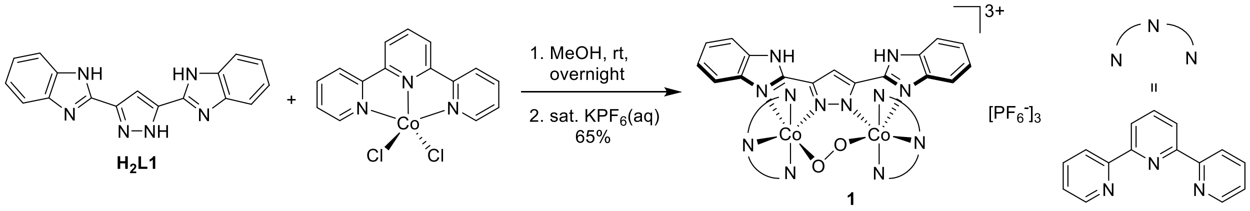

Synthesis of cobalt complex 2

Complex **2** was synthesized according to the procedure employed to synthesize complex **1**. The crude was purified on an alumina column and eluted with 50 mM KPF_6 in acetone to obtain the purple solid. Yield: 48 mg, 23%. ^{1}H NMR (500 MHz, DMSO-d_6): δ 8.89 – 8.83 (m, 6H), 8.50 (d,

J = 7.2 Hz, 4H), 8.13 – 8.08 (m, 5H), 7.61 – 7.29 (m, 11H), 5.34 (s, 2H), 2.11 (s, 6H), 1.85 (s, 6H); $^{13}C\{^1H\}$ NMR (126 MHz, DMSO-d_6): δ 157.1, 154.9, 152.5, 142.1, 140.8, 140.3, 129.6, 128.1, 123.9, 123.5, 117.3, 111.2, 103.1, 20.2, 19.7. UV-Visible (MeCN): λ_{max} = 531 nm (ε_{max} = 1.1 × 10^4 M^{-1} cm^{-1}), 325 nm (ε_{max} = 7.3 × 10^4 M^{-1} cm^{-1}), 284 nm (ε_{max} = 5.4 × 10^4 M^{-1} cm^{-1}).

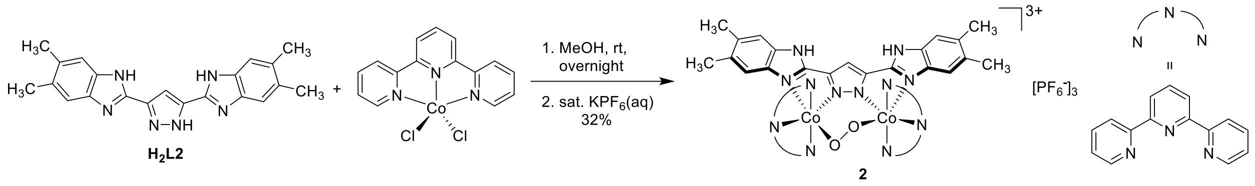

Synthesis of cobalt complex 3

Complex **3** was synthesized using the procedure employed to synthesize complex **1**, but precursors were added reversely. The crude could be purified on an alumina column and eluted with 50 mM KPF_6 in acetone to obtain the purple solid. Yield = 46 mg (40%).^1H NMR (500 MHz, CD_3CN): δ 8.64 (t, J = 8.0 Hz, 2H), 8.60 (s, 2H), 8.49 (s, 2H), 8.36 (s, 1H), 8.20 – 8.24 (m, 4H), 8.13 (s, 2H), 7.95 (s, 2H), 7.61 (s, 2H), 7.53 (s, 2H), 7.29 (s, 2H), 7.14 (s, 2H), 6.89 (s, 2H), 5.24 (s, 2H), 3.78 (s, 6H), 3.33 (s, 6H); $^{13}C\{^1H\}$ NMR (126 MHz, CD_3CN): δ 158.7, 158.4, 156.7, 156.5, 154.2, 152.6, 151.6, 150.0, 149.5, 145.5, 143.8, 142.8, 142.4, 134.2, 130.0, 129.7, 128.7, 125.6, 125.0, 118.3, 107.0, 97.3, 96.3, 56.9, 56.1. UV-Visible (MeCN): λ_{max} = 530 nm (ε_{max} = 1.04 × 10^4 M^{-1} cm^{-1}), 330 nm (ε_{max} = 6.8 × 10^4 M^{-1} cm^{-1}), 283 nm (ε_{max} = 4.6 × 10^4 M^{-1} cm^{-1}).

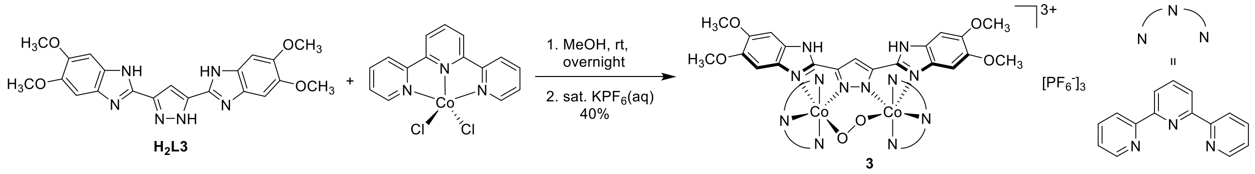

3. UV-Vis Spectral Measurements

3a. General considerations

UV-Vis spectroscopy measurements were conducted at a temperature of 25 °C using a 1 cm path quartz cell with a Cary 60 spectrometer.

3b. UV-Vis absorption spectra of Co complexes

The UV-vis absorption spectra of Co complexes in anhydrous MeCN are shown in Figure S1.

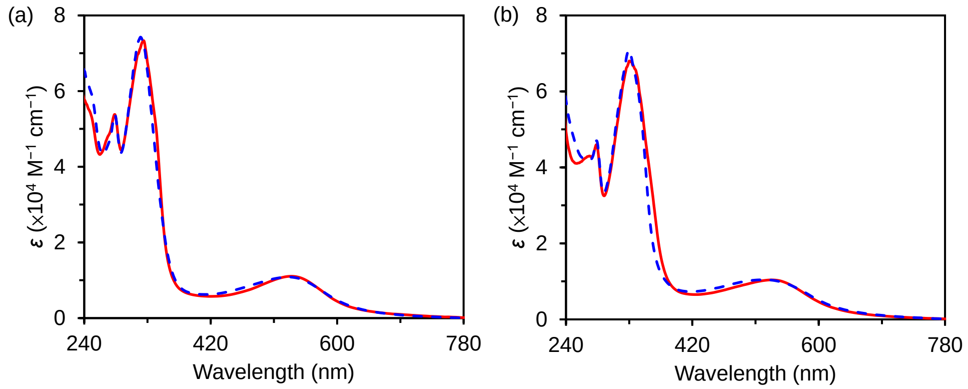

Figure S1. UV-Vis absorption spectrum of Co complexes in the absence (red solid) or presence (blue dash) of 10 mM NaOH in anhydrous MeCN. (a) **2** and (b) **3**.

3c. UV-Vis absorption spectra of ligands

The UV-Vis absorption spectra of ligands in anhydrous DMF are shown in Figure S2.

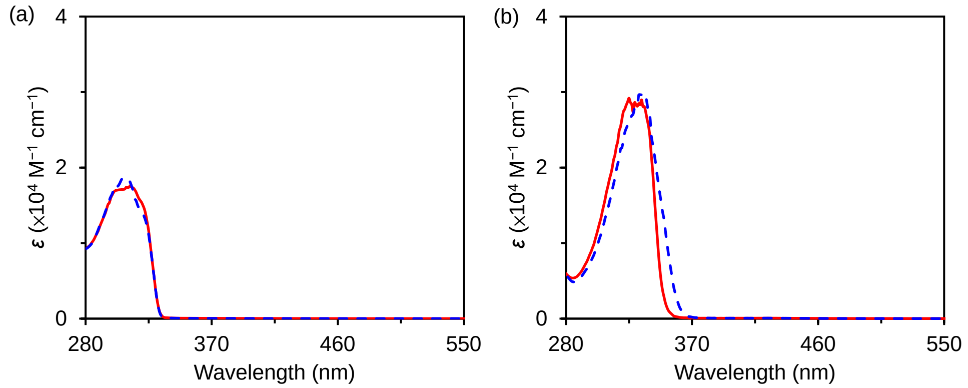

Figure S2. UV-Vis absorption spectrum of ligands in the absence (red solid) or presence (blue dash) of 10 mM NaOH in anhydrous DMF. (a) $\mathbf{H_2L2}$ and (b) $\mathbf{H_2L3}$.

3d. Stability test

The absorbance intensities of the complex are linearly dependent on the concentration of dinuclear cobalt complexes within the range from 1.25 to 25 μM (Figure S3), suggesting that the complex exists as a single species in a dinuclear form in solution, in other words, theses dinuclear cobalt complexes are stable under these conditions.

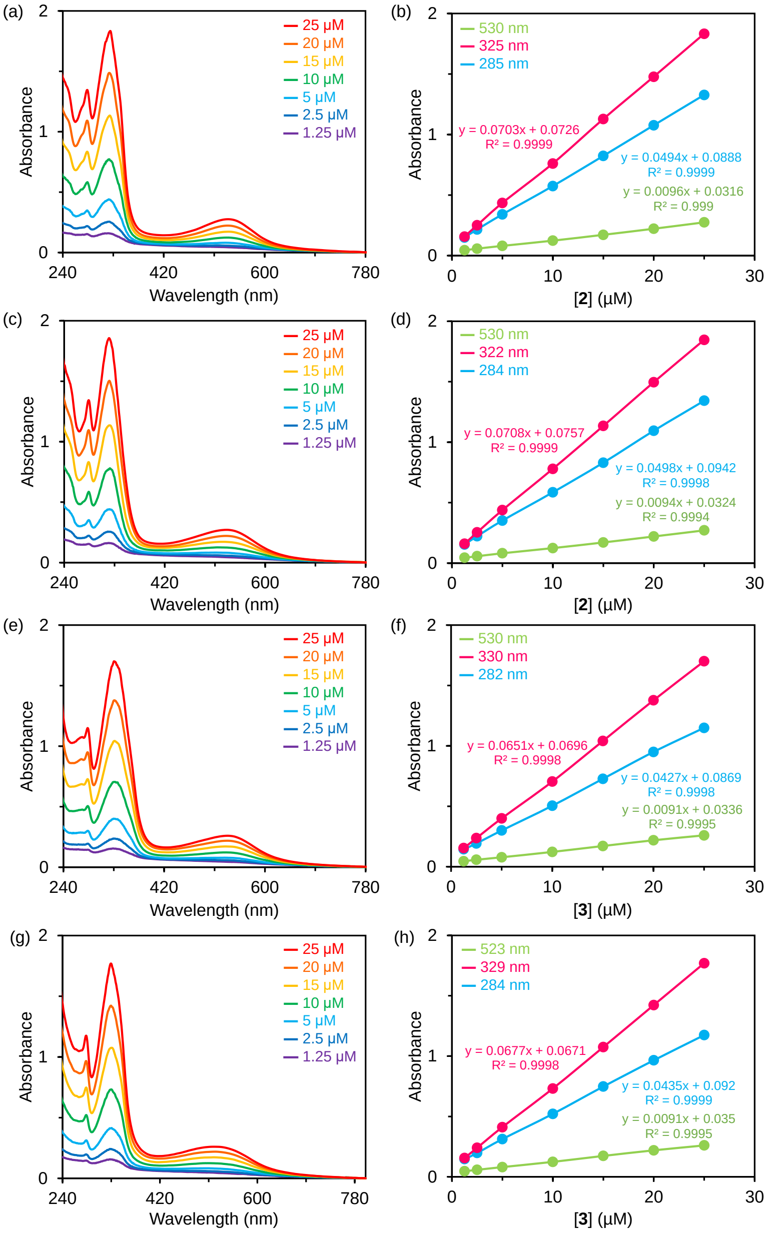

Figure S3. UV-Vis absorption spectra of various concentrations of Co complexes **2** (a) and **3** (e) in anhydrous MeCN, **2** (c), and **3** (g) in the presence of 10 mM NaOH in anhydrous MeCN. Plots of absorbances as a function of concentrations of Co complexes **2** (b) and **3** (f) in anhydrous MeCN, **2** (d), and **3** (h) in the presence of 10 mM NaOH in anhydrous MeCN.

4. Electrochemical Experiments

4a. General considerations

All cyclic voltammogram (CV), differential pulse voltammogram (DPV), and other electrochemical experiments were performed using a three-electrode setup, which included a glassy carbon (GC) working electrode (3.0 mm diameter, WE), a platinum (Pt) wire counter electrode (CE), and a non-aqueous reference electrode (RE) containing 0.01 M Ag/$AgNO_3$ (Figure S4). The supporting electrolyte used for all electrochemical investigations was 0.1 M tetrabutylammonium hexafluorophosphate ([NBu_4][PF_6]). The ferrocene/ferrocenium redox couple ($Fc^{+/0}$) in a MeCN solution was established as 0.1 V relative to Ag/$AgNO_3$ to determine the half-wave potential of cobalt complexes. Subsequently, the half-wave potentials of the cobalt complexes were converted and referenced to $Fc^{+/0}$ for the entire study duration. All CV experiments were conducted at a scan rate of 100 mV s^{-1} unless otherwise specified. DPV measurements were performed with a pulse amplitude of 50 mV, a pulse period of 0.3 s, an increment of 10 mV, and a scan rate of 10 mV s^{-1}.

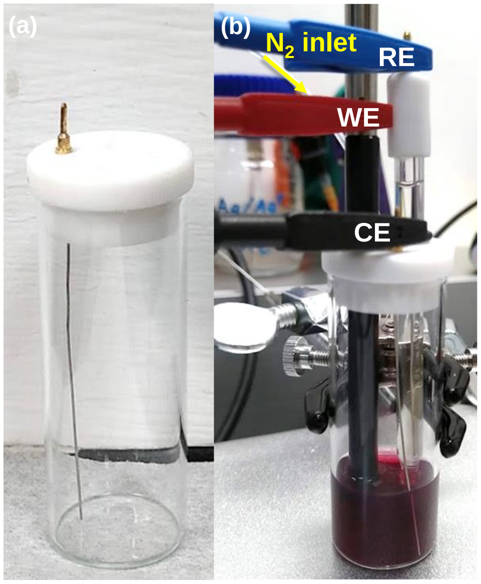

Figure S4. (a) Glass vial used for voltammetry measurements. (b) Setup for electrochemical experiments.

4b. Differential pulse voltammograms of Co complexes in anhydrous MeCN

For the DPV studies of Co complexes under anaerobic conditions, the solution of 0.4 mM Co complex with 0 or 10 mM NaOH and 0.1 M $[NBu_4][PF_6]$ in 5 mL of anhydrous MeCN (Figure S5 and Table S1)

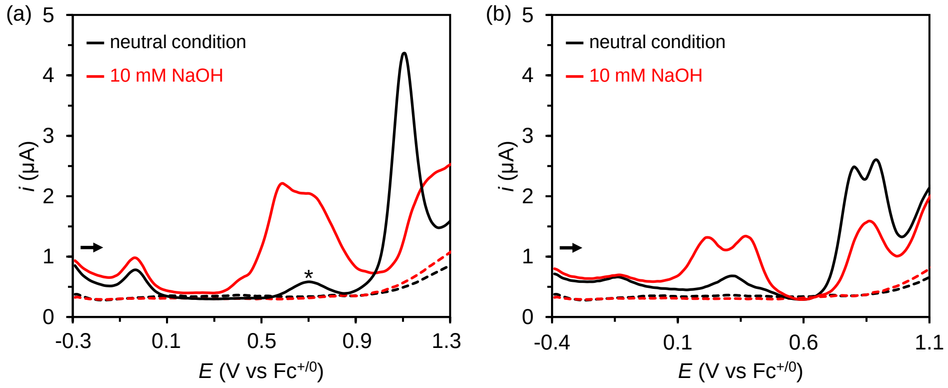

Figure S5. DPVs of 0.4 mM Co complexes **2** (a) and **3** (b) in the absence or presence of 10 mM NaOH in anhydrous MeCN. Solid trace: 0.4 mM Co complex in the absence or presence of NaOH in MeCN; dashed trace: blank with no complex in the absence or presence of 10 mM NaOH in anhydrous MeCN. DPVs were plotted using the IUPAC convention.

Table S1. A summary of half-wave potentials of Co complexes (**1**–**3**) in neutral and alkaline conditions in anhydrous MeCN.

	$E_{1/2}$ (V vs. $Fc^{+/0}$)	
Co Complexes	neutral	alkaline
**1**[a]	1.09	0.67, 0.82
**2**[b]	1.01	0.59, 0.73
**3**[c]	0.32, 0.80, 0.86	0.22, 0.37, 0.89

a: 10 mM NaOH; b: 10 mM NaOH; c: 10 mM NaOH with 1 mM 15-crown-5. The calculated redox potentials of **2** and **3** under alkaline conditions are listed in Table 2 of the main manuscript.

4c. Normalized CV curves of WOR catalyzed by Co complexes

Cyclic voltammograms (CVs) were conducted by a 5 mL MeCN solution of Co complexes (0.4 mM) containing H_2O (5 M; 9%, v/v) and $[NBu_4][PF_6]$ (0.1 M) in the absence or presence of NaOH (10 mM) at various scan rates (from 0.05 to 1.0 V s^{-1}). The normalized catalytic current, represented by the current divided by the square root of scan rate, $i/v^{1/2}$), exhibited a decrease with increasing scan rates, indicating that the current is associated with the catalytic process (Figure S6).

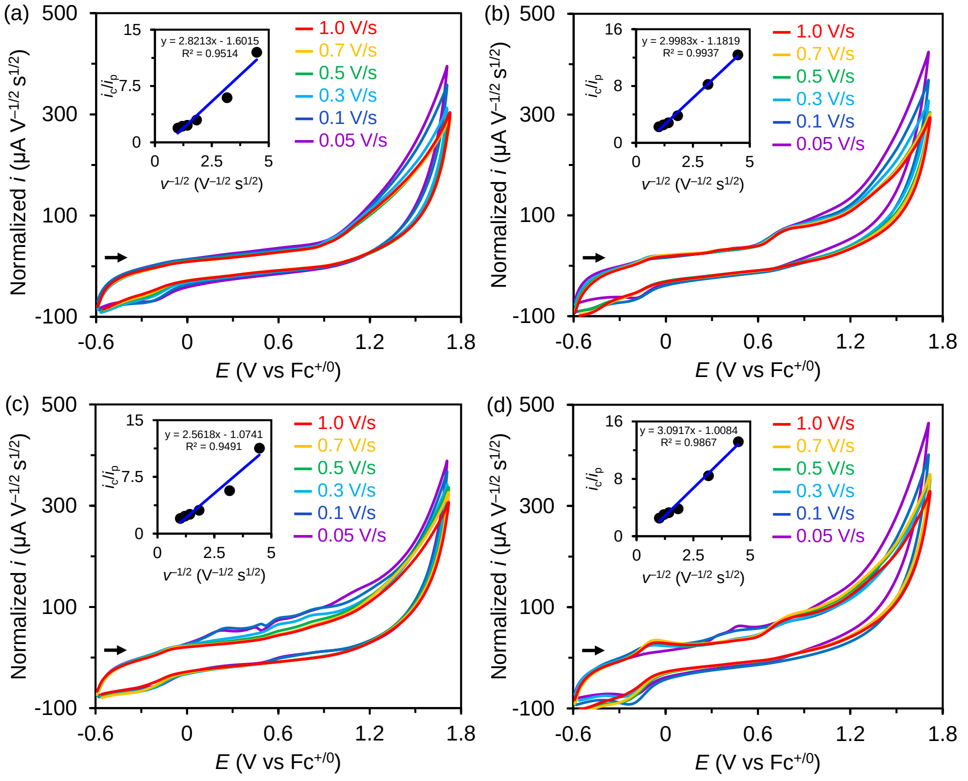

Figure S6. Normalized-current CVs (i.e., current divided by the square root of scan rate) of 0.4 mM Co complexes **2** and **3**. (a) and (b) 5 M H_2O (9%, v/v); (c) and (d) 5 M H_2O (9%, v/v) and 10 mM NaOH; (e) and (f) 10 M H_2O (18%, v/v) at various scan rates. Inset: plots of i_c/i_p at 1.50 V vs. $v^{-1/2}$. CVs were plotted using the IUPAC convention.

4d. Diffusion coefficient of Co complexes

The Diffusion-Ordered NMR Spectroscopy (DOSY) method was employed to determine the diffusion coefficients of **1–3** in this study (

Table S2). The obtained diffusion coefficient was subsequently used to derive the turnover frequency (TOF) for catalysts (**1–3**) according to equation 2 in the manuscript (see Section 9).

DOSY aims to differentiate NMR signals from a mixture of molecules by their differences in molecular translational diffusion. DOSY has found wide applications in routine NMR analysis of chemical mixtures for chemical identification and quantification.

The diffusion coefficient is affected by molecular size and shape with additional factors from temperature, solvent viscosity, etc., according to the Debye-Einstein equation,

$$D = \frac{k_B T}{f} = \frac{k_B T}{6\pi\mu R_0} \qquad \text{Equation S1}$$

where k_B is the Boltzmann's constant, T is the absolute temperature (K), f is the Friction coefficient of the solute, μ is the solvent viscosity, and R_0 is the solute radius.

Table S2. A summary of the diffusion coefficient of Co complexes (**1–3**) based on the Diffusion-Ordered NMR Spectroscopy (DOSY) in CD_3CN.

Co complex	D ($cm^2\ s^{-1}$)
**1**	9.37×10^{-6}
**2**	1.79×10^{-5}
**3**	1.64×10^{-5}

5. Supporting Evidence for a Homogeneous Electrocatalyst

5a. Rinse test

Consecutive CVs were acquired using a freshly polished glassy carbon (GC) electrode immersed in a solution of 0.1 M $[NBu_4][PF_6]$ in MeCN, 0.4 mM catalyst, 0 or 10 mM NaOH, and 5 M H_2O (9% v/v). After around 1 h of continuous CV scans, the GC electrode was rinsed with pure MeCN and subjected to an identical scan in an aqueous solution (Figure S7). The rinse test aimed to investigate whether the cobalt complexes acted solely as a molecular catalyst for water oxidation or merely served as a precursor for the electrodeposition of cobalt oxides under experimental conditions.

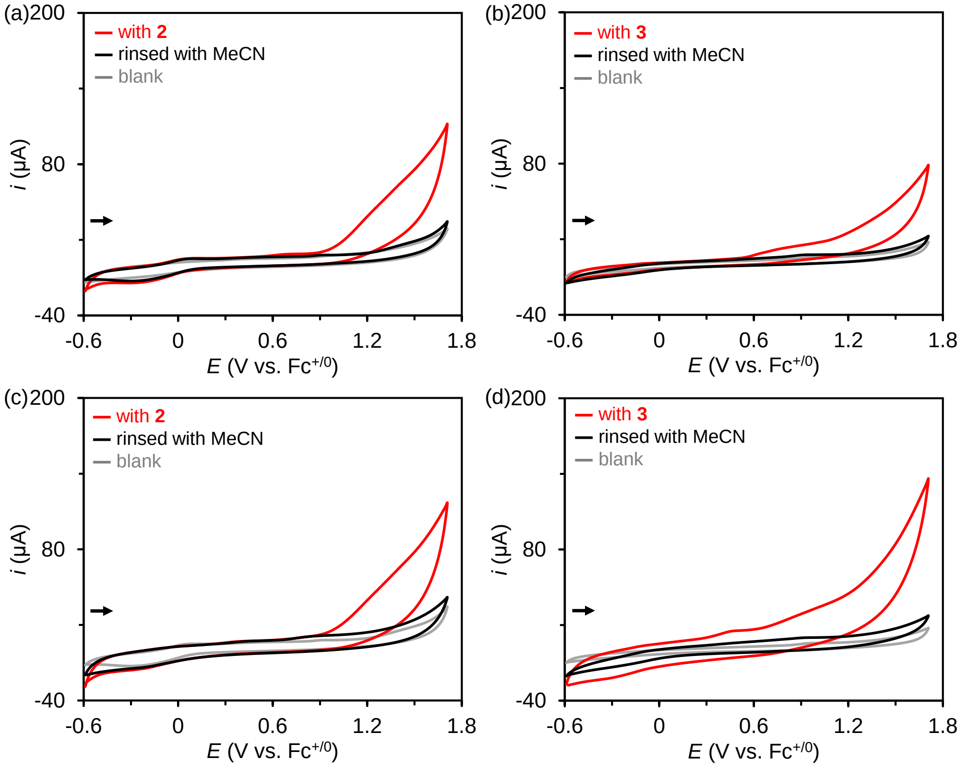

Figure S7. After continuous CV scans of around 1 h (red), the GC electrode was carefully removed from the solution and washed with MeCN. Subsequently, the rinsed GC electrode (unpolished; black) was cycled in a fresh solution of 0.1 M $[NBu_4][PF_6]$/MeCN without catalyst (scan rate of 100 mV s^{-1}). The grey trace corresponds to a blank experiment conducted in MeCN without a catalyst. (a) 0.4 mM of **2** with 5 M H_2O (9% v/v); (b) 0.4 mM of **3** with 5 M H_2O (9% v/v); (c) 0.4 mM of **2** with 5 M H_2O (9% v/v) and 10 mM NaOH; (d) 0.4 mM of **3** with 5 M H_2O (9% v/v) and 10 mM NaOH. CVs were plotted using the IUPAC convention.

5b. Consecutive CV scans

CV studies in this work employed a scan rate of 100 mV s^{-1} unless specified otherwise. A series of successive CV scans of Co complex **2** and **3** (0.4 mM) revealed a slight decrease in peak current during the first few scans. However, it remained relatively constant over 250 scans for approximately 3 hours. This observation suggests that the electrochemical decomposition and deposition of complexes do not generally occur under the experimental conditions (Figure S8)

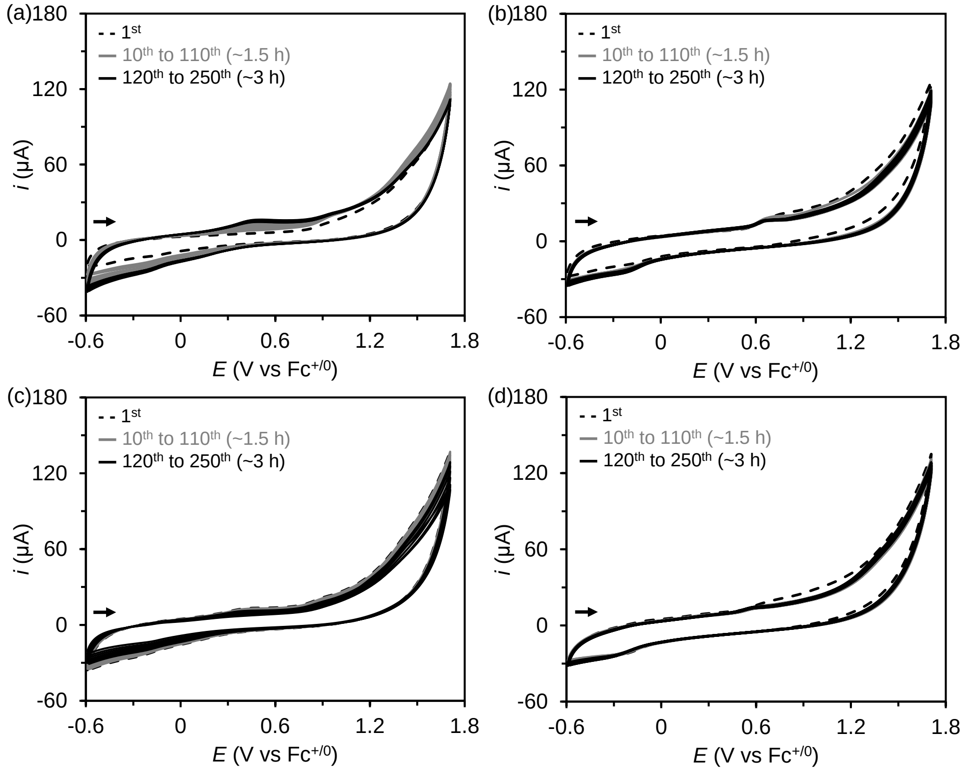

Figure S8. A series of 250 consecutive CVs were performed using a concentration of 0.4 mM of complexes. (a) 0.4 mM of **2** with 5 M H_2O (9% v/v); (b) 0.4 mM of **3** with 5 M H_2O (9% v/v); (c) 0.4 mM of **2** with 5 M H_2O (9% v/v) and 10 mM NaOH; (d) 0.4 mM of **3** with 5 M H_2O (9% v/v) and 10 mM NaOH. Scan rate: 100 mV s^{-1}. Supporting electrolyte: 0.1 M $[NBu_4][PF_6]$. CVs were plotted using the IUPAC convention.

5c. Characterization of electrodes after consecutive CV scans

The scanning electron microscopy (SEM) images and energy-dispersive X-ray spectroscopy (EDX) spectra (Figure S9) revealed the absence of any nanoparticles or precipitation on the glassy carbon electrode surface following 250 consecutive cyclic voltammetry (CV) scans.

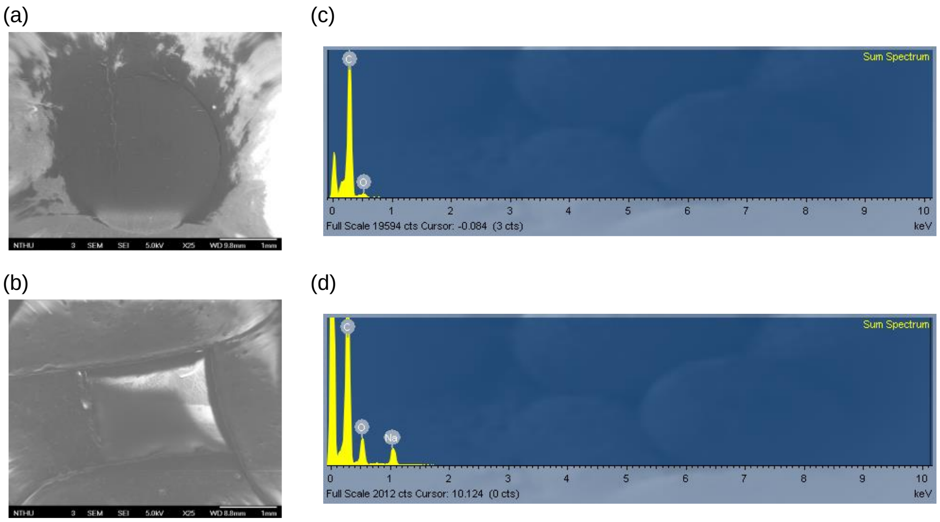

Figure S9. SEM images of a GC electrode (a) before and (b) after continuous 250 CV scans; EDX spectra of a GC electrode (c) before and (d) after continuous 250 CV scans. Conditions: 0.4 mM **3** in the presence of 10 mM NaOH with 5 M H_2O (9% v/v). Supporting electrolyte: 0.1 M $[NBu_4][PF_6]$ in MeCN.

Table S3. EDX analysis of a GC electrode before and after continuous 250 CV scans of **3** under alkaline conditions (5 M H_2O, 10 mM NaOH).

Before			After		
Element	Weight%	Atomic%	Element	Weight%	Atomic%
C K	93.63	95.14	C K	94.67	96.07
O K	6.37	4.86	O K	4.81	3.66
Total	100.00		Na K	0.50	0.26
			(Co L	≤ 0.02	≤ 0.01)
			Total	99.98	

5d. Stability test

For the stability assessment, three electrodes were immersed in an electrochemical cell containing 0.4 mM of a cobalt complex, 5 M H_2O (9%, v/v), and 0 or 10 mM NaOH in an 8 mL solution of MeCN (Figure S10). A glassy carbon (GC) rod electrode was the working electrode for controlled potential electrolysis (CPE) experiments. The auxiliary electrode consisted of a coiled platinum wire, while a non-aqueous electrode of 0.01 M $Ag/AgNO_3$ was used as the reference electrode. The supporting electrolyte employed in all electrochemical tests was 0.1 M $[NBu_4][PF_6]$. Before measurement, the sample was subjected to N_2 bubbling for 20 minutes, and the CPE experiments were conducted under an N_2 atmosphere with constant stirring. The catalyst solution was electrolyzed at a potential of 1.5 V vs. $Fc^{+/0}$ for 8 hours, matching the potential of the blank solution. The long-term durability of a molecular catalyst is a critical consideration for practical applications. Stable currents were observed during the operation of water oxidation (WO) at constant potentials, indicating the excellent stability of the catalysts under WO conditions (Figure S11).

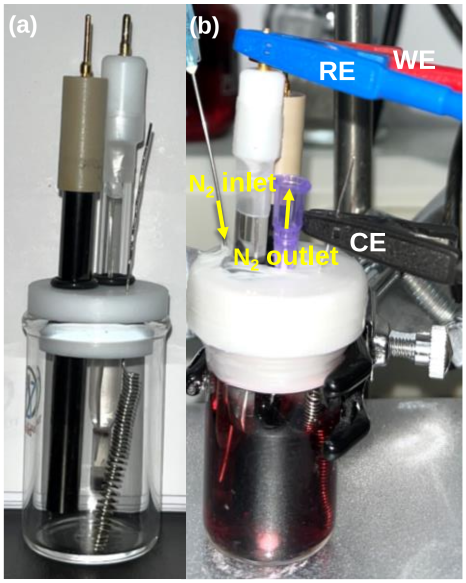

Figure S10. (a) Basic cell configuration for controlled potential electrolysis. (b) A photograph depicting the experimental setup employed for controlled potential electrolysis.

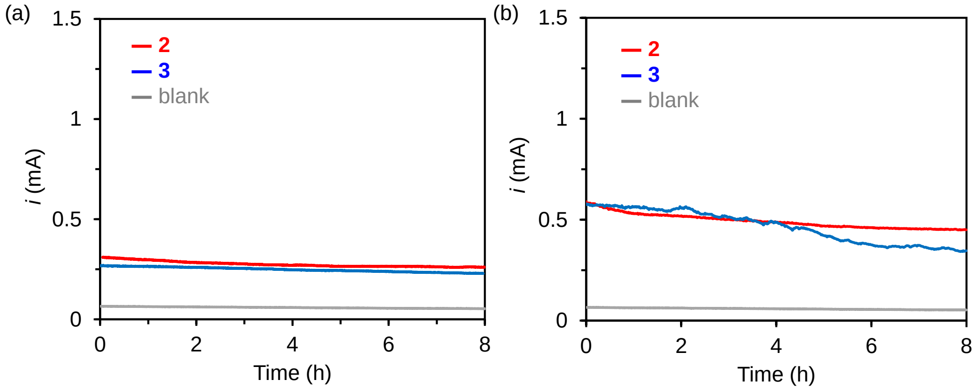

Figure S11. (a) Catalytic current curves were obtained in CPE experiments with complex **2** and **3**, at a concentration of 0.4 mM, in the presence of 5 M H_2O (9%, v/v) in MeCN at a potential of 1.5 V vs. $Fc^{+/0}$ over 8 hours. (b) Catalytic current curves were obtained in CPE experiments with complex **2** and **3**, both at a concentration of 0.4 mM, in the presence of 5 M H_2O (9%, v/v) and 10 mM NaOH in MeCN at a potential of 1.5 V vs $Fc^{+/0}$ for 8 hours. Supporting electrolyte: 0.1 M $[NBu_4][PF_6]$.

6. Controlled Potential Electrolysis (CPE) Experiments

6a. General considerations

The CPE experiments were conducted using a PalmSens4 Potentiostat connected to a computer running PSTrace software. The experiments were performed in MeCN with an applied potential of 1.5 V vs. $Fc^{+/0}$. The electrochemical cell contained 5 mL of MeCN, 0.4 mM Co complex, 5 M H_2O (9%, v/v), and either 0 or 10 mM NaOH. A glassy carbon rod electrode was the working electrode for the CPE experiments. A platinum wire was used as the auxiliary electrode, while a 0.01 M Ag/$AgNO_3$ non-aqueous electrode was the reference electrode. The supporting electrolyte used in all electrochemical experiments was 0.1 M $[NBu_4][PF_6]$. Prior to measurement, the sample was purged with N_2 for 20 minutes. The CPE experiments were conducted under an N_2 atmosphere with continuous stirring.

6b. Oxygen evolution

Oxygen evolution analysis was performed in the CPE experiments using a calibrated Microx 4 oxygen dipping probe (DP-PSt7). The probe was immersed in the solution containing the glassy carbon rod working electrode (Figure S12). The solution was vigorously stirred and bubbled with N2 for 20 minutes to remove any existing oxygen. The phase shift of the O_2 sensor on the Microx 4 probe was recorded at 3-second intervals and converted into the concentration of O_2 in the

solution using a two-point calibration curve with air and high-purity N_2. The 5 mL mixed solution and the optical fiber probe were allowed to equilibrate at room temperature for 3 minutes.

Under an applied potential of 1.50 V vs. $Fc^{+/0}$ for 1–3 hours. Background measurement from the substrate-only solution (i.e., 5 M H_2O (9%, v/v)) was obtained using the same method and subtracted. The measured dissolved oxygen concentration (μmol/L) was corrected by considering the maximum solubility of oxygen at different temperatures and then converted into a percentage for further calculations. The solubility of oxygen in acetonitrile saturated with air at 25 °C is reported to be 2.42 ± 0.14 × 10^{-3} M.[14] To determine the absolute amount of oxygen produced, the dissolved oxygen concentration was calibrated based on this literature value.

Based on the calculated total consumed charge (Equation S2) during the CPE experiment assuming a $4e^-$ catalytic process and considering the O_2 evolution, the faradaic efficiencies (Equation S3) for electrochemical O_2 were determined as follows:
For a $4e^-/4H^+$ catalytic water oxidation reaction, the theoretical number of moles of oxygen ($n_{O_2,\ theo.}$) can be determined using Faraday's law (Equation S2):

$$Q = nFN_A \qquad \text{Equation S2}$$

Here, n represents the number of electrons per mole of analyte (n = 4 for oxygen evolution), F is Faraday's constant (96,485 C mol^{-1}), N_A is the moles of analyte (i.e., $n_{O_2,\ theo.}$), and Q is the total charge in coulombs. The faradaic efficiency (FE%) can be calculated as the ratio of the experimentally observed moles of oxygen ($n_{O_2,\ exp.}$) to the theoretically expected moles of oxygen ($n_{O_2,\ theo.}$) multiplied by 100%:

$$\text{Faradaic efficiency (FE\%)} = \frac{n_{O_2,\ exp.}}{n_{O_2,\ theo.}} \times 100\% = \frac{n_{O_2,\ exp.}}{Q/nF} \times 100\% \qquad \text{Equation S3}$$

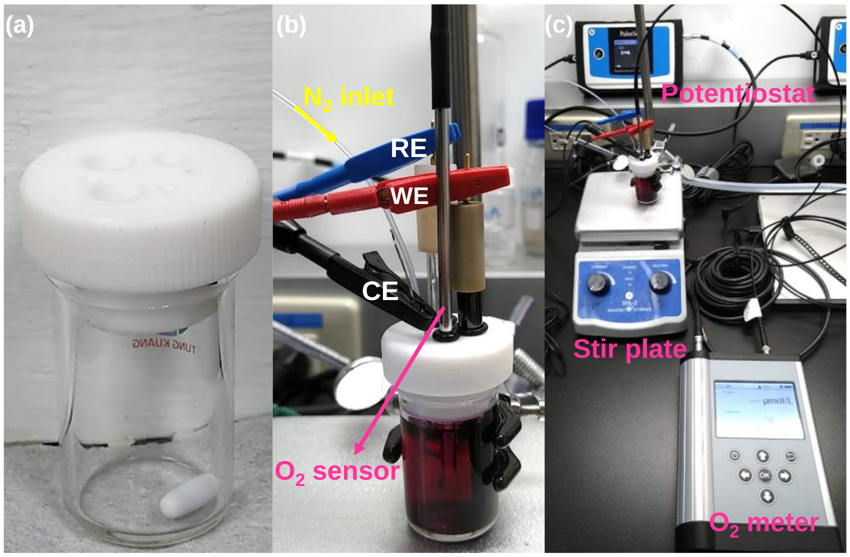

Figure S12. (a) Custom-designed cell utilized for oxygen evolution experiment. (b) and (c) Setup images demonstrating experimental configuration for oxygen evolution analysis.

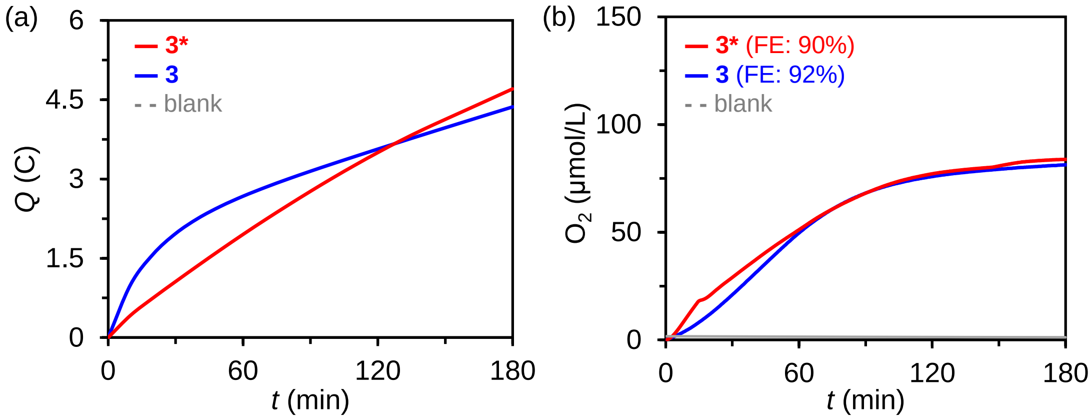

Figure S13. (a) Cumulative charge during CPE of a 0.4 mM **3** with 5 M H_2O (9%, v/v) and 0/10 mM NaOH in 0.1 M $[NBu_4][PF_6]$/MeCN at 1.5 V vs $Fc^{+/0}$ for 3 h. (b) The generation of O_2 during the electrolysis process was monitored using an oxygen dipping probe, providing real-time measurements. A dashed grey trace represents the control experiment conducted without the complex. The asterisk (*) indicates the presence of 10 mM NaOH.

6c. Selectivity of water oxidation by cobalt complexes

A colorimetric assay utilizing aqueous $Ti^{IV}(O)SO_4$ was employed to quantify hydrogen peroxide (H_2O_2), following a modified procedure from the literature.[15, 16] A calibration curve was generated by preparing a series of 10 mL DMF solutions with varying concentrations of urea·H_2O_2

(0–18 mM). Subsequently, 0.2 mL of $Ti^{IV}(O)SO_4$ solution was added to each prepared solution. The absorbance at 407 nm was measured using UV-visible spectroscopy with a 1 mm path-length quartz cell, enabling the construction of the calibration curve (Figure S14a).

The catalyst solution was electrolyzed at 1.50 V vs. $Fc^{+/0}$ for 1 h. For quantifying H_2O_2 in the catalytic reaction mixture, a 0.5 mL aliquot of the reaction mixture was mixed with 2 mL deionized water and 5 mL dichloromethane. The resulting aqueous layer (2 mL) was collected and combined with a 40 μL $Ti^{IV}(O)SO_4$ solution. The absorbance at 407 nm was measured using UV-visible spectroscopy. The absorbance at 407 nm was analyzed with (red trace) and without (black trace) the presence of $Ti^{IV}(O)SO_4$ (Figure S15). The concentrations of H_2O_2 in different catalytic reaction mixtures were calculated using the slope of the calibration curve (Figure S14b, y = 0.0954x + 0.0011) according to the following equation:

(1) ΔAbs was determined as the difference in absorbance at 407 nm between the $Ti(O)SO_4$ solution (Figure S15, red trace) and the background (Figure S15, black trace).

$$\Delta Abs = Abs@407\ nm_{Ti(O)SO4} - Abs@407\ nm_{background}$$

(2) The experimental concentration of H_2O_2 was determined by applying the following equation:

$$[H_2O_2]_{exp.}\ (mM) = (\Delta Abs–0.0011)/0.0954$$

(3) The theoretical concentration of H_2O_2 for a $2e^-/2H^+$ catalytic water oxidation reaction (H_2O is 5 M for all catalytic reactions) can be calculated using Faraday's law (Equation S2),

$$[H_2O_2]_{theo.} = (Q/(nF))/V$$

where n is the number of electrons per mole of analyte (n = 2 for H_2O_2 production), F is Faraday's constant (96,485 C mol^{-1}), Q is the total charge (C), and V is the volume of the solution.

(4) The diluted concentration of H_2O_2 can be expressed as the following formula:

$$[H_2O_2]_{dilu.} = [H_2O_2]_{theo.} \times (0.5\ mL\ (aliquot)/2\ mL\ (aqueous\ layer))$$

(1) The selectivity of H_2O_2 (%) was determined by comparing the experimental concentration with the diluted concentration:

$[H_2O_2]_{exp.}/[H_2O_2]_{dilu.}$

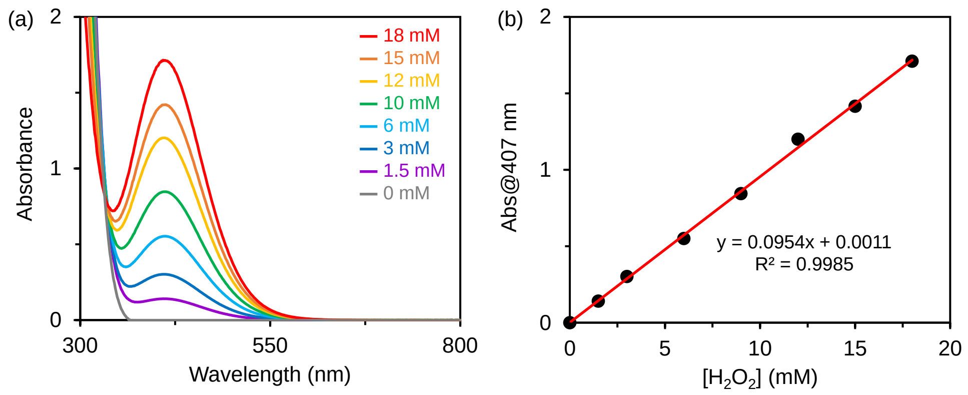

Figure S14. (a) UV-visible spectra were recorded to detect varying concentrations of H_2O_2 (0−18 mM) using $Ti^{IV}(O)SO_4$. (b) A calibration curve was established by plotting the absorbance values against the concentrations of H_2O_2 using the data obtained from Figure S14a.

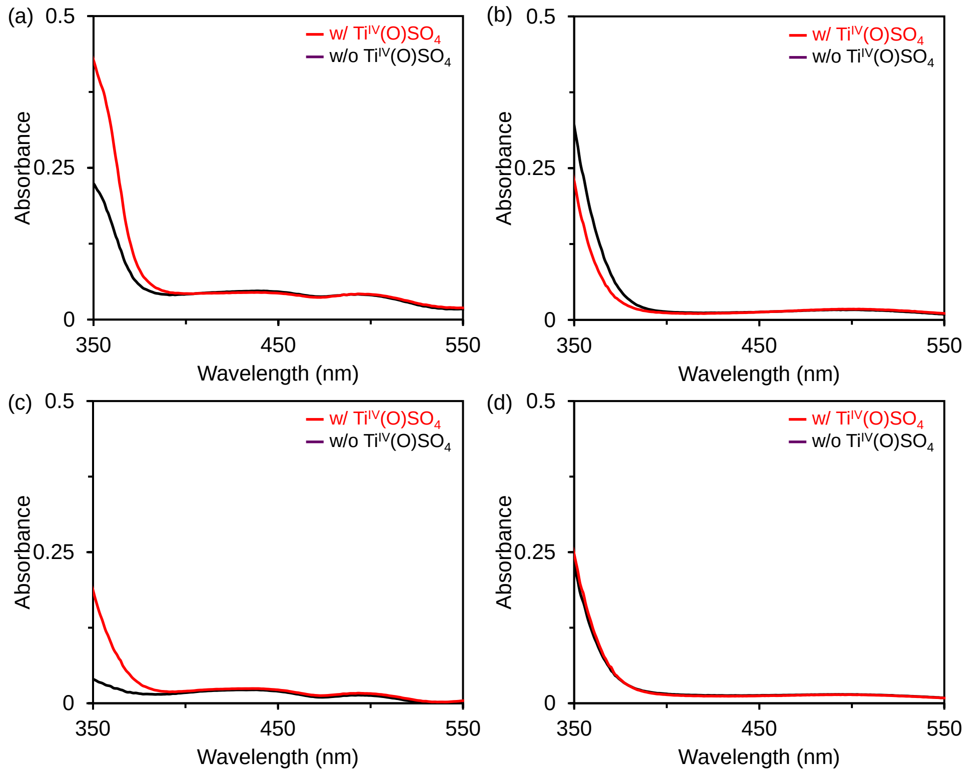

Figure S15. UV-visible spectra were obtained for H_2O_2 detection during the catalytic oxidation of H_2O using Co complexes. $Ti^{IV}(O)SO_4$ treatment resulted in a red trace, while the black trace represented the background spectra without $Ti^{IV}(O)SO_4$. Experimental conditions included: 0.4

mM complex **2** (a) & **3** (c) with 5 M H_2O (9%, v/v); 0.4 mM complex **2** (b) & **3** (d) with 5 M H_2O (9%, v/v) and 10 mM NaOH.

Table S4. A summary of selectivity of H_2O oxidation catalyzed by Co complexes under different conditions.

Co complex	**2**		**3**	
[NaOH] (mM)	0	10	0	10
Selectivity, O_2:H_2O_2 (%)	95:5	99:1	99:1	99:1

7. Rate Law Analysis of H_2O Oxidation Catalyzed by Co Complexes

7a. General considerations

Cyclic voltammetry (CV) experiments were conducted using a PalmSens4 Potentiostat interfaced with PSTrace software to ensure precise control and data acquisition. The CV measurements employed a comprehensive three-electrode configuration comprising a glassy carbon (GC) working electrode with a diameter of 3.0 mm, a counter electrode composed of platinum (Pt) wire, and a non-aqueous reference electrode of 0.01 M Ag/$AgNO_3$. To establish a conductive electrochemical environment, a supporting electrolyte solution of 0.1 M [NBu_4][PF_6] was used in all electrochemical experiments. For CV studies performed under ambient conditions, the electrochemical cell accommodated distinct concentrations of Co complexes and H_2O in a 5 mL solution of 0.1 M [NBu_4][PF_6] dissolved in acetonitrile (MeCN). Unless specified otherwise, the applied scan rate for all CV measurements was set at a uniform value of 100 mV s^{-1}.

7b. [Co]-dependence

Cyclic voltammograms (CVs) were performed on a 5 mL solution of acetonitrile (MeCN) containing various concentrations of Co complexes (0.1, 0.2, 0.4, 0.6, 0.8, and 1 mM), with H_2O (5 M; 9% v/v) in presence 0 or 10 mM NaOH and [NBu_4][PF_6] (0.1 M). The resulting catalytic current displayed a linear relationship with the concentration of Co complexes, as shown in Figure S16.

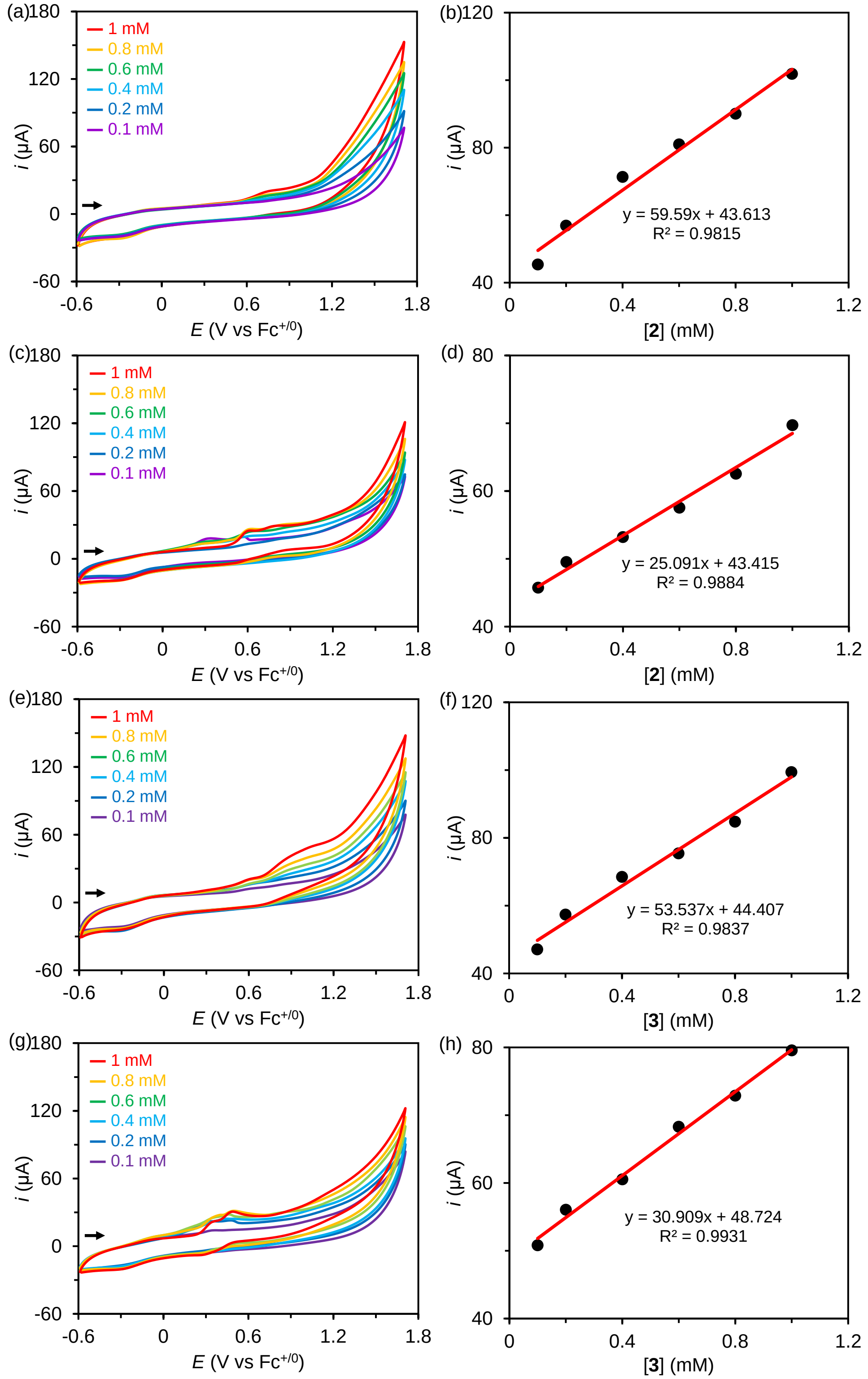

Figure S16. CVs of various concentrations of Co complexes (0.1–1 mM) in the presence of H_2O (5 M; 9%, v/v) and $[NBu_4][PF_6]$ (0.1 M) in 5 mL MeCN at the scan rate of 100 mV s^{-1}. (a) **2**; (c) **2** with 10 mM NaOH; (e) **3**; (g) **3** with 10 mM NaOH. Plots of anodic catalytic current derived

from the CV data (i, at 1.50 V vs. $Fc^{+/0}$) vs. [Co] show a first-order dependence. (b) **2**; (d) **2** with 10 mM NaOH; (f) **3**; (h) **3** with 10 mM NaOH. CVs were plotted using the IUPAC convention.

7c. $[H_2O]$-dependence

Cyclic voltammograms (CVs) were performed on a 5 mL MeCN solution containing 0.4 mM Co complexes, with varying concentrations of H_2O (1, 1.5, 3, 5, 7, 10, 15, and 20 M), and $[NBu_4][PF_6]$ (0.1 M). The catalytic current obtained from the CVs showed a linear relationship with the H_2O concentration within the range of 1–5 M H_2O, as shown in Figure S17.

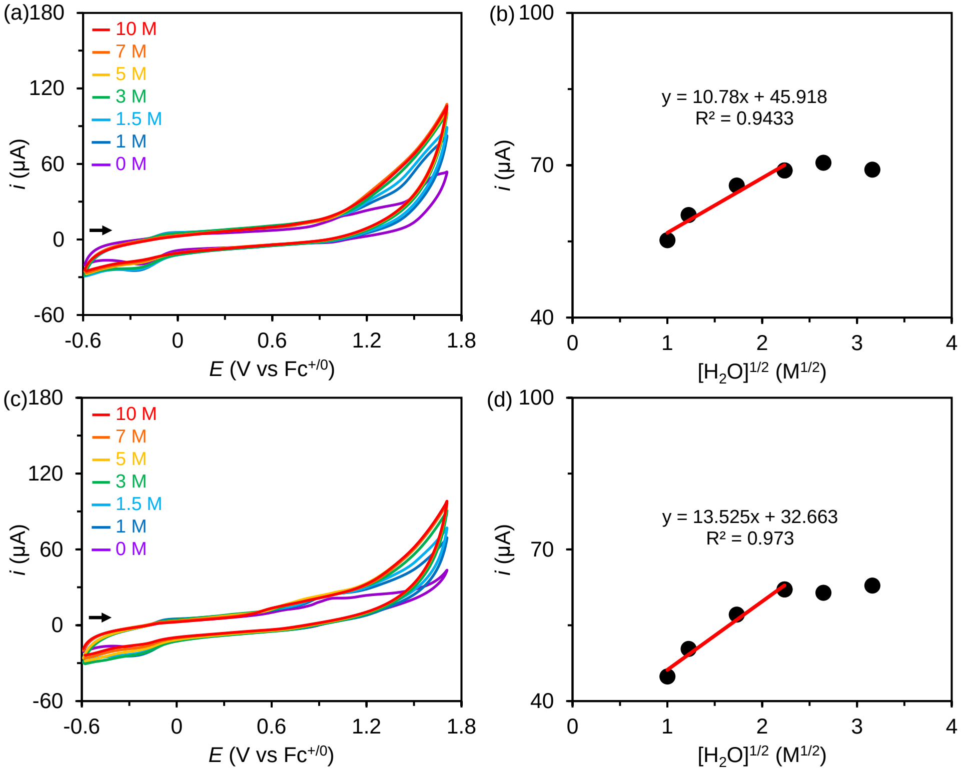

Figure S17. CVs of Co complexes (0.4 mM) in the presence of H_2O (1–10 M) and $[NBu_4][PF_6]$ (0.1 M) in 5 mL MeCN at the scan rate of 100 mV s^{-1}. (a) **2**; (c) **3**. Plots of anodic catalytic current derived from the CV data (i at 1.50 V vs. $Fc^{+/0}$) vs $[H_2O]^{1/2}$ show a first-order dependence. (b) **2**, (d) **3**. CVs were plotted using the IUPAC convention.

8. Kinetic Isotope Effects (KIE)

8a. General considerations

All cyclic voltammetry (CV) measurements were conducted using a PalmSens4 Potentiostat connected to a computer running PSTrace software. The experimental setup included a glassy carbon (GC) working electrode with a diameter of 3.0 mm, a platinum (Pt) wire counter electrode, and a non-aqueous reference electrode consisting of a 0.01 M Ag/$AgNO_3$ solution. The electrolyte used for all electrochemical experiments was 0.1 M [NBu_4][PF_6]. For CV studies performed under ambient conditions, the three electrodes were immersed in an electrochemical cell containing 0.4 mM Co complexes, 0 or 10 mM NaOH (or NaOD), 10 M H_2O (or D_2O, 9%, v/v), and 0.1 M [NBu_4][PF_6] in 5 mL of acetonitrile (or deuterated acetonitrile, CD_3CN). The scan rate for all CV measurements was set to 100 mV/s unless otherwise specified.

8b. Cyclic voltammograms analysis of Co complexes in H_2O or D_2O

The kinetic isotope effect (KIE) of the Co complexes was evaluated by calculating the ratio of rate constants, k_{cat}, H_2O/k_{cat}, and D_2O (Equation S4). Based on Equation S4, the resulting KIE values for **3** were determined at 1.50 V vs. $Fc^{+/0}$, respectively. These findings provide evidence that the rate-determining O–O formation step does not involve the cleavage of O–H bonds. This conclusion is further supported by the observations depicted in Figure S18.

$$\text{KIE} = \frac{k_{cat(H)}}{k_{cat(D)}} = \left(\frac{i_{c(H)}}{i_{c(D)}}\right)^2 \quad \text{Equation S4}$$

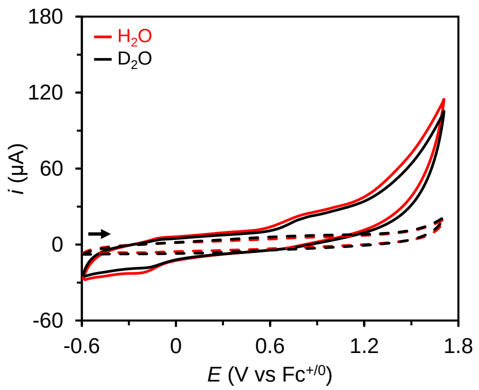

Figure S18. CVs of 0.4 mM Co complexes **3** in the presence of 5 M H_2O (9%, v/v) in MeCN (red solid trace) and 5 M D_2O in CD_3CN (black solid trace) at the scan rate of 100 mV s^{-1}. Dashed trace: blank with no complex in the presence of 5 M H_2O (red dashed trace) and D_2O (black dashed trace). CVs were plotted using the IUPAC convention.

9. Determining the TOF through Cyclic Voltammetry Measurements

9a. General procedure

$$i_c = n_c F A C^0_{cat}\sqrt{D_{cat} k_{obs}} \qquad \text{Equation S5}$$

$$k_{obs} = k_{cat} C^0_{substrate} \qquad \text{Equation S6}$$

Where i_c is the catalytic current, n_c is the number of catalytically transferred electrons during the catalytic process (n_c = 4 for O_2 production), F is the Faraday constant (96485 C mol^{-1}), A is the surface area of the working electrode (2.25×10^{-2} π cm^2 for the glassy carbon electrode used in this study), C^0_{cat} is the concentration of catalyst [M], D_{cat} is the diffusion coefficient of the catalysts (see Section 4e in the Supporting Information for details), and k_{obs} is the observed rate constant, which is identical to the TOF (s^{-1}) in this case, k_{cat} is the catalytic rate constant (M^{-1} s^{-1}), $C^0_{substrate}$ is the the substrate concentration [M].

Considering the substrate concentration does not change significantly throughout the measurement, we can express the TOF of the catalytic process as a pseudo-first-order rate constant, k_{obs} (k_{obs} = $k_{cat}C_{H_2O}$ = TOF). In the case of catalysts **2** and **3**, it was observed that the catalytic current remained unaffected by the scan rate when the scan rate exceeded 800 mVs^{-1} (as shown in Figure S19 and S20). Hence, the k_{obs} values were determined from the cyclic voltammograms obtained at a scan rate of 800 mV s^{-1} and are presented in Table S5.

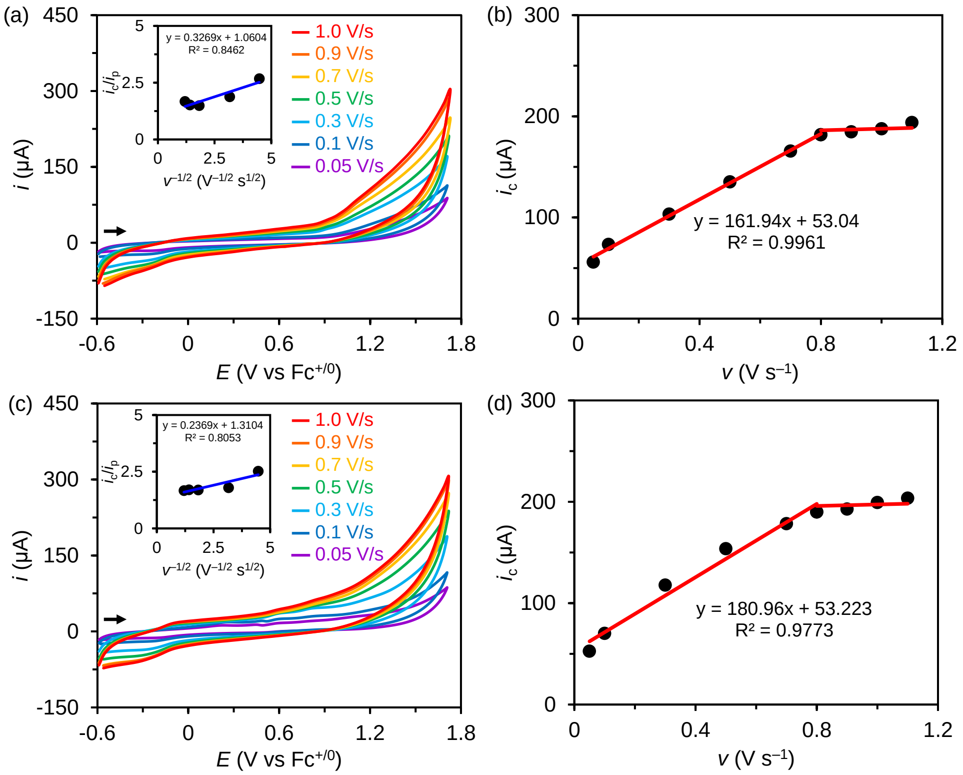

Figure S19. CVs of 0.4 mM Co complex **2** in the presence of various concentrations of H_2O (5 and 10 M) and NaOH (0 and 10 mM) in MeCN at various scan rates. Inset: plots of i_c/i_p at 1.50 V vs $v^{-1/2}$. (a) 5 M H_2O (9%, v/v); (c) 5 M H_2O (9%, v/v) and 10 mM NaOH. Plots of anodic catalytic current derived from the CVs data (i_c, at 1.50 V vs. $Fc^{+/0}$) vs scan rate (v, V s^{-1}): (b) 5 M H_2O (9%, v/v); (d) 5 M H_2O (9%, v/v) and 10 mM NaOH. CVs were plotted using the IUPAC convention.

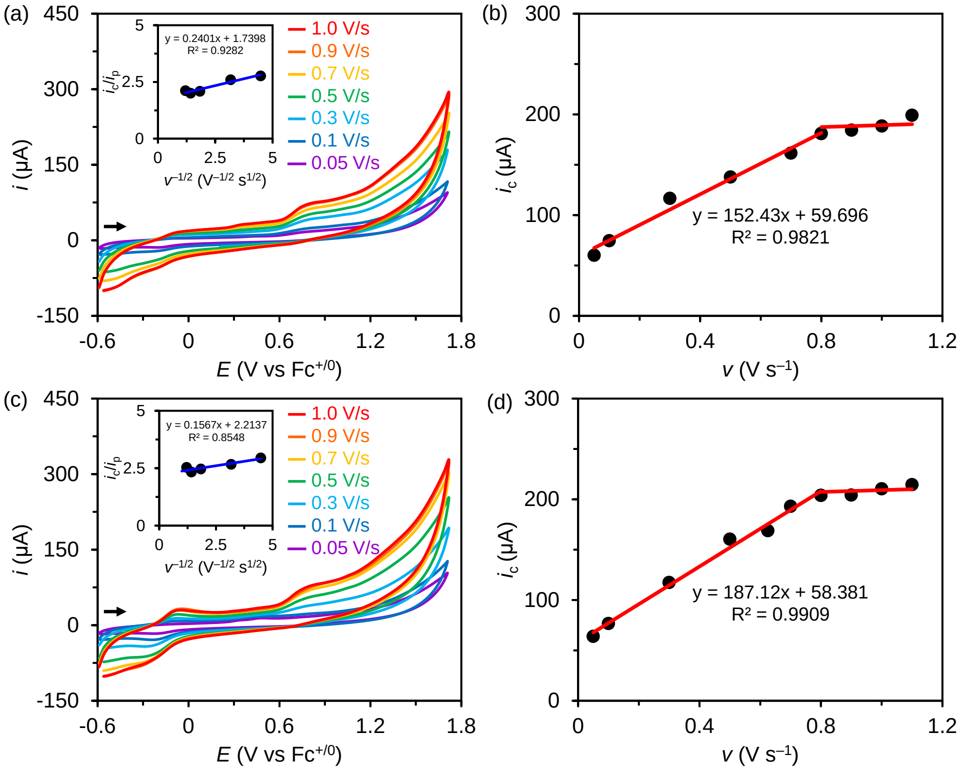

Figure S20. CVs of 0.4 mM Co complex **3** in the presence of various concentrations of H_2O (5 and 10 M) and NaOH (0 and 10 mM) in MeCN at various scan rates. Inset: plots of i_c/i_p at 1.50 V vs $v^{-1/2}$. (a) 5 M H_2O (9%, v/v); (c) 5 M H_2O (9%, v/v) and 10 mM NaOH. Supporting electrolyte: 0.1 M [NBu$_4$][PF$_6$]. Plots of anodic catalytic current derived from the CVs data (i_c, at 1.50 V vs. $Fc^{+/0}$) vs scan rate (v, V s^{-1}): (b) 5 M H_2O (9%, v/v); (d) 5 M H_2O (9%, v/v) and 10 mM NaOH. CVs were plotted using the IUPAC convention.

Table S5. A summary of turnover frequencies (TOFs) of Co complexes **1**–**3** under catalytic conditions.

Complex	[H_2O] (M)	[NaOH] (mM)	TOF (s^{-1})
**1**	5	0	2.0×10^1
	5	10	3.4×10^1
**2**	5	0	1.5×10^1
	5	10	1.7×10^1
**3**	5	0	1.7×10^1
	5	10	2.1×10^1

10. Estimation of the Thermodynamic Reduction Potential of O_2/H_2O at Non-standard State

10a. Open-circuit potential (OCP) measurement for E_{H^+/H_2} in MeCN

The H^+/H_2 potential in organic media can be determined using a recently reported protocol through open-circuit potential measurements at a Pt electrode (Figure S21).[17] The H^+/H_2 potentials were measured for MeCN containing H_2O (5 and 10 M) and NaOH (0 and 10 mM) under 1 atm H_2 (local atmospheric pressure of 756 mm Hg, correction to 1 atm < 1 mV). All MeCN solutions contained 0.1 M $[NBu_4][PF_6]$ supporting electrolyte. A stable open-circuit potential was observed, and this potential was corrected to be versus $Fc^{*+/0}$ by adding decamethylferrocene (Fc*) and performing CV at a second, glassy carbon working electrode. The H^+/H_2 potential is referenced to the decamethylferrocenium/decamethylferrocene redox couple ($Fc^{*+/0}$) due to the irreversible redox behavior of ferrocenium/ferrocene ($Fc^{+/0}$) in the presence of NaOH (Figure S22). The half-wave potential for the $Fc^{+/0}$ redox couple is 0.50 V relative to $Fc^{*+/0}$ in a solution of MeCN. The observed H^+/H_2 potentials under catalytic conditions in MeCN are listed in Table S6.

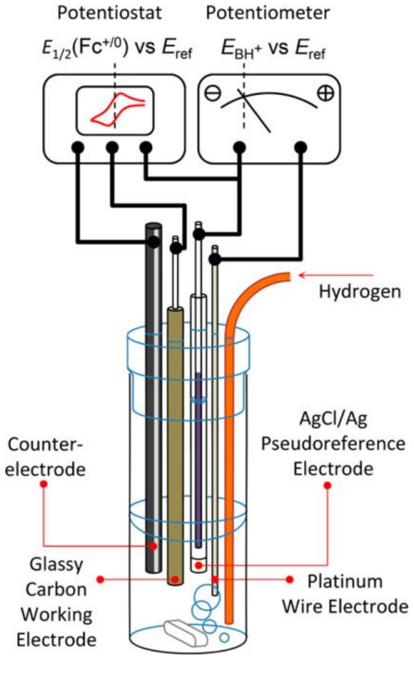

Figure S21. Schematic of the four-electrode cell configuration used for open circuit potential (OCP) measurements. The reduction potential of H^+/H_2 was measured for a MeCN solution containing $[NBu_4][PF_6]$ (0.1 M), H_2O (5 and 10 M), and NaOH (0 and 10 mM) under 1 atm H_2. (Figure S21 is reproduced with permission from ref. 17; copyright 2013 American Chemical Society.)

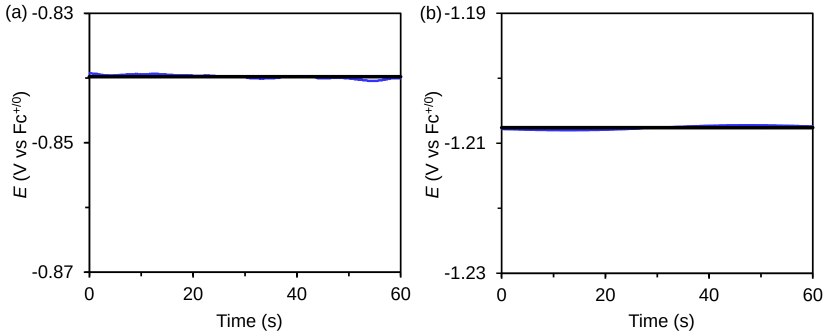

Figure S22. The black traces show the average OCP of E_{H^+/H_2} under catalytic conditions in MeCN solutions under 1atm H_2. (a) 5 M H_2O (9%, v/v); (b) 5 M H_2O (9%, v/v) and 10 mM NaOH. Supporting electrolyte: 0.1 M $[NBu_4][PF_6]$.

10b. Estimation of $E_{O2/H2O}$ based on OCP measurements of $E_{H+/H2}$

i)	$O_{2(g)} + 2\ H_{2(g)} \rightleftharpoons 2\ H_2O_{(aq)}$	$E^{\circ}_{O2/H2O(aq)} = 1.23$ V vs $E_{H+//H2(aq)}$
ii)	$4\ H^+_{(MeCN)} + 4\ e^-_{(V\ vs\ Fc+/0)} \rightleftharpoons 2\ H_{2(g)}$	$E_{H+/H2(MeCN)} = -0.84$ V vs $Fc^{+/0}$
iii)	$2\ H_2O_{(aq)} \rightleftharpoons 2\ H_2O_{(MeCN)}$	$\Delta G_{H2O(aq\rightarrow MeCN)} = < 1$ kcal mol^{-1}
**iv)**	$\mathbf{O_{2(g)} + 4\ e^-_{(V\ vs\ Fc+/0)} + 4\ H^+_{(MeCN)} \rightleftharpoons 2\ H_2O_{(MeCN)}}$	$\mathbf{E_{O2/H2O(MeCN)} = 0.39}$ **V vs** $\mathbf{Fc^{+/0}}$

The thermodynamic reduction potentials of $E_{O2/H2O}$ under catalytic conditions are listed in Table S6.

Table S6. A summary of $E_{H+/H2}$ and $E_{O2/H2O}$ under catalytic conditions.

$[H_2O]$ (M)	[NaOH] (mM)	E_{H^+/H_2} (V vs $Fc^{+/0}$)	E_{H_2O/O_2} (V vs $Fc^{+/0}$)
5	0	−0.84	0.39
5	10	−1.21	0.02

10c. Derivation of overpotential (η)

The overpotentials (η) are calculated by Equation S7 and listed in Table S7.

$$\eta = |E_{1/2} - E_{H_2O/O_2}| \qquad \text{Equation S7}$$

Table S7. A summary of overpotentials of H_2O oxidation by Co complexes.

Complex	$[H_2O]$ (M)	[NaOH] (mM)	$E_{H2O/O2}$ (V vs. $Fc^{+/0}$)	E_{cat} (V vs. $Fc^{+/0}$)	η (V)
**1**	5	0	0.39	1.09	0.70
	5	10	0.02	0.82	0.80
**2**	5	0	0.39	1.01	0.62
	5	10	0.02	0.73	0.71
**3**	5	0	0.39	0.89	0.50
	5	10	0.02	0.37	0.35

11. Spectroelectrochemical Study

General considerations

Spectroelectrolysis was performed using a BAS Inc. spectroelectrochemical quartz cell (l = 1 mm) containing a platinum (Pt) gauze working electrode, a Pt wire counter electrode, and a 0.01 M $Ag/AgNO_3$ non-aqueous reference electrode in conjunction with the PalmSens4 Potentiostat (Figure S23). UV-Vis absorption spectral changes of a 0.04 mM solution of Co complex in MeCN with 1 mM $[NBu_4][OH]$ containing 0.1 M $[NBu_4][PF_6]$ at 298 K were recorded automatically each 30 s in Cary 60 UV-Vis spectrophotometer. The spectroelectrochemical (SEC) technique allows for an in-situ spectroscopic study of the chemical stability of the catalyst, reaction intermediates, and catalyst resting state.

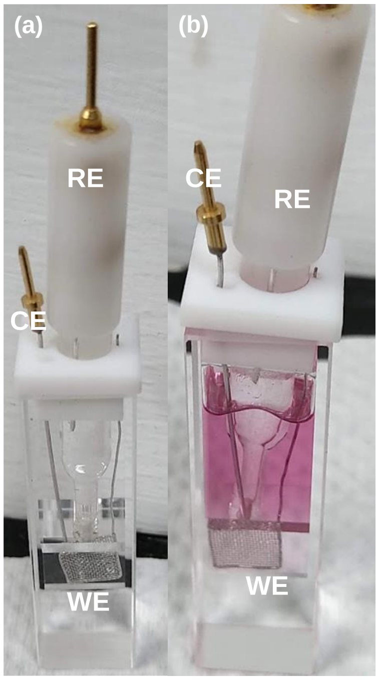

Figure S23. (a) Quartz glass spectroelectrochemical cell with a 1 mm thin layer. (b) Experimental setup of a spectroelectrochemical experiment.

12. NMR Spectroscopic Experiments

12a. General considerations for solvent suppression

Solvent suppression is a nuclear magnetic resonance spectroscopy (NMR) technique to decrease undesired signals from a sample's solvent. Furthermore, WET is an effective technique to suppress multiple solvent peaks. The experimental parameters of solvent suppression by WET are d1 = 3 sec (delay time), pw90 = 10 usec (time of pulse 90 degrees), ns = 32 (number of scans), and temp. = 300 K, suppression positions are 3.107 ppm, 2.913 ppm, 1.608 ppm, 1.363 ppm and 0.973 ppm.

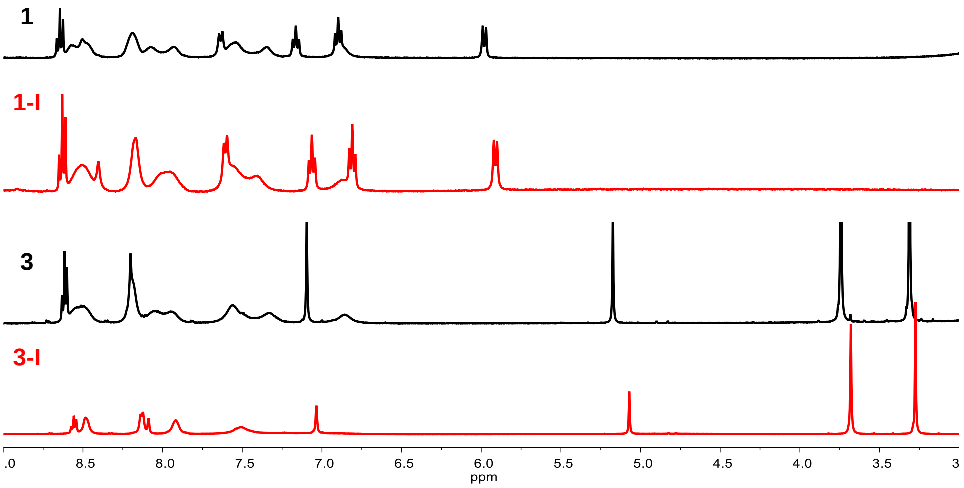

Figure S24. ^{1}H NMR spectra compare the deprotonation state before (black) and after (red) complexes **1** and **3**.

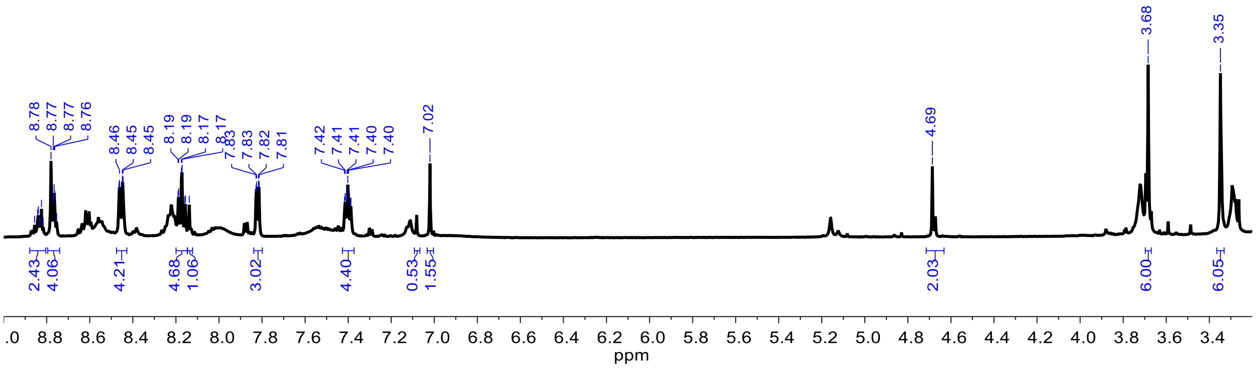

Figure S25. The ^{1}H NMR spectrum of WOR catalyzed by **3** under alkaline conditions in CD_3CN (the spectrum at 40 min of Figure 7a in the main manuscript).

13. EPR Spectroscopic Experiments and Simulations

13a. General considerations

X-band (9.63 GHz) CW EPR spectra were recorded using a Bruker (Billerica, MA) EMX plus spectrometer equipped with an ER 4116DM resonator at microwave power 10.02 mW, with 100 kHz magnetic field modulation of 4.00 G amplitude. Digital field resolution was 8192 points per spectrum. Cryogenic temperatures (10 K) were achieved and controlled using an Oxford Instruments ESR900 liquid helium cryostat in conjunction with an Oxford Instruments ITC503 temperature and gas flow controller. Pulse X-band EPR studies were performed using an ER 4116DM resonator (Bruker). All CW EPR data were acquired under nonsaturating, slow-passage conditions. The EPR experimental spectra were simulated and visualized using the Matlab software with the EasySpin toolbox.[18]

13b. EPR spectrum of **3-II**

To investigate whether the one-electron oxidized cobalt species of **3-I** was in-situ characterized with EPR by adding one equiv of $[NBu_4][IO_4]$ into the solution of **3** under alkaline conditions. The sample was vigorously shaken for 3 min at room temperature. A 300 μL aliquot of the sample was transferred into an EPR quartz tube and frozen quickly by immersion in liquid nitrogen. Then, the samples were inserted into the EPR cavity, which was precooled to the desired temperature. The experimental EPR spectrum was recorded at 10 K.

14. Cold-spray ionization mass spectrometry (CSI-MS)

14a. General considerations

The catalytically relevant cobalt intermediates were detected by a CSI-MS system, combining a home-built cold-spray ionization source and a linear ion trap mass spectrometer (LTQ XL, Thermo Fisher Scientific) to detect the unstable intermediates (Figure S26). The entire sample transport line was constructed with a dual-layered structure to regulate the temperature of the sample. In this work, the gap between the layers was purged with cold nitrogen gas to maintain the sample line at −5 °C before ionization. The sampling line was rinsed three times (or more, depending on the quality of the spectrum) with deoxygenated solvent (MeCN) that was prepared by bubbling with nitrogen gas (1 hour). An aliquot of the Co intermediate sample was injected via the sampling line into the CSI source, 5.3 kV was applied at spray, and 40 V was applied at the tube lens. The temperature of the ion transfer tube was set to 350 °C to remove solvent from the charged droplet before mass analysis.

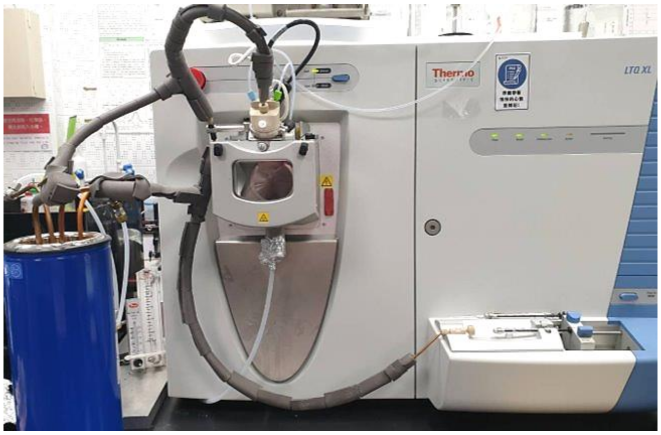

Figure S26. The home-built cold-spray ionization (CSI) source is installed on a linear ion trap (LIT) mass spectrometer.

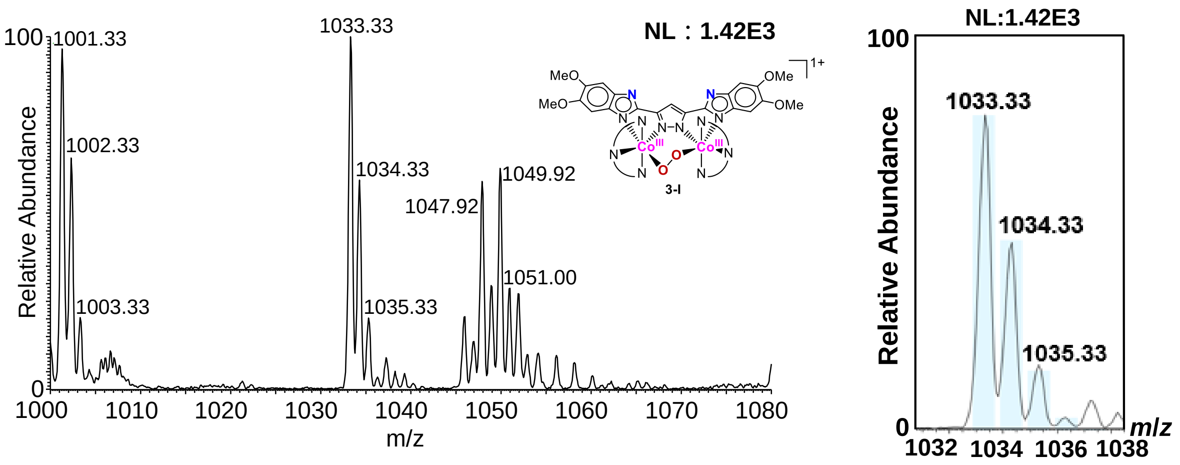

Figure S27. CSI-MS spectrum of **3-I** (*m*/*z* 1033.33) was observed in MeCN at –5 °C. Inset: zoom in on the spectrum and theoretically isotopic distribution (blue bar). NL (normalization level): base peak intensity.

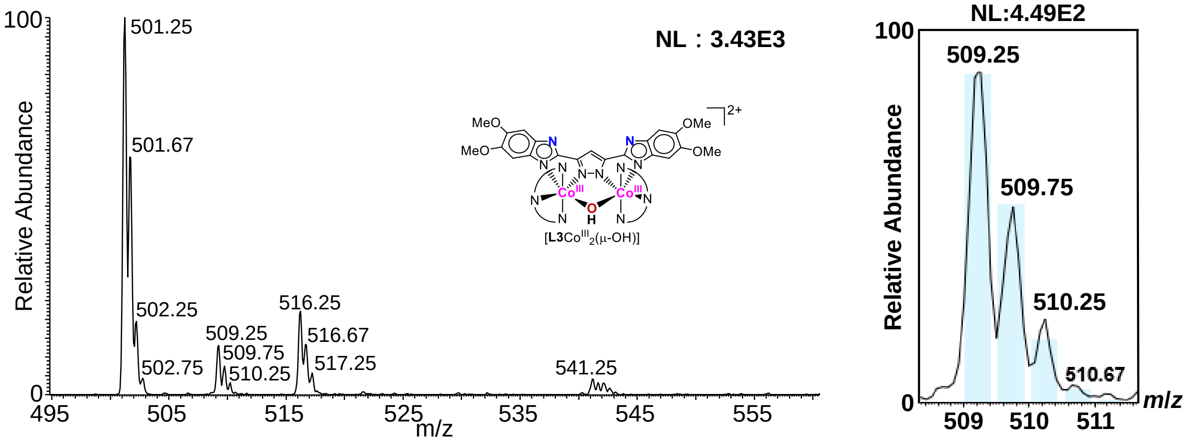

Figure S28. CSI-MS spectrum of [**L3**Co^{III}_2(μ-OH)]$^{2+}$ (*m*/*z* 509.25) observed in MeCN at –5 °C. Inset: zoom in on the spectrum and theoretically isotopic distribution (blue bar). NL (normalization level): base peak intensity.

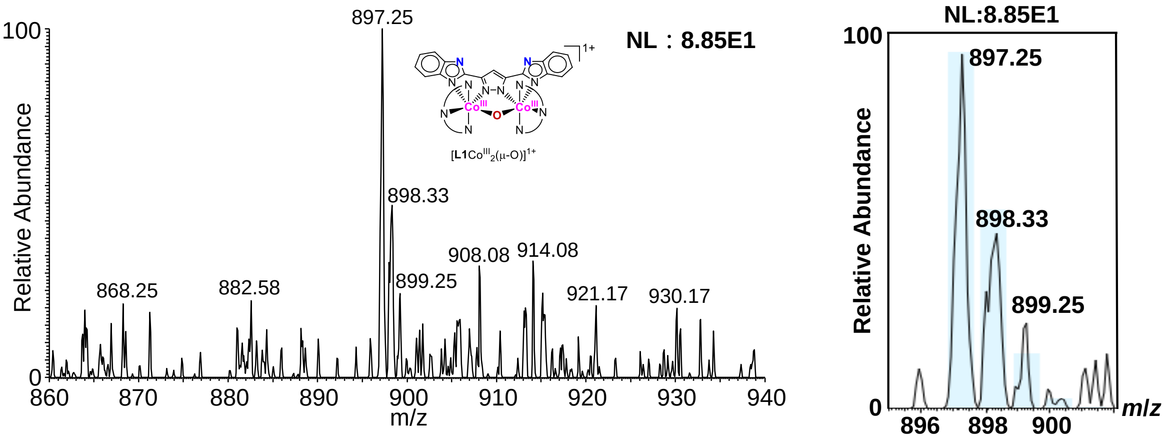

Figure S29. CSI-MS spectrum of [**L1**Co^{III}_2(μ-O)]$^{1+}$ (*m/z* 897.25) observed in MeCN at −5 °C. Inset: zoom in on the spectrum and theoretically isotopic distribution (blue bar). NL (normalization level): base peak intensity.

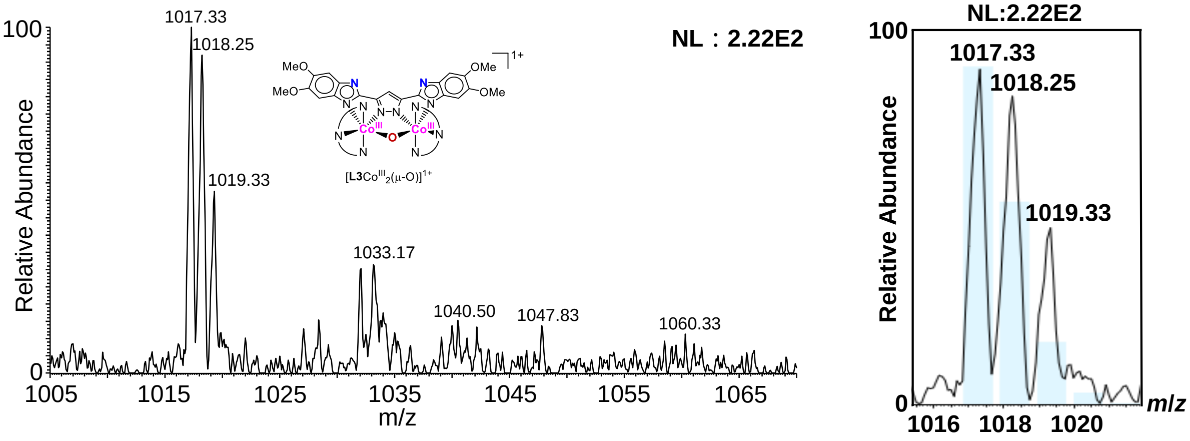

Figure S30. CSI-MS spectrum of [**L3**Co^{III}_2(μ-O)]$^{1+}$ (*m/z* 1017.33) observed in MeCN at −5 °C. Inset: zoom in on the spectrum and theoretically isotopic distribution (blue bar). NL (normalization level): base peak intensity.

15. Eyring Analysis

15a. General considerations

All cyclic voltammograms (CVs) were performed with a PalmSens4 Potentiostat interfaced to a computer with PSTrace software. The three-electrode setup for all cyclic voltammogram (CV) measurements included a glassy carbon (GC) working electrode (3.0 mm diameter), a platinum (Pt) wire counter electrode, and a 0.01 M Ag/$AgNO_3$ non-aqueous reference electrode. For the CV studies, three electrodes were immersed into the electrochemical cell containing 0.4 mM Co complex, 5 M H_2O, and 10 mM NaOH in 5 mL MeCN, and the 5 mL mixed solution was allowed to equilibrate for 5 min at desired temperatures (0, 25, 35, 45, and 55 °C, Figure S31). The supporting electrolyte for all electrochemical experiments was 0.1 M $[NBu_4][PF_6]$. All scan rates presented in this work are 100 mV s^{-1} unless otherwise noted for CV studies.

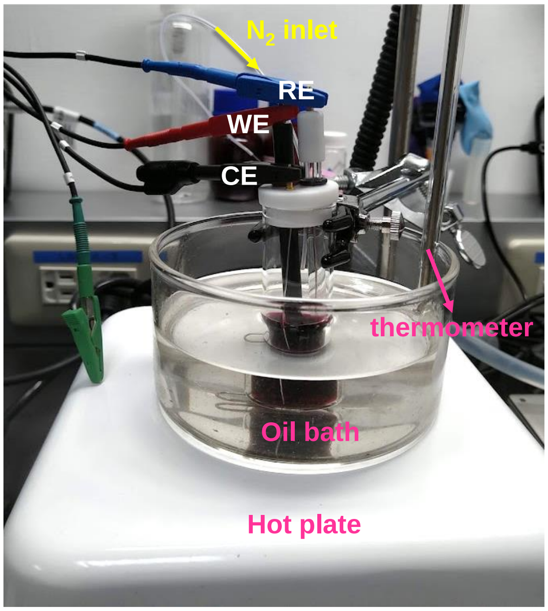

Figure S31. A picture of the setup used in Eyring analysis.

15b. Eyring plot of H_2O oxidation catalyzed by Co complexes

The rate constant of H_2O oxidation (k_{cat}), in units of $M^{-1}\cdot s^{-1}$, was obtained by Eq. (Equation S8). These data provide the basis for the Eyring plot in **Error! Reference source not found.**−Figure **S34**, and therefore $\Delta H^{\ddagger}$, $\Delta S^{\ddagger}$, and $\Delta G^{\ddagger}$ can be derived from the Eyring equation, Eq. (Equation S8), and summarized in Table S8.

$$\ln\frac{k}{T} = \frac{-\Delta H^{\ddagger}}{R}\frac{1}{T} + \ln\frac{k_B}{h} + \frac{\Delta S^{\ddagger}}{R} \qquad \Delta G^{\ddagger} = \Delta H^{\ddagger} - T\Delta S^{\ddagger} \qquad \text{Equation S8}$$

k is the rate constant, k_B is Boltzmann's constant (1.381×10^{-23} J K^{-1}), T is the temperature (K), h is Planck's constant (6.626×10^{-34} J s), R is the universal gas constant (8.3145 J K^{-1} mol^{-1}), $\Delta H^{\ddagger}$ is the enthalpy of activation (kcal mol^{-1}), $\Delta S^{\ddagger}$ is the entropy of activation (cal mol^{-1} K^{-1}), and $\Delta G^{\ddagger}$ is the Gibbs free energy of activation (kcal mol^{-1}).

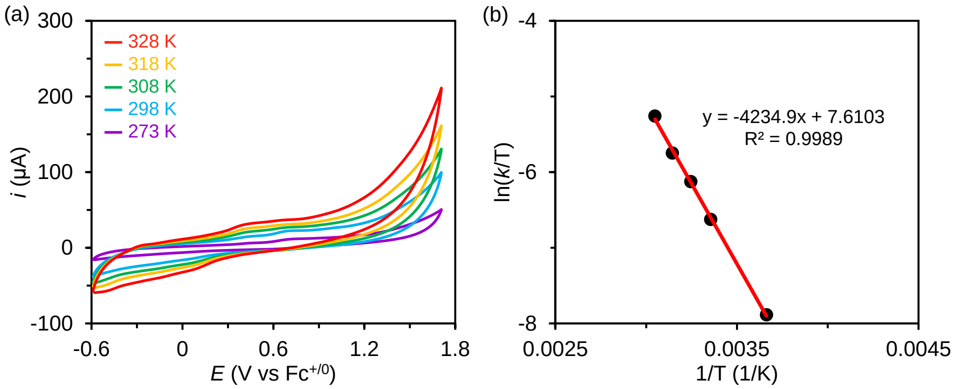

(c)

T (K)	1/T (1/K)	k (M^{-1} s^{-1})	ln(k/T)
273	3.7×10^{-3}	1.0×10^{-1}	−7.89
298	3.4×10^{-3}	4.0×10^{-1}	−6.63
308	3.2×10^{-3}	6.7×10^{-1}	−6.13
318	3.1×10^{-3}	1.0×10^{0}	−5.75
328	3.0×10^{-3}	1.7×10^{0}	−5.26

Figure S32. (a) CVs of 0.4 mM Co complex **3** with 5 M H_2O in MeCN at various temperatures (273, 298, 308, 318, and 328 K). Scan rate: 100 mV s^{-1}. Supporting electrolyte: 0.1 M [NBu_4][PF_6]. (b) The rates of H_2O oxidation were studied at various temperatures to construct the Eyring plot (i at 1.54 V vs. Fc$^{+/0}$). (c) Eyring plot of H_2O oxidation catalyzed by **3** between 273 K to 328 K. $\Delta H^{\ddagger}$ = 8.4 kcal mol^{-1}, $\Delta S^{\ddagger}$ = −32.0 cal mol$^{-1}\cdot$K^{-1}, and $\Delta G^{\ddagger}$ = 17.9 kcal mol^{-1}. CVs were plotted using the IUPAC convention.

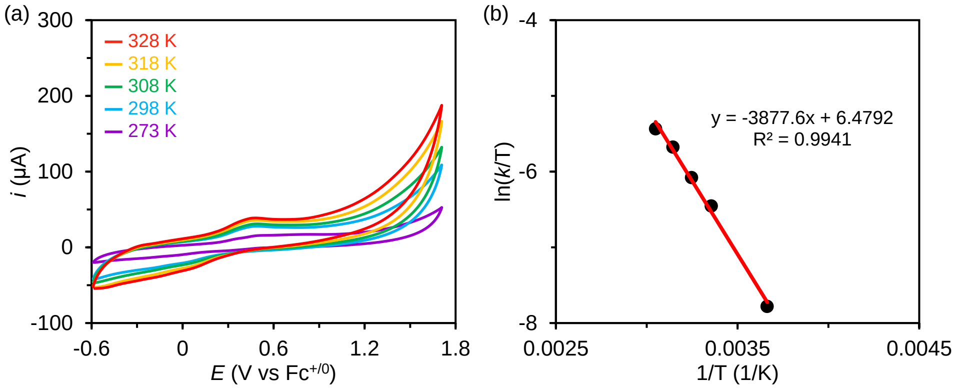

(c)

T (K)	1/T (1/K)	*k* (M^{-1} s^{-1})	ln(*k*/T)
273	3.7×10^{-3}	1.1×10^{-1}	−7.78
298	3.4×10^{-3}	4.7×10^{-1}	−6.45
308	3.2×10^{-3}	7.1×10^{-1}	−6.08
318	3.1×10^{-3}	1.1×10^{0}	−5.68
328	3.0×10^{-3}	1.4×10^{0}	−5.44

Figure S33. (a) CVs of 0.4 mM Co complex **3** in the presence of 10 mM NaOH and 5 M H_2O in MeCN at various temperatures (273, 298, 308, 318, and 328 K). Scan rate: 100 mV s^{-1}. Supporting electrolyte: 0.1 M $[NBu_4][PF_6]$. (b) The rates of H_2O oxidation were studied at various temperatures to construct the Eyring plot (i_c at 1.54 V vs. $Fc^{+/0}$). (c) Eyring plot of H_2O oxidation catalyzed by **3** between 273 K to 328 K. $\Delta H^‡$ = 7.7 kcal mol^{-1}, $\Delta S^‡$ = −34.2 cal $mol^{-1}\cdot K^{-1}$, and $\Delta G^‡$ = 17.9 kcal mol^{-1}. CVs were plotted using the IUPAC convention.

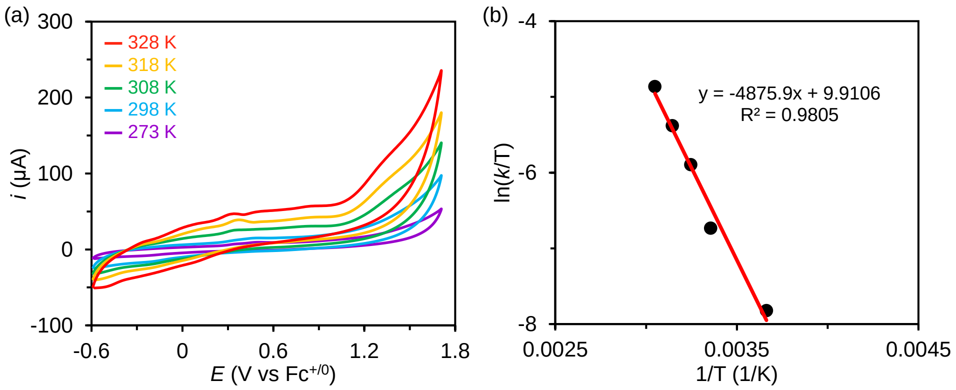

(c)

T (K)	1/T (1/K)	k (M^{-1} s^{-1})	ln(k/T)
273	3.7×10^{-3}	1.1×10^{-1}	−7.82
298	3.4×10^{-3}	3.5×10^{-1}	−6.73
308	3.2×10^{-3}	8.5×10^{-1}	−5.90
318	3.1×10^{-3}	1.5×10^{0}	−538
328	3.0×10^{-3}	2.5×10^{0}	−4.87

Figure S34. (a) CVs of 0.4 mM Co complex **3** in the presence of 10 mM NaOH, 5 M H_2O, and 1 mM crown ether in MeCN at various temperatures (273, 298, 308, 318, and 328 K). Scan rate: 100 mV s^{-1}. Supporting electrolyte: 0.1 M $[NBu_4][PF_6]$. (b) The rates of H_2O oxidation were studied at various temperatures to construct the Eyring plot (i_c at 1.54 V vs. $Fc^{+/0}$). (c) Eyring plot of H_2O oxidation catalyzed by **3** between 273 K to 328 K. $\Delta H^‡$ = 9.7 kcal mol^{-1}, $\Delta S^‡$ = −27.4 cal $mol^{-1}\cdot K^{-1}$, and $\Delta G^‡$ = 17.8 kcal mol^{-1}. CVs were plotted using the IUPAC convention.

Table S8. A summary of activation parameters for Co complexes under catalytic conditions.

Co complex	**1**		**3**		
[NaOH] (mM)	0	10	0	10	10*
$\Delta H^‡$ (kcal mol^{-1})	7.7	5.2	8.4	7.7	9.7
$\Delta S^‡$ (cal mol^{-1} K^{-1})	−33.9	−41.3	−32.0	−34.2	−27.4
$\Delta G^‡$ (kcal mol^{-1})	17.8	17.5	17.9	17.9	17.8

16. Computational Study

16a. Methods

Density functional theory (DFT) calculations were utilized to study the thermodynamics of the catalytic pathways. The D4-BJ[19, 20] correction was used for all calculations for the dispersion effect. The resolution of the identity approximation[21] was used for the Coulomb integrals with the corresponding auxiliary basis sets,[22], and the chain-of-sphere approximation[23] was used for the exchange integrals. Geometries were optimized using the TPSS[24] functional in the gas phase. The 6-31G*[25] basis set was used for the non-metal atoms. The LANL2DZ[26] basis set was used for the metal atoms. Frequency calculations were calculated in the gas phase on top of the optimized geometries using the same basis sets combined with the B3LYP[27] functional. Single point energies were calculated using the wB97X functional and the reparametrized D4 correction.[28] The def2-TZVPP[29] basis set was used for all of the atoms. The solvation effect was corrected using the continuum solvation model SMD[30] to account for the effect of the acetonitrile solvent. Final Gibbs free energies were calculated according to Equation S9. The solvation-free energy of the proton and the energy of the normal hydrogen electrode in acetonitrile are -260.2 and -104.3 kcal/mol, respectively, as reported by Kelly et al.[31] The effect of chemical concentration was corrected following the previous report.[32] Orca version 5.0.4 was used to perform all computations. Redox potentials were calculated against the Fc/Fc^+ couple.

$$G = E_{SP} + E_{disp} + E_{solv} + E_{thermo} + E_{ZPV} - TS \qquad \text{Equation S9}$$

G: Gibbs free energies; SP: single point energies; disp: dispersion corrections; solv: solvent corrections; thermo: thermal corrections; ZPV: zero-point vibrational corrections; TS: entropic energies at 298.15 K.

16b. Energies

Table S9. Absolute single point energies (SPE), zeroth point vibrational energies (ZPVE), thermal corrections (Thermo), entropic energy (TS), enthalpies (*H*), and Gibbs free energies (*G*) of **3-III**, **3a**, **3b**, **3c**, **3d**, **3-IV**, **3e**, **3f**, **3g**, and **3h**. Entropic energy was calculated at 298.15 K. Numbers are in Eh.

	SPE	ZPVE	Thermo	TS	*H*	*G*

**1-IV**	−5309.669	0.714	0.045	0.114	−5309.668	−5309.782
**1-V**	−5309.199	0.702	0.045	0.113	−5309.198	−5309.311
**2-I**	−5542.245	0.819	0.052	0.125	−5542.244	−5542.369
**2-II**	−5542.050	0.818	0.052	0.126	−5542.049	−5542.175
**2-III**	−5541.851	0.817	0.052	0.127	−5541.850	−5541.976
**3-III**	−5842.461	0.839	0.056	0.135	−5842.860	−5842.995
**3-III^{TS}**	−5919.312	0.863	0.059	0.140	−5919.311	−5919.451
**3a**	−5919.321	0.861	0.059	0.140	−5919.320	−5919.460
**3b**	−5918.688	0.852	0.058	0.139	−5918.687	−5918.826
**3b^{TS}**	−5918.685	0.852	0.058	0.139	−5918.684	−5918.824
**3c**	−5918.700	0.850	0.059	0.140	−5918.699	−5918.839
**3d**	−5768.296	0.851	0.058	0.140	−5768.295	−5768.429
**3-IV**	−5768.107	0.846	0.055	0.133	−5768.106	−5768.239
**3e**	−5844.609	0.868	0.059	0.142	−5844.608	−5844.744
**3f**	−5844.142	0.868	0.057	0.135	−5844.141	−5844.277
**3g**	−5843.906	0.846	0.057	0.136	−5843.905	−5844.042
**3h**	−5843.456	0.847	0.056	0.133	−5843.455	−5843.591
**3-V**	-5767.624	0.834	0.055	0.133	-5767.623	-5767.755

Table S10. Relative enthalpies (ΔH) and Gibbs free energies (ΔG). Numbers are referenced to **3-III** and are in eV.

	ΔH	ΔG
**3-III**	0.00	0.00
**3-III^{TS}**	0.85	1.28

**3a**	0.59	1.05
**3b**	1.50	1.51
**3b^{TS}**	0.09	0.06
**3c**	−0.31	−0.34
**3d**	−0.15	−0.60
**3-IV**	0.65	0.64
**3-IV^{TS}**	1.20	1.56
**3e**	−0.57	−0.05
**3f**	1.50	1.49
**3g**	1.91	1.88
**3h**	1.04	1.06

Table S11. Relative enthalpies (Δ*H*), Gibbs free energies (Δ*G*) and pKa of **1-V** and **3-V**. Numbers are referenced to **1-IV** and **3-IV**, respectively.

	Δ***H***	Δ***G***	**pKa**[a]
**1-V**	1.59	1.61	1.61
**3-V**	1.94	1.95	1.95

[a]$pK_a = 0.434 \times \Delta G/RT$

Table S12. Redox potentials calculated against the Fc/Fc^{+} couple using the energies ($E_{1/2}(\Delta E)$), enthalpies ($E_{1/2}(\Delta H)$), and Gibbs free energies ($E_{1/2}(\Delta G)$) for the **2-I/2-II**, **2-II/2-III**, **3-I/3-II**, and **3-II/3-III** couples under alkaline conditions. Numbers are in V.

	$E_{1/2}(\Delta E)$	$E_{1/2}(\Delta H)$	$E_{1/2}(\Delta G)$
**2-I/2-II**	0.69	0.69	0.68
**2-II/2-III**	0.79	0.79	0.80
**3-I/3-II**	0.27	0.27	0.27
**3-II/3-III**	0.29	0.29	0.29

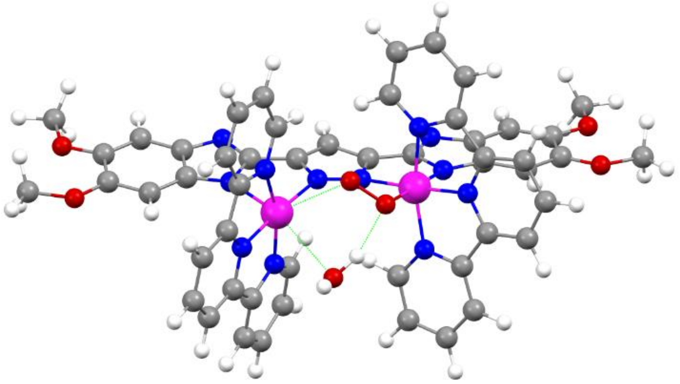

Figure S35. Optimized TS structure for the **3-III** → **3a** step (**3-III**^{TS}). Color code: Co, pink; N, blue; O, red; C, grey; H, white.

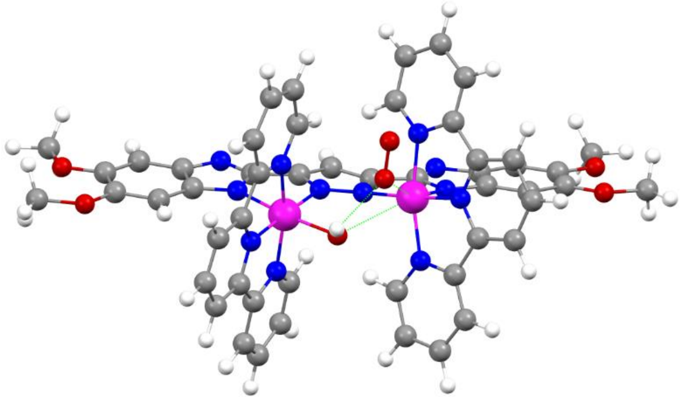

Figure S36. Optimized TS structure for the **3b** → **3c** step (**3b**^{TS}). Color code: Co, pink; N, blue; O, red; C, grey; H, white.

16c. Coordinates

1-IV

Co	2.43162231089082	2.23599357883114	6.54449641094781
Co	1.04871995252903	4.49427349201389	4.32080561231549
C	4.66763613699531	1.82320949598813	4.64596958040384
H	4.86334473386777	2.87579720346378	4.82008074430957
C	5.51336264217845	1.03871632534621	3.85645864553438
H	6.38883258181551	1.48931280129347	3.39553928376655
C	5.22744151332960	-0.32285340178395	3.69708696840148
H	5.87152962893030	-0.95935977148319	3.09426965492759
C	4.11560725434052	-0.86531012169014	4.35363425760818
H	3.89122052082642	-1.92601206535538	4.27452511522927
C	3.31626813860342	-0.03493479957043	5.14612615373589
C	2.19104251006583	-0.47804566067732	5.98004232470657

C	1.60163100151473	-1.74750895779120	6.05439988913936
H	1.95326183247429	-2.56590071363547	5.43194196631310
C	0.54085323766907	-1.94205350057839	6.95230695364827
H	0.07026283479471	-2.91999461297758	7.02614075989301
C	0.08147589510112	-0.88733476605574	7.75482522583909
H	-0.74157634509668	-1.04190234665284	8.44723936715594
C	0.70674107505044	0.36263589531850	7.64368100847855
C	0.44476278786552	1.58953169697653	8.40535182406373
C	-0.43884535081617	1.69899646230995	9.48360194568647
H	-1.05599732710668	0.85232841350735	9.77329094163804
C	-0.49001205306876	2.89601276349633	10.20889689234361
H	-1.15664026463405	2.99031929986611	11.06326017519908
C	0.35418726955849	3.94959293723509	9.84005214105050
H	0.37739991096799	4.87906242776787	10.40332695218109
C	1.20189339203135	3.78828307026848	8.73945037771311
H	1.89065925590126	4.56796068509017	8.43212062681412
C	4.66765228498325	3.55805708735160	7.78517092213603
C	4.54628533309834	1.53456083587193	8.69374546962816
C	4.30258020694941	0.20220480131891	9.15536883036624
H	3.48372132218465	-0.39177417758431	8.75961470639807
C	5.15459517146495	-0.29128233166492	10.12509640451296
H	5.01589231253982	-1.29747963048542	10.51293874947796
C	6.23339617223762	0.49839703151709	10.64331135158649
H	6.87738530587517	0.06560938416660	11.40641532230950
C	6.48896714450573	1.82112348765429	10.19713336881884
H	7.31163687718897	2.40606060210038	10.60152531326046
C	5.64534639287104	2.33406972813465	9.22518498269159
C	4.12611823990542	4.51676958992284	6.85335724382808
C	4.29156307128395	5.82702802766242	6.34486506908263
H	5.04172919108634	6.55762822993709	6.62022878671911
C	3.25075591988763	5.96477348321073	5.39609131734635
C	2.64372248546507	6.88574172615965	4.46631341581816
C	1.93013583936871	8.42242867575373	3.17884763065356
C	1.73826343930068	9.60091465679800	2.47620206793259
H	2.40161758438761	10.45302989427605	2.60399509568122
C	0.63727163871474	9.64916990198385	1.58211855277688
H	0.45850068888734	10.56057182462832	1.01485283912438
C	-0.25185358901368	8.53923837594450	1.39840261540009
H	-1.07677617527233	8.64621284260322	0.69829035669894
C	-0.07725411358171	7.35547372178987	2.08963165254349
H	-0.74942438397163	6.51263642000623	1.95657450434259
C	1.02922790518979	7.28919030131017	2.99456446424904
C	3.28229074340526	3.96044221051298	2.45158884337731
H	3.89868121412432	4.36864643354923	3.24530327975616
C	3.82326067178368	3.59501332900357	1.21544485366664
H	4.88985368119436	3.71175371874816	1.04056727873125

C	2.97157713872032	3.10812510762728	0.21609386414712
H	3.36328049677536	2.82254510480712	-0.75773745489246
C	1.59782690906517	3.02415754106558	0.47541290327010
H	0.91045106420374	2.68243767610441	-0.29430507558922
C	1.11171691258244	3.42092160220047	1.72545800096486
C	-0.30523094724255	3.49094756606504	2.10626678201176
C	-1.42864456482972	3.06400871743673	1.38511678494738
H	-1.32373954161728	2.59363466073235	0.41118824374024
C	-2.69899271661461	3.25449373520596	1.95014264218791
H	-3.58420721233429	2.93151330239935	1.40669125887671
C	-2.83783429349177	3.85657701732818	3.20950307282051
H	-3.82329115059881	3.99981054258105	3.64431601166633
C	-1.68225904827462	4.26792683478493	3.88854556978042
C	-1.57416450308927	4.96152754690798	5.17752822173954
C	-2.65048387843293	5.44269008402400	5.92970435076008
H	-3.67101571220122	5.24836337173843	5.60982003719326
C	-2.39836262535860	6.21087989245650	7.07324713266357
H	-3.22445554651244	6.61130270227971	7.65674233117401
C	-1.07237455859978	6.48475702978601	7.42791496214063
H	-0.83422628071539	7.11451217985397	8.28147761120194
C	-0.03749115759714	5.95686467618826	6.64947437919874
H	1.00409407868743	6.16275446060415	6.87256593918527
N	3.58305815198603	1.30933706161969	5.26067996409614
N	1.73448563545339	0.52027487668115	6.77550704833638
N	1.23166260612400	2.64801433806036	8.02473734880878
N	3.92143251148485	2.29669001353227	7.78062311697477
N	5.66901227028266	3.59020399370901	8.61750291937000
N	3.04889283059558	3.94595575226004	6.22594056243321
N	2.53924650883679	4.79309785018561	5.36668915657273
N	1.47112969644322	6.31027561185986	3.80157226254121
N	2.91915052655997	8.10859870920107	4.11229471678375
N	1.96359005735834	3.85765627133421	2.71288227714724
N	-0.46919288397426	4.08126769146302	3.31537288217605
N	-0.27823636618559	5.19552880893714	5.56549478945146
O	0.99827893284990	2.69370311295855	5.21353624734748
H	1.08691900703638	2.03596854331707	4.49126205081211

1-V

Co	2.42095855903563	2.36301651625815	6.51053147381609
Co	1.12682304893069	4.47892180174229	4.42728278539554
C	4.68381639539094	1.94263827570749	4.66184084504962
H	4.95932939809324	2.95440114282064	4.94110337898624
C	5.47511766492686	1.17515453977116	3.80228676542689
H	6.38915846149254	1.59800275335541	3.39352410905016
C	5.08172428885030	-0.13441697439308	3.50429590706367
H	5.68039649782902	-0.75744584497753	2.84391199947366

C	3.92196009433710	-0.64721982046585	4.09632838385647
H	3.61470829075996	-1.67363942024711	3.91365297498626
C	3.17971476658835	0.16421553672628	4.96040647401769
C	2.01274213234974	-0.26518080875114	5.74119634482618
C	1.32082654589344	-1.48248191128881	5.68539520474250
H	1.61724743340333	-2.26274848808735	4.98992069229244
C	0.23005392121086	-1.67208825859246	6.54579344240459
H	-0.31836855784083	-2.61072838809702	6.52119514430860
C	-0.16551307432832	-0.65957716614115	7.43130240240123
H	-1.01945275756644	-0.80317821224347	8.08740725248356
C	0.56279183473729	0.53751630954915	7.44594402950318
C	0.35791614778096	1.72618949325896	8.28276076679624
C	-0.54596264043390	1.82605641987187	9.34517402913171
H	-1.21394220303404	0.99964052512388	9.57379540279493
C	-0.55141014443576	2.98353755740606	10.13185876102585
H	-1.23598250372830	3.07058907608765	10.97234364735094
C	0.35888161977299	4.00644422443886	9.84329480544397
H	0.41338252445773	4.90438797904627	10.45314672251959
C	1.22829211516032	3.85494281535278	8.75924359606508
H	1.96828641835694	4.60808244327839	8.50887671590271
C	4.70096243227857	3.60403002504629	7.82707951694363
C	4.55695973874724	1.57372727585024	8.68452505578701
C	4.29114868820381	0.24083880826049	9.10334159451063
H	3.46511837437156	-0.32976378161423	8.68575657045665
C	5.13284761390750	-0.29455643903229	10.06505499414815
H	4.97416873818709	-1.30975068479945	10.42081830636304
C	6.21784218607802	0.45853615578655	10.61024599204284
H	6.84901035492933	-0.00832402141071	11.36318473838323
C	6.49441173999820	1.77898633580671	10.20589151646096
H	7.32313581447065	2.34188143735491	10.62760014592809
C	5.66129458984992	2.33745473551404	9.24022617895376
C	4.18203268359946	4.59630584839703	6.91261684895998
C	4.34497194358241	5.90762804041160	6.39606893289424
H	5.09797544026858	6.63563379448012	6.66736663354034
C	3.30194170398752	6.05323486346779	5.44554185976590
C	2.67411015900410	6.94120332815781	4.49244112324474
C	1.94871003000400	8.43405613908473	3.16205828675113
C	1.73482985566944	9.59074818583813	2.41712435012728
H	2.38382521479578	10.45625590545862	2.52281267103067
C	0.64504953298250	9.59072067786394	1.52487552917807
H	0.44958673378158	10.47850266528289	0.92743837165621
C	-0.21806246682893	8.46203545379209	1.37459397343836
H	-1.04195267851054	8.52791117820296	0.66800204289859
C	-0.02420277572077	7.30074237803068	2.10515315936564
H	-0.67931923069631	6.44005473467981	1.99435564296055
C	1.07262439914639	7.28483449697821	3.01028925148787

C	3.35179810949840	3.98650031075280	2.55140455167382
H	3.94569817437951	4.51923960034598	3.28680530371419
C	3.90239907059119	3.52844375781880	1.35088442393170
H	4.95542494199103	3.69999599432460	1.14364405397172
C	3.07575816028211	2.87479086026728	0.43008317303620
H	3.47443810608254	2.51252143849192	-0.51461583495116
C	1.71623463001277	2.72267233452718	0.72515048109893
H	1.04396561809318	2.25440411041722	0.01091285033482
C	1.22079306967635	3.21495915437133	1.93671444092168
C	-0.19116422879166	3.22886100511897	2.33818929885374
C	-1.29097392722457	2.64883886505750	1.69204567385908
H	-1.16635856405526	2.08783608492858	0.76999073267468
C	-2.56117114658542	2.80335987591111	2.26517263966795
H	-3.42882721421441	2.36440370957920	1.77838624333493
C	-2.72195182452262	3.51065442658290	3.46500227708094
H	-3.70509293752479	3.61661855722341	3.91508257227020
C	-1.58768466441930	4.06995522527139	4.06855930676090
C	-1.49902031535942	4.85338787491683	5.30696156260987
C	-2.58655312948778	5.32082048657023	6.05174039088000
H	-3.60042638203360	5.06143419626813	5.75831831726411
C	-2.35452570526762	6.15696551509596	7.14967239320771
H	-3.18967267901079	6.54492452390226	7.72815781180885
C	-1.03879764361137	6.51290400751554	7.46719902777733
H	-0.81855233827503	7.19102478826290	8.28755946491018
C	0.00790576338434	6.00039311448274	6.69546510780214
H	1.04282020249885	6.26365109942679	6.88826597584425
N	3.55481193277286	1.45901833836025	5.20964908497173
N	1.62997560114457	0.67985413233330	6.62869412063695
N	1.21287480293665	2.75554686324665	7.98507958280367
N	3.93883641156653	2.36723609296833	7.78445549947310
N	5.70680837025482	3.60618560315609	8.66688367380377
N	3.10021802423370	4.03877247044266	6.28338225476791
N	2.59469098390084	4.88010693588472	5.42962325089344
N	1.52302898366665	6.32936477291003	3.85020872229093
N	2.93762284527424	8.16521988192025	4.10584662671521
N	2.05129472809143	3.81566487816956	2.84686222271428
N	-0.38037679933335	3.93426507911985	3.47568280134528
N	-0.21315954166798	5.17543848512182	5.65657425639053
O	1.10817570086151	2.77292314179951	5.22478280487837

2-I

C	16.21148047663188	8.18622921175102	22.75096027764360
H	17.12260272478724	8.16579706118132	22.16833022181908
C	14.94314420812008	8.01119972573083	22.19130337277610
H	14.84717924696927	7.84159685341530	21.12195880451831
C	13.82080159981398	8.06011376021187	23.02497301842931

H	12.82089154083367	7.92794658344116	22.61814596190598
C	13.99746387243263	8.28604108956224	24.39421083135586
H	13.14474674621143	8.32735473737394	25.06695211957430
C	15.29332725021333	8.45493332894318	24.89044253559086
C	15.65793722174007	8.70331496893730	26.28865160856921
C	14.82329700945469	8.88173936767908	27.39724361498477
H	13.74318049262923	8.82157564979319	27.29648451783356
C	15.41169716967425	9.14999655030320	28.64196824551673
H	14.78133996377603	9.29365701490746	29.51615972115514
C	16.80314802626160	9.24026757484423	28.76687307060899
H	17.25933982243812	9.45650441904495	29.72877314337325
C	17.59243475078673	9.05717963993754	27.62324971188092
C	19.04909309978251	9.15210165156348	27.49188728510914
C	19.92899203518336	9.51332175756595	28.51828256804910
H	19.54588587170389	9.71456568349269	29.51523476860931
C	21.29160288684883	9.63470020508888	28.23784338284468
H	21.98943939471543	9.92350416434068	29.02028004746099
C	21.73733739246533	9.40173900463703	26.93103639920916
H	22.78479206245805	9.50775425090301	26.66159370515910
C	20.81197307999581	9.04080544760345	25.94972630059737
H	21.11444158384073	8.87481158557198	24.91970139689296
C	20.90403457070332	5.62548231106913	25.45315070364590
H	21.02662140042129	6.67245162057530	25.21026389321251
C	21.41583444398334	5.04766288736546	26.61807728883555
H	21.96199914728350	5.66416973638691	27.32732948349865
C	21.21286099454949	3.68213999236569	26.84413233888109
H	21.59997587143606	3.20426815683975	27.74111725337711
C	20.50611114965620	2.93190468677337	25.89823197745529
H	20.33060018031064	1.86962096824574	26.04723408761810
C	20.02152732857099	3.57044037773210	24.75316460865701
C	19.26758634186462	2.93910585645161	23.66569918582160
C	18.92169160120948	1.59349762582563	23.49969775422900
H	19.19440361410304	0.85265893168861	24.24621256979458
C	18.22262707333645	1.21968853311051	22.34238534789777
H	17.94580275912035	0.17896896212782	22.19259517693389
C	17.88396565597167	2.17431603667303	21.37589646249010
H	17.34979366159070	1.88476530428701	20.47533776976515
C	18.25763456407210	3.50771342511634	21.59099825681193
C	18.06309997496084	4.66513451363775	20.71316410074287
C	17.49695042827735	4.62036727406999	19.43432327755545
H	17.12791636701413	3.67836168138521	19.03715828700041
C	17.43705216926539	5.78966018616070	18.67320936131181
H	17.00907522279357	5.77281387974630	17.67362941660507
C	17.95787466293911	6.97395385886890	19.20910253099549
H	17.95539345577018	7.90198452118564	18.64363670716661
C	18.50761027298404	6.95694968637337	20.49250393110921

H	18.94326744655601	7.84813359587095	20.93494767173167
C	21.74620013178387	4.62610024784675	21.07581601565465
C	21.63688074851575	3.23917262272334	20.88177885827607
H	20.94549214553922	2.63038533300025	21.46308029798966
C	22.45535428510889	2.62854480068786	19.92670783898129
C	23.39716698653264	3.39871854184808	19.17017681375593
C	23.50792902180752	4.77710486872126	19.37336962449801
H	24.22972907278820	5.36416935363108	18.80685605240104
C	22.67797748216171	5.40569081322779	20.31662582976103
C	21.64662408953902	6.74503832842405	21.60852652537507
C	20.98835544856898	7.84749786585128	22.27637962335650
C	20.94085446847641	9.25562175508484	22.21229934571082
H	21.61329602525260	9.91647715796442	21.68310160559720
C	19.82066499683128	9.60915774392165	22.99288720921340
C	19.24849475793263	10.78796551384628	23.60760020627352
C	18.82126436970015	12.67629788118111	24.48959924694834
C	18.76086207348382	14.02421466815971	24.88027196249052
H	19.40441442466348	14.75975046604558	24.39920542111144
C	17.87329129197527	14.40848334546704	25.88946736147800
C	17.01890763422546	13.44008681397709	26.50963008039502
C	17.05528141897723	12.09899358857843	26.11506265516390
H	16.37824801067046	11.38434766892915	26.58153800161828
C	17.95748502584743	11.71613860504939	25.10887688700335
N	21.10961450661301	5.50305074961579	21.92557801363873
N	22.59435109131930	6.74495563813965	20.67091118294449
N	19.97278379084957	7.40920725727680	23.08706896550544
N	19.22405191773480	8.46319012642397	23.45250139364562
N	19.49895044201009	8.89916963453283	26.22101696410166
N	20.23059665127297	4.90475523624610	24.54645909286650
N	18.92701179962989	3.84270441931741	22.71729006393837
N	18.54526019585127	5.83582636507327	21.24047823010347
N	19.63039286282862	12.06347239601599	23.54276267441493
O	18.12609668675708	6.68118748502206	25.14551937374846
O	17.84992940484648	6.00937917756035	23.96426871023507
N	18.22442897190604	10.50717058736579	24.50492016232336
C	17.81052429821087	15.85689684044050	26.32577483678019
C	22.34525992480884	1.13559717396907	19.70120207581522
C	24.28200240176528	2.71416375382524	18.15005784770974
C	16.05954160075958	13.86663602538132	27.60001899266727
Co	18.07168826066707	8.58959658705365	24.94255518721114
Co	19.42945390832107	5.60638267345485	22.95109352243385
N	16.37866588945126	8.40538036094294	24.06222874904233
N	17.00048377571701	8.79465728956710	26.43591869441575
H	18.52563846275819	16.46535470018047	25.75939409029606
H	16.80845751263444	16.28599369178379	26.17224852618116
H	18.04396377271449	15.97237703815859	27.39552289381850

H	16.58642220106650	14.31441543893257	28.45664892029509
H	15.34387404290644	14.62246420832274	27.24244326227310
H	15.48259387096119	13.00854803400507	27.97033010935110
H	24.90510365906675	1.93244688718233	18.61077538319492
H	23.69202545660496	2.22453721895294	17.35987644440841
H	24.95198066859490	3.43652158913244	17.66860842358102
H	21.57726043181825	0.69772735657574	20.35300381401591
H	22.07954952840980	0.89731018777698	18.65990899716870
H	23.29507621224246	0.61986698984439	19.90891486420016

2-II

C	0.14976784526761	-0.06706676126437	0.29288183078139
H	1.23093287662028	-0.09734467174669	0.32616464594412
C	-0.57505735013622	1.12118711856042	0.41463405403469
H	-0.04657371914104	2.06179922120087	0.54449746077067
C	-1.97264985476588	1.07128678008250	0.37254446045687
H	-2.56403131665685	1.97882478864328	0.46716952169118
C	-2.60481100987677	-0.16682125116170	0.21636776252812
H	-3.68882901440606	-0.23770609355376	0.18669996943423
C	-1.82149474088230	-1.31874969752391	0.10213591630765
C	-2.32329514172940	-2.68797361890873	-0.04794736143603
C	-3.64864606662205	-3.13337076104557	-0.11923375120119
H	-4.47526908784772	-2.42992133830995	-0.07376560961695
C	-3.88724945481148	-4.50800925661038	-0.25536857562831
H	-4.90896857313636	-4.87496767818467	-0.31411557441047
C	-2.81923200891341	-5.41193419942695	-0.32258156452447
H	-3.00248271925552	-6.47669283842941	-0.43509226003464
C	-1.51244885070294	-4.91241458745940	-0.24416937910790
C	-0.24838479244777	-5.65632864701227	-0.25886660061141
C	-0.13561464823201	-7.05076144540462	-0.27280791330147
H	-1.02834742854039	-7.66823040679017	-0.32561614609326
C	1.13152443376762	-7.63450921604343	-0.19078873222359
H	1.23841984138308	-8.71662397015767	-0.19060485673867
C	2.25303797813270	-6.80401560802228	-0.08082859137479
H	3.25481077993154	-7.21384178181819	0.01805732688746
C	2.07487294608361	-5.41838130703716	-0.08077926294918
H	2.91592752740193	-4.74049105490012	0.02751627131252
C	2.79815693174886	-4.30741664681523	-3.23579158387416
H	3.05569816391663	-4.32845851372161	-2.18515093435604
C	2.70546116783158	-5.46416434237142	-4.01380849868170
H	2.89375929197458	-6.43350439242006	-3.56020169669760
C	2.37602456646495	-5.34590729667215	-5.36835620258572
H	2.29939632333076	-6.22738292591809	-6.00035916718849
C	2.15313940844758	-4.07392993629755	-5.90644668882249
H	1.89969605653878	-3.95213686211843	-6.95620837917305
C	2.26249201733831	-2.95643603859345	-5.07414177607579

C	2.07469151653622	-1.56004722621225	-5.47864940088664
C	1.74879357015744	-1.04952026957705	-6.74092564359391
H	1.58701411895035	-1.71367058647842	-7.58522105267347
C	1.63146453426061	0.33908280174916	-6.89204206851712
H	1.37781699503893	0.75652899087512	-7.86333187055815
C	1.83369387441608	1.19274928175514	-5.80009379728727
H	1.73909757212216	2.26877553874442	-5.91523094588956
C	2.15940721640566	0.62892145528676	-4.55949777310863
C	2.44998067834557	1.31254287272592	-3.29469066739724
C	2.52439790035550	2.69975329409560	-3.12815299889403
H	2.30948240114296	3.36019258614786	-3.96404503231304
C	2.90260200796819	3.21905945556552	-1.88706165787560
H	2.97539841953239	4.29413459884008	-1.74101378926181
C	3.21175074035101	2.33284413083049	-0.84844769704941
H	3.54183529447763	2.69090763625170	0.12322628124620
C	3.10963528332598	0.95806609917206	-1.07545525009550
H	3.36528277380599	0.23714475342792	-0.30491869133147
C	5.64638217459701	-0.94458945904135	-3.64482127869305
C	5.67908895157641	-0.51316748553711	-4.98690195713427
H	4.77704105047426	-0.48087585132761	-5.59553720193913
C	6.90177834756837	-0.13490597186318	-5.53012236000516
C	8.11811133961712	-0.18454646435015	-4.73565429004103
C	8.08435324641378	-0.61088549457183	-3.40838892310060
H	8.99366922873477	-0.65010986817733	-2.81213922103946
C	6.85539139413539	-0.98934722768006	-2.84419051261725
C	5.25363458836561	-1.62752227605734	-1.60894735032957
C	4.34146348431482	-2.02582571056577	-0.57415182216183
C	4.32985785934574	-2.28985806441014	0.80808318839375
H	5.17329391918075	-2.31820793935061	1.48389931954145
C	2.97327087254743	-2.50775188130815	1.11286046792840
C	2.14507777950717	-2.90364764609530	2.21662296909519
C	1.25785070025405	-3.54597274832433	4.03303090715269
C	0.95497186824981	-3.94013179714092	5.34626876536752
H	1.73478716360248	-3.95532078331990	6.10498129946780
C	-0.35275074667470	-4.31071343716680	5.65838023209518
C	-1.39277491717032	-4.28758319009569	4.64308445000361
C	-1.10880280862854	-3.89431203787418	3.34009135728769
H	-1.89870833425236	-3.87252425652359	2.59115954537348
C	0.21354256945031	-3.51841055340176	3.02629566096048
N	4.63374692861016	-1.37128930603223	-2.84351247581925
N	6.57075725803331	-1.42078996937956	-1.56709214506905
N	3.04474421974255	-2.09753135435741	-1.05054176938611
N	2.22988356337804	-2.36704660087393	-0.04521015440345
N	0.85813246704961	-4.85032517491538	-0.19098772880219
N	2.58135959764194	-3.08849556662746	-3.75080165453968
N	2.27832156558956	-0.71292113398875	-4.44305666805627

N	2.71585670863837	0.45334962853152	-2.26080579733531
N	2.46153762273587	-3.15615980945494	3.48771386022827
O	0.37807789327703	-2.77142170537290	-1.97056893797398
O	0.78346143659023	-1.52796250633531	-2.40853867103759
N	0.79551701592261	-3.09080308122481	1.87406022984659
C	-0.69672496508576	-4.74003657461820	7.06336948050798
C	6.97229569186596	0.33019312969498	-6.96500304473480
C	9.42806757957289	0.22982353870694	-5.35863638599890
C	-2.79951037125879	-4.69245292592601	5.01328212060580
Co	0.42838471165770	-2.94849451358957	-0.07122959614720
Co	2.65318306393587	-1.43193912366879	-2.77718559404259
N	-0.45597678041712	-1.25317292265840	0.13726627485758
N	-1.31008346385386	-3.58349696863134	-0.09884268820831
H	0.18764399379582	-4.71328766310931	7.70882737128214
H	-1.46274367045338	-4.08694054092914	7.50777300121399
H	-1.10364728689172	-5.76215109702421	7.08399335964907
H	-2.83606488467242	-5.72603623150732	5.38771622343074
H	-3.20541608009076	-4.05488877370564	5.81215012534870
H	-3.46968968491103	-4.61983709285142	4.14803754024206
H	9.67330428334279	-0.39195973357752	-6.23278183640887
H	9.39321838686532	1.27099675228194	-5.71320944947150
H	10.24926385400580	0.14320823299071	-4.63925273547567
H	5.98040048480236	0.31323869512880	-7.43301364563912
H	7.36669186192574	1.35436590393279	-7.03808455309361
H	7.64106934224687	-0.30675027813404	-7.56215630793876

2-III

C	0.16007198729432	-0.06133955300125	0.40306430974294
H	1.24001274103808	-0.09306418175116	0.47294192754572
C	-0.56478112535025	1.12443101564890	0.54929266127382
H	-0.03889484312297	2.05667321011907	0.73673435865760
C	-1.96067898533526	1.08262557880520	0.46019600770345
H	-2.55224005852260	1.98807444739194	0.57234025919711
C	-2.59113196180687	-0.14649036819913	0.23697834263834
H	-3.67409990812828	-0.21040188742102	0.17329798136660
C	-1.80888893973919	-1.29731577790486	0.10363414778739
C	-2.31516259844995	-2.65748735054344	-0.10610187525049
C	-3.64095056846360	-3.08249872338113	-0.26112290489081
H	-4.46088290443804	-2.37020729291169	-0.23573066202866
C	-3.88890666775732	-4.44728112411672	-0.46279552646032
H	-4.91051760038970	-4.79710027883425	-0.58988502339168
C	-2.82880082867865	-5.36235341745814	-0.51467927957445
H	-3.01923872588762	-6.41809612904344	-0.68559485455965
C	-1.52187823598163	-4.88412282808674	-0.35117005131993
C	-0.26685918834585	-5.64465988484012	-0.35474415040810
C	-0.17109961679636	-7.03849688419357	-0.42784955010582

H	-1.06844154642603	-7.64175894449185	-0.53703818655297
C	1.08584854567897	-7.64416453933016	-0.33243979331214
H	1.17786242783436	-8.72686840971444	-0.37557485177693
C	2.21466489559770	-6.83580671259904	-0.15416844853956
H	3.20714105064802	-7.26499869520674	-0.04283264692027
C	2.05424036832692	-5.44846221954143	-0.10194328581798
H	2.90174953863074	-4.78823205449958	0.05370964520625
C	2.92824420820290	-4.31557005428623	-3.21993804642509
H	3.21783768255714	-4.32440141539384	-2.17681846472897
C	2.87202008473282	-5.47837007635308	-3.99284587197088
H	3.12461033778813	-6.43542565739518	-3.54460698317118
C	2.49889320714693	-5.38185906140377	-5.33803903755382
H	2.44865367227184	-6.26831168481421	-5.96574438916645
C	2.20077031918235	-4.12359248244240	-5.87293819848302
H	1.91587684697297	-4.01918935388659	-6.91649493246988
C	2.28019370437643	-2.99807195744603	-5.04776453178827
C	2.02472445710377	-1.61365453602682	-5.45829172803813
C	1.61683827504427	-1.13428005834144	-6.70970537944204
H	1.44283223146236	-1.81569331240645	-7.53772063760224
C	1.42629340201215	0.24517975008672	-6.87005123402921
H	1.10586039762751	0.63745080444634	-7.83217815761911
C	1.63468830534602	1.12127670263446	-5.79630462813622
H	1.47527585187345	2.18905966089797	-5.91687012824681
C	2.04397263111516	0.58913776047200	-4.56631543848029
C	2.33482720692186	1.30231032344977	-3.31702942413643
C	2.33725496681586	2.69314684517004	-3.16630010356969
H	2.06686306410309	3.33249056437881	-4.00246683731369
C	2.71501931448696	3.24745132257862	-1.93917594426394
H	2.73388972768273	4.32671661844488	-1.80729820626906
C	3.09119505929667	2.39191227755205	-0.89703995523870
H	3.42133089872347	2.77993440971530	0.06321076278444
C	3.05533132619696	1.01048560327937	-1.10663850590418
H	3.35672726009460	0.31366404006692	-0.33089695713446
C	5.67816930974884	-0.92722245532838	-3.61624198869765
C	5.72800668521472	-0.51518802413854	-4.97619727401471
H	4.82602228142932	-0.47840089908763	-5.58305655641135
C	6.95224278742731	-0.16802097045838	-5.51772112708692
C	8.16713332473130	-0.22742191650054	-4.70068693933500
C	8.11724928060523	-0.63473382739897	-3.34895588510225
H	9.02542413363523	-0.67926325219135	-2.75237305140897
C	6.88880886442291	-0.97719142200865	-2.80039678307280
C	5.27878116092989	-1.59168953669292	-1.56940306632301
C	4.35118822141266	-1.99304037999404	-0.54849679129152
C	4.34650103188035	-2.27090497509494	0.83178106702927
H	5.18949324495881	-2.29454068600942	1.50942963829709
C	2.99050897577068	-2.51067909535279	1.12584597974553

C	2.17853924435344	-2.91274368508178	2.24047685759200
C	1.29775362487847	-3.53699113000941	4.06264064164094
C	1.01054057588794	-3.89796908001630	5.37224423917066
H	1.78822011637963	-3.89874917836701	6.13249521470118
C	-0.31349080674332	-4.26384387824070	5.70081813313222
C	-1.37248944758537	-4.26090705136150	4.68738802891256
C	-1.09113580852830	-3.89591110801731	3.38359834613669
H	-1.87663941892139	-3.88668908051164	2.63104438299029
C	0.24153848110920	-3.52629760475932	3.05363555788559
N	4.65995984583890	-1.33265615139925	-2.83805412748481
N	6.58116383097098	-1.39856016158292	-1.51443374225927
N	3.06117896026923	-2.07335236739803	-1.02804171073598
N	2.24873672333362	-2.37319076504077	-0.02817905333150
N	0.84853437984103	-4.85867772809031	-0.21880628589628
N	2.63582281987791	-3.10940737261331	-3.73052941339574
N	2.23976786070395	-0.74358835277653	-4.44371163942772
N	2.66995907600085	0.47116765762697	-2.27942552277125
N	2.50292008611880	-3.14611220536642	3.49655521210139
O	0.41705053665022	-2.77106560722509	-1.94587479792507
O	0.81227649768265	-1.53302421821115	-2.37924659710784
N	0.79907465622398	-3.11521466391724	1.90198398894119
C	-0.64097267175515	-4.65611152901301	7.11042045587512
C	7.05248577808296	0.27000844128360	-6.95714889261247
C	9.48575979196301	0.14479480110734	-5.30942832566973
C	-2.77256563543784	-4.65585839860477	5.08345232748149
Co	0.43855988706938	-2.94722191409461	-0.05443069495389
Co	2.66753932000026	-1.42953795193788	-2.77083582470602
N	-0.44321509924089	-1.23909252473225	0.17949948017168
N	-1.31160778782878	-3.56534766423118	-0.13569655367903
H	0.24675079810664	-4.63805722581001	7.74950025716024
H	-1.39388738762808	-3.97813486045810	7.54292316943273
H	-1.08093542398113	-5.66451297035724	7.14991401245205
H	-2.80199754474874	-5.68157212673076	5.47802613675174
H	-3.16522068015759	-4.00082210005038	5.87399697353112
H	-3.45300849686977	-4.60050795486603	4.22642430712517
H	9.46386867620398	1.17094320064689	-5.70795552432933
H	10.29982722624203	0.07069951304762	-4.58240211006123
H	9.72270579902298	-0.50857023230795	-6.16410425018702
H	6.06847811896425	0.26099478089375	-7.43902809778559
H	7.46477147781665	1.28577743179775	-7.03927162605908
H	7.71957853864643	-0.38974596463338	-7.52995025359415

3-I

C	16.20969777238818	8.17887170166201	22.76596361337440
H	17.11883320308236	8.16420975734283	22.17961372261782
C	14.93972475510269	7.99551444058008	22.21219134801198

H	14.83963345111164	7.82507671672858	21.14332586445277
C	13.82121386032134	8.03626576325459	23.05140241804998
H	12.82031563609505	7.89663995943948	22.64943167687856
C	14.00281692887517	8.26373488276232	24.41991606052615
H	13.15312802322246	8.29751924903016	25.09699933566192
C	15.29994130656279	8.44235051243333	24.90965284545818
C	15.66959948917549	8.69303505057620	26.30658180803749
C	14.83872999812834	8.86699244144528	27.41936495588204
H	13.75829664080489	8.80340339597229	27.32393590642395
C	15.43371120816361	9.13029088174805	28.66285550188710
H	14.80754590542678	9.26430372136038	29.54174092059551
C	16.82532707507611	9.22566313251100	28.78161530382054
H	17.28545241360417	9.43340865735625	29.74362361126731
C	17.61080995085584	9.05286658461715	27.63230657036640
C	19.06488078793314	9.15299752640129	27.49347187828817
C	19.94942568613251	9.51748825244281	28.51601145043023
H	19.56974492076116	9.72347210689351	29.51337721308058
C	21.31089539460703	9.63474413588976	28.23117300745856
H	22.01166322776535	9.92490549582513	29.01052758934647
C	21.75292076295633	9.39486035508064	26.92359961444080
H	22.80012603072291	9.49577096231662	26.65130507011944
C	20.82383051622999	9.03355404885622	25.94593126283023
H	21.12354699305056	8.86194337679149	24.91589640393417
C	20.89164327173920	5.60969265406748	25.46769394778458
H	21.02481723875709	6.65576524327840	25.22541468703802
C	21.38658354092104	5.02756405902052	26.63800345409521
H	21.93071797059698	5.63935937927181	27.35291977851114
C	21.16857773868528	3.66407478493556	26.86232538112257
H	21.54110897210222	3.18327902937946	27.76395936264098
C	20.46552105898614	2.91958616342054	25.90891861777031
H	20.27772906226343	1.85921516810292	26.05699912033794
C	19.99948512961468	3.56201624820285	24.75819560780671
C	19.25096129754098	2.93675718860617	23.66280722331009
C	18.89745347343793	1.59316417319275	23.49186164726356
H	19.16016263111168	0.84853476207799	24.23823461805236
C	18.19847639862902	1.22846853775157	22.33057631701804
H	17.91053100178948	0.19092093200976	22.17886807511350
C	17.87264917568733	2.18734234700186	21.36400770773076
H	17.33454327403370	1.90473763490954	20.46346235341026
C	18.25844653829385	3.51822722118212	21.58182192259662
C	18.07678784180246	4.67834288700811	20.70738161210030
C	17.51748309388258	4.64111988853695	19.42423054425794
H	17.15102646447933	3.70115545384318	19.01971901021241
C	17.46034300644801	5.81441894448285	18.67027685278588
H	17.03746057695079	5.80314557568816	17.66840000006805
C	17.97655720894903	6.99694827062870	19.21630224135586

H	17.97412199984153	7.92880233716308	18.65719828909550
C	18.52081107408176	6.97260029507948	20.50197365657608
H	18.95191176358574	7.86220685960286	20.95221563554779
C	21.74857288825459	4.63044760414188	21.07640371849413
C	21.63319612172527	3.23693748183587	20.89457389343876
H	20.94846409085645	2.64053015068628	21.48854437229432
C	22.45086852769043	2.64388571486806	19.93531280244638
C	23.38999602900761	3.41981037850375	19.16744919703881
C	23.50823567765948	4.79484307169923	19.35902751112796
H	24.22096822699202	5.39197682859859	18.79792455806635
C	22.67257421651240	5.41132556897375	20.31203649311654
C	21.65383692394961	6.74629102124376	21.61816552624350
C	20.99817940932074	7.84473284875348	22.28798958997320
C	20.94663113271147	9.25294037846285	22.21902242666722
H	21.61842273783537	9.91364388180892	21.68882774951729
C	19.82332946378133	9.60674484795218	22.99580669601022
C	19.25266020049164	10.78424074995696	23.60650566693915
C	18.82503217927245	12.67396472632344	24.48506681891616
C	18.76506111581703	14.03228287680356	24.85630431561690
H	19.40698973196973	14.75632323462172	24.36319090145671
C	17.87050353723625	14.40462502603342	25.85760023888220
C	17.01489680496494	13.43106693001562	26.48473649631410
C	17.04892797508794	12.08879903830603	26.11280995170345
H	16.38100330006293	11.36642146480410	26.57057151797559
C	17.96498182830523	11.71505105284343	25.10801415086837
C	25.07866281962224	3.40249999656320	17.49022216626884
H	24.58943118531953	4.17015533778869	16.87201301243134
H	25.54954492809253	2.65275582937287	16.84957329377229
H	25.83665858167481	3.87783475394115	18.13057785467224
C	21.54500018553274	0.48932874304629	20.37141583799261
H	21.69222644881070	-0.52675469881641	19.99577090768869
H	20.50051429814751	0.80194216191469	20.20652987827004
H	21.76125755131599	0.52267212538511	21.45166756614084
C	15.31121107964550	13.03204096500293	28.09497577931817
H	14.62510585947696	12.55798915199407	27.37433848509604
H	14.74039277788010	13.62655684190245	28.81327504939230
H	15.87361978754861	12.24482360653773	28.62399035549973
C	18.52488105376429	16.69043660759321	25.73980782341609
H	18.32525574086181	16.77861457017295	24.66142447626637
H	19.59336649016336	16.48010553603915	25.89780887399386
H	18.24923992983351	17.61877262942542	26.24632927011029
Co	18.08017182991733	8.58787841480967	24.94978167055149
Co	19.43364741254543	5.60679523169705	22.95520180002178
N	16.38136136885006	8.40014960236547	24.07608596145746
N	17.01189623581485	8.79207678826610	26.44709753082465
N	19.51129120034380	8.89595767384984	26.22091922781745

N	20.22205224212511	4.89447755320136	24.55413838096950
N	18.92542622232825	3.84391115582417	22.71316330665327
N	18.55682932817337	5.84731085033027	21.24334085649640
N	21.12064717862996	5.49976789529155	21.93467919361220
N	22.59420492609391	6.74618909698161	20.66998202958107
N	19.98480912060438	7.40622920159148	23.10459776855092
N	19.22380905197783	8.45900944522466	23.45284023783800
N	18.22927044661583	10.50712403569092	24.50913754766469
N	19.63168074245465	12.06295177829654	23.54025017575551
O	18.12950377132013	6.67830935484016	25.14973445550023
O	17.85393762297486	6.00955264952215	23.96633054822419
O	22.44970212749596	1.30468596814241	19.63563665451591
O	24.12078843620926	2.68511379607160	18.27324706764055
O	16.18830630197917	13.94851143938559	27.45040561304124
O	17.70779685398125	15.67763288575385	26.33296713299999

3-II

C	16.27164153521951	8.38364876170580	22.73083098433488
H	17.18182493830903	8.40289268122851	22.14624026910412
C	15.00359083256633	8.27221095427942	22.15440557015481
H	14.90898872329200	8.19741347506426	21.07436109224460
C	13.87844146013330	8.26334047569058	22.98575713262060
H	12.87870540966824	8.17931993659762	22.56648425204121
C	14.05318383622314	8.37195088739473	24.36978720298718
H	13.19725837547100	8.36998263816294	25.03948336647509
C	15.34873886142654	8.48304818401649	24.88261562432887
C	15.70759756044528	8.61578636182115	26.29837229443273
C	14.86732267463584	8.66693265677701	27.41718362444194
H	13.78892342875917	8.59292008228908	27.30797719710208
C	15.44824516852938	8.80864308695404	28.68558756066552
H	14.81392922644991	8.84523447688752	29.56792631894479
C	16.83947018804771	8.89418821642076	28.82621295596068
H	17.29019896460717	8.99580977629156	29.80942522817391
C	17.63424780208830	8.83927177341329	27.67302860315780
C	19.09410068521896	8.93275130863553	27.56339872743469
C	19.96837541623052	9.17522632323465	28.62859136336909
H	19.58124731924771	9.26877534130571	29.63975318241970
C	21.33484720353585	9.31679460816300	28.37159436519198
H	22.02867603665042	9.51359199491605	29.18537874454223
C	21.78809923219035	9.22588038377606	27.05022585882794
H	22.83778790439084	9.35700520993944	26.80094316372810
C	20.86618585630711	8.97667145794498	26.03036911740871
H	21.17340966918118	8.92204371693805	24.99015365850253
C	21.04746920247019	5.62532300290366	25.32110670869617
H	21.20445707980932	6.66370980388777	25.06079834699982
C	21.61261540506999	5.03771129894798	26.45612395502648

H	22.23493350987603	5.63560691456310	27.11662297003257
C	21.36508837758968	3.68538789294907	26.71510745471842
H	21.79181974329209	3.19989026053453	27.58948535775742
C	20.56374170038455	2.95794395207665	25.82835721430189
H	20.35611177426502	1.90545787179051	26.00252703328794
C	20.03021663085537	3.60506198642636	24.71021929730876
C	19.18446155529074	2.99077921631775	23.68174719713736
C	18.74204057710328	1.66585617146155	23.58371441837010
H	19.00674024137074	0.93296899496390	24.34083695827550
C	17.94232026188039	1.30573796884351	22.48945804567341
H	17.58439721532172	0.28323459891196	22.39674016892161
C	17.59442036392641	2.25417078463787	21.51885555237344
H	16.96888779274504	1.97745491618048	20.67483454863032
C	18.06511257994025	3.56614807251089	21.66518004437446
C	17.86032759223207	4.71471533701119	20.77628135895089
C	17.19249734349967	4.67357961675437	19.54737682032689
H	16.73952209382171	3.74705852530381	19.20469123836688
C	17.13718178085408	5.82795232398038	18.76159978906682
H	16.63004937362878	5.81343441985056	17.79983498337669
C	17.76532268392616	6.99113627753733	19.22217968569947
H	17.77180062010878	7.90373873546527	18.63201135321000
C	18.41138208364067	6.97062678129456	20.46082036662711
H	18.93010587846426	7.84431450464825	20.84371461978298
C	21.75789352708130	4.65850126744466	21.04406435431656
C	21.64752844526917	3.26422593720378	20.87496583502790
H	20.91701122135006	2.67907932299033	21.42337300886853
C	22.52971084964458	2.66255731346801	19.98184922417766
C	23.52667843923610	3.44030752520738	19.25556644989187
C	23.63157761377031	4.81963955409195	19.42894252435292
H	24.37160389310421	5.41078208666629	18.89885037177427
C	22.74027902555317	5.43863099142161	20.32136571979172
C	21.63075152037952	6.78178863494581	21.53411518007626
C	20.98395802205672	7.88154382353303	22.19803104294494
C	20.98151790549390	9.29032412169481	22.19024397013036
H	21.65527776993786	9.95043191255667	21.66191842411776
C	19.90399372536993	9.64534771444174	23.02553387383580
C	19.33374181172178	10.81263135196714	23.64291190136068
C	18.82835248418895	12.71828034354583	24.42988403323255
C	18.72068293669179	14.08385473279569	24.74235807193354
H	19.33542030682991	14.81235897963700	24.22316239342295
C	17.80930456580591	14.45601610903193	25.72952106796840
C	16.98805512195390	13.46240362983009	26.41102836350405
C	17.07985196573106	12.10789617259075	26.10266926785887
H	16.45606594640043	11.37394592244380	26.60189642244435
C	18.00441120124108	11.73869912395646	25.10594239020946
C	25.32173033598525	3.37278307010741	17.68136408582113

H	24.87246360842477	4.11670695712028	17.01038177219963
H	25.81416992141476	2.58911871045367	17.10337202594569
H	26.03932262098593	3.85643190271458	18.35711719620141
C	21.62878378593039	0.49236066128063	20.37696714359502
H	21.83190054471074	-0.51556760745328	20.01012497038628
H	20.59533346776638	0.78234433066434	20.13481005511650
H	21.78260112811636	0.53742725030853	21.46513147794887
C	15.29449204567111	13.09416568336182	28.04692603366859
H	14.62099604219680	12.57363192786032	27.35042351991039
H	14.71759852816746	13.72319525593986	28.72760895746389
H	15.88163681615331	12.35887531887795	28.61723078380421
C	18.34542863160943	16.77550887441730	25.53200619677211
H	18.13959343475164	16.81355800457928	24.45422835400886
H	19.41939970912699	16.62954496019961	25.70784595161521
H	18.00311123058614	17.69175114194160	26.01580529695666
Co	18.12912128752970	8.59419084470445	24.96255369110792
Co	19.41038338505752	5.63754059163256	22.91014551067059
N	16.43850328741255	8.48492176894747	24.05694501663586
N	17.04796558569724	8.71456648491384	26.46076004542027
N	19.55171556136219	8.81182226061441	26.27674803291408
N	20.27755719006196	4.92882128197863	24.47341588086316
N	18.84436340845065	3.88442361209589	22.72374692052252
N	18.44343974141852	5.86766890995615	21.23424766885451
N	21.06850247610596	5.53442003498019	21.82818501069548
N	22.63217122719506	6.77127858598627	20.65075999031175
N	19.96640167119605	7.44187551754934	23.01840636331572
N	19.29749339205636	8.49288325935368	23.47922981812657
N	18.32907742374677	10.52645395039187	24.57437531785287
N	19.65541361996200	12.10258995856748	23.51698980514576
O	18.15669130893223	6.68926284867638	25.13197259376261
O	17.85971164643992	6.03905777739237	23.94967494981976
O	22.56442653641432	1.33789377317719	19.69791763288702
O	24.29899825025666	2.69385442619253	18.43677761468090
O	16.15663944258525	13.99344984806782	27.34005529680267
O	17.58568763751572	15.71922925138028	26.15196718106174

3-III

C	16.33528932102266	8.57754787588697	22.71058276311976
H	17.25217233043111	8.64899732987683	22.13933880255917
C	15.07670811187054	8.52665947577256	22.10533017276579
H	14.99910334144577	8.55675930879548	21.02187161914223
C	13.93701983580000	8.44574582693659	22.91306833356852
H	12.94423332510389	8.40551762076154	22.47138106174546
C	14.08932211196462	8.42923651038629	24.30391228134778
H	13.22127848244532	8.37508670486778	24.95557035509097
C	15.37594501058298	8.48833808994126	24.84734968548777

C	15.70398076468460	8.50500820278539	26.27677400254868
C	14.83907756685439	8.42114601075865	27.37544530133607
H	13.76578495153290	8.32836966650552	27.23452137895720
C	15.38893228790603	8.44161898093697	28.66505678331660
H	14.73548642235500	8.37052597634437	29.53126256564465
C	16.77543063141392	8.53416480843508	28.84591927775914
H	17.20324841985109	8.53006293525221	29.84455151119043
C	17.59519689712499	8.61562271662860	27.71204238721138
C	19.05778227432624	8.71890643872967	27.64951399561546
C	19.90232810759818	8.85812594330173	28.75612467423023
H	19.48900660536782	8.85185634710525	29.76123749586009
C	21.27505138269377	9.02886718997047	28.55153319449503
H	21.94569614732146	9.14833395117034	29.39916729220599
C	21.76322438649201	9.06935437448266	27.24016965227311
H	22.81799968543428	9.23138645577860	27.03333874804479
C	20.86912592750803	8.91233888366350	26.17736659674921
H	21.20434424368521	8.95592470819607	25.14580371746939
C	21.18497228061022	5.62006850213257	25.21539135278741
H	21.37681942347979	6.64261985701092	24.91584635173795
C	21.79786407214704	5.03591877331093	26.32718448582460
H	22.49413045246875	5.61776320432642	26.92503927584267
C	21.50519680824979	3.70455989240454	26.64292099817329
H	21.96708196253591	3.22218107440374	27.50102070115566
C	20.61543626242242	2.99279461144557	25.83058169564834
H	20.37641381165941	1.95495419421355	26.04709342680594
C	20.03944512553317	3.63356203744014	24.72976161490303
C	19.11223456467928	3.02466024759153	23.77017325030200
C	18.58024839741442	1.72877772832628	23.76695456537458
H	18.83737337316339	1.01884608217861	24.54821562155089
C	17.69334514436376	1.37064279359093	22.74181668814603
H	17.26321332693270	0.37208521849485	22.72515263412968
C	17.34279847269252	2.29498773571461	21.74839527452947
H	16.64145533238454	2.02318350181062	20.96448157618982
C	17.90448355594192	3.57805763737910	21.79882284033771
C	17.69389752136718	4.70354742853790	20.88057985933459
C	16.94324139144146	4.65221297147364	19.70089005901469
H	16.42436184903258	3.73848152854302	19.42341040008811
C	16.88888252822543	5.77983970466531	18.87573020244632
H	16.31855517011342	5.75514103773501	17.95017961154837
C	17.59931926375353	6.92679582449929	19.24897102109095
H	17.60880808557155	7.81556639436129	18.62322755447457
C	18.32163542377842	6.91862344271764	20.44535688160707
H	18.90068429092701	7.77972034943132	20.76346512486412
C	21.77679605362664	4.68351617781096	20.99068542967382
C	21.69782785104255	3.28971091468919	20.81576086949369
H	20.95999820700190	2.68909161619760	21.33680714611589

C	22.62081261905101	2.71324675045632	19.94655362743224
C	23.63467001226256	3.52864363491031	19.24216157777607
C	23.70555341331601	4.91489501524920	19.42088990321374
H	24.44536742849010	5.52439529293780	18.91203630230030
C	22.77902002050817	5.49638015703275	20.28881999058309
C	21.61284192882174	6.79795616425273	21.48237475942500
C	20.96880689696878	7.88606867674845	22.16885951785720
C	21.00878878114427	9.29365002206252	22.20314512134048
H	21.68385113095203	9.95370865515273	21.67517535179930
C	19.96619680872968	9.64255161417110	23.08373569030141
C	19.41332083775416	10.81969114618575	23.69963155556415
C	18.88619281114017	12.75106487301625	24.38586958890870
C	18.76175844363628	14.12237911571989	24.61834776680951
H	19.36995587209414	14.83411534775731	24.06933749972476
C	17.82837689417718	14.52818331019692	25.57903877902182
C	17.00602976701776	13.54391874180974	26.31624967277334
C	17.12558672305956	12.17580416334455	26.08466181515616
H	16.51594692091507	11.45658133857139	26.62111963006474
C	18.06450305504910	11.77644649493354	25.11592911030231
C	25.48170383724367	3.47582429121511	17.70526877665144
H	25.03642048285583	4.20102562044990	17.01471568760509
H	25.97948557518841	2.67550986594381	17.15777825892096
H	26.17656641630136	3.96539074494640	18.39692883531785
C	21.78569114801920	0.49653191791223	20.28347487249815
H	22.05166261594062	-0.48583200114951	19.89175041687747
H	20.75412341029637	0.75368959364774	20.00917199523711
H	21.91577406101420	0.52441919801454	21.37306776757774
C	15.29700153685581	13.27392749119249	27.97007309597860
H	14.62983482951603	12.70836017047202	27.30681010190700
H	14.72373742308243	13.96209833733251	28.59218677660369
H	15.89040105782059	12.59399068345697	28.59549375970241
C	18.30448699366182	16.86523919882699	25.28126703912461
H	18.10451510381004	16.85021764544828	24.20391992561813
H	19.37620972868972	16.76024781438710	25.48486921823870
H	17.91146679494256	17.77478120071795	25.73546415526690
Co	18.15693434047197	8.58606452167413	24.99822916589272
Co	19.39772957412323	5.63657391316866	22.89957847113676
N	16.48262285318306	8.55239583763199	24.04418471846480
N	17.03772616513814	8.62147071735959	26.47961678714473
N	19.54965550492079	8.72043825867865	26.36953555874947
N	20.32485454598035	4.94077609122781	24.44112317835285
N	18.77686587322173	3.89024728018877	22.78462664706312
N	18.35716471758629	5.84327288665379	21.25577630691579
N	21.05011047518409	5.53552566008371	21.75262759426315
N	22.63470185266091	6.81969359039776	20.62758793572304
N	19.95857374263325	7.44536286610507	22.99270443523527

N	19.35205177433717	8.49440586815032	23.52945539134731
N	18.41385494235267	10.55184566828592	24.65482215957285
N	19.72116615451241	12.10205163731801	23.50921732487885
O	18.16439111572279	6.68549335658054	25.13175713176128
O	17.86596696016002	6.05174670972509	23.95141204626294
O	22.70781270967095	1.41143243925206	19.65303438495023
O	24.43095083718753	2.80648315753055	18.45762844383300
O	16.17100633780196	14.11738352383930	27.18913940227533
O	17.57446556744413	15.78644391311595	25.93033980976635

3-III^{TS}

Co	2.21844887779725	1.81908946199027	6.92328165590515
Co	0.59868314622350	4.44002558329334	3.95368978244005
C	3.49346135890341	1.13275809215884	4.35870081099309
H	3.49601639625570	2.20481527611519	4.21196142480848
C	4.00288116476856	0.24388675232826	3.40772080893443
H	4.42008277320236	0.63207895280007	2.48284406975494
C	3.95696379911100	-1.12897900806751	3.66717397751539
H	4.34542668573522	-1.84478677530533	2.94674270696058
C	3.40199782697653	-1.57608019446280	4.87260990450188
H	3.35464985159170	-2.63760894392389	5.10049248695521
C	2.90761948522487	-0.63812142538780	5.78205403654381
C	2.30913067294167	-0.94380126201255	7.08697310759960
C	2.10673147890106	-2.19348686420535	7.68814797809274
H	2.39112513678095	-3.10984256180676	7.17876380078854
C	1.52668547535331	-2.23704648186006	8.96372277230939
H	1.35957437943046	-3.19693420016779	9.44628872811500
C	1.15473569614768	-1.05587944839572	9.62068436547577
H	0.69977126453733	-1.09218347546976	10.60638396029498
C	1.37568781749377	0.16768168307809	8.97497150345247
C	1.07003468326173	1.52315960325522	9.44878521379019
C	0.49317242529419	1.84156116798548	10.68097793077847
H	0.22903196498855	1.05462789498936	11.38240754885792
C	0.26180145270056	3.18524842337880	10.99731314752463
H	-0.18001152979990	3.45338735375949	11.95397877930357
C	0.60968432713675	4.17208487284468	10.06928400616958
H	0.45457312282535	5.22651310813900	10.28203308287603
C	1.17680423955276	3.78969216079007	8.84936743172653
H	1.47164494658753	4.51517603552229	8.09969311806327
C	4.49152237435816	3.35851256521897	7.55965450166597
C	4.89070846126475	1.49275604928880	8.60129350789822
C	4.96408091198000	0.22898298409897	9.17930384550508
H	4.24036080938533	-0.56105782118129	9.00882719216019
C	6.05146058163392	-0.05275813958107	10.02699868554881
C	7.08406512561643	0.96711156159667	10.29496357959350
C	7.00235093544002	2.23112530322150	9.71839053968185

H	7.75100705458247	2.99442239870077	9.90375839193888
C	5.91364624003036	2.50839416448959	8.87586247139757
C	3.70652934025928	4.19492659989379	6.70353331244102
C	3.74842233355076	5.54077172000734	6.32059627250220
H	4.48221904859233	6.28969752471804	6.58579888195419
C	2.62152335622577	5.68782361475353	5.50082150964049
C	2.12006205073781	6.76526692357801	4.69135402399996
C	1.78169389998118	8.61423055868615	3.72135447366012
C	1.77634291034790	9.94185556221819	3.25599186580622
H	2.48012635371929	10.66275790333571	3.65919541041690
C	0.85199078899121	10.28870487797902	2.27677294152826
C	-0.10025556652731	9.29178207504775	1.74138387159161
C	-0.09315238248074	7.96520578964111	2.21152823379788
H	-0.81091020628633	7.26529550460345	1.79362953607304
C	0.83239442760583	7.62603051125255	3.19170296633810
C	3.25101372842997	4.14785941776639	2.62986442643379
H	3.65717778617109	4.39676317276590	3.60585963626114
C	4.08717560312555	3.93825622955824	1.52933158668149
H	5.16446894615472	4.01711449163878	1.65179743010407
C	3.51367607488171	3.64786705732445	0.28608117727518
H	4.13571290790972	3.48151148032451	-0.59021531394255
C	2.11998255115697	3.60515421330584	0.18166746336260
H	1.64371846696248	3.41711644748931	-0.77697893595826
C	1.33965788397406	3.83137824903989	1.32005912124902
C	-0.12418835859385	3.91384905466654	1.33031551805002
C	-1.01280777120566	3.71721745477131	0.26588862473548
H	-0.64937501889653	3.42860137141913	-0.71640995313052
C	-2.38503552159787	3.89833908302945	0.49005521087451
H	-3.09016957558212	3.74500481457125	-0.32315809883971
C	-2.85242098239971	4.27967895568428	1.75499386827830
H	-3.91460935014260	4.42528400486540	1.93107915619771
C	-1.92298436950604	4.46380441448341	2.78705542457145
C	-2.16123625210074	4.89213494826298	4.16831122777661
C	-3.39281406084033	5.29250385892257	4.69579428484479
H	-4.28905869056177	5.25383302880908	4.08221064249359
C	-3.44995346778005	5.75671477466530	6.01352667277607
H	-4.39698449229316	6.07555702923124	6.44203096978571
C	-2.26822575242002	5.81890215818090	6.76155491418783
H	-2.26807298142038	6.19257504968761	7.78210525659158
C	-1.07200058367320	5.39336379743553	6.18020396316917
H	-0.12819385681378	5.41297610569632	6.71188666854924
N	2.95937835036997	0.70614923272622	5.51415151085084
N	1.94496875478287	0.17895644757049	7.74815829477957
N	1.40594999207246	2.49995918306189	8.54835706122087
N	4.00119341678357	2.07588097913123	7.74068068295278
N	5.62830788514248	3.66359003108871	8.21121053216872

N	2.60470899842917	3.57767543721195	6.14177786895457
N	1.93480091608648	4.48987129298255	5.40706352494988
N	1.08132119983898	6.45292832398378	3.84572510475625
N	2.57992039362196	8.03869574598779	4.66071580976600
N	1.90812366921052	4.07269627849209	2.54501276367067
N	-0.60027695884694	4.27077628578472	2.55040250837343
N	-1.01524466854492	4.92473833063021	4.91989227649399
O	0.52576566837036	1.63137519443698	6.10687600405428
O	-0.19622831774587	2.72983907928133	6.10262405878170
O	0.54414719189075	1.69436663983490	3.35323104779140
H	0.52392679971699	1.44744832562418	4.30628385354494
H	-0.34073291339176	1.43299642520033	3.03067935815005
O	0.73378975752980	11.51527046625493	1.73767365989596
O	-1.02150881443953	9.53125765700342	0.80296618663802
O	8.06748868818759	0.56603478827001	11.11822595116820
O	6.03696243515751	-1.29016659017505	10.53394405558205
C	1.62450322820057	12.56704738805059	2.19658075290948
H	1.33607234679317	13.44730773974185	1.62126736550271
H	2.66432806591218	12.29069920438533	1.98671368733855
H	1.47434745770190	12.73868577177974	3.26878733612139
C	-1.27973212919040	10.80939294972804	0.14589928961597
H	-2.10512857322968	10.58253233038654	-0.53050481084309
H	-0.39507341189657	11.13377570958283	-0.40713383744157
H	-1.57069531106662	11.55991218731051	0.88473350587741
C	9.13601655237250	1.49845718797757	11.43958852609134
H	9.79775554432421	0.94384132874617	12.10542355067727
H	8.72116799708514	2.37634436003794	11.94815692000193
H	9.66230903419533	1.78848976442885	10.52296226811072
C	7.04443688771786	-1.87543487426273	11.41549572411282
H	6.67586553644359	-2.88880794250374	11.58128277396228
H	7.09457950315477	-1.31653590129922	12.35285200471956
H	8.01743859618586	-1.88739391750452	10.91870167794054

3a

Co	2.21306704880931	1.81698097703258	6.97824916075897
Co	0.67436401438751	4.34899854085985	3.78165734825645
C	3.35146392066894	0.83472931147412	4.44718924723122
H	3.42199413066475	1.88790051830893	4.20626793131908
C	3.79917970614483	-0.16638420768290	3.57973120019173
H	4.24316903894348	0.10945018528747	2.62709736848361
C	3.66718558202648	-1.50604902488879	3.95821964586073
H	4.00811056472568	-2.30470034657723	3.30380390169716
C	3.09418261429102	-1.81000445636374	5.19905299181255
H	2.98532641192746	-2.84194876440494	5.52191611261607
C	2.66983406257879	-0.76603034291390	6.02445635382513
C	2.07834109344227	-0.91711536759654	7.35922555539088

C	1.75425677637576	-2.09121439005740	8.05050943229645
H	1.92652733819782	-3.06712247478352	7.60560778148044
C	1.19360387306807	-1.97945934370931	9.33124528046835
H	0.93430942683822	-2.87865534058376	9.88478397941659
C	0.95583880037170	-0.72191948139933	9.90187061127595
H	0.51406387883602	-0.63945821485191	10.89068252101713
C	1.29337539856925	0.42155191831419	9.16563128427977
C	1.14905940386547	1.83247447149644	9.54642471760738
C	0.65609008523550	2.28792698974675	10.77237813595081
H	0.32799286318499	1.57742348747149	11.52621850216024
C	0.59775503126740	3.66431217808280	11.01596329312114
H	0.21971233632675	4.03675837895989	11.96508874604206
C	1.04158365099355	4.54867111761823	10.02691352467848
H	1.02322081469700	5.62457427436252	10.17895753479996
C	1.52782216714226	4.03317515662234	8.82138040102045
H	1.89288571968217	4.68332536180917	8.03255791243432
C	4.59084671710985	3.24059858480453	7.36412131428808
C	4.90902996462251	1.42904681971343	8.53437948572840
C	4.92149063488377	0.20866334338875	9.20630141081556
H	4.14858460944918	-0.54659819500986	9.10985088566161
C	6.00691460153163	-0.07531067704053	10.05326930253299
C	7.09842822844237	0.89415729561848	10.22314074979412
C	7.07849333280450	2.11369249368986	9.54924705188330
H	7.87625828289594	2.84089021597588	9.66048019844900
C	5.98816631672188	2.39574083340794	8.71226484762144
C	3.80522760677668	4.07028584675126	6.49956757132492
C	3.84005966517852	5.40968439622480	6.10136602932117
H	4.59371950723830	6.15353944859948	6.32063282384263
C	2.66083598663979	5.57006475482744	5.36235076742303
C	2.11277779880986	6.66252586076954	4.61114733141656
C	1.76849311371542	8.50939694926744	3.64046717011369
C	1.78335120714267	9.82856804697320	3.15591263904773
H	2.55493275301290	10.51927872069124	3.48018981377817
C	0.78798795296173	10.20924747794485	2.26115597908688
C	-0.25814300377308	9.25523804998473	1.83637723698339
C	-0.27352986066947	7.94000754437875	2.33064179004238
H	-1.07398845300274	7.28199162020762	2.00673255839292
C	0.72227602855598	7.56373509234171	3.22880062276275
C	3.38250705033647	4.22496935440046	2.52756501113544
H	3.74334337886070	4.12699120091142	3.54668053177717
C	4.26686572843537	4.28387613935953	1.44652273155090
H	5.33714832647861	4.21969185815774	1.62446972417022
C	3.75179836653470	4.43529773849067	0.15417254854143
H	4.41411020009542	4.48856702062325	-0.70651514111612
C	2.36602194567045	4.52170682237374	-0.01446736666272
H	1.93658160070528	4.64157220664947	-1.00563234515768

C	1.53447808678422	4.45492275138406	1.10813273111523
C	0.06968574160448	4.54476748079685	1.07656110956041
C	-0.76712787323964	4.72019589537922	-0.03343957920236
H	-0.35480620083640	4.79119760837295	-1.03601970385677
C	-2.15085886037836	4.80391338097754	0.17350602155662
H	-2.81653236152034	4.93535402663145	-0.67599375180980
C	-2.68302110400579	4.72755570434823	1.46863084589505
H	-3.75459298239570	4.80560386052458	1.63011626724753
C	-1.80450463608514	4.55726586010928	2.54669055745884
C	-2.11215516189682	4.51675681937849	3.98031417465052
C	-3.38941354377259	4.61626287821663	4.53923710370960
H	-4.26288821856801	4.69556889156810	3.89733305110567
C	-3.52718151089454	4.61652117379824	5.93176436981762
H	-4.51315250453438	4.68942411078239	6.38413915957572
C	-2.37880252141335	4.53013850765800	6.72578735763673
H	-2.44098222961626	4.53362109250154	7.81054596400486
C	-1.12953513688914	4.43220942294588	6.10826442487247
H	-0.20832139397106	4.36124794691854	6.67305527680420
N	2.79986928055426	0.54306137755674	5.63725896600451
N	1.84976508587976	0.28103141145560	7.94308023368667
N	1.57645132236864	2.70881833261418	8.58276518617186
N	4.03953377101288	1.99902353425983	7.64105175275428
N	5.75228239731721	3.52186485803760	7.97040698705966
N	2.65695949289087	3.46567523440453	6.01311716015397
N	1.95179626873257	4.37927283633762	5.31327081071095
N	0.96424913250394	6.39604064191543	3.89461810784837
N	2.62936980289324	7.90681646842651	4.50641772979733
N	2.04587761624413	4.30621428291732	2.37375046295813
N	-0.46855957704486	4.45723419827696	2.31905136140435
N	-0.99125360427248	4.41097851828716	4.76921890239959
O	0.45252441652515	1.78424269777958	6.25666562312440
O	-0.54082939666272	1.52475384097969	7.06878721050159
O	0.67873843197536	2.13926946580600	3.65123260020270
H	0.38997357796818	1.87159477985368	4.56651631103858
H	-0.03245659794962	1.83757370214033	3.05050553194681
O	0.68163298285483	11.43194011125071	1.71331438197864
O	-1.24922764566597	9.52761436497738	0.97915924325301
O	8.07756370561023	0.50544976688466	11.06242215533266
O	5.92650531964406	-1.27220453620793	10.65422088557647
C	1.65668501234944	12.44739573458000	2.07362686243651
H	1.35644877320947	13.33300582815217	1.51281077242164
H	2.65982560172851	12.12146641714317	1.77517692185395
H	1.61173956600862	12.63801498426491	3.15207610259077
C	-1.53072517439136	10.82124799485864	0.36383630283643
H	-2.43346764241699	10.63193601574138	-0.21904126852540
H	-0.70160938627197	11.12049106506058	-0.28178841993232

H	-1.71243838999994	11.57436644000861	1.13441395376256
C	9.19867131456922	1.39949367948211	11.28751960096111
H	9.84284024291058	0.86778351427648	11.98861185059029
H	8.84385026123911	2.33974758624981	11.72583179767784
H	9.72445799262783	1.58310593010019	10.34311555125874
C	6.93099470437413	-1.85479764327354	11.53704930470173
H	6.51942105685547	-2.83683418939461	11.77598725385214
H	7.04111920724500	-1.24691249405768	12.43840554797374
H	7.88659038772663	-1.94739424316288	11.01526286136257

3b

Co	2.16119720968440	1.92617680176420	7.02858981141034
Co	0.81696483964551	4.43720636186329	3.82459433863370
C	3.05870009440280	1.02466814131935	4.38721638272636
H	3.15587957520084	2.08169492970654	4.18941434045883
C	3.35682576310538	0.04631907171940	3.43095060953332
H	3.71476998325626	0.35132606881134	2.45155431151400
C	3.17999425369733	-1.30139473493958	3.75156135447052
H	3.40040413581233	-2.07952723645558	3.02474320614610
C	2.71415707930456	-1.64401675483345	5.02776456975917
H	2.56591619328351	-2.68445135768339	5.30418430005178
C	2.43840766637799	-0.62540368914463	5.94155922837373
C	1.94466825802238	-0.81251269804568	7.30969954969155
C	1.63108666786865	-2.00399395327768	7.97708742538205
H	1.74278218523933	-2.96512355052946	7.48340472828536
C	1.15823831571416	-1.92858342529154	9.29457452804428
H	0.90802247152312	-2.84173421057620	9.82917653915114
C	0.99158867252086	-0.68825266529090	9.92546339576780
H	0.61237313601833	-0.63314922094063	10.94180324858966
C	1.31261413762665	0.47360657565421	9.21200244890022
C	1.20891370643763	1.87151239588243	9.64891171585312
C	0.77196952376025	2.28641293474504	10.91031916217732
H	0.47661341640401	1.55174316686303	11.65450269936783
C	0.71956000589655	3.65377921994922	11.19987022323246
H	0.38262442014865	3.99466369346731	12.17594329873707
C	1.10945823475898	4.57043363885122	10.21766383689941
H	1.08658674713768	5.64133051050097	10.40114233091904
C	1.54369675065800	4.09300862387518	8.97733338362795
H	1.86489433726445	4.77040513304292	8.19241342259956
C	4.55598130512923	3.32724953476315	7.49105626527852
C	4.92083699786262	1.44646266554615	8.51193803652504
C	4.96240716599413	0.17848113668650	9.08250399407144
H	4.20435593206602	-0.58173666763531	8.92489180431149
C	6.06467412505579	-0.14723096268208	9.89445247062433
C	7.14257139015869	0.83459293942214	10.13498416242992
C	7.09095851218487	2.10411952928559	9.56626090360838

H	7.87346464598394	2.83754544492270	9.73128183842854
C	5.98694047601081	2.42487163759325	8.76038241156808
C	3.76836971062388	4.19167568610486	6.66087899610970
C	3.81503318974059	5.54457172843650	6.29409116101067
H	4.56100253469134	6.28271888113350	6.55617210734227
C	2.67241957360311	5.70966685777267	5.49841566216001
C	2.11502876288569	6.77031070864514	4.70803101407799
C	1.62988293400009	8.57710842771795	3.72130898351420
C	1.53813282091465	9.89613484245280	3.24905484038197
H	2.21024214414880	10.65785222761097	3.63048444571892
C	0.57178187766054	10.18396972444851	2.28883956964324
C	-0.33296540353398	9.13014326558884	1.78289524446735
C	-0.23891148240458	7.81097567402757	2.26221367493005
H	-0.93234783140625	7.07454653171821	1.86967830655584
C	0.72447291819864	7.53258466815177	3.22644171347558
C	3.58766977767381	4.33791325181734	2.73645621979550
H	3.90094139143042	4.37818607566098	3.77474748951397
C	4.52142563941906	4.27127452509016	1.69765900474746
H	5.58259265520297	4.25675906766690	1.93175081515890
C	4.06581897010364	4.23279947903956	0.37553170756742
H	4.76759741685269	4.18512980535092	-0.45372045319722
C	2.68857446883646	4.25937086785004	0.13334983289557
H	2.30409455570289	4.23035217568839	-0.88264325748382
C	1.80582994397299	4.32420707001507	1.21617576647634
C	0.34204767600018	4.34948375206805	1.11031570538769
C	-0.45027147334124	4.27973470167082	-0.04350775519879
H	0.00286414672396	4.21148885705102	-1.02842536294227
C	-1.84409578564717	4.28382942883143	0.09962822599480
H	-2.47669760735231	4.22506665240980	-0.78267656054461
C	-2.43012299942294	4.34629310114339	1.37134914170958
H	-3.51054487486951	4.33027613148275	1.48351216102311
C	-1.59170126679861	4.41790671459366	2.49176743271285
C	-1.95788372705063	4.48894848713714	3.90994116673225
C	-3.25874271174186	4.54354830724944	4.41695249834158
H	-4.11020758493973	4.51182891570882	3.74245514911881
C	-3.44677169817036	4.64079584094659	5.80035740638214
H	-4.45157286429611	4.67940544941162	6.21390750836777
C	-2.32714970694088	4.69310381167988	6.63680061429457
H	-2.43116142841930	4.77334095582510	7.71519790570293
C	-1.05116662388986	4.63499387573554	6.07058513676332
H	-0.14743925900999	4.66321756692365	6.66691049608959
N	2.61535175893480	0.69382029897904	5.61097333281984
N	1.79085430286230	0.36786355378855	7.95280744275219
N	1.59549865194211	2.77754206825975	8.69566060213529
N	4.02960963269346	2.05937638419599	7.67526101197555
N	5.72041072996806	3.59338139733964	8.10954325358558

N	2.64839762572804	3.59088292288147	6.11807759354266
N	1.98749946421326	4.51288724261563	5.39653938101296
N	1.05733213541591	6.39051939106072	3.89836751330126
N	2.49187961583520	8.06008725592352	4.64385564923601
N	2.26244955534761	4.36544510591318	2.50612473446804
N	-0.25128795189295	4.44043886548062	2.31885300095820
N	-0.86974281504147	4.52237802632248	4.74367838810608
O	0.36429245083849	1.99892998539293	6.39374951907758
O	-0.57457113677048	1.71310329047738	7.24714923753597
O	0.82209469464303	2.58162023021970	3.73450237174738
H	0.30858578697005	2.27999505564260	4.51587076022505
O	0.37342941935196	11.39610211284370	1.74620292492693
O	-1.28632141796256	9.30781819308002	0.86384696106438
O	8.13329637039321	0.39329391783646	10.92682246976201
O	6.02253495799661	-1.38454362648891	10.39662633746284
C	1.21075263022339	12.50422066674931	2.17801295043832
H	0.85567237287532	13.36068905290232	1.60432268553796
H	2.25941564450245	12.28801731806275	1.94395895295420
H	1.07475692466870	12.67326009345914	3.25229692354208
C	-1.64856735056325	10.56896418044253	0.22103847702427
H	-2.48082620496635	10.29114376530723	-0.42719400537978
H	-0.80566283419223	10.94927660007001	-0.36084754121040
H	-1.96096802684894	11.29757941268862	0.97286000677587
C	9.24512930819025	1.28341067554408	11.22255960167054
H	9.90287210786026	0.70101994515300	11.86827213391978
H	8.87687574312127	2.17371859724393	11.74495823934877
H	9.75669475639296	1.55704789079517	10.29273774337934
C	7.04243642992656	-2.01847964680760	11.22930924091329
H	6.64605446017195	-3.02162317077797	11.39245524474331
H	7.14617082813908	-1.47738665248687	12.17277319469433
H	7.99488697665809	-2.05355463116292	10.69520725771346

3b^{TS}

Co	2.25386343076455	2.00323199331275	6.74696856332934
Co	0.85138463126364	4.51127454832651	4.05568449410988
C	3.52314490898215	1.23959283929732	4.20886310069449
H	3.69687468767465	2.30391397491684	4.12481372334033
C	3.95628560414851	0.33111207791562	3.24092652795578
H	4.49386706789843	0.69096075186092	2.36762531933700
C	3.69012146196068	-1.03165631456429	3.41881549878745
H	4.01441813411630	-1.76326951771632	2.68265537759836
C	3.00434646957041	-1.44336173357221	4.56625189255893
H	2.78852345425034	-2.49489635863919	4.73621856130038
C	2.60395209416967	-0.48252664114597	5.50002805202989
C	1.90656359024716	-0.75285259399887	6.76014155605471
C	1.50127084914005	-1.98555651066333	7.28605111512859

H	1.67366919277757	-2.90678876200632	6.73643226867440
C	0.86695224907366	-2.00948498323368	8.53564847654738
H	0.54005940183737	-2.95621043098962	8.95843783568107
C	0.65363897450115	-0.81913025166952	9.24329596197370
H	0.16607746232417	-0.83497746829727	10.21399623836951
C	1.07849174435445	0.38777342738282	8.67450326629718
C	0.98618198791681	1.73776146407885	9.23642578401219
C	0.50289309031632	2.04331805264800	10.51329823125539
H	0.12512158509189	1.25185800962786	11.15512194624178
C	0.52657599085899	3.36793570307600	10.95674833278623
H	0.15905626266133	3.62308929460410	11.94777508634083
C	1.05211364929271	4.35212141308809	10.11016559701240
H	1.11709540136245	5.39118392766079	10.42290357449200
C	1.51841139215200	3.98305681044994	8.84706880327316
H	1.96196992714990	4.71237989090705	8.17683719382282
C	4.67967555203598	3.40462710646215	7.49682346386384
C	4.98749411224508	1.51418597312975	8.49504134368829
C	4.98958149179326	0.23116728611207	9.03377957667447
H	4.22614439573771	-0.51113135612195	8.82071005672298
C	6.04952824496663	-0.12596107110129	9.88575815235250
O	5.97495122128998	-1.37586474748634	10.35522098510311
C	7.12430687168771	0.84050319037716	10.19994373520744
O	8.07142174824644	0.36876365854683	11.02912044791370
C	7.11485132411778	2.12328422234014	9.66040977859395
H	7.89779038840869	2.84175914339487	9.88022016302712
C	6.05253906923101	2.47535223561184	8.81034883177610
C	3.93835028208238	4.30332173501541	6.65141237309348
C	4.01120597225135	5.66137656989614	6.28849657057880
H	4.76856735934172	6.38275518231741	6.56445227707200
C	2.87384063706834	5.85721517298402	5.48496225185708
C	2.28269238576462	6.87864057645451	4.66594416186539
C	1.72734782482682	8.61856788326849	3.59427556790102
C	1.60270192355103	9.90639311166818	3.05310451240731
H	2.28242586184017	10.69340365588861	3.36316000535055
C	0.59353003798532	10.13310249209042	2.11918243804665
O	0.36256415810899	11.31299166922496	1.52137114366662
C	-0.32026075157000	9.04651472434354	1.71109077944483
O	-1.31263601278676	9.16511879363271	0.82321745412255
C	-0.19219333863601	7.75834233301266	2.25989400603115
H	-0.89068064715230	6.99376870520967	1.93497619501156
C	0.81524723597717	7.54137162254733	3.19596222718032
C	3.44410139714470	4.33394069925871	2.63222502723801
H	3.88046068139521	4.57047408909147	3.59777716314909
C	4.23996977368986	4.14861108042674	1.49711542697827
H	5.32005412548459	4.23711309101270	1.58086989973687
C	3.62377871622876	3.86412367182485	0.27347977695917

H	4.21589806102337	3.71641857550186	-0.62658530473904
C	2.22780397649269	3.79217372072049	0.21876090656553
H	1.72027454334814	3.59416223772190	-0.72161826718320
C	1.48713248539825	3.99509848502911	1.38777004405689
C	0.02139077926841	4.01238341901345	1.46750401658712
C	-0.91271531141125	3.78947219982277	0.44679493108718
H	-0.59040899836161	3.54891207247832	-0.56232512115115
C	-2.27747961818734	3.87726562433115	0.75448488773389
H	-3.01711171403379	3.70622039497426	-0.02387944777047
C	-2.69789442783219	4.17523781254674	2.05843393446401
H	-3.75582001069710	4.23415330424036	2.29839610555259
C	-1.72312548884008	4.38760427019462	3.04217862806975
C	-1.90276017019354	4.70522823458604	4.46429409817444
C	-3.12593812639615	4.90178007198847	5.11206989738776
H	-4.05799974801065	4.80513311114616	4.56157701760016
C	-3.13092685897568	5.23119049304110	6.47221003928951
H	-4.07247279892433	5.38798908184279	6.99291869858509
C	-1.91091035781108	5.36606223259125	7.14420950538632
H	-1.87215557219369	5.63154294080802	8.19710754382312
C	-0.72157623933837	5.15415593174707	6.44218368283339
H	0.25259012082283	5.24591498659222	6.90485905447328
N	2.86122963212198	0.85022848321555	5.31125032727862
N	1.68007622864464	0.39026636598443	7.45784427131860
N	1.47469312381619	2.71054182238663	8.39698203568648
N	4.12914781558309	2.14309539230776	7.64064705618763
N	5.82386802543014	3.65491204708372	8.16787302898486
N	2.81762111729531	3.72843469662910	6.08725185982976
N	2.17845289050100	4.67114605990926	5.39844585793887
N	1.19181882124720	6.44171425850847	3.91044906632411
N	2.63249138235985	8.16365079230180	4.51092117440817
N	2.10252475653409	4.24480959682410	2.58533253589842
N	-0.41165050265590	4.31372718655502	2.71290258896091
N	-0.71560366004844	4.81784062944673	5.14186350431230
O	-0.71649765974870	1.70943027425305	6.49665776327114
O	-1.43260752562331	1.94920033038179	7.48042165199411
O	0.87173684810378	2.66926113480188	4.41240204107748
H	-0.05475232605993	2.35927323996000	4.47327015882466
C	1.20850916183445	12.44835756673443	1.85567446613108
H	0.82417307736142	13.26877813539800	1.24911436597228
H	2.24851160089071	12.22483065724834	1.59195840643385
H	1.11437489469635	12.67795637918943	2.92319134857864
C	-1.69940637529905	10.38241966471633	0.11404233559208
H	-2.55075602685272	10.06290423511003	-0.48866432005563
H	-0.87737436448815	10.72695583643034	-0.51784665646758
H	-1.99102181383372	11.15677734291470	0.82751556005711
C	9.17220613570858	1.24126873518323	11.40192491937080

H	9.79168564946131	0.63903961560540	12.06717640396329
H	8.78509323382717	2.12323528164163	11.92508377460555
H	9.73405522409849	1.53219232421052	10.50687766924409
C	6.94783971129107	-2.03671374023071	11.22020248727819
H	6.53665228291009	-3.04002233279386	11.34123694143554
H	7.01099453796871	-1.51896908071699	12.18027630169065
H	7.92514346448688	-2.06841222822006	10.73262710157352

3c

Co	2.58263067794556	2.34644566887918	6.53185183917401
Co	1.04680597851179	4.60189959633132	4.43919345901857
C	4.68037453552002	2.06981912346611	4.48791970556090
H	4.92603401574419	3.09008762697818	4.76327031679499
C	5.42416086795445	1.35909588623293	3.54163920212032
H	6.27022922012816	1.83749383873652	3.05531463920569
C	5.07365551480269	0.03481193259364	3.25625469577088
H	5.63499611154890	-0.54347887589786	2.52617446239799
C	4.01310507312144	-0.55307263329749	3.95431961514559
H	3.74901147448288	-1.59395922873799	3.78707202148214
C	3.32024246706369	0.20318425135276	4.90508876686877
C	2.29544480670556	-0.31171563450879	5.82106522438740
C	1.72576126844751	-1.59056539755106	5.87259642557269
H	1.99156249177262	-2.35017164100424	5.14280619500588
C	0.80264115108888	-1.87224965124547	6.88991711807737
H	0.34892817280202	-2.85874835953019	6.94658218025887
C	0.45677865689265	-0.89444113906889	7.83286814426258
H	-0.26408839864515	-1.11192229441234	8.61585782657255
C	1.05364188167011	0.36895612319611	7.73351285641895
C	0.86329515070914	1.54629711776478	8.58675871249962
C	0.13452516968562	1.57582783158410	9.77812054147799
H	-0.37416584452557	0.68160684229436	10.12791630003429
C	0.08702347979291	2.76116721018313	10.51925363556020
H	-0.46721422857466	2.80082937260769	11.45386168637836
C	0.78085135349804	3.88239307172596	10.05144331343534
H	0.79390333035622	4.81318117841050	10.61225264040863
C	1.48720568795073	3.79313678099719	8.84922831870129
H	2.06790224565139	4.62253226044453	8.46105790099874
C	4.85263002471452	3.65022939611864	7.66273915593855
C	4.85455728856389	1.62038944497126	8.52181484273577
C	4.68278663237175	0.30093492038091	8.93647045621306
H	3.88941542038125	-0.34538789098292	8.57401204056756
C	5.59145033864881	-0.22502477072297	9.86998746371279
O	5.34187439671541	-1.49973916095636	10.21383178909497
C	6.68916012387516	0.59999394502916	10.38365360351020
O	7.49238583774318	-0.00994610205212	11.27844938150624
C	6.85618173247485	1.91803845081810	9.95268290375605

H	7.66864346211810	2.53810940302102	10.31768402051684
C	5.94215248050609	2.43761662823079	9.02726235433362
C	4.24722791933344	4.64198277840113	6.80068317711442
C	4.36558546762371	5.95796502672240	6.28882530390168
H	5.12710072462262	6.69053143894076	6.51956345228375
C	3.26301404417042	6.10199159805476	5.41018647791558
C	2.58444306856759	7.00094361310644	4.50160410224060
C	1.80473773498187	8.54368828511755	3.26308322245331
C	1.56241094159550	9.73147761095298	2.56107302418916
H	2.22798663785313	10.57962729428090	2.68530668809520
C	0.45560711408339	9.78692634154794	1.71054621600079
O	0.11121359140840	10.87161176448892	0.98765007767770
C	-0.42714581051241	8.62760680123095	1.54827640745422
O	-1.51380202842802	8.57352646752828	0.75973813307095
C	-0.17421502337609	7.43776973260997	2.25058563543112
H	-0.84683754060247	6.59943860838397	2.09801790233220
C	0.92937331334838	7.39777870200009	3.10076414782645
C	3.21210183710942	4.12462904728178	2.50380852079532
H	3.82930547482557	4.62694916037808	3.24111224056136
C	3.72668554553680	3.69040470609500	1.27880142570123
H	4.77783101568900	3.84847657789045	1.05256120952140
C	2.86819190328237	3.07785881630497	0.35911273813936
H	3.23973528101236	2.73152761924219	-0.60242969311554
C	1.51130568627155	2.95156376511523	0.67635518791650
H	0.81257884915311	2.52262370082581	-0.03703111570234
C	1.05286881648972	3.42364014426002	1.91020264698335
C	-0.35376814228331	3.50017220719902	2.32240192651262
C	-1.49215077541472	3.03713970788562	1.64996221814843
H	-1.40522406883091	2.49726441894372	0.71124325586269
C	-2.75118789995273	3.28613757272681	2.21498904242916
H	-3.64716157954203	2.93445187437485	1.70926725146994
C	-2.86626683630260	3.98184929526186	3.42661519461290
H	-3.84178328281164	4.16804221480810	3.86659736038995
C	-1.69555269842075	4.41969929271332	4.05873595572350
C	-1.54975725011819	5.16214504286081	5.31528695542866
C	-2.59621909361889	5.71123365328326	6.06039290578885
H	-3.62476820360177	5.57679809087096	5.73666578404639
C	-2.30022848847646	6.44933338657536	7.21103988122585
H	-3.10091851390113	6.89229297440776	7.79833631392626
C	-0.96153280767851	6.62850849168423	7.57695291492610
H	-0.68766448949092	7.22206737327427	8.44515157087734
C	0.04087184738042	6.04893355799819	6.79522324525016
H	1.09355778579425	6.18947550870369	7.01488656023298
N	3.63670277436977	1.51750582669253	5.13026227523227
N	1.94747572986145	0.61167555912084	6.74711075806201
N	1.51380660159256	2.66136743306298	8.12185650087967

N	4.17098620831401	2.41424088834251	7.64076181121796
N	5.90949860723469	3.69540989015447	8.46625748105653
N	3.13038272898688	4.07722278713087	6.24674486848658
N	2.55997428946904	4.92790713390601	5.42769560309040
N	1.44942973749989	6.41636787039105	3.89998498054019
N	2.82568635282950	8.25734892392528	4.14303460768124
N	1.91627754267692	3.96842197898624	2.82458521455763
N	-0.49373426519397	4.17240173766216	3.48821096154064
N	-0.24284986350460	5.31530289962827	5.70311313254507
O	-1.38016898541270	2.22951576420395	6.45461310740982
O	-1.64458001794039	2.94872349368958	7.45545088828736
O	1.12672816210422	2.75788732509137	5.21646149895835
H	0.26883618963466	2.61279724495957	5.68829517091718
C	0.92711523581842	12.06689114536944	1.08993700048239
H	0.44927631025442	12.78844446809875	0.42636176164028
H	1.94861656973027	11.85227671495693	0.75404302106894
H	0.92557368562722	12.43350941971887	2.12329792891120
C	-1.99112539780165	9.64415567410464	-0.10673673672657
H	-2.86527971530725	9.20663068368261	-0.59217664877554
H	-1.22735390880940	9.90765833299334	-0.84266861914519
H	-2.27150398801410	10.51804557508856	0.48656602193044
C	8.59744305274158	0.73810609318698	11.84796349499130
H	9.07932670602061	0.04320381037941	12.53656821203140
H	8.21718671144277	1.61406809587823	12.38675013000488
H	9.29295090576445	1.03952382418231	11.05563565640766
C	6.13630731906695	-2.30395353847842	11.13429561166281
H	5.63147840970574	-3.27155715442037	11.12593429667992
H	6.11980415757227	-1.86433074429338	12.13467582712949
H	7.16300266160264	-2.39659352713270	10.77148201026801

3d

Co	2.32263253094331	2.23896667678169	6.60463429157316
Co	0.98298736927843	4.48435268260696	4.33674336548524
C	4.61903755067742	1.89846994219522	4.79478004328690
H	4.77840241857219	2.95280211287293	4.99424015827740
C	5.50463728146021	1.14574781679736	4.01939645037165
H	6.37743914378171	1.62507181886757	3.58381879839302
C	5.26072750955462	-0.22142995010390	3.84057707729836
H	5.93660421594331	-0.83286992504926	3.24739246457313
C	4.15193498174823	-0.80227924361125	4.46583236760653
H	3.96182115842552	-1.86874149278613	4.37778143362753
C	3.30754270668918	0.00061871693943	5.23962089756725
C	2.17572995430572	-0.47625726285600	6.04502093833890
C	1.62871293959815	-1.76402349572760	6.11544448259101
H	2.02302555231638	-2.57327172487698	5.50723890820126
C	0.55706998775984	-1.98822849323036	6.99182154783257

H	0.11850903213558	-2.98063089276360	7.06271396827459
C	0.04585242025251	-0.94558566364894	7.77737665304771
H	-0.78459406686664	-1.12234523878402	8.45498719823487
C	0.63102525921654	0.32301943160375	7.67000532231748
C	0.31832974161795	1.54605105225182	8.41818094562880
C	-0.58869234790424	1.63682613903394	9.47818232495364
H	-1.18601268484633	0.77415554253795	9.76136051157710
C	-0.68913764352728	2.83746405768988	10.18975980653957
H	-1.37852717473624	2.92156582248223	11.02648109196173
C	0.13514435078743	3.90970858297568	9.83155127988787
H	0.11971801292350	4.84437455841658	10.38581092373843
C	1.01066062167886	3.76328332341931	8.75186183465613
H	1.69036978712692	4.55344217714845	8.45228657232202
C	4.39581525223236	3.60265488206078	7.99996481824960
C	4.27725285954339	1.59781022123482	8.91629367330738
C	4.05367370641952	0.28705281541219	9.33887745448340
H	3.34367362821589	-0.38434958521555	8.86558636507816
C	4.79189008923219	-0.19493595561792	10.43066912159517
O	4.49568548051580	-1.46375144558746	10.77138092784263
C	5.77384935814522	0.66273980745935	11.09439514329997
O	6.42658272990486	0.09424672648786	12.13068646816168
C	5.99336426904626	1.97191762292054	10.65689214784148
H	6.72749277522758	2.61423919843403	11.13271246442679
C	5.24243432488793	2.45017369703373	9.57560860974619
C	3.91276303705645	4.56121116126988	7.03047674151908
C	4.05247251177806	5.88500309352168	6.54584019962908
H	4.73795164573632	6.64641746877056	6.89236227633795
C	3.09278480027914	5.99227231402091	5.50924905842952
C	2.46551884599085	6.90225321766413	4.57666608558736
C	1.67324919166869	8.48653282635713	3.39753821040326
C	1.41204518411622	9.70598511348075	2.75998175888439
H	2.02485780007957	10.57433762271192	2.97993838782642
C	0.35510756283008	9.76873298276985	1.84763244700203
O	-0.00585024144477	10.88934567504507	1.18658998088419
C	-0.44864885532723	8.58369705275575	1.54855916395173
O	-1.48947330819759	8.53237169723281	0.69518268735569
C	-0.17048350266377	7.36172626267653	2.17948157742601
H	-0.78265655072854	6.50270870254444	1.92222467950900
C	0.88060662967969	7.31453842133178	3.09609200876614
C	3.24359721796790	3.97284119300603	2.52220391958027
H	3.83013377011209	4.38400512732242	3.33662739585848
C	3.81522033015626	3.60964326375995	1.30014530968505
H	4.88619908177064	3.72202053067234	1.15337815446769
C	2.98799495923504	3.12905625879369	0.27761857775289
H	3.40468474229029	2.84491470603477	-0.68577850467855
C	1.60842665208722	3.05058245710736	0.49797300818743

H	0.93887104228543	2.71976393999970	-0.29165510984081
C	1.09367717827771	3.43828246784575	1.73939638384202
C	-0.33067470790476	3.51584460585652	2.08965042968724
C	-1.44331964927542	3.11520335812257	1.33853438837951
H	-1.32264513036573	2.65648719000997	0.36117606135739
C	-2.72190323582198	3.31933345116262	1.87741975069051
H	-3.59924195273492	3.01672094832714	1.31092811924069
C	-2.88005910224277	3.91010866725250	3.13908392409628
H	-3.87191371889787	4.06663318694519	3.55361609420176
C	-1.73390383022266	4.29484057426207	3.84725140564092
C	-1.63961976141133	4.97731130910548	5.14314175262100
C	-2.72183703864060	5.47131474977317	5.87752962110216
H	-3.73839767896193	5.29640738367710	5.53532298040868
C	-2.47723199298747	6.22440615393684	7.03123968781650
H	-3.30644123719892	6.63142540915402	7.60499881739170
C	-1.15383921736848	6.47532501169024	7.40982271448553
H	-0.92026851743831	7.09286528123884	8.27315194390844
C	-0.11398200864322	5.93778672161490	6.64603154378097
H	0.92787486906713	6.12585438849264	6.88325333450709
N	3.53108046087734	1.34923321443278	5.36757129025825
N	1.66812006997121	0.51118139523807	6.82125502476799
N	1.08128237011611	2.62224055110462	8.04373990375531
N	3.75975656762166	2.34612409735351	7.89239402847620
N	5.29025035327290	3.69266407345832	8.97568081112831
N	2.91873923974636	3.95547797007608	6.31021324807401
N	2.45064475267731	4.78709195841484	5.40664618983021
N	1.41946635624579	6.29989643863877	3.84142584126679
N	2.65329774385789	8.19091669741824	4.32410999199437
N	1.92125816458158	3.86299224734627	2.74907256732298
N	-0.51215997366242	4.09170745501159	3.30201350441834
N	-0.34819519941923	5.18478904561528	5.55606033027402
O	0.91814358239620	2.66359063177448	5.21623284400971
H	1.05756586556611	2.01819566537511	4.49165236345978
C	-1.93235559418687	9.62266803445400	-0.16260337578055
H	-2.73863674873628	9.17519592355409	-0.74692799564294
H	-1.11889725226582	9.95373602258662	-0.81330718812597
H	-2.30522754570597	10.45553451029677	0.43876400777747
C	0.73398460312546	12.11100250967078	1.43225961291552
H	0.25689118250852	12.85911314058669	0.79799430396754
H	1.78433196760664	11.97844084074015	1.14586625749796
H	0.65239444782895	12.39416233331621	2.48864606272343
C	7.42128293405961	0.87424634614676	12.83930239687728
H	7.79924616223164	0.20708079595431	13.61495989793744
H	6.95593170355477	1.76021953316762	13.28807460346167
H	8.22818222590801	1.16416574133759	12.15539783512425
C	5.15767830821882	-2.23466928699093	11.81454895558874

H	4.67773698584330	-3.21311328865511	11.75112696044400
H	4.98575086221407	-1.78074441246817	12.79369486301391
H	6.22872110607590	-2.31379144147771	11.61138950650450

3-IV

Co	2.24651356646295	2.25462503460878	6.66730530717446
Co	0.90316405382734	4.50005320588202	4.39982714454694
C	4.56901083335356	1.93006476479989	4.87612194869389
H	4.72834361651631	2.98341780240003	5.08030513328848
C	5.46231456430827	1.18383718679911	4.10239203282855
H	6.34004444271193	1.66660146558891	3.68035489321427
C	5.21885075724561	-0.18107678085301	3.90699819364086
H	5.89924569358882	-0.78755655469205	3.31349737748553
C	4.10138835692424	-0.76607477263424	4.51465950446850
H	3.90874586507230	-1.83059980163007	4.40899364614980
C	3.25184216036768	0.02841836253978	5.29118291535655
C	2.10731277385693	-0.45740131002872	6.07381970609743
C	1.54946381231258	-1.74237645540061	6.10802025619193
H	1.94890015554692	-2.54339517457042	5.49205425462932
C	0.45563559313042	-1.97429862209598	6.95538031168624
H	0.00737168811500	-2.96432480673139	6.99717129394270
C	-0.06765325623587	-0.94093218394829	7.74600827411860
H	-0.91783476213131	-1.12307155610093	8.39741931592171
C	0.52902468890473	0.32553345553257	7.67526710942716
C	0.19797879831437	1.54066020465122	8.42936399968626
C	-0.74034084461200	1.62221859302739	9.46281662429280
H	-1.34883900130763	0.75863434667007	9.71856022218177
C	-0.85839545286922	2.81474309052187	10.18672155085899
H	-1.57059483588860	2.88972228517723	11.00527899567695
C	-0.02235756897169	3.88964572845057	9.86479352463429
H	-0.05113652711972	4.81703646772736	10.43097238818310
C	0.88324853714576	3.75398056458906	8.80802431820835
H	1.56992522241468	4.54890695315224	8.53770115472286
C	4.34822246677903	3.61127604584542	8.03789135098615
C	4.28468671307147	1.59885518712600	8.93394643757820
C	4.08719328690665	0.29302437974064	9.36640034508720
H	3.33684374915659	-0.37450417515463	8.95482190261422
C	4.91321198849950	-0.19300163126309	10.40118584922401
O	4.66180177339287	-1.44924501499213	10.75587948514246
C	5.95508191827050	0.67048447578727	11.00159394834101
O	6.66715736513359	0.09097910006565	11.96920607812602
C	6.14031531549025	1.98354863942675	10.55508591987196
H	6.89901793616517	2.63115202829018	10.98308904522836
C	5.31477095888750	2.45095121350876	9.53005855875112
C	3.83591979877904	4.57775578617568	7.09040326902062
C	3.97622853662134	5.90371066097750	6.61209561710785

H	4.67924256602241	6.65751052158625	6.94203279761358
C	2.98678896206074	6.01994927378635	5.60495943383313
C	2.37872229498175	6.92790258804397	4.65600321493516
C	1.70553302760470	8.50032391605646	3.39827174905324
C	1.52401727425883	9.71332648842257	2.73019576694923
H	2.16801796847218	10.55893208056126	2.95038834994474
C	0.49743863964563	9.79533241710710	1.78295363098753
O	0.20666472935518	10.88541260745169	1.07148333060220
C	-0.36607725778904	8.62660797664168	1.50062448536650
O	-1.36287945128466	8.60527271163993	0.62131926220185
C	-0.17007055509030	7.40769431577104	2.18264824479684
H	-0.82700779430023	6.57769697536974	1.94280177911971
C	0.85342267620691	7.34285621378373	3.12036554773506
C	3.23416434344387	4.06744359992025	2.64412056549141
H	3.79211169625350	4.48327116304874	3.47603751290484
C	3.84915191615311	3.73216523748103	1.43463062567826
H	4.91991329331588	3.87829739228389	1.31766913751385
C	3.06504663763206	3.23471717610817	0.38668493334948
H	3.51566336119147	2.97012366836149	-0.56719571078501
C	1.68255441724031	3.11105466596441	0.57082579426564
H	1.04632233596252	2.76045967355236	-0.23774299950605
C	1.12178223724293	3.47729638934792	1.79856701120277
C	-0.31396025545547	3.50045614610260	2.11010922597628
C	-1.38853316969579	3.04906587233209	1.33217758780025
H	-1.22423211045128	2.59626039658653	0.35827280148084
C	-2.68813942823932	3.18899077471049	1.84179844061589
H	-3.53653246742466	2.84436063644130	1.25505932115607
C	-2.90350439502313	3.76536807379143	3.10205634697104
H	-3.91113857409727	3.86731836279721	3.49544025956072
C	-1.79374904889396	4.20368149675914	3.83784558406976
C	-1.76377636188131	4.87651449591572	5.14228837135757
C	-2.88582788680744	5.30789001074828	5.85650022125623
H	-3.88490256586965	5.08613024351210	5.49039984367151
C	-2.70582081630321	6.05985408812343	7.02362683895944
H	-3.56759105662487	6.41909229471311	7.58146355402739
C	-1.40545747745264	6.37109642025433	7.43650885032530
H	-1.22382716574487	6.99017393888899	8.31136638640744
C	-0.32257397545897	5.89333014861245	6.69187474194719
H	0.70249228998543	6.12757854006796	6.95869946591768
N	3.47643930363957	1.37624063492626	5.43763894724829
N	1.59094292312874	0.51905381370772	6.85831780080973
N	0.97427977938623	2.61991570197462	8.09050508145969
N	3.67919175129137	2.36620664651838	7.97482637631981
N	5.31629433065820	3.69336165801875	8.94024669783889
N	2.82366922370796	3.98061347067048	6.39221490549882
N	2.33007335838022	4.82433933387280	5.51629944050805

N	1.30935637787059	6.34283914875663	3.93711443136853
N	2.63918303361694	8.19398427992702	4.36057641088469
N	1.90820924202050	3.92287162739417	2.83363906279839
N	-0.55145939270912	4.06627035381957	3.31793113521270
N	-0.49397168096170	5.14369885008610	5.58811126730288
O	0.86020980008520	2.68017239141592	5.26780046703408
H	1.00534872700542	2.03639171970921	4.54265273740152
C	-1.80576918891122	9.70950196634833	-0.23920278468993
H	-2.63364227140711	9.27255236918020	-0.79812982119024
H	-0.99211311873780	10.00984942553894	-0.90334268335026
H	-2.14037336435298	10.54716669172488	0.37703968165247
C	0.98687730149658	12.10895969574033	1.26302592369171
H	0.54343198450162	12.82620443062408	0.57260731858488
H	2.03400467383000	11.91502098663668	1.00729796061142
H	0.88200573154740	12.44691697049451	2.29950052607944
C	7.72805720012019	0.84191666220248	12.64219890545571
H	8.13396046875689	0.14394814899808	13.37422386482676
H	7.29550031737517	1.71891812592711	13.13555914045744
H	8.48993057258608	1.12823487546931	11.90930250811788
C	5.35101394705188	-2.22864083763459	11.79210863637624
H	4.84368647016342	-3.19302885970926	11.75681557968072
H	5.22205680799117	-1.74660418884635	12.76385074977438
H	6.40910869798923	-2.32614752850626	11.53856868629279

3-IV^{TS}

Co	2.20491269745745	1.54938759252292	6.42058173738258
Co	0.32464352436934	4.52863597205207	4.19789127609112
C	4.67364754682316	1.90122869946158	4.74078300608303
H	4.42005132870381	2.95671821501179	4.75394036914705
C	5.83500135675022	1.44937454355204	4.10816745933414
H	6.48867720941083	2.16142398531234	3.61078684511296
C	6.13852321891623	0.08327595794588	4.14204232163504
H	7.03521241108382	-0.30135482916209	3.66175077793374
C	5.27287806594588	-0.78133166430230	4.82062075525088
H	5.49096884620574	-1.84455979165200	4.87773243813619
C	4.12834595967327	-0.26466810179282	5.43781295079104
C	3.16963426051253	-1.05420258417326	6.21895141191472
C	3.19489134514719	-2.43108296403697	6.47749266887170
H	3.97885117523121	-3.06141029152664	6.06698718257408
C	2.18559511518614	-2.98562310505193	7.27743009010596
H	2.18561560752690	-4.05288081877830	7.48591676624090
C	1.17544441213016	-2.17113541743667	7.80690662385237
H	0.39059093494912	-2.59757886958196	8.42560117130090
C	1.19387329724449	-0.79977557207593	7.51632109348410
C	0.24933035985706	0.23527646088662	7.94680113024733
C	-0.86949682493621	0.02719344442786	8.75773405958055

H	-1.10240215208551	-0.96807822322357	9.12722141176164
C	-1.68384495237973	1.11782754692045	9.08651595981245
H	-2.55701809732919	0.97787741449869	9.71931859293326
C	-1.35427882448231	2.38181305511586	8.58646062142495
H	-1.96041279455615	3.25412525417083	8.81578170723126
C	-0.22697697526001	2.52267591402994	7.77227551935981
H	0.05619518214925	3.46935448796044	7.33617246559977
C	3.98256099522430	3.30337170211687	7.90713063848996
C	4.22977639839037	1.54987097880780	9.12172315836151
C	4.22793294052044	0.32361008824099	9.77741294744128
H	3.57204058496525	-0.50019753204629	9.51328774406660
C	5.11715591411808	0.15555122249658	10.85552082524241
O	5.06214952055146	-1.02679979043772	11.43672883688597
C	6.03896751322569	1.26715667597140	11.27928475777113
O	6.80551325626096	0.95017002717133	12.31686947932852
C	6.04494890928847	2.48511869711409	10.61591686379284
H	6.70264142922590	3.29734010509107	10.90844260381197
C	5.15401331407227	2.64438863420482	9.53758577703463
C	3.40752050534919	4.08357644343598	6.86562562698886
C	3.63664897541292	5.38248187433069	6.38700257908032
H	4.36346438443181	6.10170049255963	6.74090408891433
C	2.69388102174680	5.53018995491934	5.36576569544157
C	2.27496217593027	6.56231309507193	4.47622708451489
C	2.01353027548667	8.34994665927115	3.37877406636171
C	2.10286097434221	9.63229036783071	2.81448300825871
H	2.89760841301642	10.30711112842471	3.11590393953119
C	1.14542628422251	9.99456760566809	1.87335539704585
O	1.08731948797832	11.16782135695425	1.24983712500926
C	0.05907334465215	9.04539666310560	1.47789581336807
O	-0.88744653748982	9.31303566394375	0.59423717394879
C	-0.02046602442334	7.75963234778019	2.04757628610125
H	-0.82406439717276	7.10609396798156	1.72450874137886
C	0.94338693823924	7.40363152100343	2.98429611691548
C	2.20433547892133	3.27755266225953	2.31372020852146
H	2.91164686013768	3.38238465998860	3.12781129093730
C	2.56838419325047	2.74164168357940	1.07577741478702
H	3.59353561769661	2.42161385071230	0.90796220324283
C	1.60208376040490	2.64252224831581	0.06640576938077
H	1.85688797060024	2.23498517058928	-0.90919004907696
C	0.30179753479895	3.08990880397068	0.32514069140075
H	-0.46438665066130	3.03758567010089	-0.44395119804754
C	-0.00039683519028	3.62114799614098	1.58337037410883
C	-1.30026255166005	4.16208615426903	1.99430220112000
C	-2.50605799986128	4.17438171724582	1.28098049710343
H	-2.56811456392847	3.75142597017016	0.28215332845031
C	-3.63914064172729	4.73082339329673	1.89167311225896

H	-4.58716075632092	4.74553537487337	1.35923148469250
C	-3.56162645087384	5.25316690914126	3.18978389384770
H	-4.44380958012795	5.66780444463583	3.66942843683365
C	-2.32958687981456	5.21658301877169	3.85789243812682
C	-2.01087724090374	5.69312763348393	5.20804558753314
C	-2.90766249437328	6.34098636163525	6.06433993162329
H	-3.93731944820962	6.50240167383314	5.75635991841443
C	-2.46146609143502	6.78658303388983	7.31317751247778
H	-3.14332948173406	7.29648436418288	7.98980105351277
C	-1.12324539561308	6.57818179272831	7.66656211608574
H	-0.72863553922270	6.92286924471721	8.61886077892160
C	-0.27800826088229	5.91960791049676	6.76787181971175
H	0.76714449321222	5.74737576477066	7.00462426497117
N	3.82187535301174	1.07467884090482	5.38504907109787
N	2.17979644002787	-0.28506541542747	6.73738109218929
N	0.56540249473672	1.48071140706501	7.46132794370727
N	3.49281415427257	2.02270119635750	8.08346698475703
N	4.97048824709208	3.72118549909819	8.75036557197961
N	2.37490561659395	3.47540836164018	6.13873891693224
N	1.93885983152998	4.36064507835689	5.25625286606447
N	1.15542977796511	6.26587000900092	3.70218843151042
N	2.81825919854204	7.78598132480370	4.31307202601150
N	0.95156658745534	3.70003370039225	2.56807574290969
N	-1.24849532770632	4.69932613794645	3.23471160853759
N	-0.70833461494075	5.47465627246222	5.57187196446094
O	-0.31836221974734	2.94331353308108	4.90228265414489
H	0.11249895438206	2.22032483925309	4.38878385368379
C	-1.07594060428627	10.54935884379863	-0.18030672033744
H	-1.95940177813479	10.33205867872318	-0.78058130702356
H	-0.19886087486574	10.72578415565669	-0.80697472352200
H	-1.24941503807922	11.38517082372795	0.50112512254523
C	2.09097638831768	12.19524280175878	1.53926788517989
H	1.81389045374344	13.03482088906486	0.90223337676107
H	3.08337581660580	11.81325150447269	1.27738942957627
H	2.03158056791529	12.46846421552390	2.59818687178046
C	7.75154165724936	1.93567603349307	12.84879588994422
H	8.23526170373904	1.42767761800152	13.68269989470588
H	7.19743494507441	2.81609423653357	13.19098114251101
H	8.47687172261338	2.19380860943620	12.06995605901664
C	5.83308756698488	-1.51252211459414	12.59313233123562
H	5.45652478431239	-2.52598075201026	12.73075574874407
H	5.62261895203289	-0.88400957995125	13.46121141603871
H	6.89773005061143	-1.50813291814428	12.34934463363752
O	0.98814428611976	0.57207839586093	4.15272158478000
H	1.42891800216581	0.21338819004299	3.35705381196824
H	0.25514397812450	-0.05029312293908	4.33490277156753

3e

Co	2.33314847205172	1.54509315201273	6.53058765957986
Co	0.25465239761704	4.52515278970814	4.21316518364577
C	4.75473042737968	2.19368012471205	4.93617831350446
H	4.37871240486317	3.21159902122400	4.95233519057621
C	5.96227413202064	1.87291324051453	4.30616820336351
H	6.53629086163537	2.65668238641379	3.81861572842394
C	6.41168857180901	0.54805914940756	4.32814857389353
H	7.34847720894399	0.27004149073839	3.85080942868688
C	5.63818801643185	-0.41929411292673	4.98065020209804
H	5.96341563995728	-1.45579359020125	5.01450956395655
C	4.44200800179090	-0.03645326726594	5.59524933551574
C	3.54024668532845	-0.93699655074196	6.32490117996576
C	3.66607061821244	-2.31619779840754	6.54187131245617
H	4.51011014684502	-2.86982078987788	6.14013551239156
C	2.67548843875389	-2.97211130264539	7.28732689555557
H	2.75373211102496	-4.04287653441577	7.46103500440103
C	1.58533967269858	-2.26211338872241	7.81255753753986
H	0.82292432575644	-2.77271038773444	8.39435985124718
C	1.50526443988603	-0.88349728109723	7.57177622516680
C	0.49734920621408	0.07608576324837	8.03925374074123
C	-0.59586706103551	-0.22514171686601	8.85608061016064
H	-0.77086581172552	-1.24625569163504	9.18463030955350
C	-1.45499614686920	0.80820101123170	9.25101302551697
H	-2.30664223179968	0.59504127469000	9.89282111169878
C	-1.19715974746860	2.11145770164358	8.81342972498596
H	-1.83789430416313	2.93949078555346	9.10459265849405
C	-0.09170517269129	2.35051701264335	7.99010940361873
H	0.15209201840765	3.33519752546143	7.61249929880597
C	3.81951491781306	3.34095603703341	8.04695414854835
C	4.11522124671369	1.44022046409424	9.08626592524836
C	4.17254447790361	0.15523625337371	9.61145705260068
H	3.58198356306051	-0.68129590105076	9.25505865159896
C	5.04195859663348	-0.08308061282043	10.69761797662646
O	5.03060480114882	-1.33981977482373	11.13237130040124
C	5.86681747923389	1.00799572824269	11.26047530620226
O	6.64056540425073	0.64712408569731	12.28708646361288
C	5.80279663930376	2.29460893183274	10.72291177116493
H	6.39533089475849	3.11178841894741	11.12181624918655
C	4.94460562466216	2.51655248554748	9.63955046330102
C	3.23527940170807	4.14206691943191	7.00963462655772
C	3.47771943920317	5.41352149268400	6.47279324154244
H	4.16859976834804	6.16847174254255	6.82417391877289
C	2.61155629092300	5.47519822092726	5.37292332219637
C	2.21503499821524	6.48890901297116	4.43783815351141

C	1.93553070631355	8.23364981708694	3.27222888520522
C	2.03458704847945	9.49183242033571	2.66665298745834
H	2.82704112839347	10.17361437618335	2.95851978354299
C	1.09454745833873	9.82938870834168	1.69182243852802
O	1.06091510829445	10.98988756505193	1.03216432064455
C	0.02900957329125	8.87576587572525	1.31359292756780
O	-0.91455365331863	9.10027840938196	0.40364047926260
C	-0.05483009684725	7.60573155951624	1.92413327654187
H	-0.84845525969857	6.94617686430647	1.59134943887004
C	0.88893485354651	7.27896545553211	2.88999622263663
C	2.00844634534192	3.02774214013079	2.38701732191931
H	2.69661972542421	3.04925684335841	3.22278879382007
C	2.32634787430204	2.42713221667895	1.16417138062023
H	3.30329020904213	1.97090489960067	1.02885334130721
C	1.37905459762570	2.42861777952891	0.13481886831548
H	1.60079264653839	1.96967765621478	-0.82583304860741
C	0.13606312086172	3.03610737438884	0.35390787526371
H	-0.61674361420734	3.05467778554150	-0.42986661935118
C	-0.12121190686535	3.62693901094843	1.59393587864982
C	-1.35467620625882	4.33005601399645	1.96743490671770
C	-2.51022724390549	4.54487472826188	1.20359309344737
H	-2.59502832637481	4.14764190807952	0.19592915070064
C	-3.55806780527472	5.28831356512167	1.76711120053707
H	-4.46368649051337	5.46341435882598	1.19087629369310
C	-3.44981634006883	5.80857162138083	3.06486385719019
H	-4.26270068705042	6.38596838588658	3.49619515369250
C	-2.27598663613837	5.56392657815207	3.79071194277955
C	-1.92674007982985	6.00920683478623	5.14539207706131
C	-2.73375924428416	6.80764376416775	5.96188721329994
H	-3.70851706130864	7.13800618100170	5.61260373537790
C	-2.26759813522369	7.18238856981030	7.22733847442768
H	-2.87777711455141	7.80975689871208	7.87299557060923
C	-1.00496408658854	6.74346200094643	7.64068299823171
H	-0.59941523441324	7.02027011346090	8.61046012448421
C	-0.24600156454565	5.94269316482277	6.78004314847657
H	0.74362903776067	5.59508958736412	7.05814974756082
N	4.01075830424495	1.26532667998888	5.56610928498359
N	2.47194767525398	-0.28035900463005	6.83638310213644
N	0.73368835375831	1.35921450074786	7.61330693967998
N	3.38409923353668	2.02041529035907	8.07699772519028
N	4.73326572261640	3.68483618730011	8.95693557951973
N	2.27191922921530	3.48555799406944	6.27148747407227
N	1.89839351602494	4.29965093778622	5.25738734126430
N	1.11956924442825	6.15629036748618	3.64880132327137
N	2.73753002562217	7.70222781236290	4.24661808861096
N	0.81643446085718	3.61195265212852	2.59597601127436

N	-1.28441487331422	4.83674939696664	3.22343421178451
N	-0.69258464867071	5.58023323673696	5.56274045085800
O	-0.45241908399916	2.90927095601279	4.98253892249945
H	-1.22789863676331	2.60748820836097	4.46520283249254
C	-1.07337019539905	10.30421118788325	-0.42207857008748
H	-1.94423213403280	10.07265543483226	-1.03584507254499
H	-0.18174652710706	10.45081431657856	-1.03588589451406
H	-1.25579769017244	11.16996842962244	0.21843528813932
C	2.06985340776510	12.00987665030352	1.31397177465302
H	1.81648363436048	12.83660547558592	0.65051083072245
H	3.06352407489947	11.61208939225366	1.08111712185385
H	1.99646777933640	12.31427140711617	2.36365870720989
C	7.49826434620044	1.64223590679843	12.92896727670299
H	8.00873609627302	1.09424462605268	13.72079981980502
H	6.87728309084950	2.44289595558286	13.34503850419415
H	8.21514554444463	2.03075645730364	12.19749719362385
C	5.79110358558171	-1.88764754986368	12.26282525630113
H	5.47566250819338	-2.93088603937257	12.29328481642997
H	5.51818982597838	-1.36269031633108	13.18100665706042
H	6.86173109902456	-1.80114363071600	12.06408102450471
O	1.35815372244685	1.29367058172685	4.88602821861690
H	1.01217154199628	0.37799066413252	4.84025139198128
H	0.41858083671885	2.04845664621411	4.90843334858746

3f

Co	2.46693703426628	1.50488931671349	6.30030403554639
Co	0.30088118064540	4.50345561157874	4.18945257244878
C	4.86555899358033	1.99596826049430	4.60579259205699
H	4.51652359914636	3.02297691621603	4.57552913543288
C	6.03653071674000	1.60259738661762	3.94920235504385
H	6.61111105139203	2.33803636540072	3.39236680365973
C	6.44673366804040	0.26781401222110	4.03071264920493
H	7.35454637212283	-0.06656896211811	3.53425147105915
C	5.66891275426488	-0.63635116270114	4.76204304997417
H	5.95897529758960	-1.68095613309201	4.83845519870016
C	4.50807545981476	-0.18059053232584	5.39447140396700
C	3.58903275535855	-1.01735357067893	6.17638131622276
C	3.67684041564233	-2.38747260709983	6.45530286600004
H	4.50671001367420	-2.98026745403262	6.08140171523093
C	2.66647025169558	-2.98215402973240	7.22453430650354
H	2.71581205654936	-4.04516130972381	7.44790828971034
C	1.59210709352801	-2.22144765807258	7.70602410376461
H	0.80992626279291	-2.68311107708012	8.30197468702073
C	1.55095730974927	-0.85319544148163	7.40398924503661
C	0.55766223895651	0.14960684755604	7.80255701916238
C	-0.59232406972714	-0.09469744883320	8.55472020162071

H	-0.82124921815547	-1.10001578166844	8.89812720856878
C	-1.44516736073806	0.97727915171015	8.85314017075691
H	-2.34624631553005	0.80983480881482	9.43828373077773
C	-1.12098587724755	2.25407857520544	8.39154798100111
H	-1.75829054627362	3.10877247850810	8.60016620872189
C	0.04392443751927	2.43804462866905	7.63464854191843
H	0.33771060165312	3.39845000819154	7.24005547553438
C	4.12935759027277	3.26945756719023	7.70900765137983
C	4.32633753654124	1.41929743465362	8.83524866484468
C	4.29904840715565	0.16020356815724	9.42760516268075
H	3.62139267338261	-0.63454552106408	9.13516052438624
C	5.18454259230475	-0.09418675878877	10.49020141549634
O	5.08503152266505	-1.32756956540816	10.99604846325879
C	6.11911275554694	0.94807308754218	10.96030260101168
O	6.90790183769343	0.57399987835531	11.98063072540335
C	6.14475142821220	2.20469881081761	10.36062078942956
H	6.82248901886668	2.98407231006759	10.69346529595065
C	5.26168897903509	2.45127193881701	9.29792688175366
C	3.53440776936858	4.06700087204657	6.67792401820719
C	3.75683193328485	5.33516461593873	6.12157090409118
H	4.49155526755599	6.07419570503679	6.41132612062460
C	2.78985367645657	5.42447816348004	5.10994113087383
C	2.34991243667443	6.44148709449552	4.19703494770392
C	2.04986257193587	8.18020603301059	3.03181903496525
C	2.12088514017114	9.42767487081878	2.39151880117555
H	2.94482650729142	10.10131790900684	2.60304145406008
C	1.11070980302513	9.76276556900022	1.49366922152012
O	1.04788630077900	10.91992531508943	0.81462274586659
C	-0.00327030940351	8.82984894323846	1.22066047562370
O	-1.01656862154614	9.06295577237409	0.38040775567573
C	-0.06651510913688	7.57982193513193	1.86099010254072
H	-0.89684568216803	6.92379606859542	1.62105945183807
C	0.94951199022639	7.24887703164283	2.75257709114924
C	1.83249717638174	2.87981963210110	2.29659181176712
H	2.60540034546710	2.93309390872186	3.05115486244178
C	2.00250795402492	2.18910638943168	1.09265806065877
H	2.94796141424229	1.69370161622425	0.88916295960097
C	0.94889590738750	2.14636688209876	0.17467044492218
H	1.05411335573684	1.61523632216931	-0.76829621191327
C	-0.24882668869986	2.80100407436601	0.48358016587933
H	-1.08456257823938	2.78595516234558	-0.21092077658336
C	-0.35727339119564	3.47838949077677	1.70059125621304
C	-1.53443442019511	4.21888318058742	2.16401919516127
C	-2.77588683858633	4.37519578663216	1.53346481598759
H	-2.97199916412133	3.91891052945397	0.56725657574294
C	-3.76706118093829	5.12423687828287	2.18168616440026

H	-4.73844288660591	5.25406911248392	1.71115782502600
C	-3.52202570057983	5.69407424288827	3.43808670531867
H	-4.29683618351191	6.25900207597247	3.94851221466402
C	-2.26257884418176	5.50787861651447	4.02373149269176
C	-1.78188733694430	5.98526139247925	5.32553043265197
C	-2.52423776440312	6.76318153648096	6.22012962607770
H	-3.54164327090278	7.05629626877551	5.97445089445462
C	-1.94009983685335	7.16059368068871	7.42700069236897
H	-2.50033605773874	7.76833196386857	8.13363055807727
C	-0.62506859896807	6.77039948813362	7.70323477905675
H	-0.12866938005474	7.06513525132829	8.62402143733371
C	0.06345028269877	5.99269138977639	6.76700941395110
H	1.08760805986884	5.67724807604962	6.93992923643129
N	4.12066941784499	1.13034224301290	5.31549891733215
N	2.54212246103541	-0.30766563303100	6.65749948804154
N	0.86243213725744	1.41040303216834	7.35665519506179
N	3.60206180805553	1.99005447083727	7.82550408358439
N	5.11297668214516	3.59265315847880	8.56468461907692
N	2.49750958874426	3.43678194149726	6.01788181450722
N	2.03324676544112	4.26754950931326	5.07153514002379
N	1.18751734297835	6.13676397094576	3.50847926370361
N	2.90547587953872	7.64155176353542	3.94800233607261
N	0.68377206893158	3.51058497423282	2.59058439825045
N	-1.31875886615737	4.80577655202724	3.36218969064510
N	-0.49785298959538	5.60313792427473	5.60759530643536
O	-0.46837795719576	3.03069963371279	5.00129071690910
O	1.52525497168110	1.24191046452216	4.69791070481462
H	0.21418532871614	2.31487202771324	4.83839805053808
C	2.10105047385763	11.90410245426714	1.00671517199676
C	-1.19808495663308	10.24962550359269	-0.45161083252038
C	7.85888090684210	1.53353560263790	12.52018195188086
C	5.82576062157262	-1.85756448228575	12.13867420280734
H	2.12088440474327	12.22532786782876	2.05445429471423
H	1.82851803864879	12.73374589335760	0.35384768895253
H	3.06530939467346	11.47656597371100	0.70895004250402
H	-2.11591255452476	10.03433043796652	-1.00065575112512
H	-0.35072205241268	10.36546467049133	-1.13152106690832
H	-1.31402273621827	11.13547231576939	0.17747550087771
H	8.57331583718038	1.82363702080801	11.74127419900539
H	8.36288943092160	1.00132344052646	13.32734948181647
H	7.32235046080838	2.40744380662198	12.90678937604939
H	5.42501493195187	-2.86577576530207	12.25275671898315
H	6.89486096451791	-1.88180318672019	11.91488702667941
H	5.62917088195317	-1.25329785446381	13.02744254293042
H	1.07458501170822	0.37280888003811	4.74518535734816

3g

Co	2.44832847688589	1.49064553526419	6.33688970027072
Co	0.29498822459282	4.50486681971482	4.16718051425627
C	4.82164266270908	1.96961842830150	4.59639640020497
H	4.50081802157311	3.00618249238059	4.60241679698388
C	5.96688531087843	1.56652935772864	3.90097051336606
H	6.54747104952629	2.30328281131385	3.35180653844606
C	6.34604119835364	0.22016940684627	3.93559011437399
H	7.23281363421946	-0.12235721952495	3.40742588828713
C	5.56525135049869	-0.68402984381322	4.66460621282951
H	5.83359497759333	-1.73633865006281	4.70700615359199
C	4.43262952783951	-0.21849957902905	5.34020675311527
C	3.52394015281127	-1.05165118844028	6.13669975999577
C	3.59105485073860	-2.43053370431099	6.37983322706063
H	4.39015891302227	-3.03276456441326	5.95679782328842
C	2.59932258492404	-3.02278019171738	7.17486592240418
H	2.63166012736071	-4.09249620267753	7.36821269349747
C	1.56279651494986	-2.24991017209742	7.71826939639753
H	0.79345990548962	-2.71072145969291	8.33159939721478
C	1.54019679813073	-0.87442193487290	7.44944886824984
C	0.58080175713257	0.13546885912448	7.90972144825098
C	-0.52666337942266	-0.10679413776242	8.72696086904660
H	-0.74395756945971	-1.11417571131660	9.07215381113530
C	-1.35075789324526	0.96525630387625	9.09126490059352
H	-2.21675313467253	0.79715932063190	9.72709767288228
C	-1.04197793888713	2.24738019125104	8.62624464597599
H	-1.65653384466854	3.10449009756034	8.88745109761721
C	0.07776170912217	2.42702384238058	7.80742154401523
H	0.35659934160436	3.39417452498651	7.41084221936274
C	4.11409034070082	3.25941042416625	7.72243942627329
C	4.34522820907971	1.40725744936483	8.84656000112648
C	4.34127847697538	0.14850653065231	9.43750433259152
H	3.68609712193628	-0.66309565335500	9.13953727617525
C	5.23208644624303	-0.09084035591517	10.50974323367599
O	5.15337397403826	-1.31819982741989	11.01091371358257
C	6.14127263206642	0.97127735579080	10.98489262059995
O	6.92598507600187	0.62028922502832	12.00384244005424
C	6.13965613092675	2.23315712777304	10.37714538330012
H	6.79913090727312	3.02773484364936	10.71198953497857
C	5.25610086619748	2.45459415849897	9.31379568776068
C	3.50775439666899	4.05863837144082	6.69506453667088
C	3.73779082248932	5.32339896895751	6.13532879054218
H	4.47703011572944	6.05962955621298	6.42236356379986
C	2.77585209008657	5.40909840018997	5.11881551356965
C	2.33860262123877	6.42483744095833	4.20466211055468
C	2.03711473497060	8.16981365683781	3.03883021024832

C	2.13314523818165	9.41404900272958	2.40535973924556
H	2.97000520474338	10.07200361725217	2.61813357914664
C	1.12656143971549	9.77490349942219	1.49777705482764
O	1.08757934739696	10.92427799869268	0.82538711099002
C	0.00245843905895	8.85992998009197	1.22543929120914
O	-1.00099042021187	9.09747382134425	0.39093715109782
C	-0.07922451466752	7.60206086001286	1.87389062556469
H	-0.92255442053175	6.96431576612949	1.63092397354754
C	0.92819463237481	7.25683635450590	2.76612414825131
C	1.87821141828434	2.91728608185357	2.26289358776082
H	2.63423195746850	2.95520830851384	3.03617615614129
C	2.07366136827330	2.24867002029956	1.04971632705977
H	3.02389008846965	1.75852744906338	0.85600393460442
C	1.03883358625375	2.22199943205693	0.10941027631330
H	1.16424475627683	1.70807544814510	-0.84072151717318
C	-0.16830194328429	2.86744615446988	0.40556314401234
H	-0.98966550423130	2.85865199406810	-0.30603140196638
C	-0.30490286591510	3.52417113016271	1.63113736326084
C	-1.49956667201197	4.24248625290986	2.08745805839581
C	-2.73492822933799	4.38597849683694	1.44068537950257
H	-2.90937520339683	3.94058211848142	0.46516358918723
C	-3.75110828395147	5.10416088837674	2.08671471474175
H	-4.71905211264357	5.21910126220790	1.60447743252824
C	-3.53680119940772	5.66140966722682	3.35518199904057
H	-4.33179871115960	6.20193417050236	3.86114968355519
C	-2.28300968650615	5.49166391313601	3.95877832438476
C	-1.83082489105173	5.96525074289633	5.27221040046607
C	-2.59223553145398	6.73729273480874	6.15582339847463
H	-3.60814171157488	7.02279687363356	5.89581800635194
C	-2.02906063814650	7.14110098006307	7.37154946730825
H	-2.60517414224056	7.74536592692060	8.06853099824636
C	-0.71468710130337	6.76286186362359	7.66946966369987
H	-0.23514045506193	7.06534841106395	8.59683878868022
C	-0.00475857725748	5.98753243437719	6.74667309600704
H	1.01971352080649	5.68132643791435	6.93441146960775
N	4.07204743495945	1.10306500155541	5.30175928117331
N	2.51253674160971	-0.33067662117150	6.67612796247729
N	0.86998904350191	1.39879664923364	7.45950360560701
N	3.61282264454302	1.96721239855239	7.83059619468513
N	5.08503756781877	3.59699523608224	8.58089115553062
N	2.47155736531633	3.42772848629688	6.03859245388788
N	2.01953338832382	4.25449414768927	5.07744333282929
N	1.16018277520215	6.13527292647607	3.52432091295165
N	2.89245366176241	7.61864430054529	3.95387384833650
N	0.72227521436378	3.54244956334379	2.53917369844541
N	-1.31821961282492	4.81427170497467	3.29967377285733

N	-0.54822913855497	5.59420255475230	5.57948767547424
O	-0.45270242984794	3.03423953740205	4.90154859207750
O	1.45292682432485	1.26497022153421	4.76201880398390
H	0.28631962780091	2.32154451022876	4.89043851088824
C	2.15161622615491	11.91541858103135	1.00459943705457
C	-1.19217336319991	10.29018216150862	-0.44924197093226
C	7.86656362004791	1.58995222550922	12.56846375424109
C	5.91636414558736	-1.86148374983373	12.14394913460797
H	2.17025802525116	12.24181120156171	2.04983777276445
H	1.87204313025495	12.73650940807391	0.34486484575351
H	3.10856408354946	11.47778345063162	0.70121581002136
H	-2.11432468466850	10.06960787187776	-0.98692334585805
H	-0.34659614397409	10.39602323176145	-1.13229117533331
H	-1.30092622956807	11.17166326919760	0.18647853842740
H	8.58559892935622	1.88820770178418	11.79802470765347
H	8.36199071276698	1.05087605333194	13.37572180581107
H	7.31070339560604	2.45052988436310	12.95547944150015
H	5.53533836967153	-2.87878117467972	12.23515826874234
H	6.98243670653746	-1.85452754929930	11.90630421110752
H	5.70793931039277	-1.27586841432042	13.04208904826777
H	1.01564844909473	0.38769544097043	4.77863167220175

3h

Co	2.43802499858249	1.58018460145902	6.36393149078882
Co	0.34841043782630	4.47579399605802	4.25823521080319
C	4.84376925271674	2.07659267454892	4.67445968824523
H	4.56006898172254	3.12212759294204	4.74087606484232
C	5.97362932021921	1.67330651564254	3.95605184346336
H	6.58042189094026	2.41861302227924	3.44865746369907
C	6.30243267362178	0.31389022723496	3.91415019807465
H	7.17624131713141	-0.03034100564344	3.36606209389260
C	5.49093297477685	-0.60011866243670	4.59362377300800
H	5.72231098230643	-1.66182576512960	4.58097876548431
C	4.37385120361201	-0.13277494147638	5.29371675153710
C	3.43889951007661	-0.97484189355608	6.04936777768822
C	3.45110738743834	-2.36735332488174	6.20413706265509
H	4.22478083452243	-2.97313182980273	5.74111754683685
C	2.43442804782143	-2.96691397677425	6.95980827395660
H	2.42322547386615	-4.04654995993062	7.08778842949223
C	1.42385836879837	-2.18810428926132	7.53923483793703
H	0.62595675763062	-2.65322639141757	8.11116156738313
C	1.46005826138837	-0.79945503316916	7.35510094061273
C	0.52549851602254	0.21655354890761	7.84755514669166
C	-0.59971886347523	-0.02564882208683	8.63909841503026
H	-0.85320375286091	-1.04017533414027	8.93464502037627
C	-1.39061861126828	1.05642292913134	9.04290643130204

H	-2.26931435713454	0.88962943223681	9.66129930426792
C	-1.03140281709708	2.34720788351508	8.64421838657916
H	-1.61683051465828	3.21276102334596	8.94234319258631
C	0.10083603642873	2.52556535072944	7.84318723309851
H	0.42011692052232	3.49594503160456	7.49253817106613
C	4.12841906315418	3.31672234531466	7.80856252184955
C	4.36007366371275	1.43032384490432	8.85959617656174
C	4.35441496712219	0.14636553477294	9.39599712674029
H	3.68304306402919	-0.64216510988657	9.07236401124079
C	5.25885693619928	-0.14218235940739	10.43358808978253
O	5.18332234167156	-1.39676657262029	10.88752264143687
C	6.18836542731064	0.89199499151953	10.93485401039868
O	6.99683305147768	0.48349325900940	11.92611597580950
C	6.19131869092642	2.17384010534143	10.39046369329298
H	6.86636785012026	2.94526725824774	10.74643328966080
C	5.28982309860303	2.45425264619269	9.35196615295426
C	3.52155926183993	4.14080149742137	6.80173525309342
C	3.73136791870478	5.42035490017537	6.26183024529731
H	4.45787667671042	6.16271454205305	6.56377998276849
C	2.77535570611025	5.50829505996126	5.23746058928950
C	2.32509477678809	6.50233601202211	4.30390898462593
C	1.99705975005521	8.20887204870709	3.09742407292634
C	2.04500797597079	9.44712634319947	2.43868059385456
H	2.83338676626443	10.15590005224886	2.66997463014227
C	1.06050200833664	9.72677639197013	1.49369510461348
O	0.98115854122405	10.86846098175225	0.79152163309836
C	-0.00322500008758	8.74592151794016	1.19285090849028
O	-0.98921722081192	8.92365776842210	0.30827155451295
C	-0.04312735380601	7.50524619760392	1.85337198125112
H	-0.83451834031519	6.81194809657091	1.58796225677441
C	0.94699301771662	7.22988306341642	2.79202900734139
C	1.96404436719431	2.82716426401061	2.47647993027819
H	2.70866110184041	2.93078921436072	3.25212630130668
C	2.18111744513546	2.07642030729910	1.31557660771267
H	3.13706703986830	1.58025047542254	1.17246472606682
C	1.15979476029023	1.97151395769716	0.36777494347648
H	1.30091989945077	1.38895305791705	-0.53942253221284
C	-0.05543751678307	2.62759050646529	0.59929751742371
H	-0.86940687001328	2.56108501623329	-0.11749305276739
C	-0.21017008915005	3.36871101337593	1.77288750468647
C	-1.41129843274662	4.11174373404040	2.16429385030256
C	-2.61878605793133	4.26021846645573	1.46820769604201
H	-2.76732966859823	3.78262926970312	0.50392699164561
C	-3.63431887909061	5.03449797767626	2.04484509137478
H	-4.57934737083307	5.15884100409948	1.52170033072759
C	-3.44697129737379	5.64310892568535	3.29358909029950

H	-4.23893094660137	6.23489256587463	3.74350973036516
C	-2.22172129344889	5.46295180313561	3.94861322140127
C	-1.79922139384882	5.98662403262388	5.25380309701557
C	-2.57545302195130	6.80585601690933	6.08029765806430
H	-3.57685423647809	7.09733278237190	5.77490959672174
C	-2.04557775406189	7.24617002675707	7.29738262219655
H	-2.63299822467836	7.88593108045096	7.95169438455377
C	-0.74965561486043	6.85615391117293	7.65339736348758
H	-0.29538193885004	7.18348260412738	8.58487810772096
C	-0.02452520242491	6.03550941442528	6.78372762994792
H	0.98855520385522	5.72123158581664	7.01429549721657
N	4.05958370399037	1.19894747139621	5.32623754139454
N	2.46694550768910	-0.24835811496775	6.64128084118202
N	0.86064782327968	1.48683409198031	7.45796592783877
N	3.61743702000750	2.02735950111254	7.88081922492095
N	5.11805556911050	3.62123258170795	8.66520901136958
N	2.49814775693746	3.50758855926090	6.12528204405726
N	2.04401655934937	4.33868649993836	5.18277786423812
N	1.19795071195963	6.14051895218204	3.57637600513063
N	2.83921025222490	7.71823836199574	4.05333778877688
N	0.80259662889957	3.46323986152829	2.68915224434011
N	-1.25647975999066	4.72388204583377	3.35989916414512
N	-0.53467006556206	5.60592457913420	5.61516589554960
O	-0.27715016429537	3.01886730189921	5.05796009087118
O	1.49596435489235	1.29827136631587	4.79362760834177
C	1.98617441078172	11.89776846763465	1.00744012921874
C	-1.17967258447256	10.08534282774453	-0.55715044443937
C	7.94780684470935	1.42880458290363	12.49005798284856
C	5.96114725907682	-1.97192894724544	11.98240751503224
H	1.95093759380051	12.23614409627453	2.04927646615492
H	1.70694117766175	12.70482508170475	0.32963623610028
H	2.97755747775090	11.50455002531547	0.75522385711562
H	-2.06522138777069	9.82392293406507	-1.13815177393808
H	-0.30963174298123	10.21836131336987	-1.20450356842428
H	-1.35408705251551	10.97906461002879	0.04659588748582
H	8.64504471549364	1.75997350858032	11.71196720585362
H	8.47152086077988	0.86752567525789	13.26429503712364
H	7.40988901691811	2.27943535179980	12.92377166700702
H	5.57986275716561	-2.99157467081993	12.05338892647772
H	7.02451911692232	-1.96560280786071	11.73145343476797
H	5.77745140105282	-1.41787674662404	12.90600347723159
H	0.56168329534273	1.64310498917053	4.91949921695628

3-IV

Co	2.32958523932549	2.30908798315504	6.50936952122763
Co	1.02665539487479	4.42465300731770	4.41184497315126

C	4.58696123957125	1.90794790960643	4.66455534292579
H	4.87910963272363	2.90567346839422	4.97607982403746
C	5.35821130295184	1.15979075584545	3.77076837416128
H	6.27533192095087	1.58248858122044	3.36887465546488
C	4.93800224275908	-0.12919320781058	3.42422353092921
H	5.51912502587218	-0.73633235047569	2.73409952459614
C	3.77353519585939	-0.64238218833689	4.00525320019389
H	3.44419251713493	-1.65466266952818	3.78568616838648
C	3.05310400787963	0.15009449063864	4.90469457550345
C	1.87749423104639	-0.28203429674660	5.66994251798027
C	1.14996853679890	-1.47435572724196	5.56091520376823
H	1.42958407344420	-2.23517359757216	4.83747063138030
C	0.04231700883884	-1.66105328239312	6.39974262136662
H	-0.53463349290932	-2.58032994228606	6.33393353985424
C	-0.33831842143756	-0.66517528578812	7.30993748515527
H	-1.21190332766060	-0.79899772349516	7.94177604832867
C	0.42623382108218	0.50706070190717	7.37592632764902
C	0.22773385678897	1.68625174771174	8.22675598961995
C	-0.70280699351457	1.79217401948976	9.26537297514283
H	-1.39253323575532	0.97643037112730	9.46622888173902
C	-0.70965946916424	2.94267555249078	10.06119084717151
H	-1.41751878517675	3.03694240669696	10.88132157743413
C	0.22709930025569	3.95040976974549	9.80641371666191
H	0.27729330624109	4.84473588135438	10.42188409084633
C	1.12352647911144	3.79098946340307	8.74606125501036
H	1.87962303292300	4.53491346321899	8.51693018498765
C	4.60206090419830	3.57614881045151	7.83517028065500
C	4.53984759507507	1.58815494462245	8.73165181245647
C	4.32786794417062	0.28298342076334	9.17094523115494
H	3.53184824611767	-0.35123565106210	8.79168205877219
C	5.19175271993159	-0.23311354898495	10.14986632366349
O	4.92221578321775	-1.49056961891303	10.51516566750588
C	6.29038977788701	0.59472241292590	10.69344759687647
O	7.04004825735676	-0.01338720596772	11.62507746822115
C	6.49694185467445	1.90096036847349	10.24649007308211
H	7.30000786688762	2.51789236843683	10.63633517158873
C	5.63121646662586	2.40594041663859	9.26886140866884
C	4.07067072450597	4.55716217637949	6.91337860548945
C	4.22949984868864	5.86982061041178	6.39722592791382
H	4.97592624226507	6.60291730354460	6.67239437742099
C	3.19045852723667	6.00595784415613	5.44006270311748
C	2.56691300185653	6.90050602832643	4.48763505242621
C	1.91517108838439	8.44955806669300	3.19830886515453
C	1.73657957994256	9.63525171317722	2.47539975176147
H	2.40878418066753	10.47374246770857	2.62574886300758
C	0.67698042824678	9.69664220968558	1.56812924191252

O	0.39078696537281	10.76582961684621	0.81000643618669
C	-0.23138190561532	8.54515266993417	1.37564363436176
O	-1.26955600099528	8.51516697949597	0.53368011820073
C	-0.04362111619012	7.36054063180032	2.10450963644197
H	-0.72804807252571	6.53342866437494	1.93999657504741
C	1.01644421162396	7.30769176908540	3.00707754885080
C	3.25263625706177	3.93902644579490	2.54922075047234
H	3.83504578369048	4.50478009830603	3.26921623420382
C	3.81471529957655	3.44848718138415	1.36723800130197
H	4.86550980675257	3.62958696885751	1.15694455895759
C	3.00339904939814	2.74528671565538	0.46982261198804
H	3.41141477046343	2.35513804180145	-0.45963082920515
C	1.64712579973023	2.57768518527887	0.76886705795674
H	0.98484996670540	2.06962551897657	0.07265514198341
C	1.14160149084059	3.10239344663959	1.96256899394121
C	-0.26840374805686	3.09249295283979	2.36932507737831
C	-1.35244885442608	2.44884831034337	1.75828669135589
H	-1.21671323031308	1.86016842077922	0.85520809504106
C	-2.61951288669417	2.57040411023175	2.34544356440304
H	-3.47548125445336	2.08163679166821	1.88638073711313
C	-2.78924797768279	3.30116554076408	3.52954587035850
H	-3.76657046068981	3.37266157590546	3.99872978595621
C	-1.66962125238301	3.92335593512871	4.09721209029344
C	-1.58707898107136	4.72074543753367	5.32647430631114
C	-2.67644141777429	5.14555919864823	6.09388248626615
H	-3.68495200835442	4.84410348537874	5.82295135535041
C	-2.45369178298773	5.98955347463461	7.18696694150914
H	-3.28994835150348	6.34196555462851	7.78615905650380
C	-1.14628145232512	6.39740235590918	7.47434978367405
H	-0.93419125199971	7.08014229957515	8.29299685863792
C	-0.09805274774663	5.92683391606227	6.67875998431165
H	0.93078051990097	6.22606382780002	6.85118841360167
N	3.45261803852444	1.42660500281832	5.20231941338102
N	1.51890377297487	0.63929941037448	6.59161959656581
N	1.11142091563041	2.69932037207805	7.96165742013244
N	3.89084197739210	2.35516423616768	7.81368631837317
N	5.63381255703439	3.64354690471029	8.68033656012232
N	2.99769679809160	3.99197790227855	6.28072599575127
N	2.49151091757282	4.83030882017459	5.42066231427850
N	1.44946947504097	6.32483262002832	3.84178112276248
N	2.87128365644138	8.15193827192452	4.13460211338792
N	1.95623309696398	3.75304293059299	2.85169132744251
N	-0.46943194373455	3.83339389007890	3.48177494747993
N	-0.30738514149291	5.09346143146660	5.64466605683250
O	0.99807959036725	2.69909561483199	5.21002661907918
C	1.22447284840448	11.95385215671254	0.91878233894941

C	-1.70503273352025	9.59416076477979	-0.34832756489739
C	8.14485955939107	0.71573612798982	12.23038851183613
C	5.64969280960168	-2.28267085760178	11.50283216479528
H	1.17300863594533	12.34816208968323	1.93996133803968
H	0.79236773956991	12.66072866928016	0.21005626268700
H	2.25612161596653	11.70828156157235	0.64277042342797
H	-2.57166436801027	9.16894043577250	-0.85663141414625
H	-0.91428729118800	9.84150645237388	-1.06077913695700
H	-1.98601364212416	10.47159864941086	0.23929110881802
H	8.87473802158992	0.98683395243846	11.45923929374364
H	8.57946892077267	0.01623055737983	12.94485929593688
H	7.76260043404811	1.60635575179313	12.74171951383324
H	5.12091983357452	-3.23689901245046	11.49863910719470
H	6.69006289831201	-2.40879089721318	11.19334648174031
H	5.59194232117871	-1.80612699982529	12.48446009813090

17. Compound Spectra

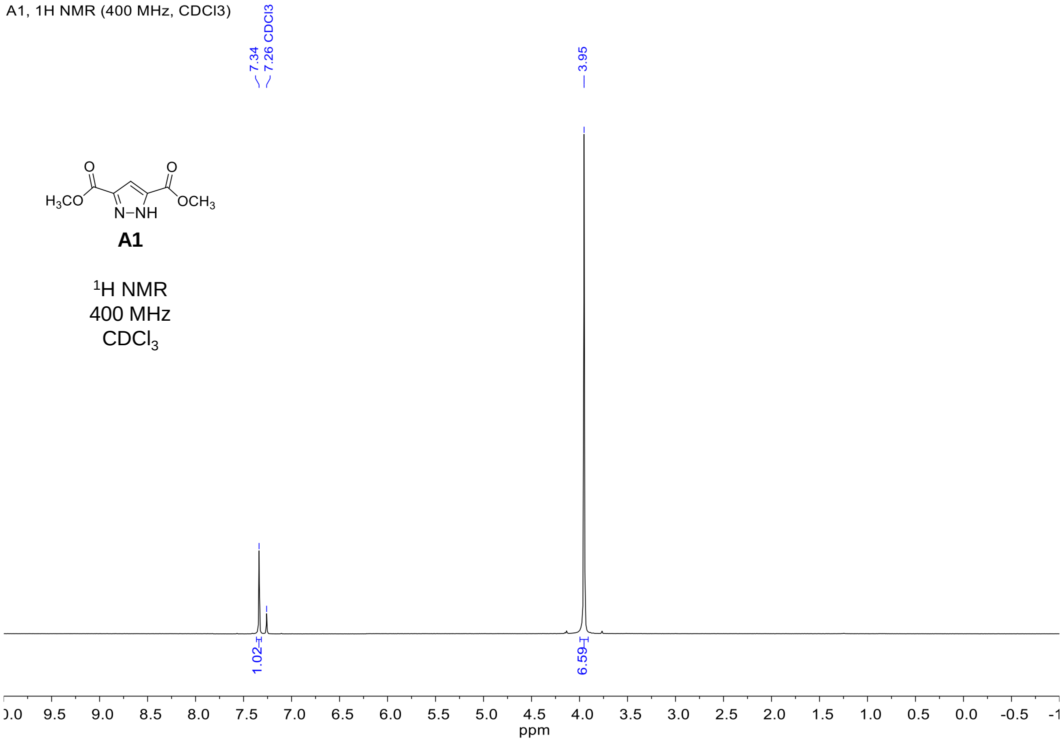

Figure S37. ^{1}H NMR spectrum of **A1**.

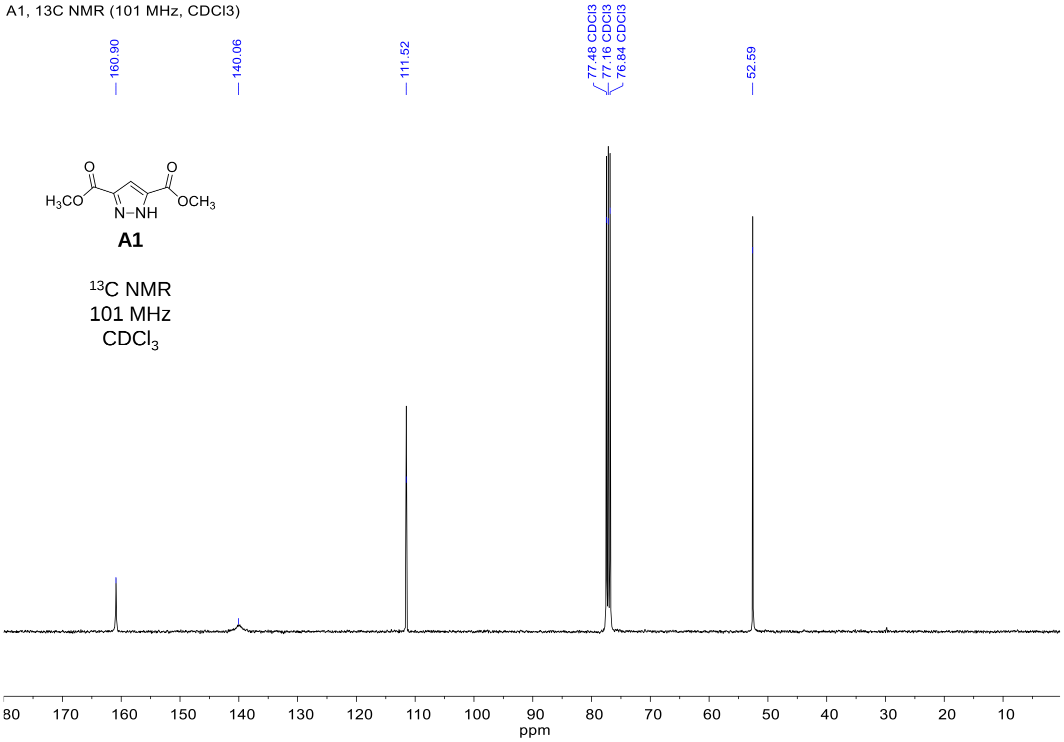

Figure S38. $^{13}C\{^{1}H\}$ NMR spectrum of **A1**.

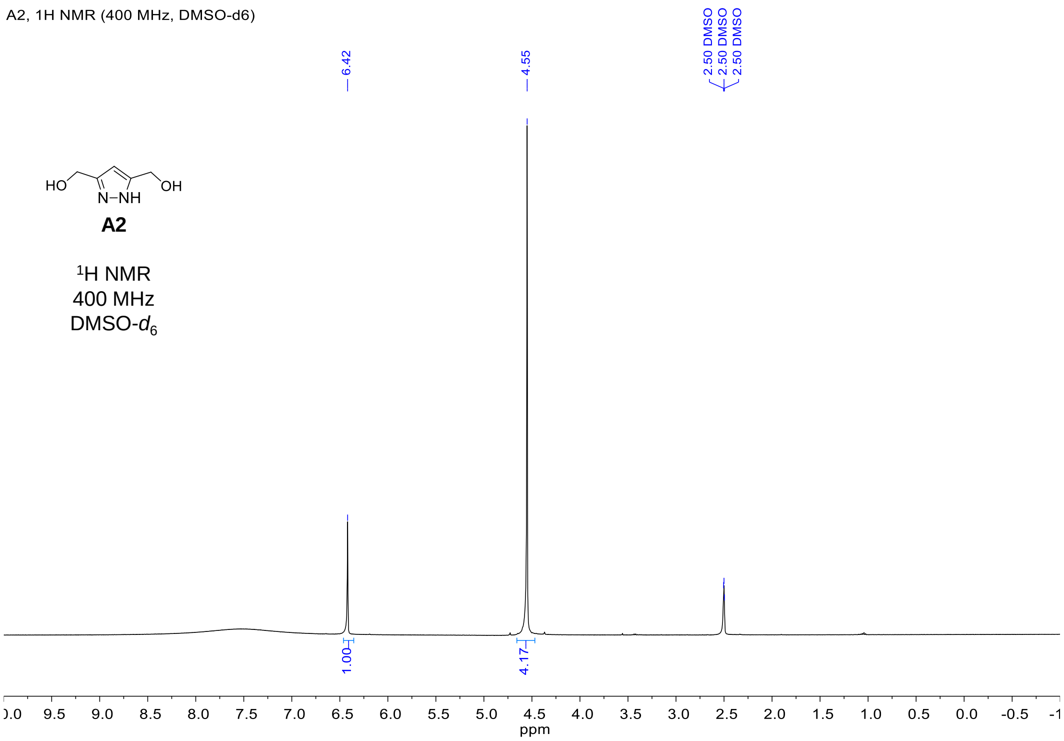

Figure S39. ^{1}H NMR spectrum of **A2**.

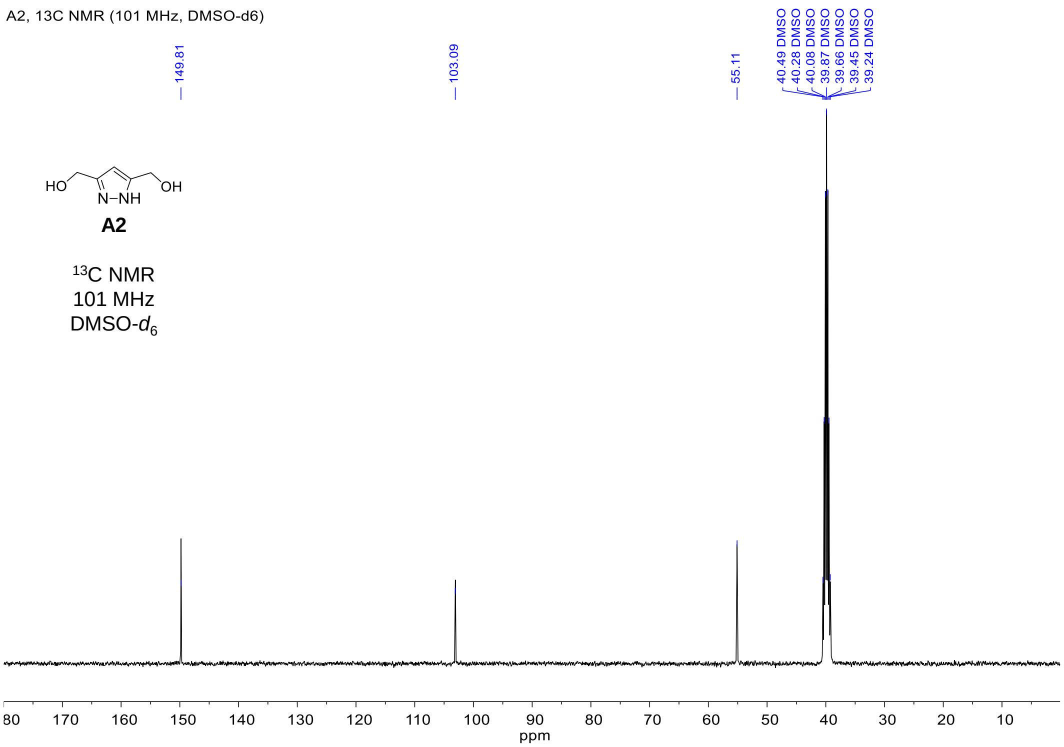

Figure S40. ^{13}C{^{1}H} NMR spectrum of **A2**.

Data:YTW-1-006-
Comment:
Description:
Ionization Mode:ESI-
History:Average(MS[1] 0.44..0.45)

Acquired:1/17/2022 2:57:56 PM
Operator:AccuTOF
m/z Calibration File:2021201-TFANa_...
Created:1/17/2022 3:01:12 PM
Created by:AccuTOF

Charge number:1
Tolerance:300.00[ppm], 250.00 .. 250....
Unsaturation Number:-100.0 .. 200.0 (...
Element:^{12}C:5 .. 5, ^{1}H:0 .. 8, ^{14}N:2 .. 2, ^{23}Na:0 .. 1, ^{16}O:2 .. 2

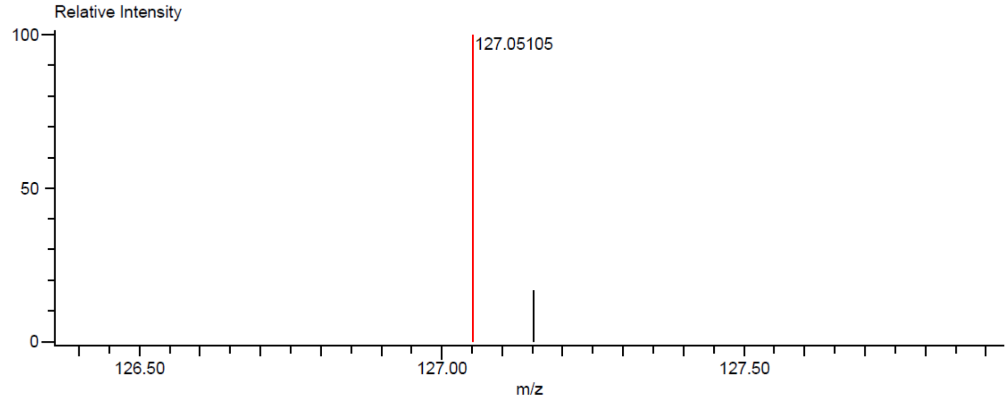

Mass	Intensity	Calc. Mass	Mass Difference [mDa]	Mass Difference [ppm]	Possible Formula
127.05105	2907.78	127.05075	0.30	2.33	$^{12}C_5{}^{1}H_7{}^{14}N_2{}^{16}O_2$

Figure S41. ESI-MS of **A2**.

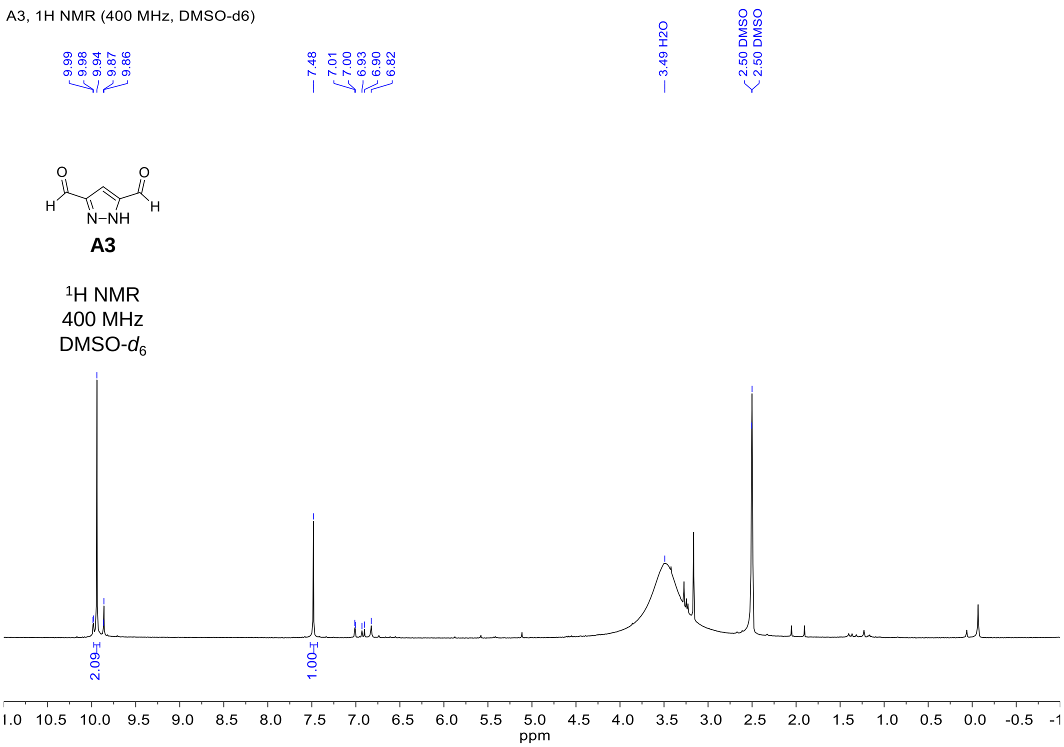

Figure S42. ^{1}H NMR spectrum of **A3**.

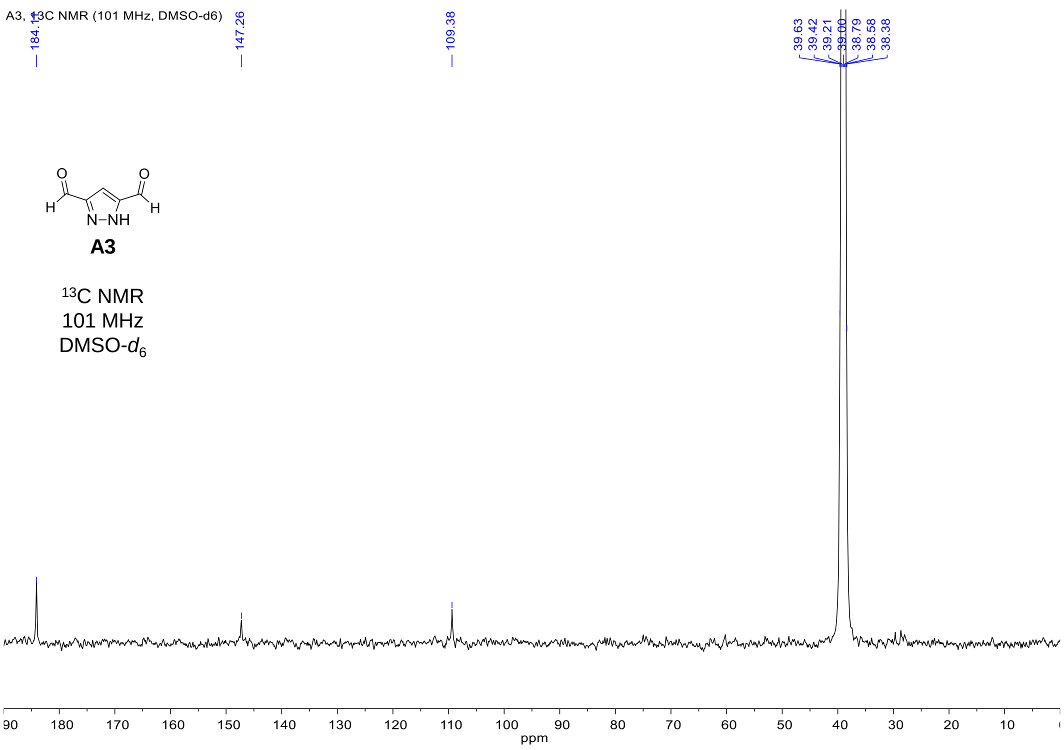

Figure S43. $^{13}C\{^1H\}$ NMR spectrum of **A3**.

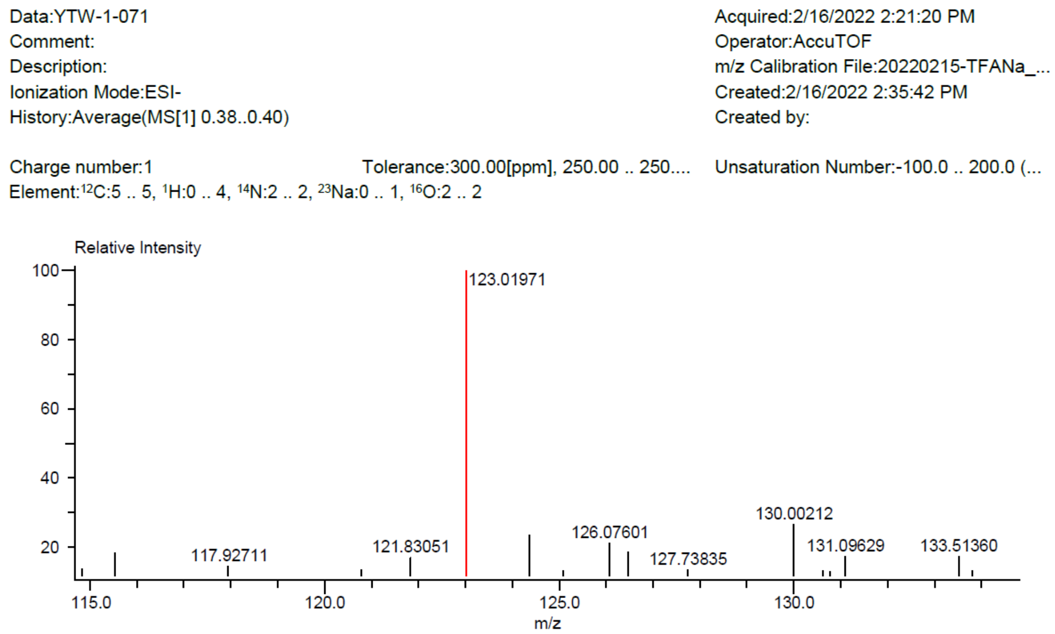

Mass	Intensity	Calc. Mass	Mass Difference [mDa]	Mass Difference [ppm]	Possible Formula
123.01971	3346.50	123.01945	0.26	2.12	$^{12}C_5{}^1H_3{}^{14}N_2{}^{16}O_2$

Figure S44. ESI-MS of **A3**.

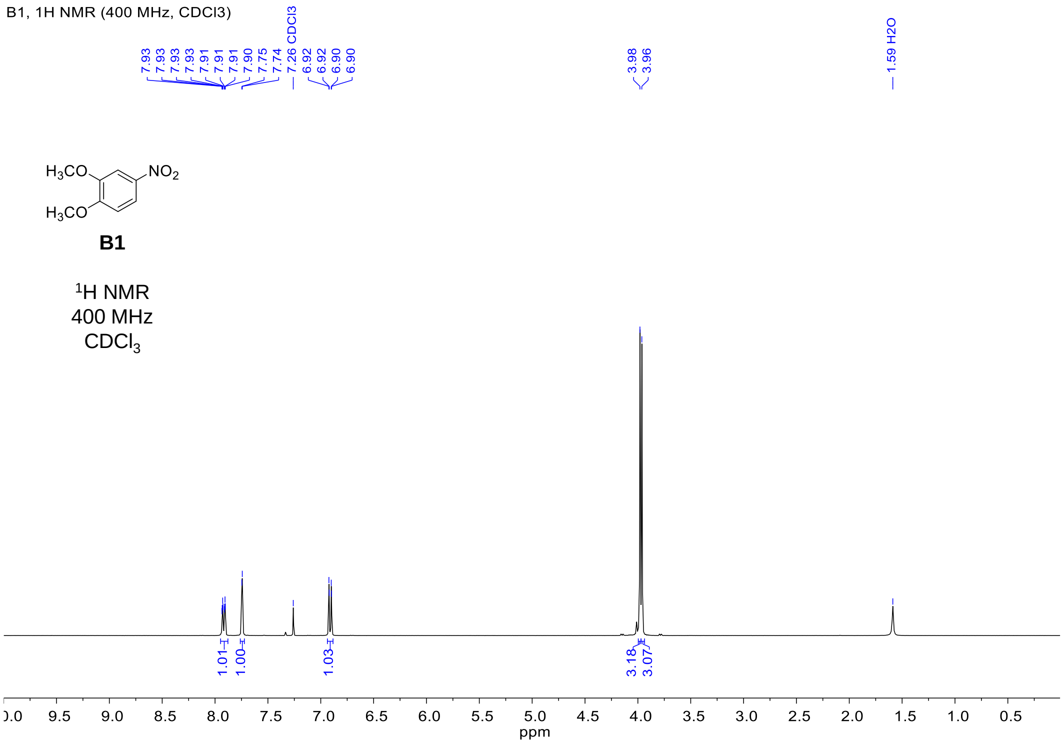

Figure S45. ^{1}H NMR spectrum of **B1**.

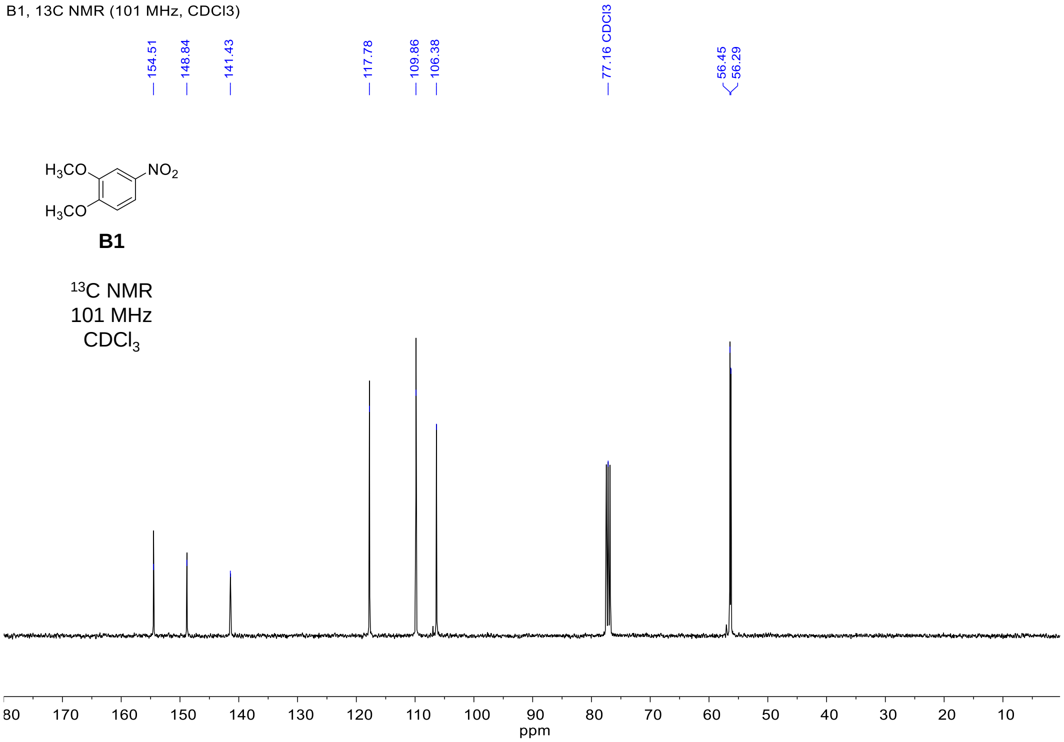

Figure S46. ^{13}C{^{1}H} NMR spectrum of **B1**.

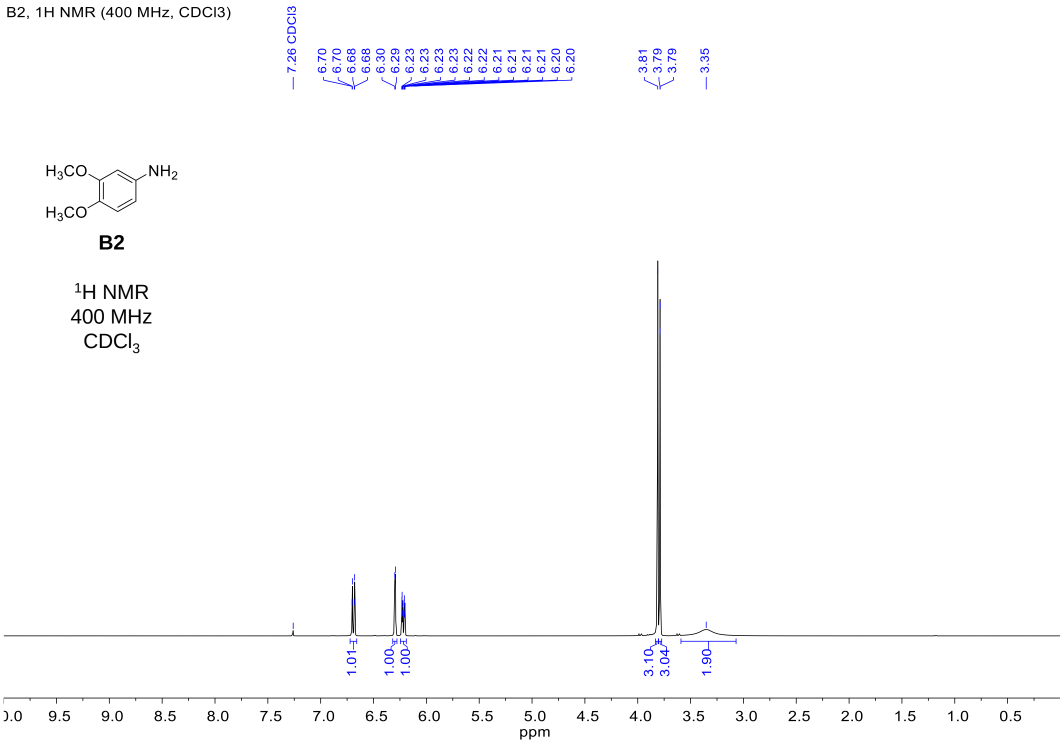

Figure S47. ^{1}H NMR spectrum of **B2**.

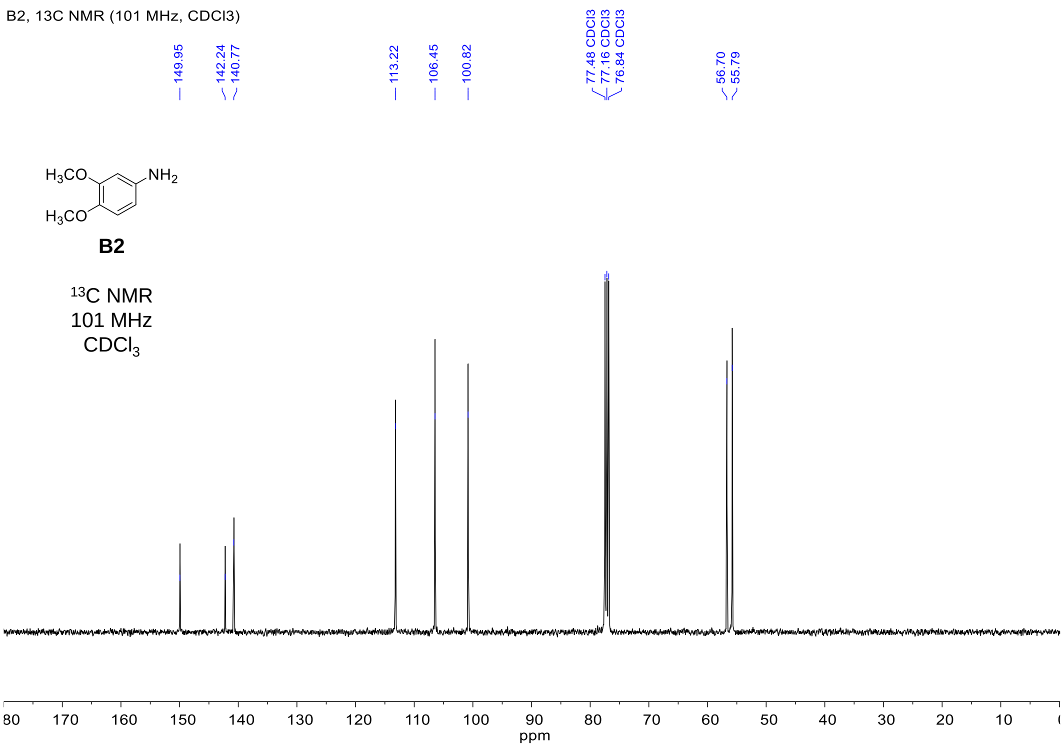

Figure S48. $^{13}C\{^{1}H\}$ NMR spectrum of **B2**.

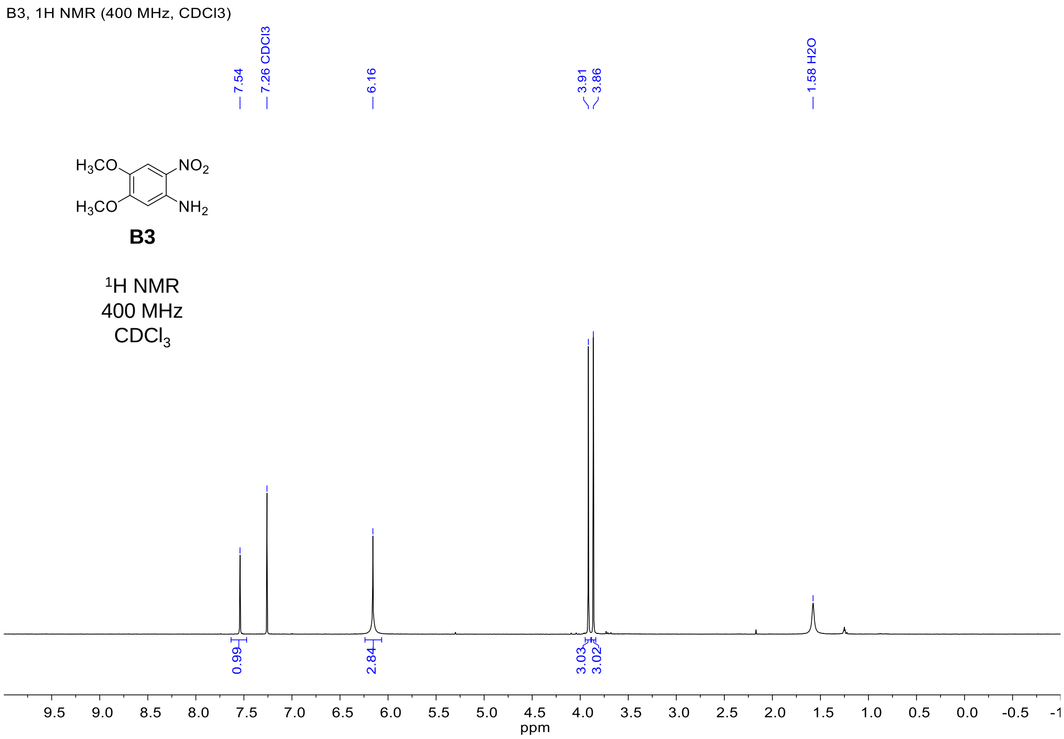

Figure S49. ^{1}H NMR spectrum of **B3**.

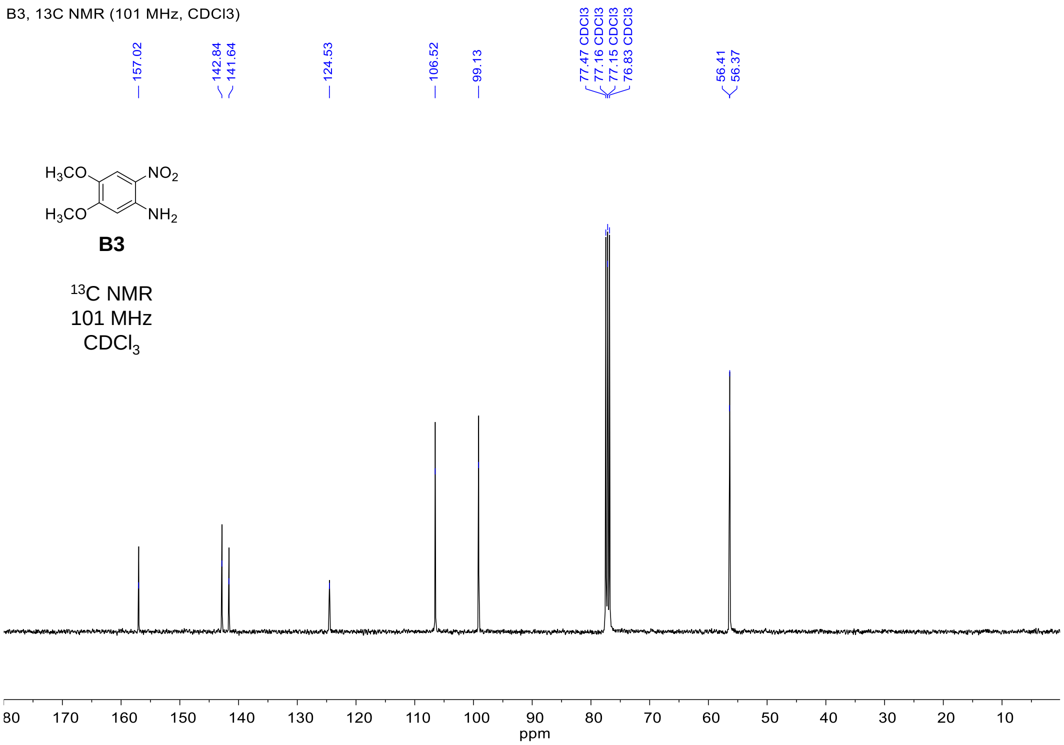

Figure S50. $^{13}C\{^{1}H\}$ NMR spectrum of **B3**.

Data: YTW I-237s
Comment:
Description:
Ionization Mode:ESI-
History:Average(MS[1] 0.34..0.36)

Acquired:3/4/2022 3:50:18 PM
Operator:AccuTOF
m/z Calibration File:20220216-TFANa_...
Created:3/4/2022 4:05:43 PM
Created by:AccuTOF

Charge number:1
Tolerance:300.00[ppm], 250.00 .. 250....
Unsaturation Number:-100.0 .. 200.0 (...
Element:^{12}C:8 .. 8, ^{1}H:0 .. 10, ^{14}N:2 .. 2, ^{23}Na:0 .. 1, ^{16}O:4 .. 4

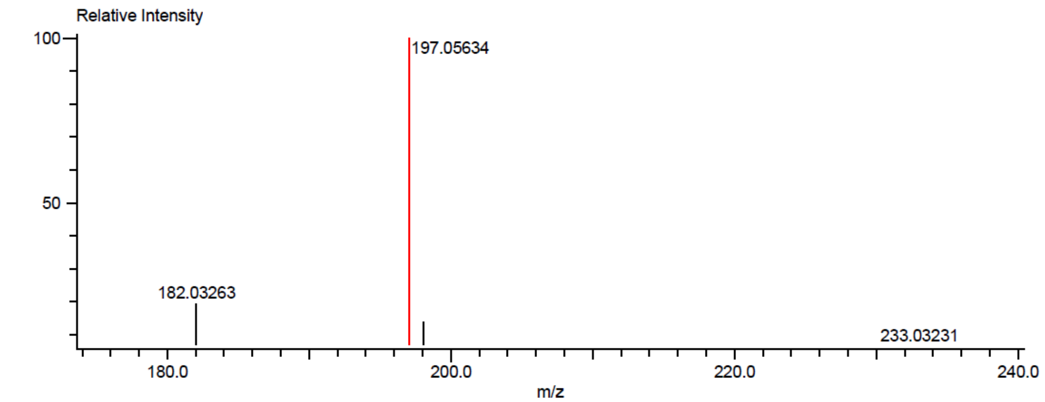

Mass	Intensity	Calc. Mass	Mass Difference [mDa]	Mass Difference [ppm]	Possible Formula
197.05634	13906.73	197.05623	0.11	0.54	$^{12}C_8{}^{1}H_9{}^{14}N_2{}^{16}O_4$

Figure S51. ESI-MS of **B3**.

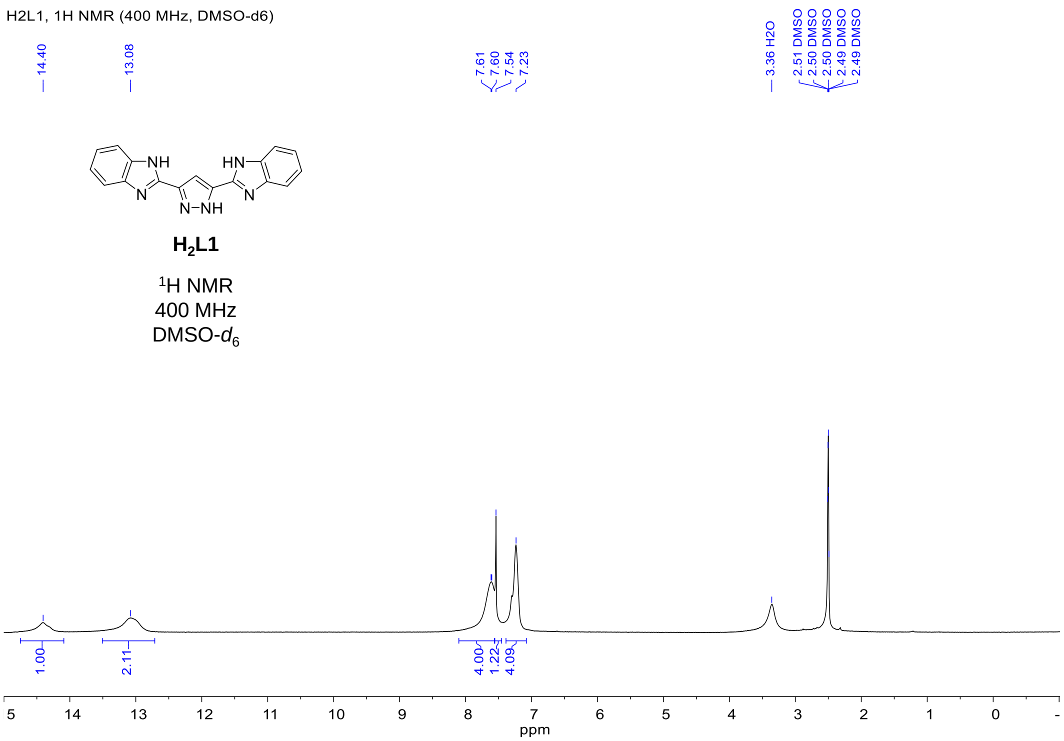

Figure S52. ^{1}H NMR spectrum of **H_2L1**.

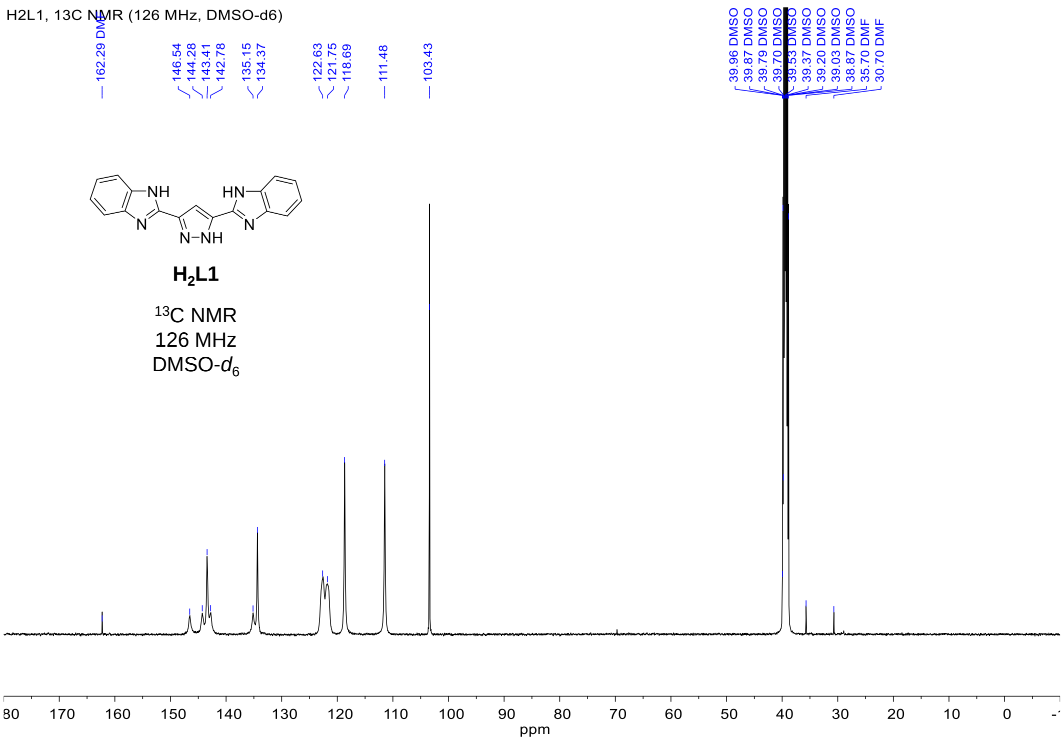

Figure S53. $^{13}C\{^{1}H\}$ NMR spectrum of **H2L1**.

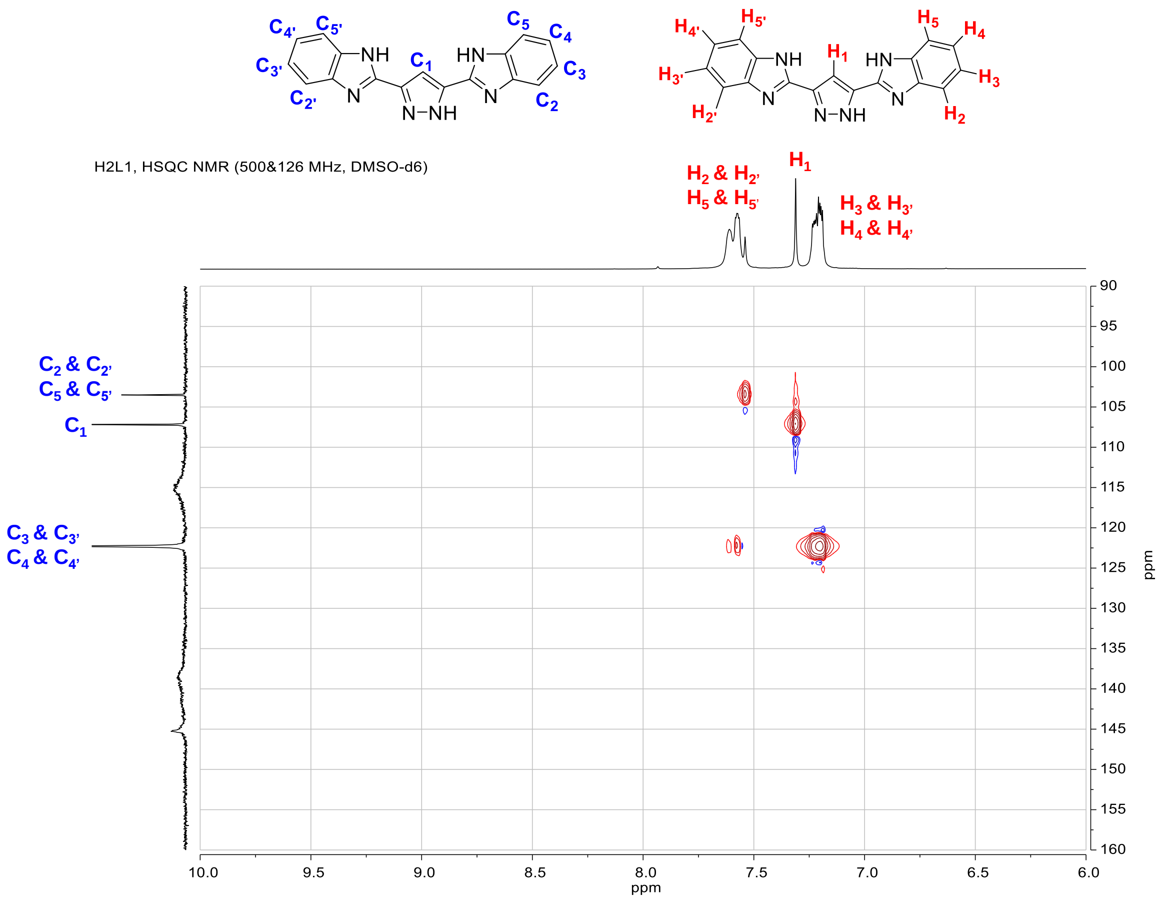

Figure S54. ^{1}H -^{13}C HSQC NMR spectrum of **H₂L1**.

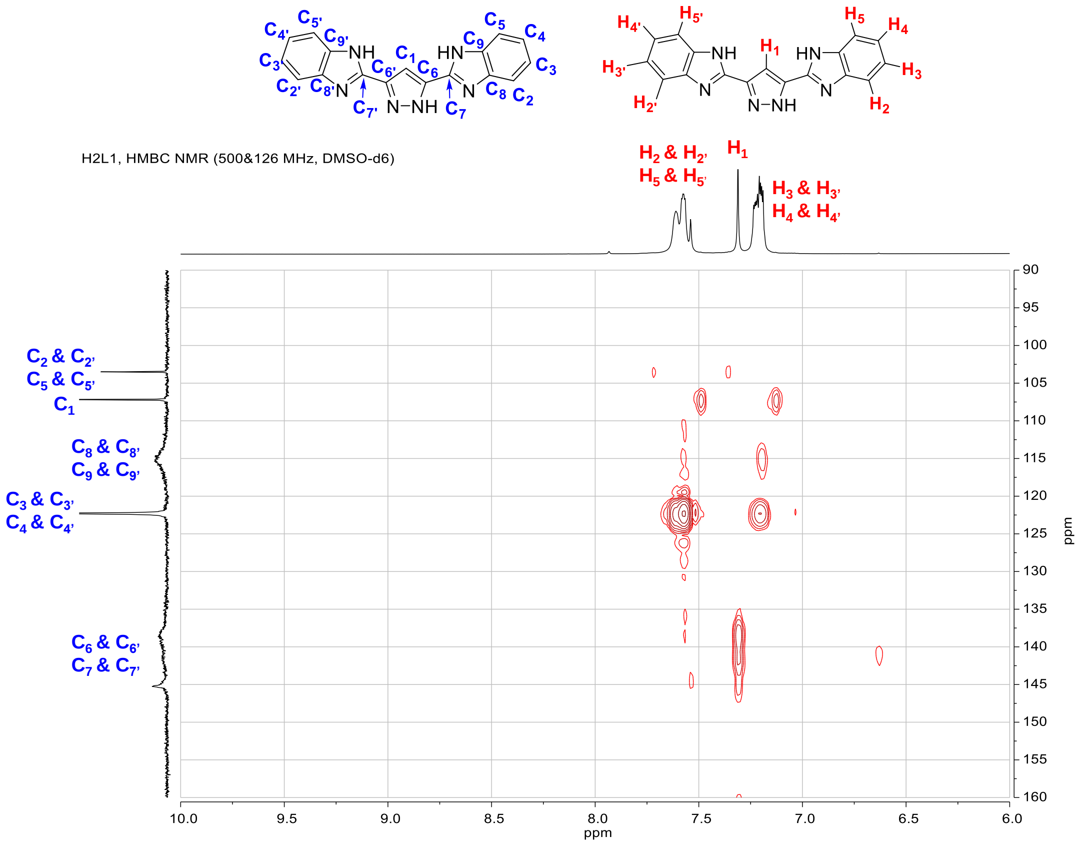

Figure S55. ^{1}H-^{13}C HMBC NMR spectrum of **H_2L1**.

Data:YTW-1-040
Comment:
Description:
Ionization Mode:ESI+
History:Average(MS[1] 0.13..0.17)

Acquired:11/16/2021 10:00:16 AM
Operator:AccuTOF
m/z Calibration File:20211110-TFANa_...
Created:11/16/2021 11:53:08 AM
Created by:AccuTOF

Charge number:1
Tolerance:300.00[ppm], 250.00 .. 250....
Unsaturation Number:-100.0 .. 200.0 (...
Element:^{12}C:17 .. 17, ^{1}H:0 .. 13, ^{14}N:6 .. 6, ^{23}Na:0 .. 2

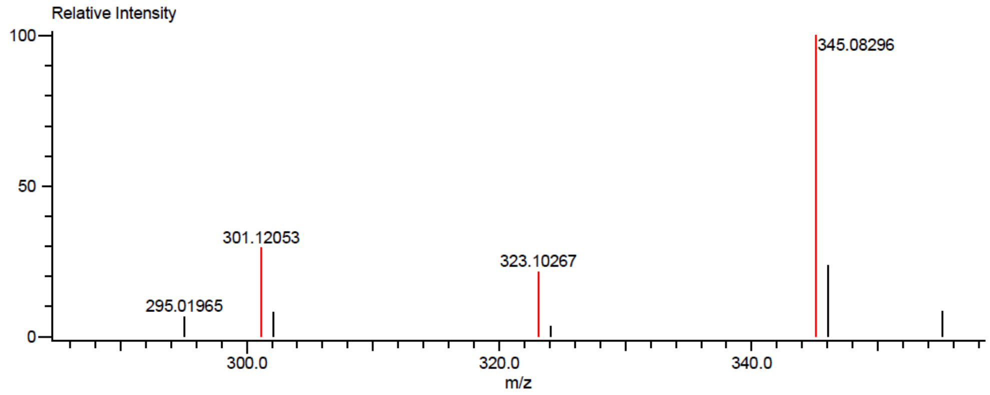

Mass	Intensity	Calc. Mass	Mass Difference [mDa]	Mass Difference [ppm]	Possible Formula
301.12053	4181.41	301.12017	0.36	1.19	$^{12}C_{17}{}^{1}H_{13}{}^{14}N_{6}$
323.10267	3028.40	323.10211	0.56	1.73	$^{12}C_{17}{}^{1}H_{12}{}^{14}N_{6}{}^{23}Na_{1}$
345.08296	14266.11	345.08406	-1.10	-3.19	$^{12}C_{17}{}^{1}H_{11}{}^{14}N_{6}{}^{23}Na_{2}$

Figure S56. ESI-MS of **H_2L1**.

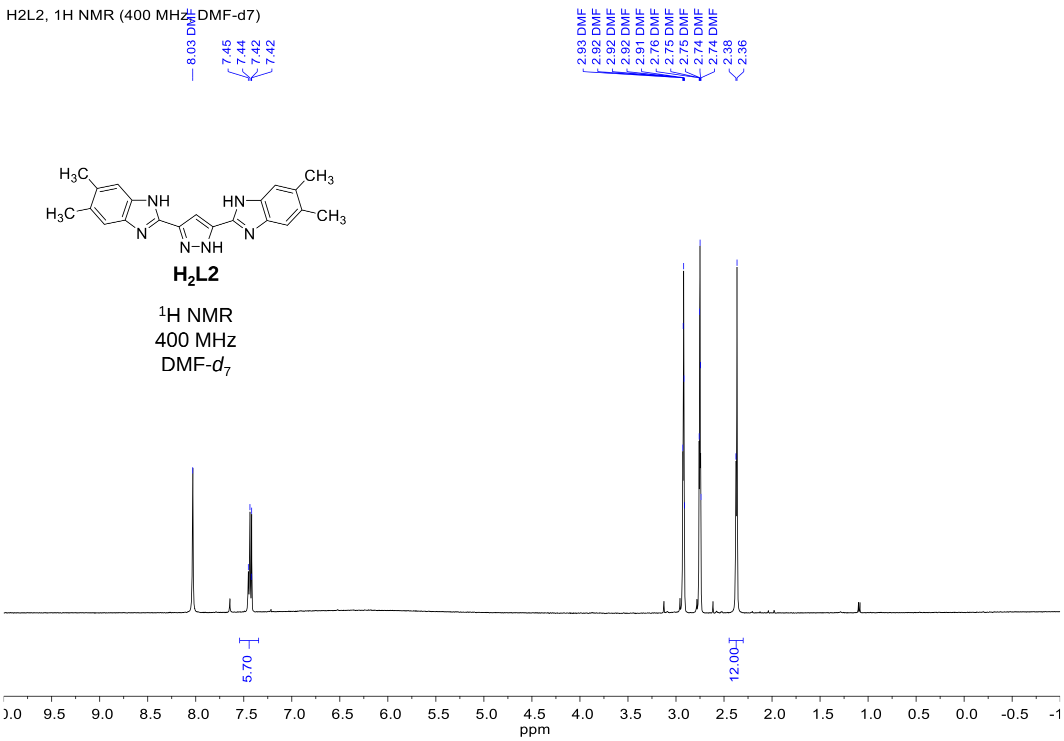

Figure S57. ^{1}H NMR spectrum of **H_2L2**.

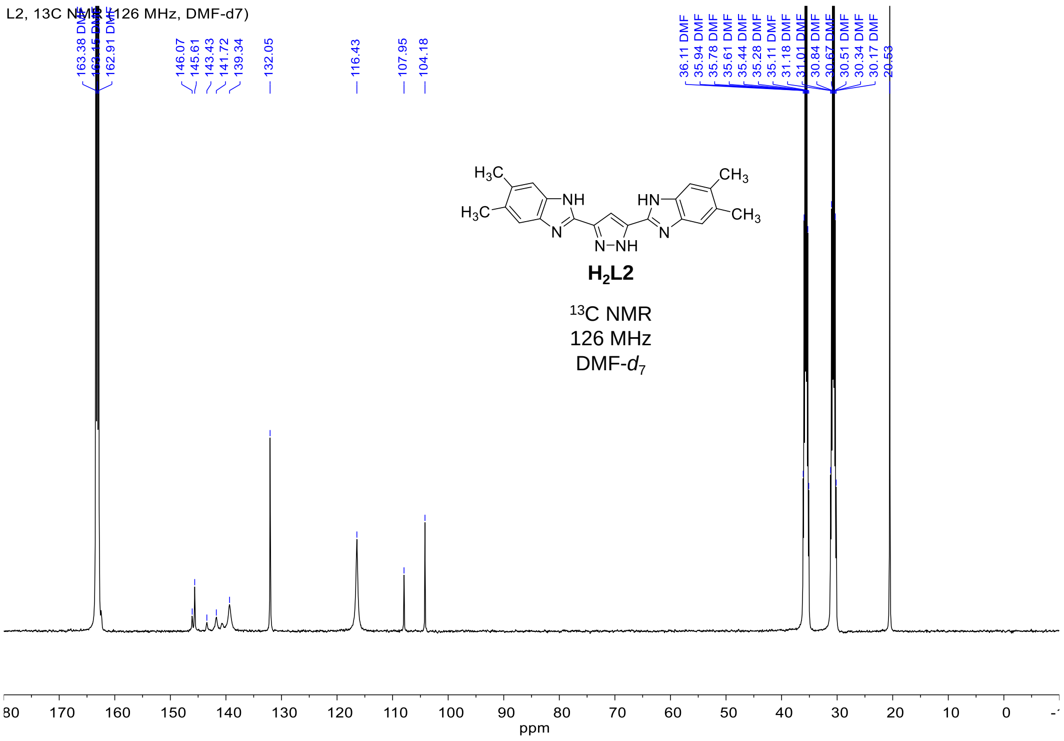

Figure S58. $^{13}C\{^{1}H\}$ NMR spectrum of **H_2L2**.

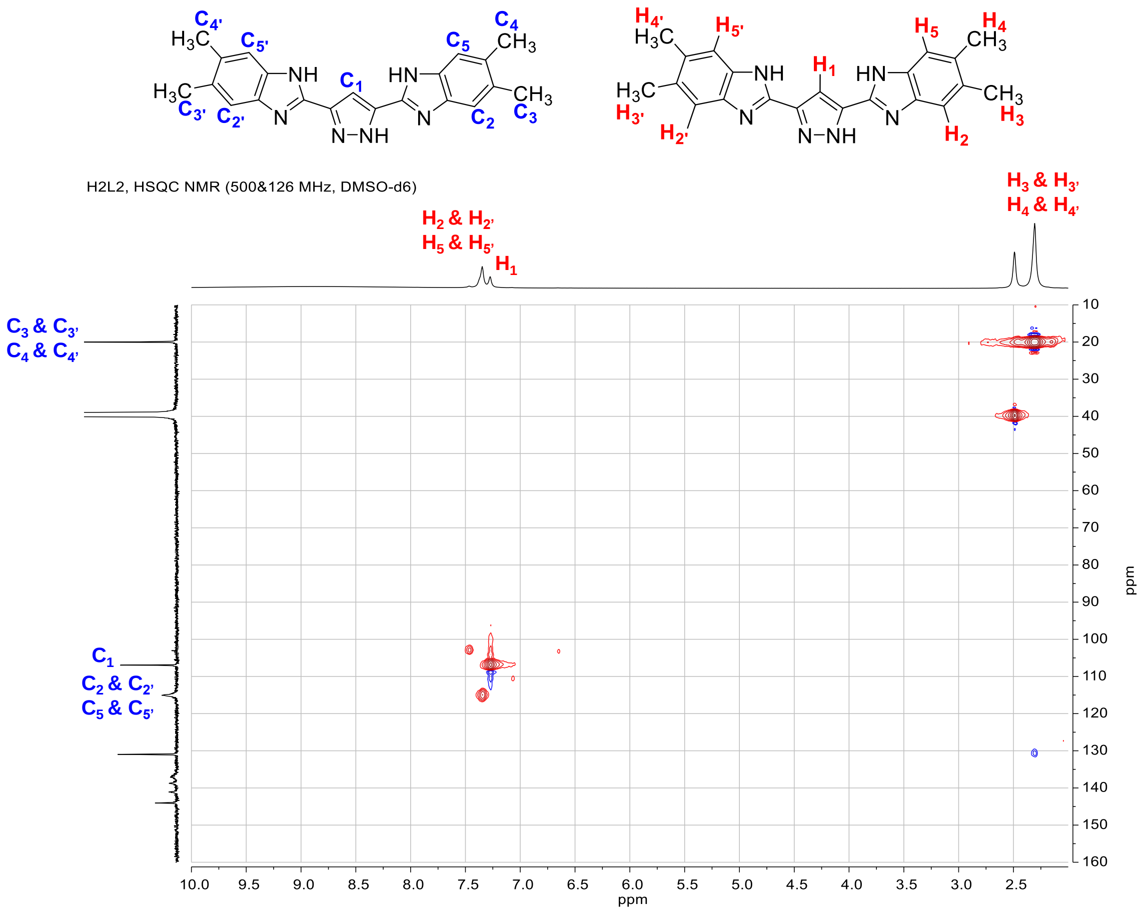

Figure S 59. ^{1}H -^{13}C HSQC NMR spectrum of **H_2L2**.

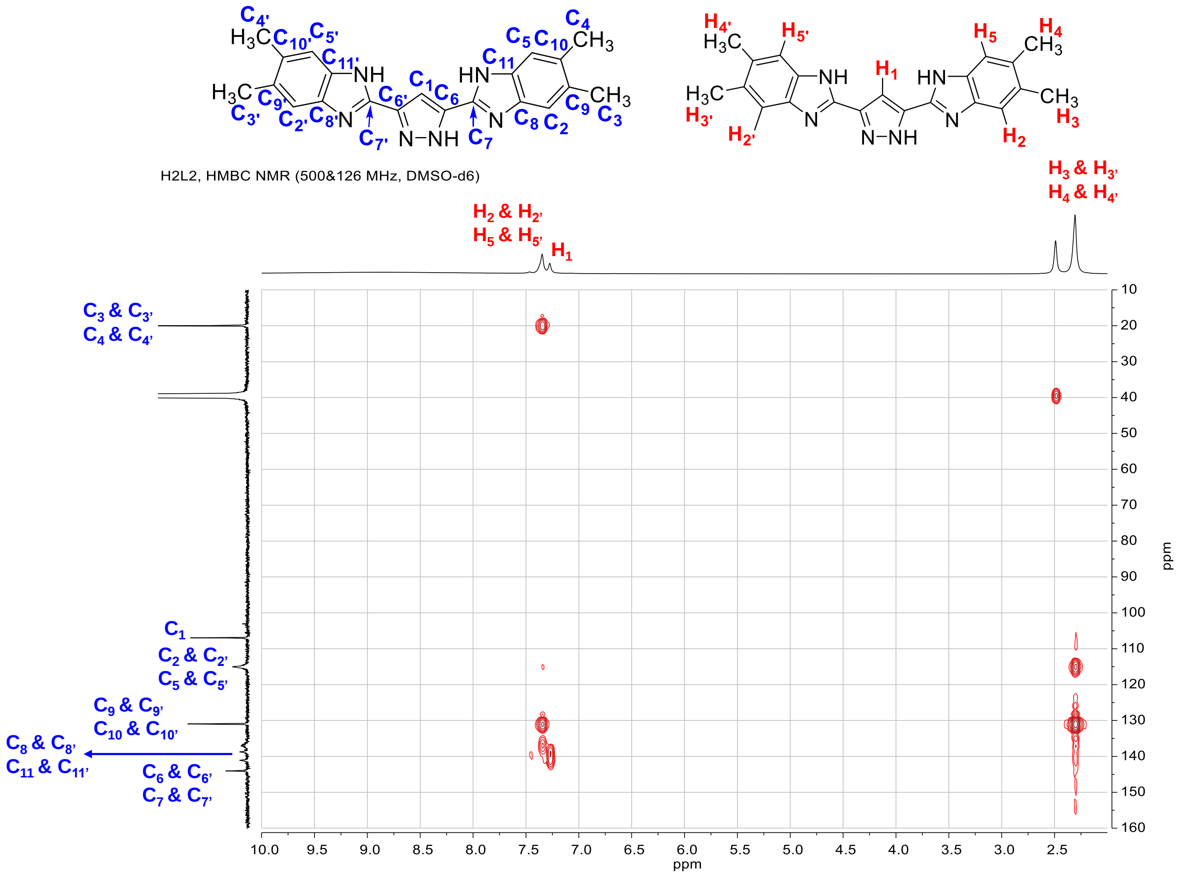

Figure S60. ^{1}H -^{13}C HMBC NMR spectrum of **H_2L2**.

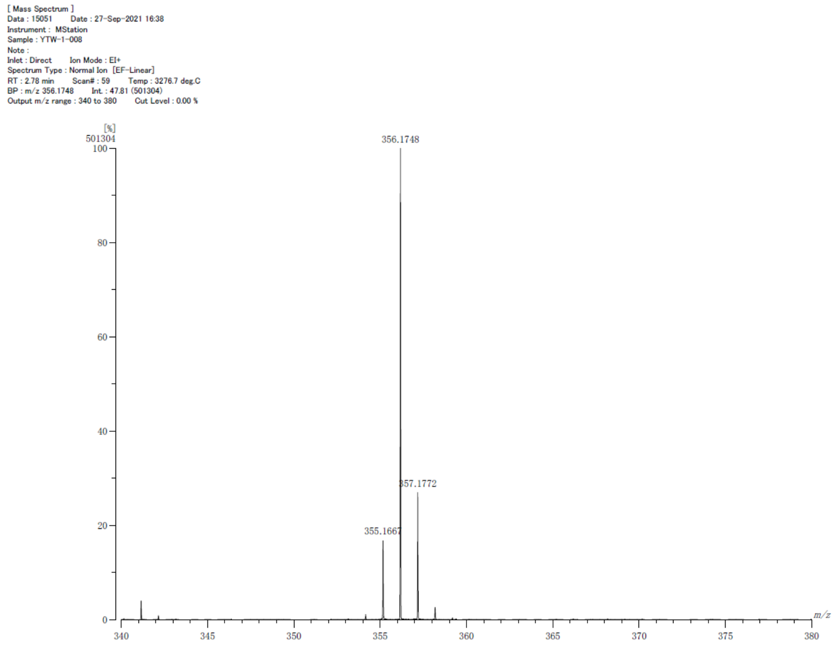

Figure S61. EI-MS of **H_2L2**.

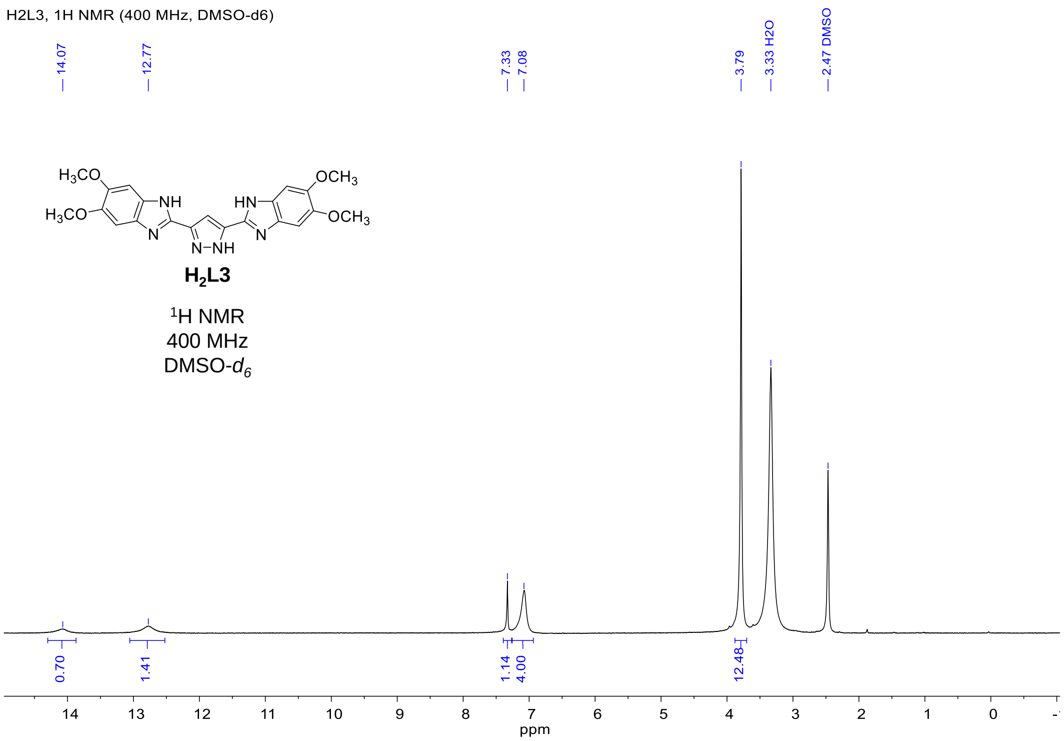

Figure S62. ^{1}H NMR spectrum of **H$_2$L3**.

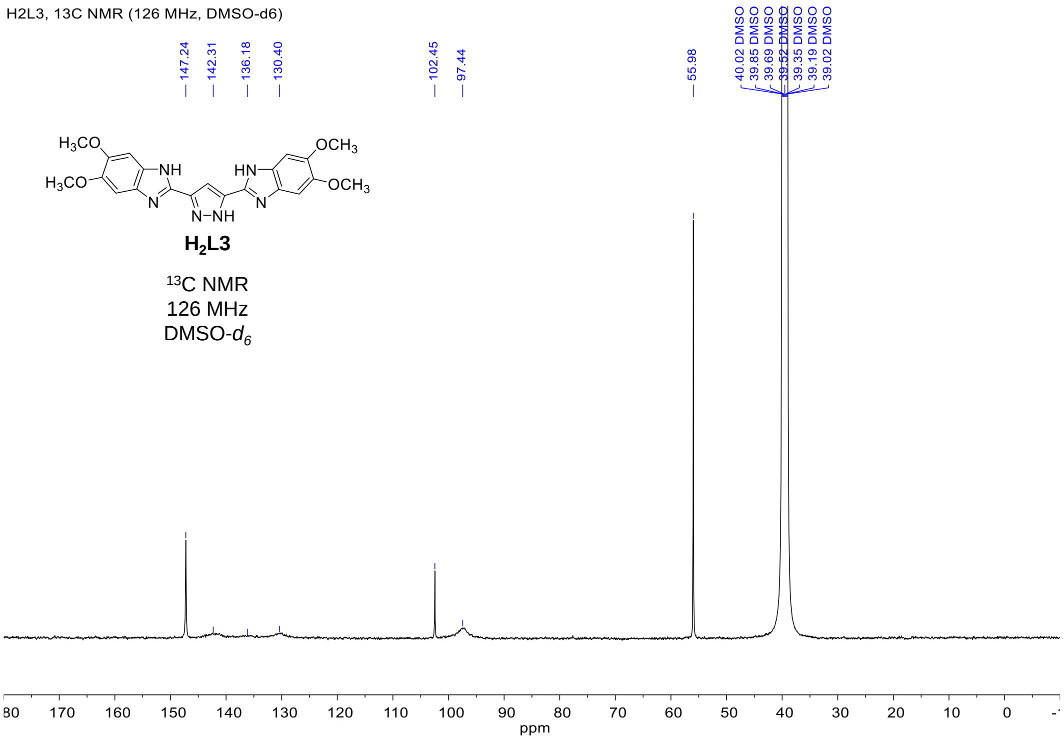

Figure S63. ^{13}C{^{1}H} NMR spectrum of **H$_2$L3**.

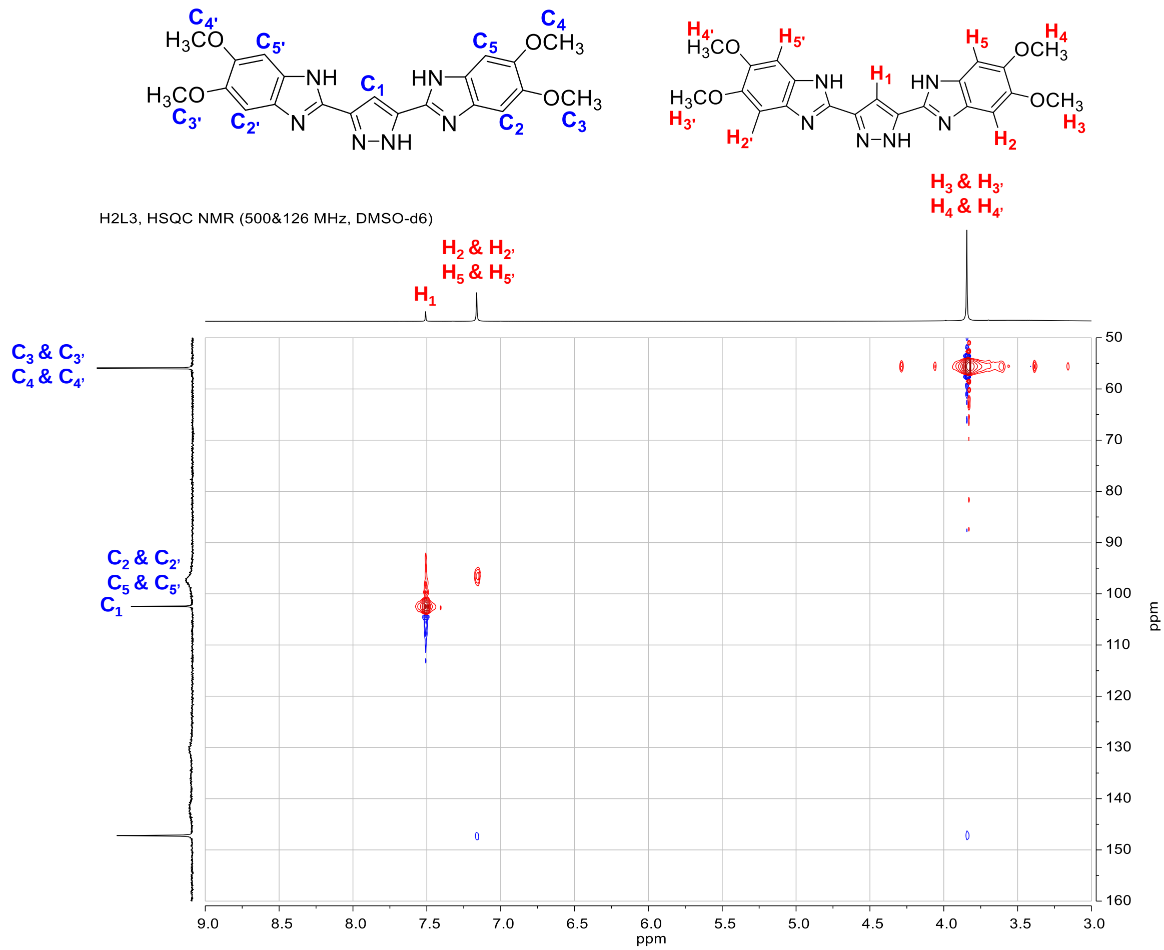

Figure S64. ^{1}H -^{13}C HSQC NMR spectrum of **H_2L3**.

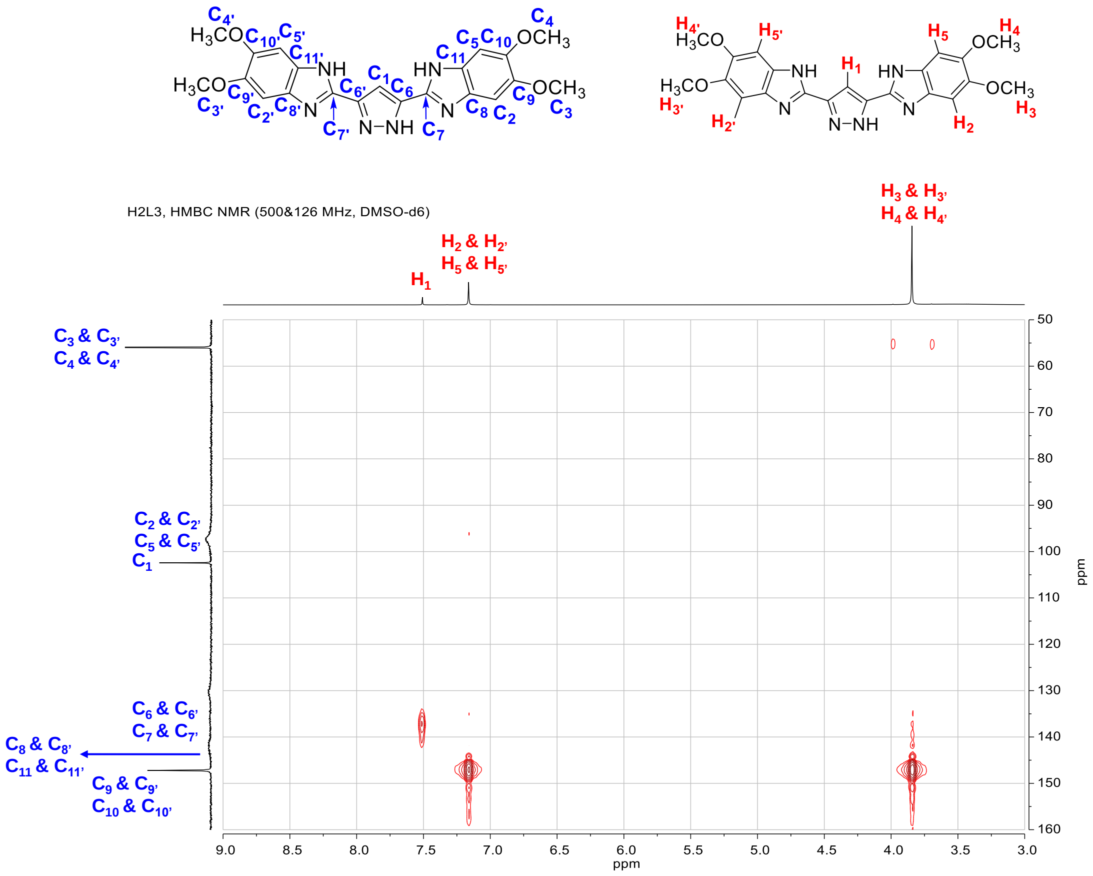

Figure S65. ^{1}H -^{13}C HMBC NMR spectrum of **H$_2$L3**.

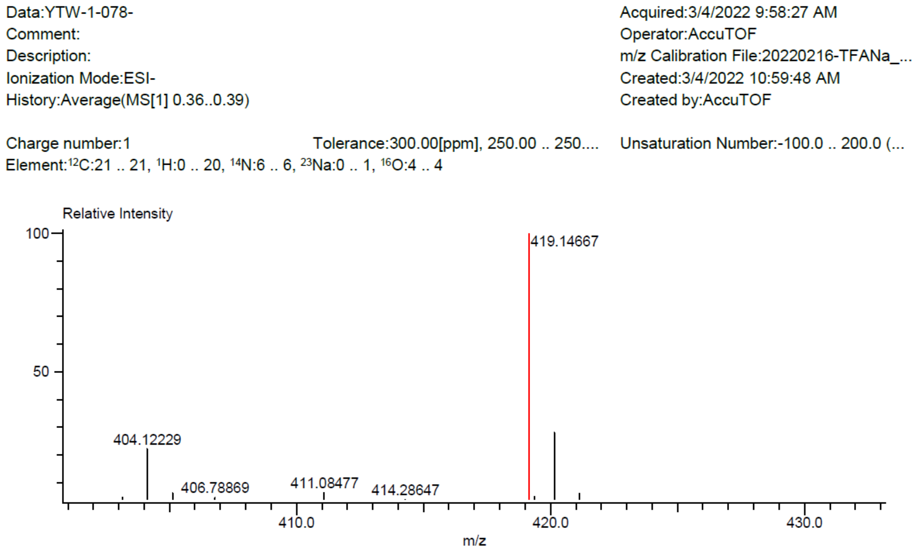

Mass	Intensity	Calc. Mass	Mass Difference [mDa]	Mass Difference [ppm]	Possible Formula
419.14667	12439.80	419.14678	-0.11	-0.26	$^{12}C_{21}{}^{1}H_{19}{}^{14}N_6{}^{16}O_4$

Figure S66. ESI-MS of **H$_2$L3**.

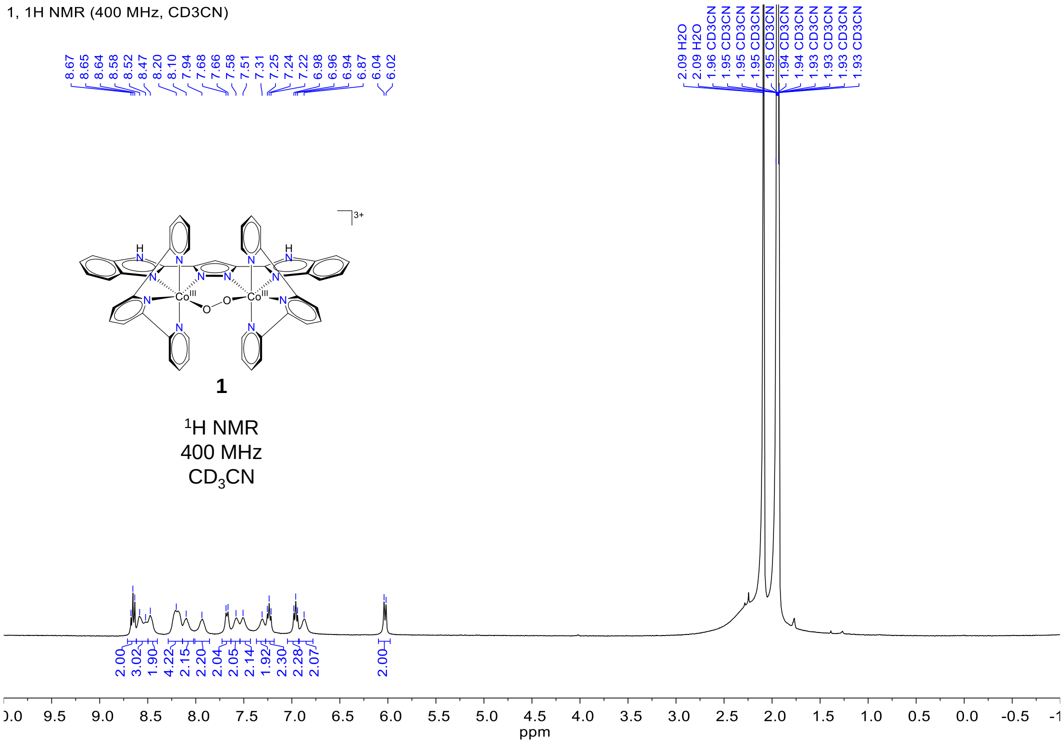

Figure S67. ^{1}H NMR spectrum of **1**.

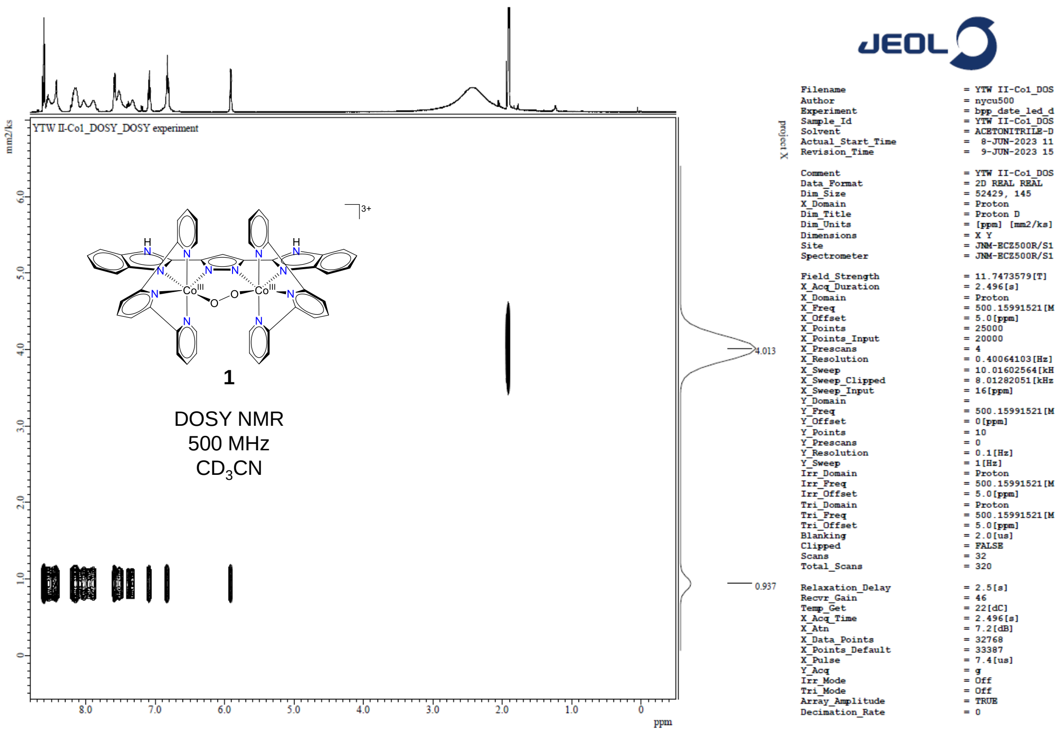

Figure S68. DOSY NMR spectrum of **1**.

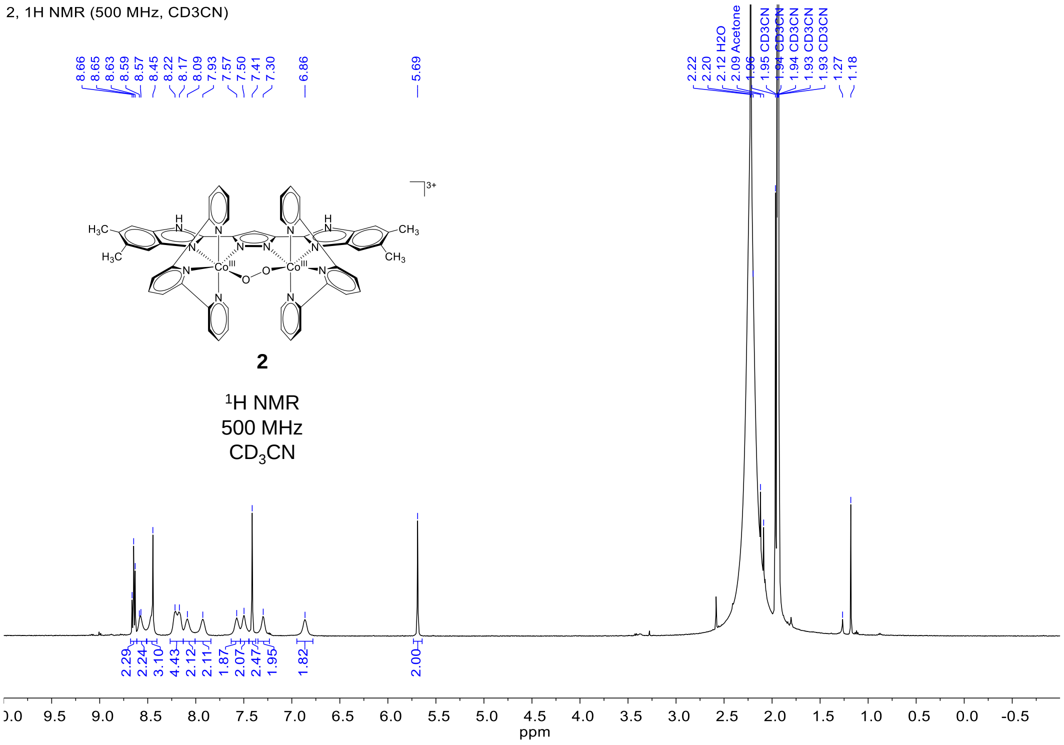

Figure S69. ^{1}H NMR spectrum of **2**.

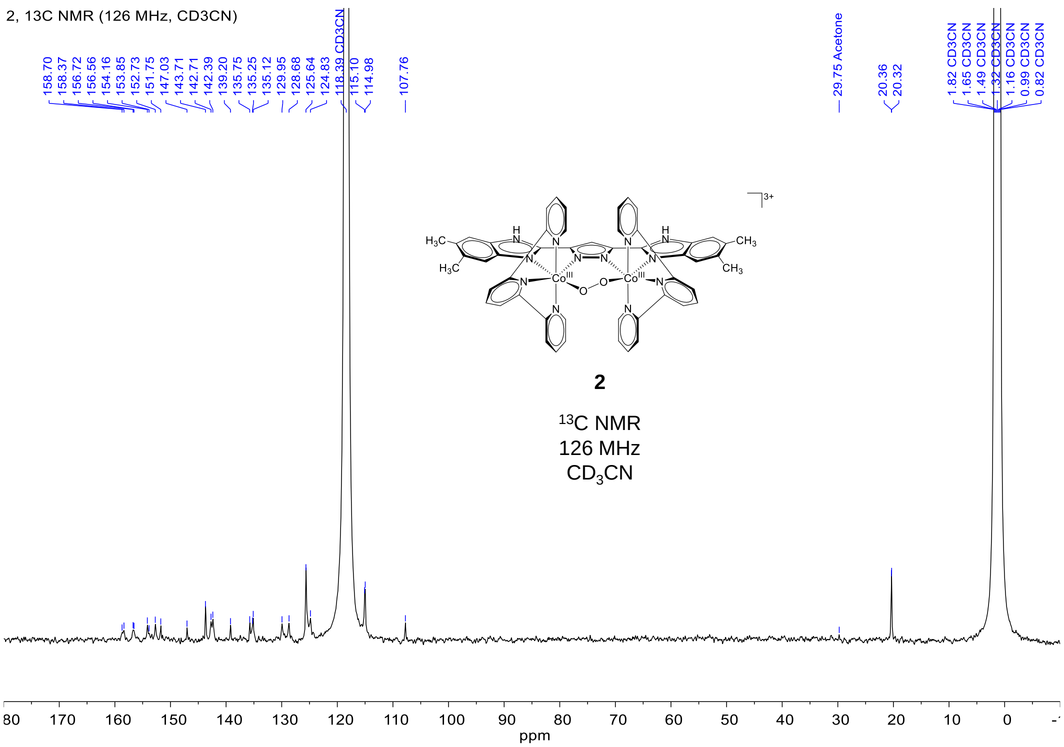

Figure S70. $^{13}C\{^1H\}$ NMR spectrum of **2**.

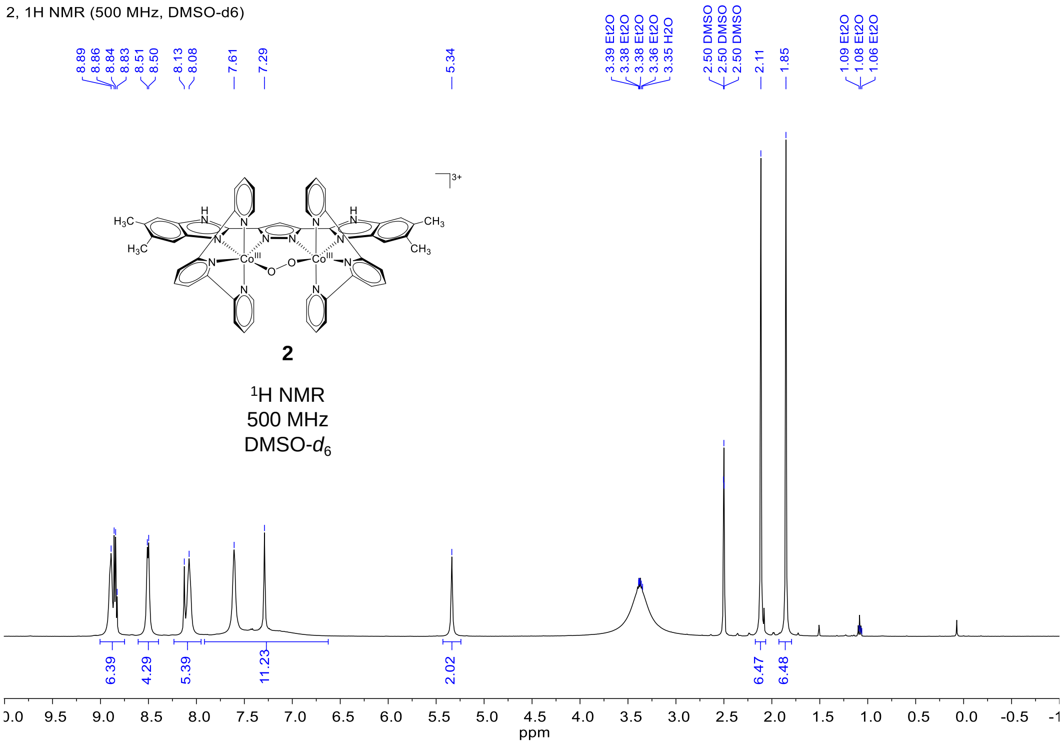

Figure S71. ^{1}H NMR spectrum of **2**.

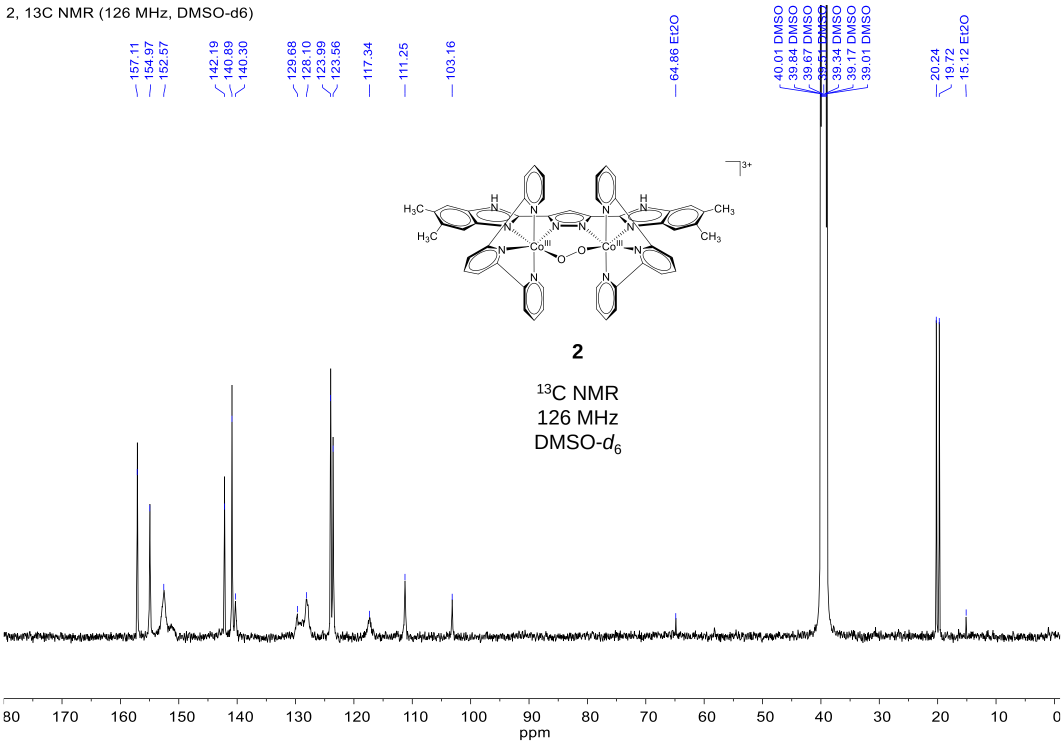

Figure S72. ^{13}C{^{1}H} NMR spectrum of **2**.

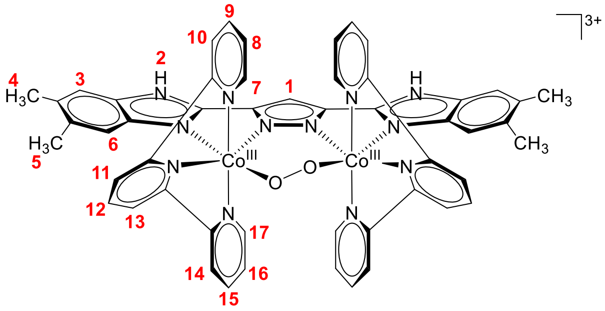

2, COSY NMR (500 MHz, CD3CN)

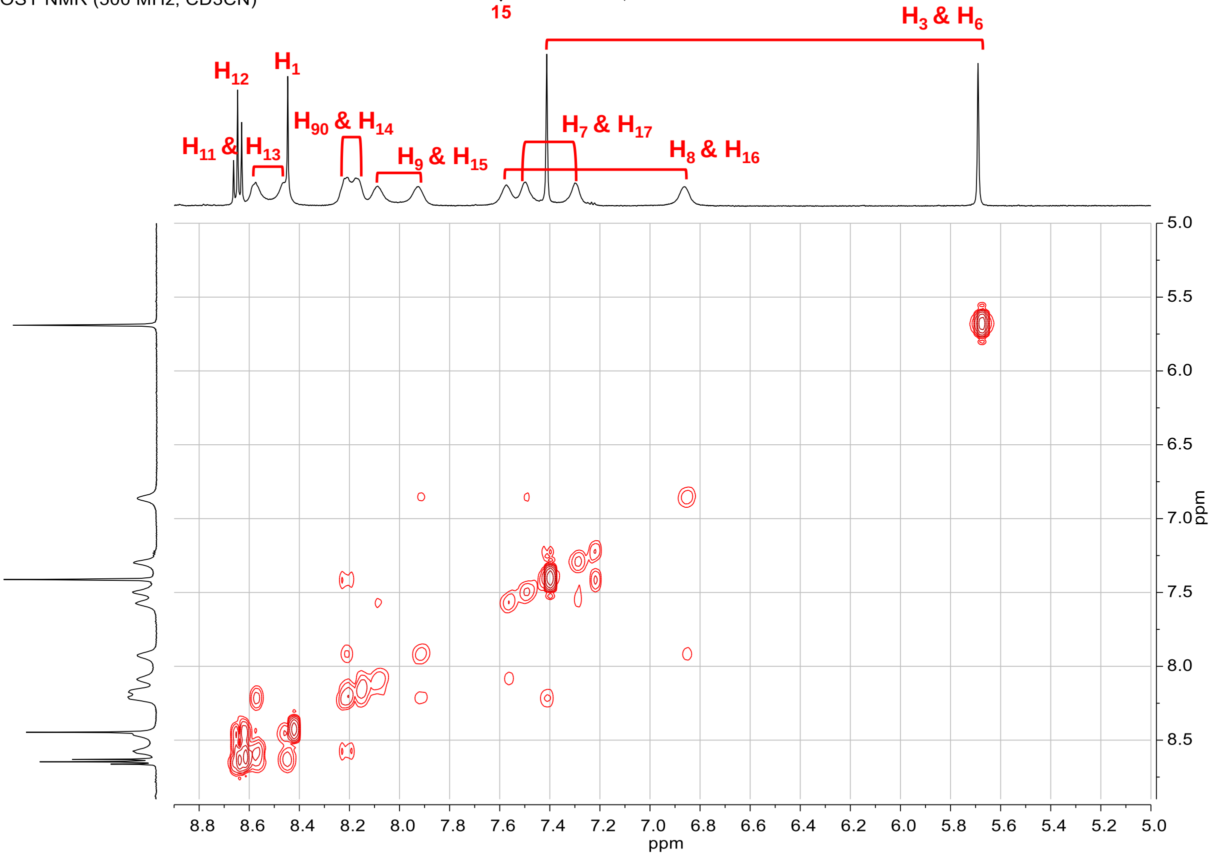

Figure S73. ^{1}H-^{1}H COSY NMR spectrum of **2**.

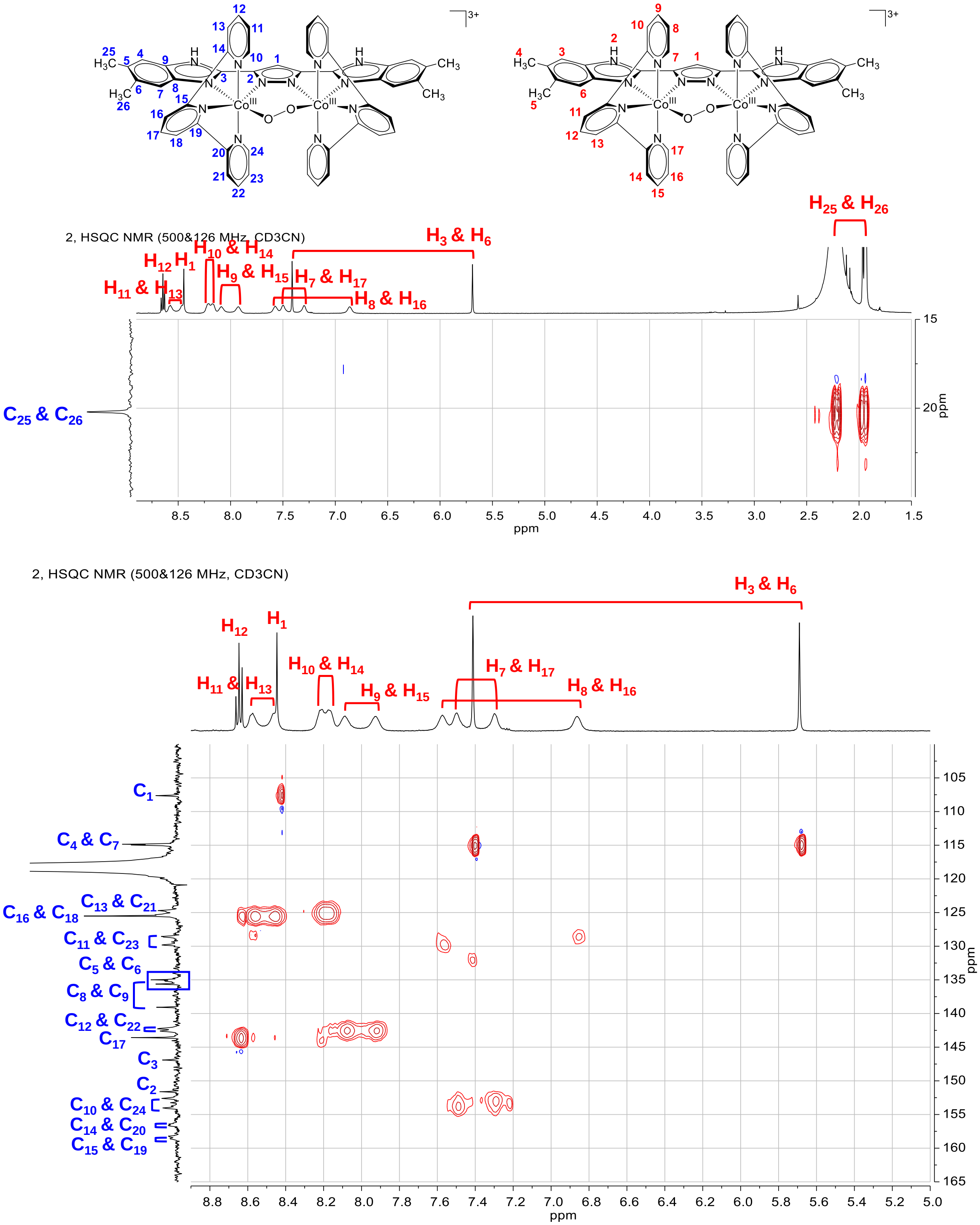

Figure S74. ^{1}H-^{13}C HSQC NMR spectrum of **2**.

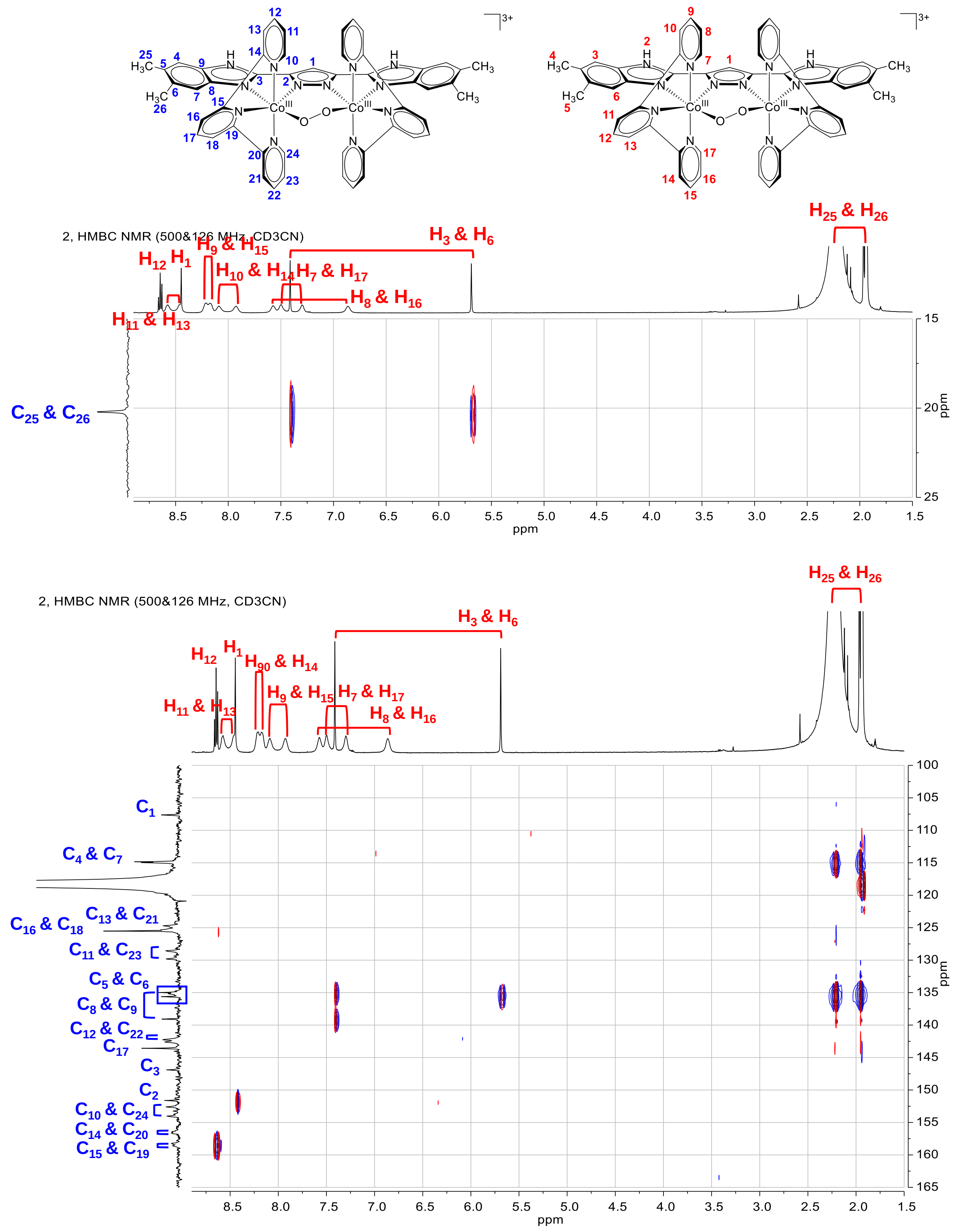

Figure S75. ^{1}H-^{13}C HMBC NMR spectrum of **2**.

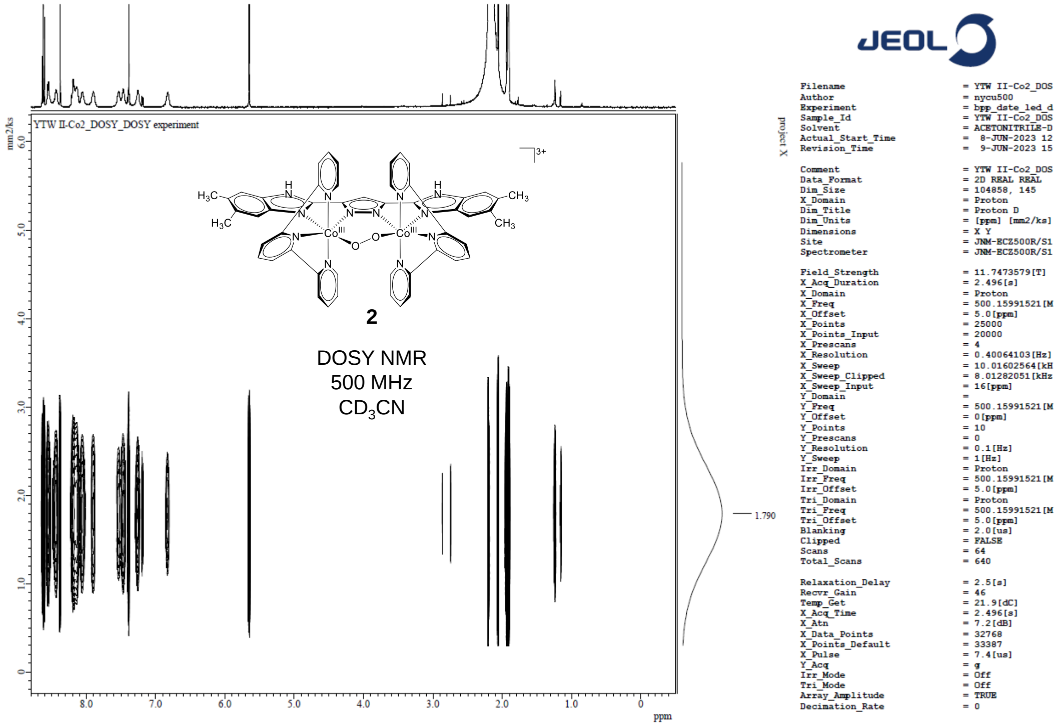

Figure S76. DOSY NMR spectrum of **2**.

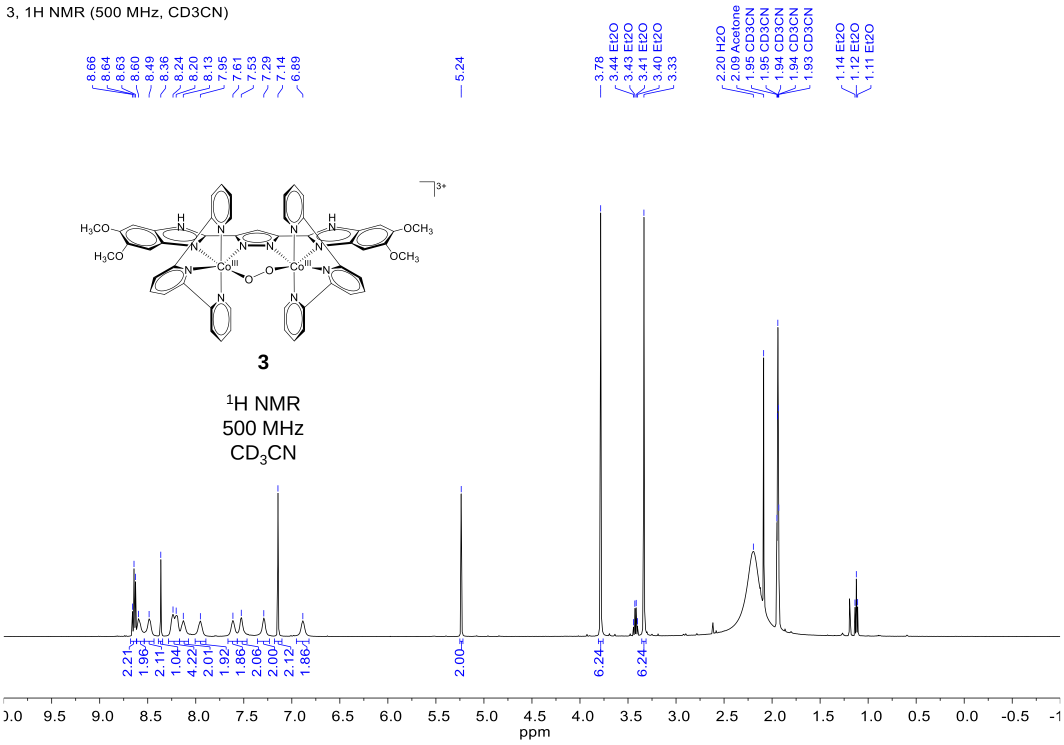

Figure S77. ^{1}H NMR spectrum of **3**.

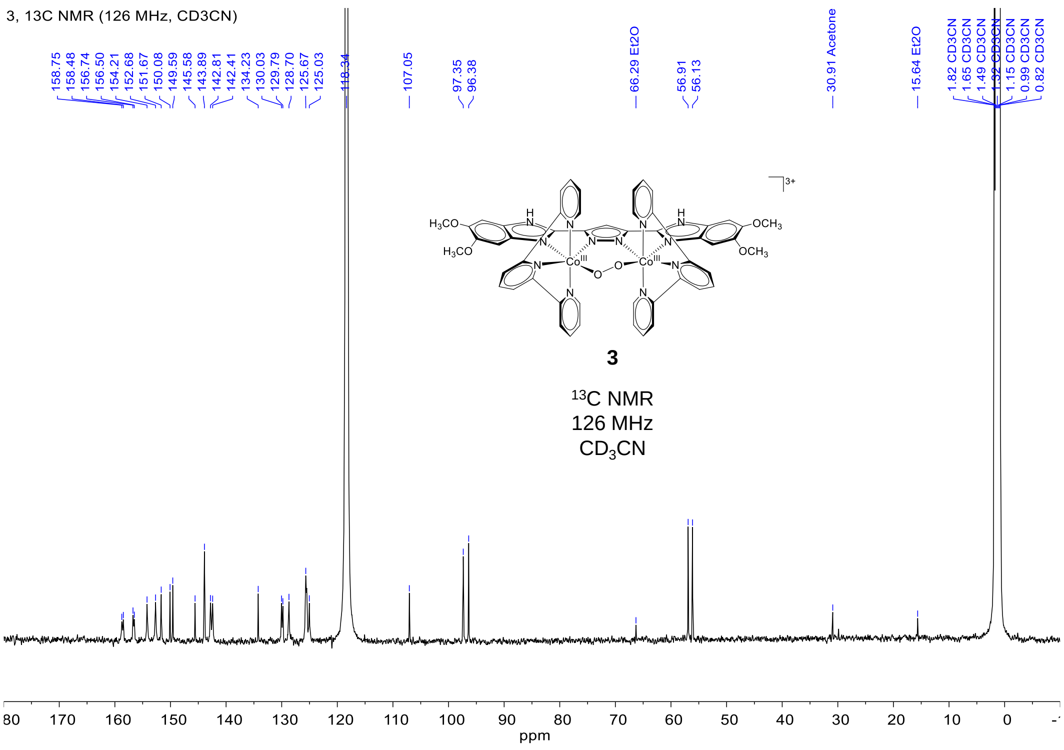

Figure S78. ^{13}C{^{1}H} NMR spectrum of **3**.

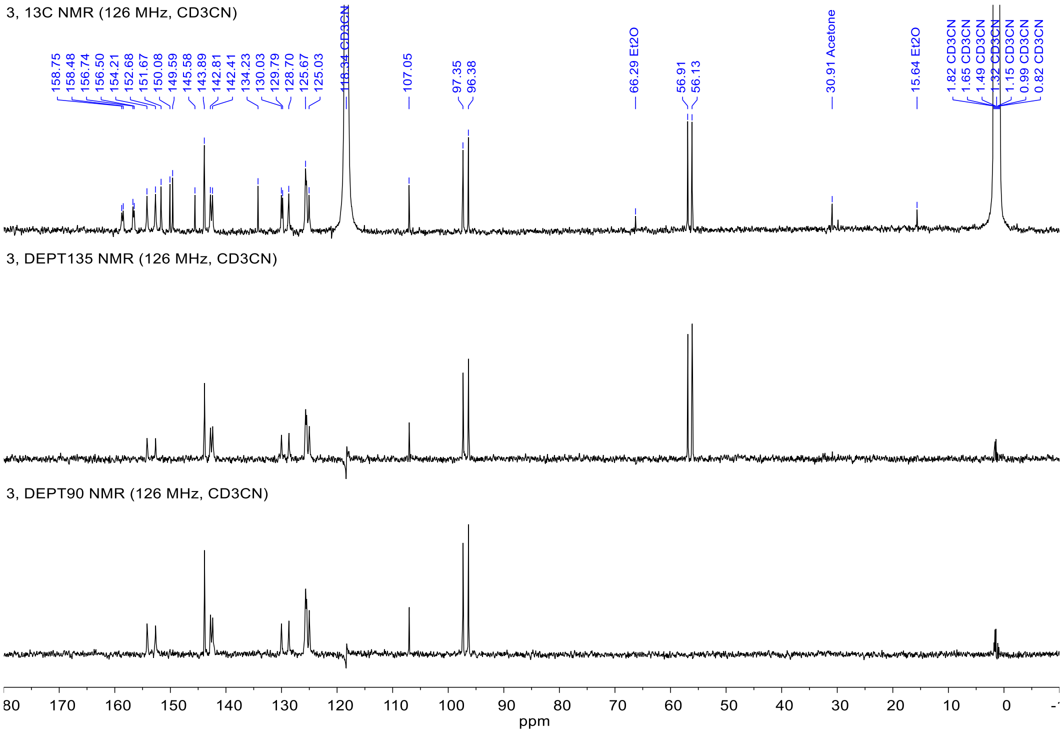

Figure S79. DEPT135 and 90 NMR spectrum of **3**.

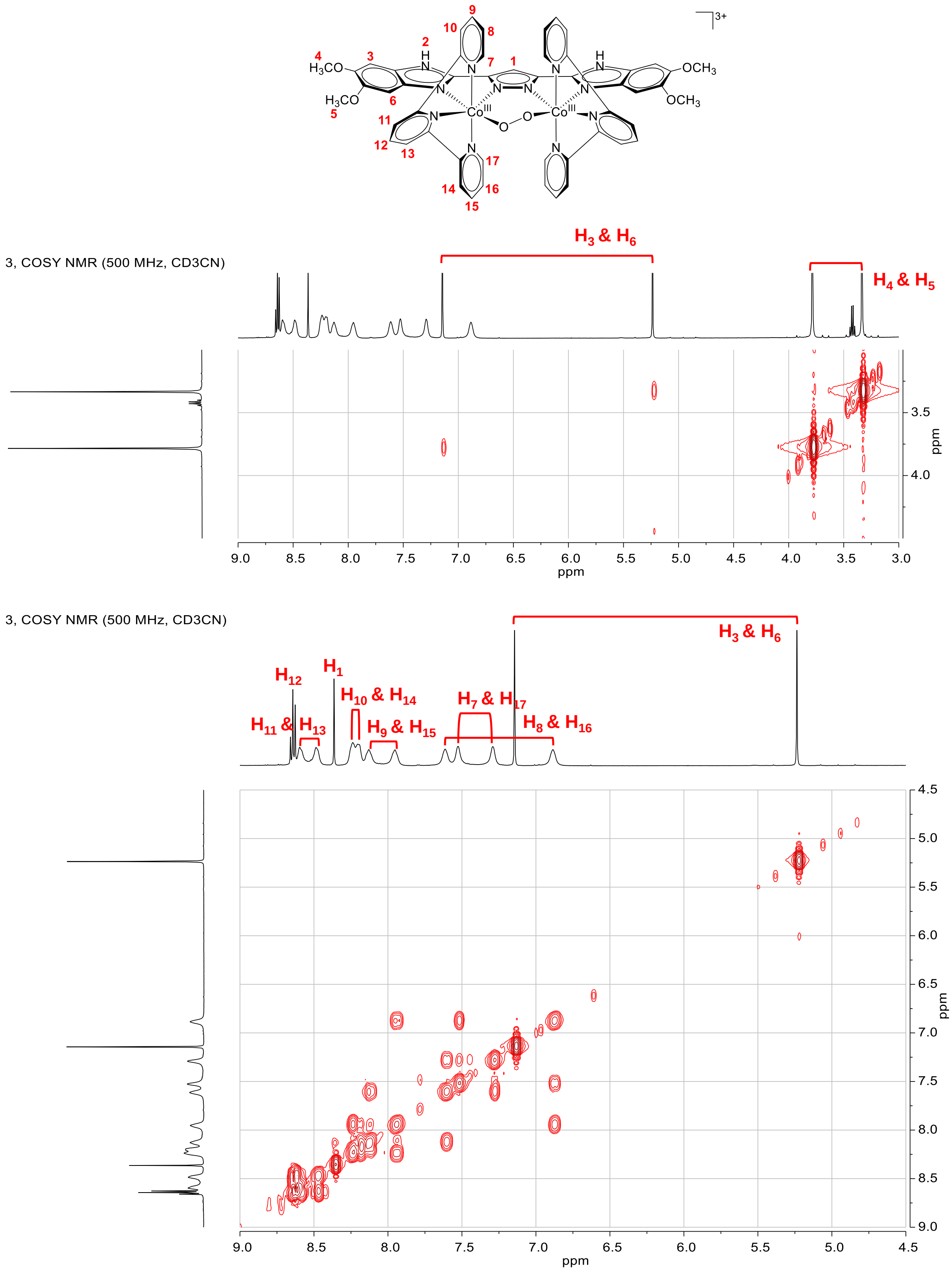

Figure S80. ^{1}H-^{1}H COSY NMR spectrum of **3**.

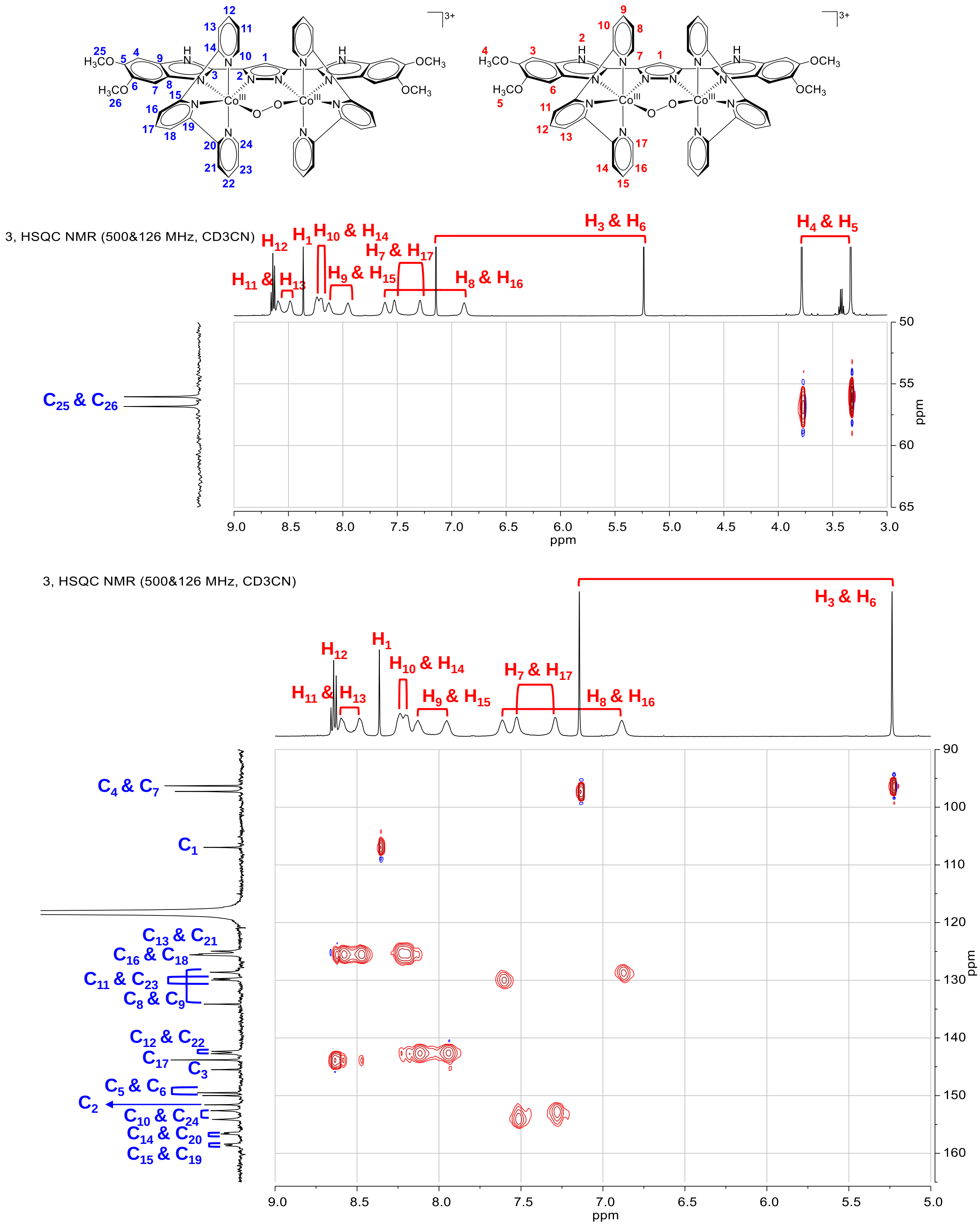

Figure S81.^{1}H-^{13}C HSQC NMR spectrum of **3**.

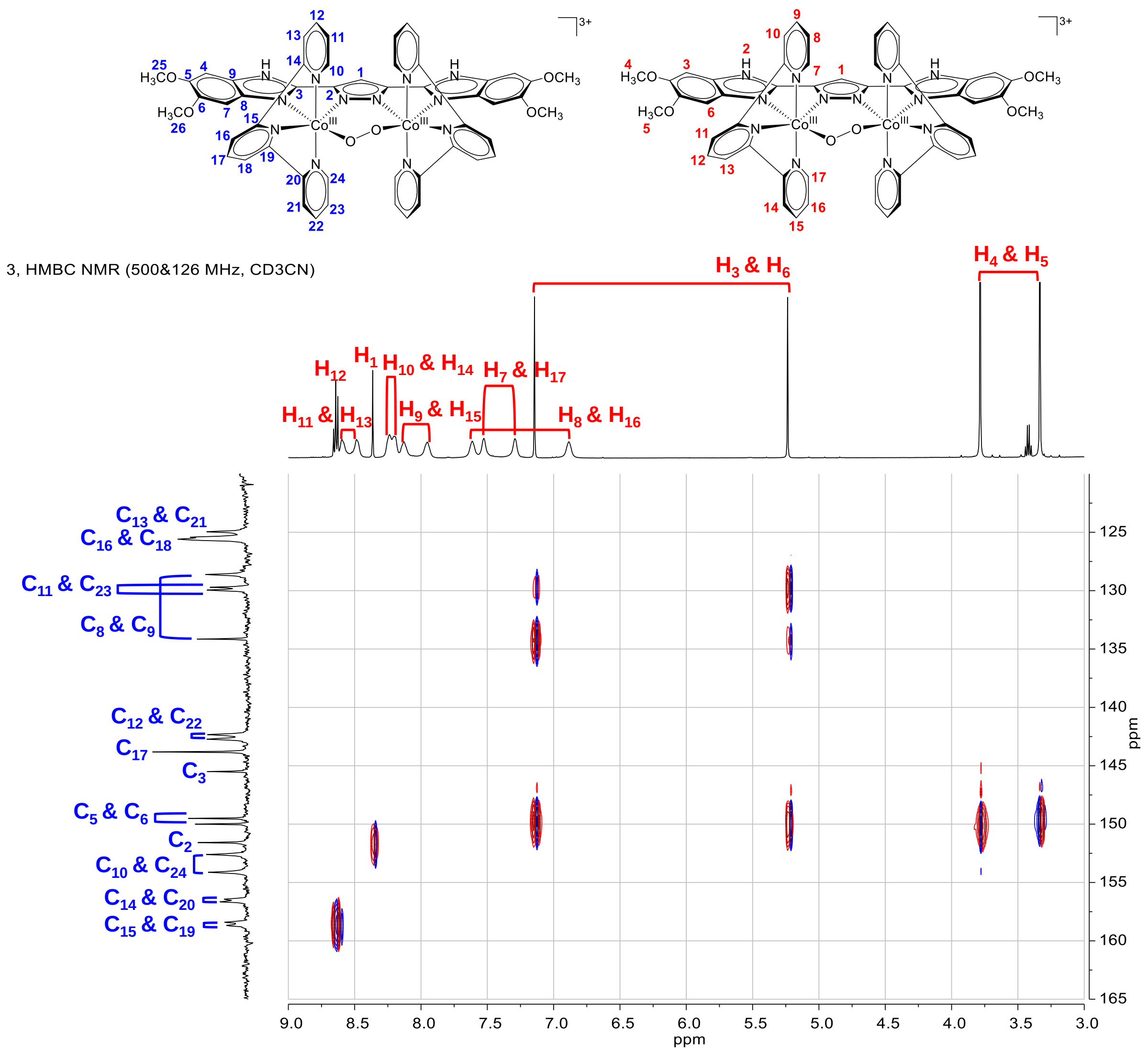

Figure S82. ^{1}H-^{13}C HMBC NMR spectrum of **3**.

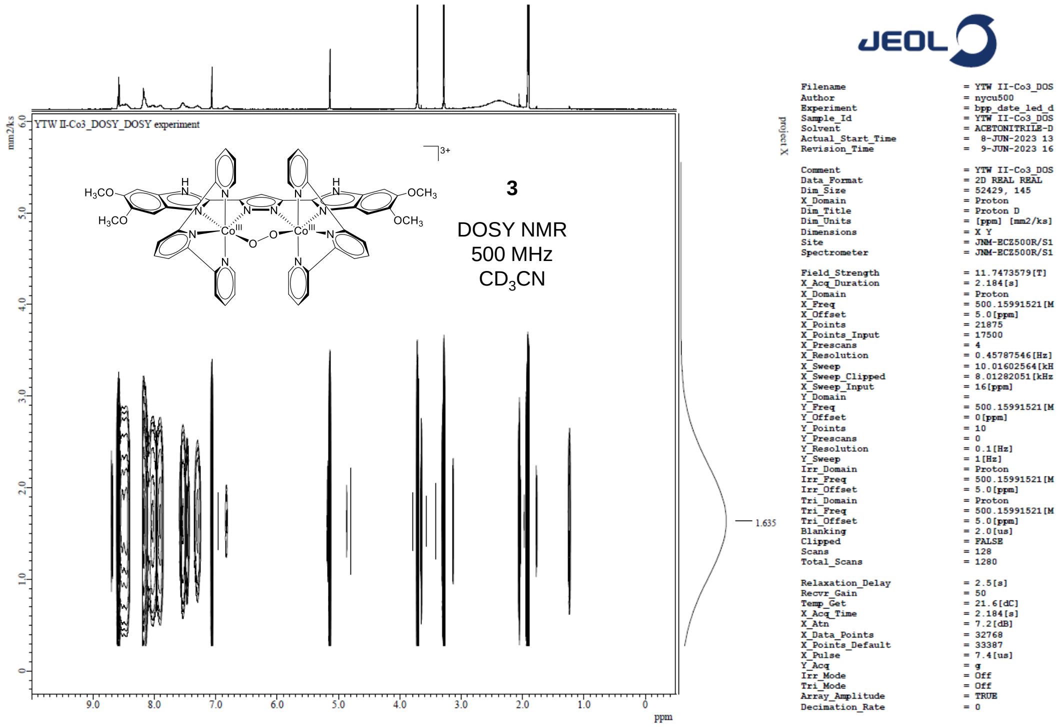

Figure S83. DOSY NMR spectrum of **3**.

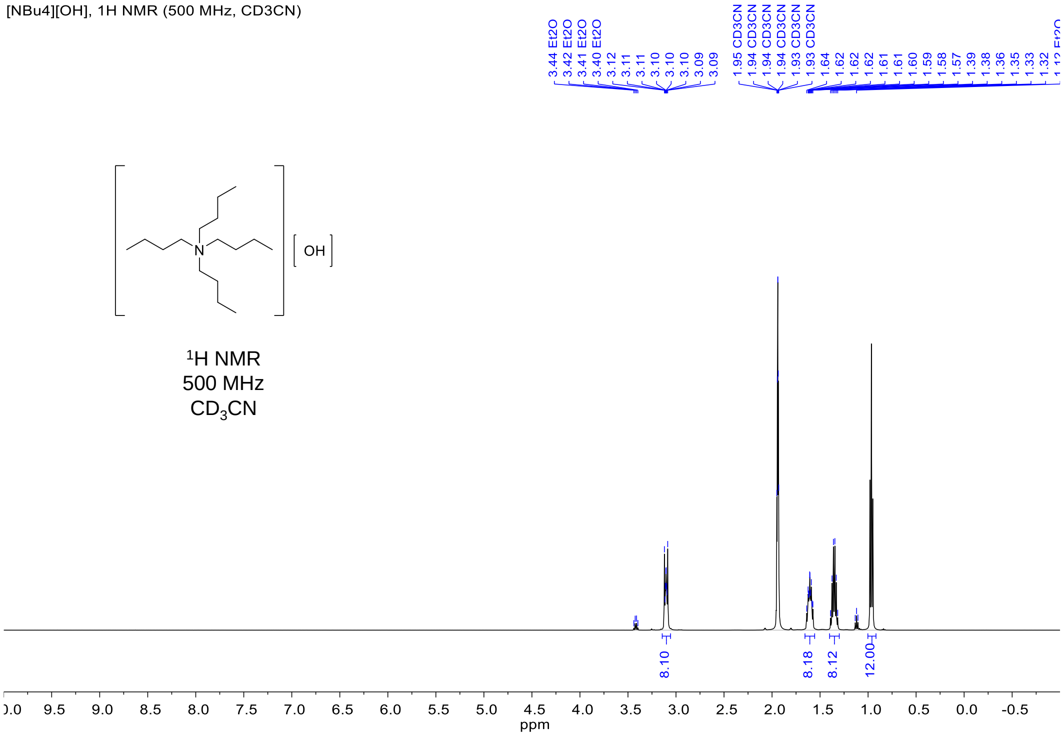

Figure S84. ^{1}H NMR spectrum of **[NBu₄][OH]**.

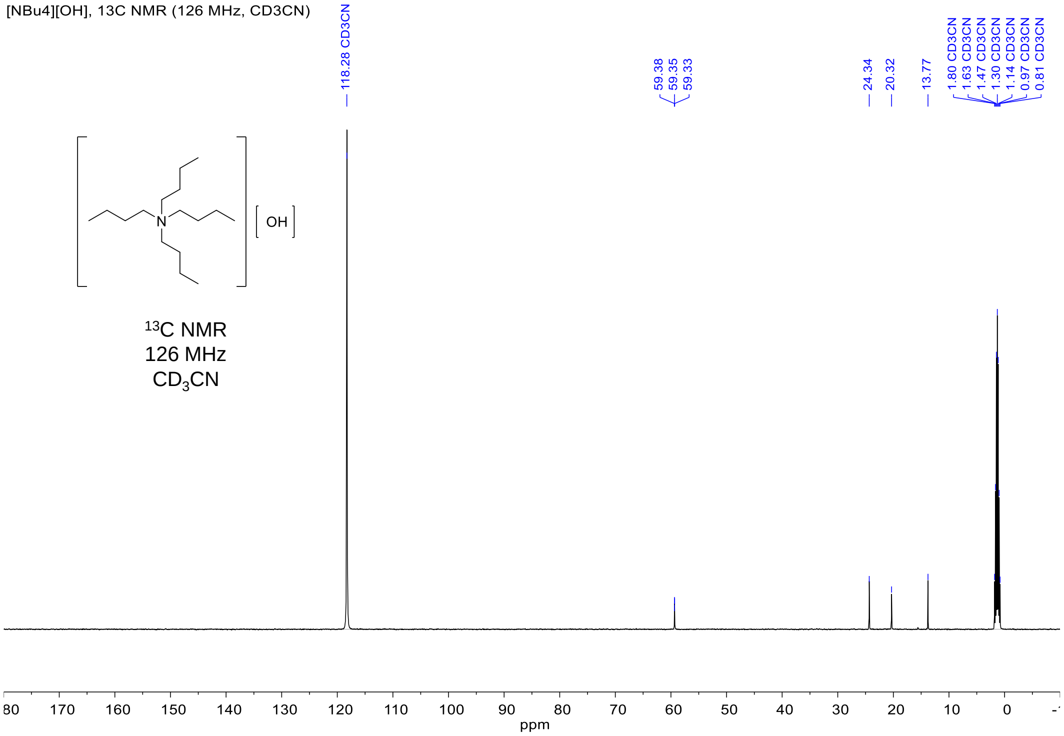

Figure S85. ^{13}C{^{1}H} NMR spectrum of **[NBu₄][OH]**.

Data:YTW I-184
Comment:
Description:
Ionization Mode:ESI+
History:Average(MS[1] 0.45..0.50)

Acquired:8/30/2022 12:25:52 PM
Operator:AccuTOF
m/z Calibration File:202200803-TFANa...
Created:8/30/2022 2:22:07 PM
Created by:AccuTOF

Charge number:1
Tolerance:300.00[ppm], 250.00 .. 250....
Unsaturation Number:-100.0 .. 200.0 (...
Element:^{12}C:16 .. 16, ^{1}H:36 .. 37, ^{14}N:1 .. 1, ^{23}Na:0 .. 1

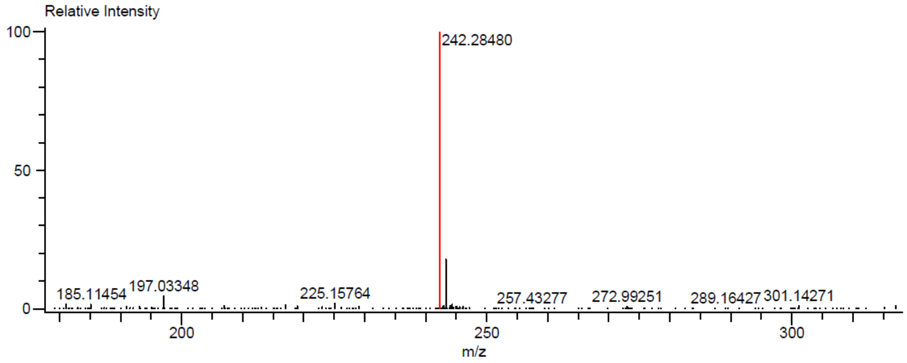

Mass	Intensity	Calc. Mass	Mass Difference [mDa]	Mass Difference [ppm]	Possible Formula
242.28480	93595.84	242.28477	0.02	0.10	$^{12}C_{16}{}^{1}H_{36}{}^{14}N_1$

Figure S86. ESI-MS of **[NBu4][OH]**.

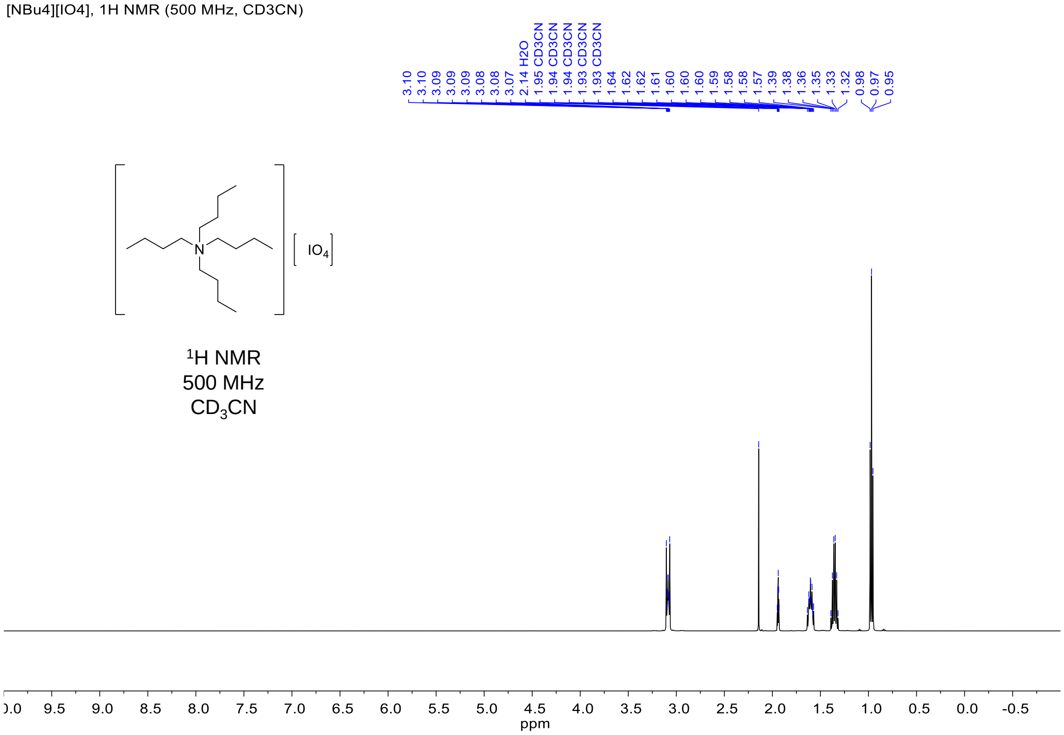

Figure S87. ^{1}H NMR spectrum of **[NBu4][IO4]**.

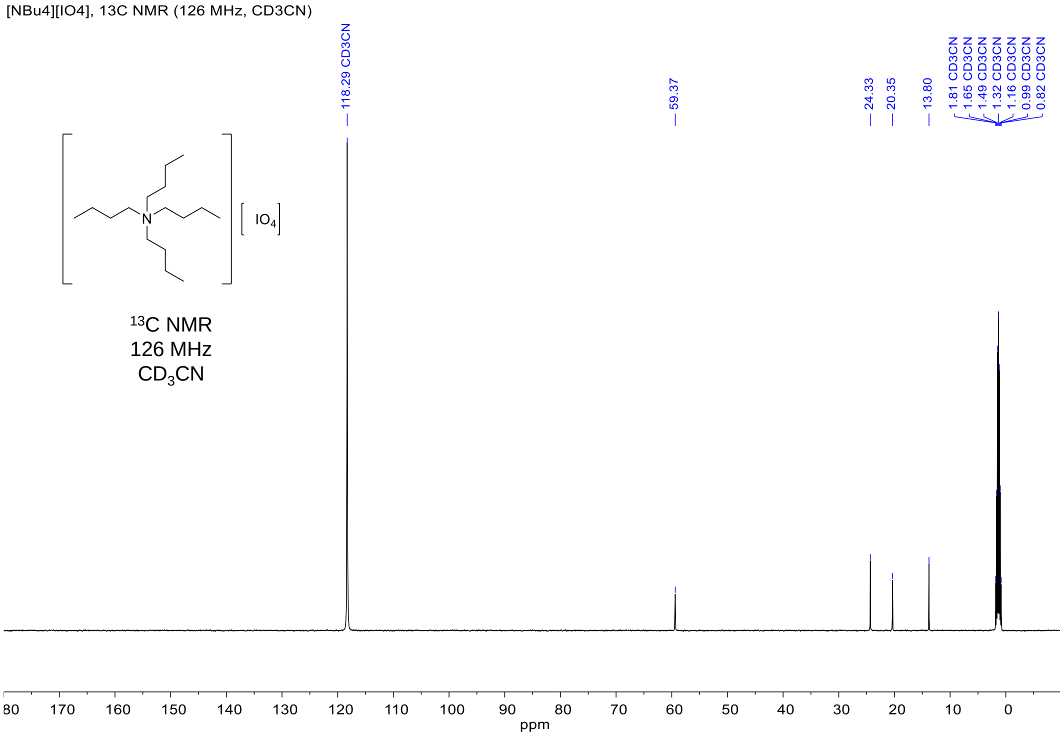

Figure S88. $^{13}C\{^{1}H\}$ NMR spectrum of **[NBu4][IO4]**.

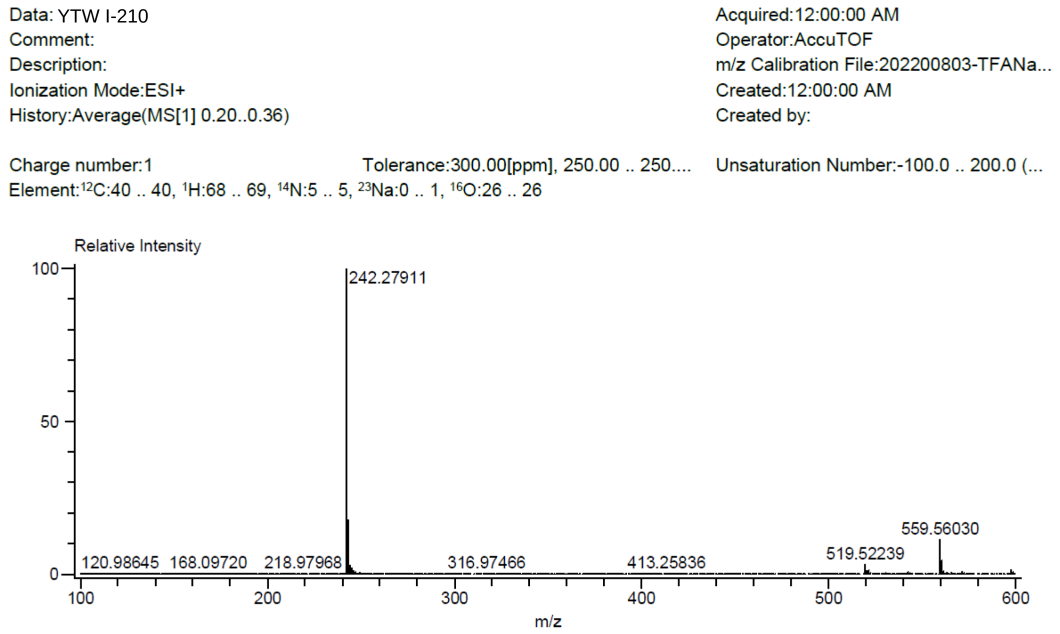

Figure S89. ESI-MS of **[NBu4][IO4]**.

Data:YTW I-210
Comment:
Description:
Ionization Mode:ESI-
History:Average(MS[1] 0.20..0.31)

Acquired:9/29/2022 2:14:31 PM
Operator:AccuTOF
m/z Calibration File:20220901-TFANa_...
Created:12:00:00 AM
Created by:

Charge number:1
Tolerance:300.00[ppm], 250.00 .. 250....
Unsaturation Number:-100.0 .. 200.0 (...
Element:^{12}C:19 .. 19, ^{1}H:0 .. 22, ^{56}Fe:1 .. 1, ^{14}N:4 .. 4, ^{23}Na:0 .. 1, ^{16}O:4 .. 4

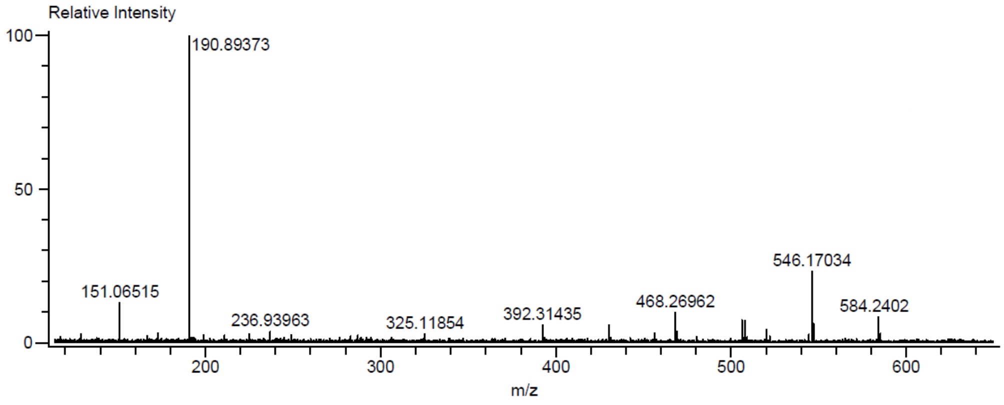

Figure S90. ESI-MS of **[NBu$_4$][IO$_4$]**.

18. Crystallographic Data

18a. Single crystal X-ray crystallography for **2**.

Data Collection

Single crystals suitable for X-ray analysis were obtained by diffusion of diethyl ether into a saturated CH_3CN solution of **2**. A red crystal with approximate dimensions 0.2 × 0.15 × 0.07 mm^3 was selected under oil under ambient conditions and attached to the tip of a MiTeGen MicroMount©. The crystal was mounted in a cold nitrogen stream at 100 K and centered in the X-ray beam using a video camera. The crystal evaluation and data collection were performed on a Bruker X8 APEX with Mo Kα (λ = 0.71073 Å) radiation and the diffractometer.

The initial cell constants were obtained from three series of ω scans at different starting angles. Each series consisted of 12 frames collected at intervals of 0.5º in a 6º range of about ω with an exposure time of 10 s per frame. The reflections were successfully indexed by an automated indexing routine built into the APEXII program suite. The final cell constants were calculated from a set of 9963 strong reflections from the actual data collection. The data were collected using the Laue symmetry data collection routine to survey the reciprocal space to the extent of a full sphere to a resolution of 0.8 Å. A total of 58192 data were harvested by collecting 3 sets of frames with 0.5º scans in ω with exposure times of 12 sec per frame. These highly redundant datasets were corrected for Lorentz and polarization effects. The absorption correction was based on fitting a function to the empirical transmission surface sampled by multiple equivalent measurements.

Structure Solution and Refinement

The systematic absences in the diffraction data were consistent for the space group $P2_1/c$ and yielded chemically reasonable and computationally stable refinement results.

A successful solution by charge-flipping provided most non-hydrogen atoms from the E-map. The remaining non-hydrogen atoms were located in an alternating series of least-squares cycles and difference Fourier maps. All non-hydrogen atoms were refined with anisotropic displacement coefficients. All hydrogen atoms were included in the structure factor calculation at idealized positions and were allowed to ride on the neighboring atoms with relative isotropic displacement coefficients.

The asymmetric unit contains one molecule of the cobalt complexes and three molecules of PF_6^-. The final least-squares refinement of 797 parameters against 14165 data resulted in residuals R (based on F2 for I≥2σ) and wR (based on F2 for I≥2σ) of 0.1185 and 0.2940, respectively.

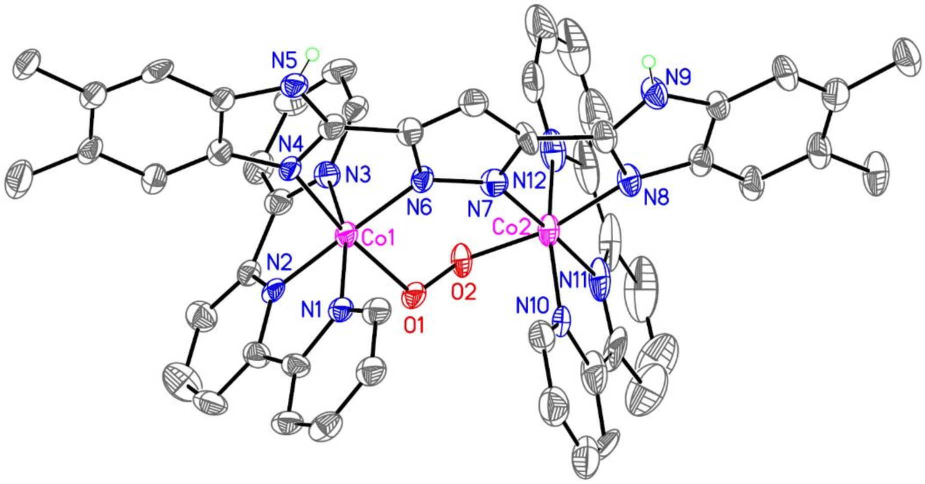

Figure S91. ORTEP drawings of **2** with thermal ellipsoids at 30% probability. Hydrogen atoms and counter ions are omitted for clarity.

Table S13. Crystal data and structure refinement for **2**.

Identification code	210332lt_0m_a_sq
Empirical formula	$C_{51}H_{41}Co_2F_{18}N_{12}O_2P_3$
Formula weight	1406.73
Temperature/K	100.15
Crystal system	monoclinic
Space group	$P2_1/c$
a/Å	13.9952(12)
b/Å	24.435(2)
c/Å	20.8554(18)
α/°	90
β/°	98.248(3)
γ/°	90
Volume/Å^3	7058.4(10)
Z	4
ρ_{calc}g/cm^3	1.324
μ/mm^{-1}	0.628
F(000)	2832.0
Crystal size/mm^3	0.2 × 0.15 × 0.07
Radiation	MoKα (λ = 0.71073)
2Θ range for data collection/°	2.582 to 52.744
Index ranges	-17 ≤ h ≤ 17, -23 ≤ k ≤ 30, -25 ≤ l ≤ 25
Reflections collected	58192
Independent reflections	14165 [R_{int} = 0.0741, R_{sigma} = 0.1065]
Data/restraints/parameters	14165/14/797
Goodness-of-fit on F^2	1.040
Final R indexes [I>=2σ (I)]	R_1 = 0.1185, wR_2 = 0.2940
Final R indexes [all data]	R_1 = 0.1913, wR_2 = 0.3373
Largest diff. peak/hole / e Å^{-3}	1.20/-0.76

Table S14. Fractional Atomic Coordinates (×10^4) and Equivalent Isotropic Displacement Parameters (Å^2×10^3) for **2**.

**Atom**	***x***	***y***	***z***	**U(eq)**
C1	1475(6)	2933(4)	5637(5)	47(2)
C2	611(7)	2615(4)	5463(5)	53(2)
C3	344(7)	2494(4)	4819(6)	56(3)
C4	873(6)	2682(4)	4380(5)	53(3)
C5	1702(6)	2970(4)	4572(4)	43(2)
C6	2337(6)	3201(4)	4140(4)	45(2)
C7	2283(7)	3125(4)	3475(5)	60(3)
C8	3004(8)	3362(6)	3170(6)	79(4)
C9	3760(7)	3655(4)	3522(5)	57(3)
C10	3771(6)	3699(3)	4180(4)	40(2)
C11	4522(6)	3977(4)	4653(5)	44(2)
C12	5304(7)	4260(4)	4485(5)	56(3)
C13	5944(7)	4496(4)	4986(5)	58(3)
C14	5771(7)	4462(4)	5611(5)	56(3)
C014	5324(6)	2276(4)	8097(5)	47(2)
C15	4958(6)	4178(3)	5738(5)	44(2)
C16	2890(7)	1990(4)	6395(5)	57(3)
C17	2279(10)	1528(6)	6229(6)	83(4)
C18	2667(11)	1064(6)	6047(6)	88(4)
C19	3634(14)	1028(4)	6001(6)	93(5)
C20	4241(9)	1503(5)	6192(6)	76(4)
C21	5263(11)	1528(5)	6216(6)	91(5)
C22	5876(13)	1125(7)	6032(9)	121(5)
C23	6748(15)	1241(8)	6051(10)	128(5)
C24	7178(10)	1728(9)	6250(9)	127(7)
C25	6562(10)	2168(7)	6477(7)	95(5)
C26	6746(7)	2732(7)	6715(6)	86(5)
C27	7616(10)	2965(9)	6849(8)	118(6)
C28	7690(9)	3496(8)	7093(8)	115(6)
C29	6892(9)	3765(7)	7190(7)	109(6)
C30	5980(8)	3506(5)	7049(6)	69(3)
C31	1946(5)	4606(3)	5248(5)	43(2)
C32	1780(6)	4748(4)	4594(5)	50(2)
C33	1236(6)	5228(4)	4420(5)	50(2)
C34	894(6)	5550(4)	4894(5)	46(2)
C35	1039(6)	5401(3)	5526(5)	51(3)
C36	1600(6)	4917(3)	5704(4)	42(2)
C37	2417(6)	4233(3)	6178(5)	43(2)
C38	2918(6)	3836(4)	6629(4)	41(2)
C39	3072(6)	3716(4)	7283(5)	50(2)
C40	3678(6)	3240(4)	7312(4)	47(2)
C41	4232(6)	2885(4)	7784(5)	48(2)
C42	5081(6)	2534(4)	8654(4)	44(2)
C43	5532(6)	2411(4)	9275(5)	55(3)
C44	6270(7)	2032(4)	9326(5)	58(3)
C45	6520(7)	1766(4)	8764(5)	57(3)
C46	6037(6)	1889(4)	8153(5)	55(3)
C47	1037(8)	5382(5)	3718(6)	76(3)
C48	339(7)	6056(4)	4717(5)	58(3)
C49	6840(8)	1922(5)	9976(5)	72(3)
C50	7337(7)	1348(5)	8837(6)	72(3)
Co1	3214.2(8)	3496.7(4)	5372.5(6)	37.4(3)
Co2	4751.4(8)	2563.0(5)	6587.8(6)	43.2(3)
N1	1998(5)	3096(3)	5207(4)	39.1(17)
N2	3075(4)	3497(2)	4467(3)	33.6(15)
N3	4349(5)	3924(3)	5269(4)	42.2(17)

Atom	*x*	*y*	*z*	U(eq)
N4	2464(5)	4172(3)	5559(3)	36.9(16)
N5	1881(5)	4668(3)	6293(4)	44.8(18)
N6	3378(5)	3480(3)	6292(3)	38.0(16)
N7	3845(5)	3121(3)	6714(4)	45.1(18)
N8	4763(5)	2496(3)	7553(3)	44.2(18)
N9	4365(5)	2916(3)	8430(4)	52(2)
N10	3840(6)	1973(3)	6375(3)	48.5(19)
N11	5613(6)	2013(4)	6434(4)	62(2)
N12	5928(5)	3000(4)	6790(4)	63(2)
O1	3849(4)	2807(2)	5346(3)	43.3(14)
O2	4728(4)	2781(2)	5698(3)	42.6(14)
F1	1375(4)	3197(3)	7997(3)	92(2)
F2	271(5)	2524(4)	7786(4)	101(3)
F3	-163(5)	3409(4)	7690(4)	110(3)
F4	-210(4)	2881(4)	6816(3)	97(3)
F5	890(5)	3547(3)	7002(4)	100(3)
F6	1341(4)	2657(3)	7115(3)	87(2)
P1	588(2)	3042.2(16)	7401.8(15)	75.6(10)
F7	4256(7)	87(4)	8057(4)	125(3)
F8	3999(10)	38(5)	7022(5)	178(5)
F9	4858(12)	750(4)	7477(5)	178(6)
F10	5424(13)	159(5)	6930(6)	201(6)
F11	5814(11)	230(9)	8051(6)	246(9)
F12	5135(8)	-511(4)	7559(5)	139(3)
P2	4946(5)	118.6(18)	7509.1(19)	116.4(17)
F13	8835(7)	798(4)	6054(6)	161(5)
F14	8716(11)	1345(6)	5150(8)	227(7)
F15	9692(9)	1547(4)	6037(6)	157(4)
F16	10305(8)	1265(4)	5155(6)	150(4)
F17	10408(7)	726(5)	5984(6)	170(5)
F18	9371(11)	532(4)	5146(6)	186(5)
P3	9547(3)	1032(2)	5570(3)	113.1(15)

Table S15. Anisotropic Displacement Parameters ($Å^2×10^3$) for **2**.

Atom	U_{11}	U_{22}	U_{33}	U_{23}	U_{13}	U_{12}
C1	38(5)	40(5)	61(6)	-6(5)	0(4)	12(4)
C2	43(5)	45(6)	69(7)	-3(5)	0(5)	10(4)
C3	38(5)	41(5)	89(8)	0(5)	8(5)	-5(4)
C4	41(5)	40(5)	71(7)	-15(5)	-11(5)	2(4)
C5	34(4)	43(5)	49(5)	-11(4)	-5(4)	3(4)
C6	42(5)	45(5)	46(5)	-6(4)	-3(4)	7(4)
C7	56(6)	61(7)	56(6)	-24(5)	-11(5)	1(5)
C8	66(7)	118(11)	57(7)	-6(7)	18(6)	-7(7)
C9	51(6)	62(7)	56(6)	-3(5)	2(5)	1(5)
C10	42(5)	30(4)	46(5)	4(4)	0(4)	4(4)
C11	43(5)	37(5)	52(6)	-6(4)	6(4)	8(4)
C12	54(6)	52(6)	64(7)	8(5)	17(5)	2(5)
C13	49(6)	54(6)	71(7)	-3(5)	11(5)	-16(5)
C14	48(5)	41(5)	77(7)	-13(5)	2(5)	-7(4)
C014	31(4)	50(5)	57(6)	13(5)	-1(4)	6(4)
C15	46(5)	32(5)	52(5)	0(4)	1(4)	7(4)
C16	53(6)	61(7)	53(6)	15(5)	-5(5)	-6(5)
C17	90(9)	84(9)	70(8)	27(7)	-2(7)	-13(8)
C18	97(11)	82(10)	80(9)	30(8)	-5(8)	17(8)
C19	188(17)	20(5)	70(8)	7(5)	11(9)	17(8)

Atom	U_{11}	U_{22}	U_{33}	U_{23}	U_{13}	U_{12}
C20	71(8)	91(10)	66(7)	21(7)	6(6)	47(7)
C21	133(12)	55(8)	86(9)	25(7)	14(8)	66(8)
C22	108(10)	93(9)	170(12)	23(9)	45(11)	52(9)
C23	118(10)	93(9)	179(13)	24(9)	38(12)	44(10)
C24	58(8)	183(18)	146(15)	79(14)	35(9)	68(10)
C25	74(9)	132(13)	84(9)	73(10)	31(7)	55(9)
C26	20(5)	158(14)	79(8)	65(9)	4(5)	19(7)
C27	45(6)	191(15)	112(10)	72(11)	-12(6)	-15(9)
C28	32(5)	195(15)	111(10)	74(11)	-17(6)	-32(9)
C29	62(8)	147(14)	104(11)	53(10)	-41(7)	-39(9)
C30	53(6)	79(8)	70(7)	39(7)	-13(5)	8(6)
C31	24(4)	37(5)	63(6)	4(4)	-11(4)	2(4)
C32	30(4)	44(5)	71(7)	18(5)	-13(4)	-12(4)
C33	38(5)	30(5)	76(7)	13(5)	-13(5)	-10(4)
C34	29(4)	35(5)	70(7)	6(5)	-8(4)	-3(4)
C35	28(4)	25(4)	93(8)	-7(5)	-8(5)	-6(3)
C36	31(4)	32(4)	58(6)	3(4)	-11(4)	-4(4)
C37	34(4)	34(5)	58(6)	-5(4)	-1(4)	9(4)
C38	28(4)	43(5)	52(5)	-1(4)	2(4)	10(4)
C39	42(5)	47(5)	57(6)	-6(5)	-1(4)	5(4)
C40	40(5)	66(6)	31(5)	6(4)	-4(4)	16(4)
C41	35(5)	58(6)	50(6)	8(5)	-1(4)	8(4)
C42	36(4)	46(5)	46(5)	6(4)	-6(4)	7(4)
C43	37(5)	77(7)	47(5)	13(5)	-6(4)	5(5)
C44	51(6)	56(6)	65(7)	16(5)	-1(5)	-4(5)
C45	42(5)	67(7)	60(6)	19(5)	-5(5)	11(5)
C46	39(5)	62(6)	62(6)	17(5)	-8(4)	7(5)
C47	72(7)	56(7)	94(9)	27(6)	-5(6)	6(6)
C48	46(5)	32(5)	89(8)	13(5)	-10(5)	-1(4)
C49	60(7)	89(9)	59(7)	18(6)	-12(5)	2(6)
C50	54(6)	87(8)	76(8)	28(6)	7(5)	37(6)
Co1	30.7(6)	32.2(6)	45.8(7)	3.5(5)	-6.4(5)	6.8(5)
Co2	34.1(6)	47.2(7)	46.4(7)	12.6(6)	-0.8(5)	15.9(5)
N1	33(4)	30(4)	51(4)	-3(3)	-5(3)	9(3)
N2	25(3)	22(3)	53(4)	2(3)	0(3)	9(3)
N3	31(4)	41(4)	51(5)	-3(3)	-6(3)	8(3)
N4	35(4)	26(4)	46(4)	3(3)	-5(3)	4(3)
N5	40(4)	33(4)	57(5)	-7(3)	-8(3)	5(3)
N6	34(4)	32(4)	46(4)	6(3)	-1(3)	6(3)
N7	33(4)	43(4)	57(5)	-4(4)	-2(3)	10(3)
N8	32(4)	56(5)	44(4)	8(4)	2(3)	16(3)
N9	42(4)	63(5)	46(5)	2(4)	-4(3)	13(4)
N10	61(5)	39(4)	41(4)	17(3)	-7(4)	11(4)
N11	49(5)	82(7)	56(5)	31(5)	9(4)	37(5)
N12	36(4)	90(7)	62(6)	29(5)	3(4)	16(4)
O1	39(3)	30(3)	57(4)	2(3)	-6(3)	10(3)
O2	30(3)	53(4)	44(3)	15(3)	2(2)	18(3)
F1	56(4)	143(7)	75(4)	28(4)	-1(3)	-3(4)
F2	76(5)	139(7)	94(5)	35(5)	24(4)	-18(4)
F3	63(4)	181(9)	87(5)	-16(5)	12(4)	24(5)
F4	48(3)	181(8)	61(4)	22(4)	8(3)	22(4)
F5	62(4)	133(7)	106(5)	69(5)	15(4)	30(4)
F6	48(3)	128(6)	85(5)	27(4)	10(3)	26(4)
P1	40.2(15)	123(3)	64.2(19)	31.4(19)	9.3(13)	11.6(16)
F7	148(8)	118(7)	109(7)	28(5)	18(6)	20(6)
F8	263(14)	142(9)	102(7)	0(6)	-67(9)	61(9)
F9	360(18)	87(7)	101(7)	-11(5)	78(9)	-8(9)
F10	345(19)	123(9)	175(11)	-24(8)	171(12)	11(10)
F11	207(13)	400(30)	137(10)	-109(14)	39(10)	-107(15)

Atom	U_{11}	U_{22}	U_{33}	U_{23}	U_{13}	U_{12}
F12	165(9)	107(7)	138(8)	54(6)	4(6)	35(6)
P2	216(5)	78(3)	53(2)	3.2(19)	10(3)	-4(3)
F13	103(7)	137(8)	254(13)	56(9)	65(8)	-9(6)
F14	244(15)	161(11)	242(15)	55(11)	-80(12)	48(11)
F15	201(11)	81(6)	201(11)	-17(7)	73(9)	-31(7)
F16	172(10)	112(7)	174(10)	33(7)	52(8)	11(7)
F17	91(6)	190(11)	235(13)	61(10)	40(7)	37(7)
F18	259(15)	93(7)	190(11)	-28(7)	-19(10)	-36(8)
P3	98(3)	95(3)	147(4)	34(3)	21(3)	-2(2)

Table S16. Bond Lengths (Å) for **2**.

Atom	Atom	Length/Å	Atom	Atom	Length/Å
C1	C2	1.439(13)	C35	C36	1.438(12)
C1	N1	1.299(11)	C36	N5	1.377(11)
C2	C3	1.373(14)	C37	C38	1.460(12)
C3	C4	1.338(14)	C37	N4	1.310(11)
C4	C5	1.368(12)	C37	N5	1.343(10)
C5	C6	1.465(13)	C38	C39	1.382(13)
C5	N1	1.364(11)	C38	N6	1.338(10)
C6	C7	1.392(13)	C39	C40	1.436(13)
C6	N2	1.362(10)	C40	C41	1.451(12)
C7	C8	1.394(15)	C40	N7	1.333(11)
C8	C9	1.396(15)	C41	N8	1.338(11)
C9	C10	1.374(13)	C41	N9	1.334(11)
C10	C11	1.497(12)	C42	C43	1.389(12)
C10	N2	1.310(10)	C42	N9	1.400(11)
C11	C12	1.382(13)	C43	C44	1.380(14)
C11	N3	1.347(11)	C44	C45	1.426(15)
C12	C13	1.399(14)	C44	C49	1.496(13)
C13	C14	1.361(14)	C45	C46	1.387(13)
C14	C15	1.391(13)	C45	C50	1.526(13)
C014	C42	1.406(13)	Co1	N1	1.951(7)
C014	C46	1.366(12)	Co1	N2	1.870(7)
C014	N8	1.392(11)	Co1	N3	1.938(7)
C15	N3	1.353(11)	Co1	N4	2.024(7)
C16	C17	1.431(16)	Co1	N6	1.899(7)
C16	N10	1.336(12)	Co1	O1	1.909(5)
C17	C18	1.333(18)	Co2	N7	1.906(7)
C18	C19	1.374(19)	Co2	N8	2.018(7)
C19	C20	1.460(19)	Co2	N10	1.934(8)
C20	C21	1.425(19)	Co2	N11	1.863(8)
C20	N10	1.357(14)	Co2	N12	1.957(9)
C21	C22	1.394(17)	Co2	O2	1.926(6)
C21	N11	1.338(17)	N6	N7	1.344(9)
C22	C23	1.25(2)	O1	O2	1.340(7)
C23	C24	1.37(3)	F1	P1	1.583(7)
C24	C25	1.50(2)	F2	P1	1.595(8)
C25	C26	1.48(2)	F3	P1	1.566(8)
C25	N11	1.372(17)	F4	P1	1.581(7)
C26	C27	1.337(18)	F5	P1	1.580(8)
C26	N12	1.347(14)	F6	P1	1.592(7)
C27	C28	1.39(2)	F7	P2	1.600(10)
C28	C29	1.34(2)	F8	P2	1.563(12)
C29	C30	1.418(16)	F9	P2	1.549(11)
C30	N12	1.347(15)	F10	P2	1.465(11)
C31	C32	1.393(13)	F11	P2	1.561(14)
C31	C36	1.359(13)	F12	P2	1.562(10)
C31	N4	1.391(10)	F13	P3	1.622(10)

Atom	Atom	Length/Å	Atom	Atom	Length/Å
C32	C33	1.418(12)	F14	P3	1.552(12)
C33	C34	1.401(14)	F15	P3	1.586(11)
C33	C47	1.498(14)	F16	P3	1.568(11)
C34	C35	1.354(13)	F17	P3	1.568(11)
C34	C48	1.479(12)	F18	P3	1.508(11)

Table S17. Bond Angles (˚) for **2**.

Atom	Atom	Atom	Angle/˚	Atom	Atom	Atom	Angle/˚
N1	C1	C2	121.9(9)	N6	Co1	O1	91.2(3)
C3	C2	C1	117.6(10)	O1	Co1	N1	87.1(3)
C4	C3	C2	119.7(9)	O1	Co1	N3	94.6(3)
C3	C4	C5	120.3(10)	O1	Co1	N4	169.0(3)
C4	C5	C6	125.6(9)	N7	Co2	N8	80.2(3)
N1	C5	C4	121.8(9)	N7	Co2	N10	97.7(3)
N1	C5	C6	112.5(7)	N7	Co2	N12	97.8(4)
C7	C6	C5	127.6(8)	N7	Co2	O2	90.9(3)
N2	C6	C5	112.4(7)	N10	Co2	N8	94.6(3)
N2	C6	C7	119.9(9)	N10	Co2	N12	164.4(4)
C6	C7	C8	117.4(9)	N11	Co2	N7	177.9(4)
C7	C8	C9	121.2(10)	N11	Co2	N8	101.6(3)
C10	C9	C8	117.6(10)	N11	Co2	N10	81.1(4)
C9	C10	C11	126.8(9)	N11	Co2	N12	83.4(4)
N2	C10	C9	121.7(8)	N11	Co2	O2	87.4(3)
N2	C10	C11	111.6(8)	N12	Co2	N8	86.8(3)
C12	C11	C10	124.6(9)	O2	Co2	N8	168.6(3)
N3	C11	C10	112.2(8)	O2	Co2	N10	93.7(3)
N3	C11	C12	123.2(8)	O2	Co2	N12	87.4(3)
C11	C12	C13	117.5(9)	C1	N1	C5	118.6(8)
C14	C13	C12	120.5(9)	C1	N1	Co1	126.5(6)
C13	C14	C15	118.3(9)	C5	N1	Co1	114.9(6)
C46	C014	C42	120.0(8)	C6	N2	Co1	117.8(6)
C46	C014	N8	131.1(9)	C10	N2	C6	122.1(8)
N8	C014	C42	108.9(7)	C10	N2	Co1	119.0(5)
N3	C15	C14	122.7(9)	C11	N3	C15	117.7(8)
N10	C16	C17	122.0(11)	C11	N3	Co1	114.9(6)
C18	C17	C16	119.2(14)	C15	N3	Co1	127.4(7)
C17	C18	C19	121.3(14)	C31	N4	Co1	141.4(6)
C18	C19	C20	118.0(12)	C37	N4	C31	106.0(7)
C21	C20	C19	125.8(12)	C37	N4	Co1	112.5(5)
N10	C20	C19	120.1(11)	C37	N5	C36	106.5(8)
N10	C20	C21	114.1(12)	C38	N6	Co1	121.2(5)
C22	C21	C20	127.9(16)	C38	N6	N7	107.8(7)
N11	C21	C20	111.4(10)	N7	N6	Co1	130.7(6)
N11	C21	C22	120.7(15)	C40	N7	Co2	119.7(6)
C23	C22	C21	118(2)	C40	N7	N6	109.6(7)
C22	C23	C24	126.2(19)	N6	N7	Co2	130.3(6)
C23	C24	C25	118.1(15)	C014	N8	Co2	140.1(6)
C26	C25	C24	134.2(14)	C41	N8	C014	105.0(7)
N11	C25	C24	112.2(16)	C41	N8	Co2	112.1(6)
N11	C25	C26	113.5(11)	C41	N9	C42	106.8(8)
C27	C26	C25	125.3(15)	C16	N10	C20	119.3(10)
C27	C26	N12	122.2(18)	C16	N10	Co2	126.5(7)
N12	C26	C25	112.5(11)	C20	N10	Co2	114.2(8)
C26	C27	C28	119.5(17)	C21	N11	C25	124.7(12)
C29	C28	C27	119.6(14)	C21	N11	Co2	118.9(8)
C28	C29	C30	119.7(16)	C25	N11	Co2	115.8(10)
N12	C30	C29	119.3(13)	C26	N12	Co2	114.4(10)
C36	C31	C32	121.3(8)	C30	N12	C26	119.6(11)

Atom	Atom	Atom	Angle/˚	Atom	Atom	Atom	Angle/˚
C36	C31	N4	108.3(8)	C30	N12	Co2	125.6(7)
N4	C31	C32	130.5(9)	O2	O1	Co1	114.9(4)
C31	C32	C33	117.7(10)	O1	O2	Co2	115.3(4)
C32	C33	C47	118.5(10)	F1	P1	F2	90.5(4)
C34	C33	C32	120.6(9)	F1	P1	F6	90.7(4)
C34	C33	C47	120.9(8)	F3	P1	F1	89.5(4)
C33	C34	C48	121.0(9)	F3	P1	F2	90.6(5)
C35	C34	C33	121.1(8)	F3	P1	F4	90.0(4)
C35	C34	C48	117.9(9)	F3	P1	F5	90.1(5)
C34	C35	C36	118.2(9)	F3	P1	F6	178.7(5)
C31	C36	C35	121.0(9)	F4	P1	F1	178.9(4)
C31	C36	N5	107.1(7)	F4	P1	F2	88.5(4)
N5	C36	C35	131.9(9)	F4	P1	F6	89.7(4)
N4	C37	C38	118.1(7)	F5	P1	F1	91.0(4)
N4	C37	N5	112.1(8)	F5	P1	F2	178.3(5)
N5	C37	C38	129.8(9)	F5	P1	F4	89.9(4)
C39	C38	C37	140.3(8)	F5	P1	F6	91.2(4)
N6	C38	C37	108.5(8)	F6	P1	F2	88.1(4)
N6	C38	C39	111.3(7)	F8	P2	F7	85.2(7)
C38	C39	C40	102.6(8)	F9	P2	F7	91.5(6)
C39	C40	C41	140.1(9)	F9	P2	F8	92.4(8)
N7	C40	C39	108.7(7)	F9	P2	F11	84.5(9)
N7	C40	C41	110.9(8)	F9	P2	F12	174.8(9)
N8	C41	C40	116.8(8)	F10	P2	F7	170.2(9)
N9	C41	C40	129.4(9)	F10	P2	F8	85.2(8)
N9	C41	N8	113.5(8)	F10	P2	F9	86.6(6)
C43	C42	C014	122.8(8)	F10	P2	F11	100.7(10)
C43	C42	N9	131.5(9)	F10	P2	F12	91.6(6)
N9	C42	C014	105.6(7)	F11	P2	F7	88.7(6)
C44	C43	C42	116.8(10)	F11	P2	F8	173.1(8)
C43	C44	C45	120.8(9)	F11	P2	F12	91.0(9)
C43	C44	C49	119.1(10)	F12	P2	F7	91.0(6)
C45	C44	C49	120.0(9)	F12	P2	F8	92.3(6)
C44	C45	C50	119.5(9)	F14	P3	F13	92.7(8)
C46	C45	C44	120.7(9)	F14	P3	F15	88.6(8)
C46	C45	C50	119.7(10)	F14	P3	F16	91.3(8)
C014	C46	C45	118.8(10)	F14	P3	F17	178.4(9)
N1	Co1	N4	88.9(3)	F15	P3	F13	86.1(6)
N2	Co1	N1	81.9(3)	F16	P3	F13	174.9(8)
N2	Co1	N3	81.7(3)	F16	P3	F15	90.8(6)
N2	Co1	N4	102.2(3)	F16	P3	F17	87.1(6)
N2	Co1	N6	178.5(3)	F17	P3	F13	88.9(6)
N2	Co1	O1	87.3(3)	F17	P3	F15	91.1(8)
N3	Co1	N1	163.4(3)	F18	P3	F13	90.9(7)
N3	Co1	N4	92.2(3)	F18	P3	F14	91.3(8)
N6	Co1	N1	98.3(3)	F18	P3	F15	177.0(8)
N6	Co1	N3	98.1(3)	F18	P3	F16	92.2(8)
N6	Co1	N4	79.3(3)	F18	P3	F17	89.0(8)

Table S18. Torsion Angles (˚) for **2**.

A	B	C	D	Angle/˚	A	B	C	D	Angle/˚
C1	C2	C3	C4	-1.4(13)	C38	C37	N5	C36	178.1(8)
C2	C1	N1	C5	1.4(12)	C38	C39	C40	C41	173.0(12)
C2	C1	N1	Co1	-176.8(6)	C38	C39	C40	N7	0.9(10)
C2	C3	C4	C5	3.1(14)	C38	N6	N7	C40	0.7(9)
C3	C4	C5	C6	-179.4(9)	C38	N6	N7	Co2	-171.9(6)
C3	C4	C5	N1	-2.6(14)	C39	C38	N6	Co1	174.3(6)
C4	C5	C6	C7	-7.4(15)	C39	C38	N6	N7	-0.1(10)

A	B	C	D	Angle/˚	A	B	C	D	Angle/˚
C4	C5	C6	N2	176.1(8)	C39	C40	C41	N8	-174.8(11)
C4	C5	N1	C1	0.3(12)	C39	C40	C41	N9	-2(2)
C4	C5	N1	Co1	178.7(7)	C39	C40	N7	Co2	172.5(6)
C5	C6	C7	C8	-176.4(10)	C39	C40	N7	N6	-1.0(10)
C5	C6	N2	C10	174.1(7)	C40	C41	N8	C014	171.1(8)
C5	C6	N2	Co1	5.9(9)	C40	C41	N8	Co2	6.0(10)
C6	C5	N1	C1	177.4(7)	C40	C41	N9	C42	-170.4(9)
C6	C5	N1	Co1	-4.1(9)	C41	C40	N7	Co2	-2.1(11)
C6	C7	C8	C9	1.2(17)	C41	C40	N7	N6	-175.6(7)
C7	C6	N2	C10	-2.8(12)	C42	C014	C46	C45	-1.0(14)
C7	C6	N2	Co1	-170.9(7)	C42	C014	N8	C41	2.0(10)
C7	C8	C9	C10	0.4(17)	C42	C014	N8	Co2	160.3(8)
C8	C9	C10	C11	177.9(10)	C42	C43	C44	C45	-2.6(14)
C8	C9	C10	N2	-3.2(14)	C42	C43	C44	C49	174.6(9)
C9	C10	C11	C12	3.7(14)	C43	C42	N9	C41	176.5(10)
C9	C10	C11	N3	-176.7(8)	C43	C44	C45	C46	1.2(16)
C9	C10	N2	C6	4.5(12)	C43	C44	C45	C50	-179.8(10)
C9	C10	N2	Co1	172.5(7)	C44	C45	C46	C014	0.7(15)
C10	C11	C12	C13	-179.9(8)	C46	C014	C42	C43	-0.5(14)
C10	C11	N3	C15	-177.2(7)	C46	C014	C42	N9	177.7(8)
C10	C11	N3	Co1	1.4(9)	C46	C014	N8	C41	-175.7(10)
C11	C10	N2	C6	-176.4(7)	C46	C014	N8	Co2	-17.5(17)
C11	C10	N2	Co1	-8.5(9)	C47	C33	C34	C35	177.3(8)
C11	C12	C13	C14	-2.5(15)	C47	C33	C34	C48	-1.0(13)
C12	C11	N3	C15	2.3(12)	C48	C34	C35	C36	-178.3(7)
C12	C11	N3	Co1	-179.1(7)	C49	C44	C45	C46	-176.0(10)
C12	C13	C14	C15	1.3(15)	C49	C44	C45	C50	3.1(15)
C13	C14	C15	N3	1.8(14)	C50	C45	C46	C014	-178.3(9)
C14	C15	N3	C11	-3.6(12)	Co1	N6	N7	C40	-173.0(6)
C14	C15	N3	Co1	178.0(6)	Co1	N6	N7	Co2	14.5(11)
C014	C42	C43	C44	2.3(14)	Co1	O1	O2	Co2	88.6(5)
C014	C42	N9	C41	-1.5(10)	N1	C1	C2	C3	-0.9(13)
C16	C17	C18	C19	-1.8(19)	N1	C5	C6	C7	175.6(9)
C17	C16	N10	C20	-0.1(14)	N1	C5	C6	N2	-0.9(10)
C17	C16	N10	Co2	-179.8(7)	N1	Co1	N2	C6	-6.5(6)
C17	C18	C19	C20	3.5(19)	N1	Co1	N2	C10	-175.0(6)
C18	C19	C20	C21	175.7(12)	N1	Co1	N6	C38	-81.2(7)
C18	C19	C20	N10	-3.5(17)	N1	Co1	N6	N7	91.8(7)
C19	C20	C21	C22	4(2)	N2	C6	C7	C8	-0.1(14)
C19	C20	C21	N11	-177.6(10)	N2	C10	C11	C12	-175.2(8)
C19	C20	N10	C16	1.9(14)	N2	C10	C11	N3	4.3(10)
C19	C20	N10	Co2	-178.4(8)	N3	C11	C12	C13	0.6(14)
C20	C21	C22	C23	175.3(16)	N3	Co1	N2	C6	176.0(6)
C20	C21	N11	C25	-176.4(10)	N3	Co1	N2	C10	7.5(6)
C20	C21	N11	Co2	-5.2(13)	N3	Co1	N6	C38	96.7(6)
C21	C20	N10	C16	-177.4(9)	N3	Co1	N6	N7	-90.3(7)
C21	C20	N10	Co2	2.3(12)	N4	C31	C32	C33	-178.3(8)
C21	C22	C23	C24	2(3)	N4	C31	C36	C35	179.4(7)
C22	C21	N11	C25	2.3(18)	N4	C31	C36	N5	-1.6(9)
C22	C21	N11	Co2	173.4(10)	N4	C37	C38	C39	-178.6(10)
C22	C23	C24	C25	1(3)	N4	C37	C38	N6	2.2(11)
C23	C24	C25	C26	-179.2(15)	N4	C37	N5	C36	-2.7(9)
C23	C24	C25	N11	-1.7(19)	N4	Co1	N2	C6	-93.6(6)
C24	C25	C26	C27	-8(2)	N4	Co1	N2	C10	97.9(6)
C24	C25	C26	N12	172.2(12)	N4	Co1	N6	C38	6.1(6)
C24	C25	N11	C21	0.1(15)	N4	Co1	N6	N7	179.0(7)
C24	C25	N11	Co2	-171.3(8)	N5	C37	C38	C39	0.6(18)
C25	C26	C27	C28	-177.7(13)	N5	C37	C38	N6	-178.6(8)
C25	C26	N12	C30	175.1(10)	N5	C37	N4	C31	1.7(9)

A	B	C	D	Angle/˚	A	B	C	D	Angle/˚
C25	C26	N12	Co2	1.6(12)	N5	C37	N4	Co1	-177.2(5)
C26	C25	N11	C21	178.2(10)	N6	C38	C39	C40	-0.5(10)
C26	C25	N11	Co2	6.8(12)	N7	C40	C41	N8	-2.9(12)
C26	C27	C28	C29	0(2)	N7	C40	C41	N9	170.3(9)
C27	C26	N12	C30	-4.6(17)	N8	C014	C42	C43	-178.5(8)
C27	C26	N12	Co2	-178.1(10)	N8	C014	C42	N9	-0.4(10)
C27	C28	C29	C30	1(2)	N8	C014	C46	C45	176.5(10)
C28	C29	C30	N12	-3.2(18)	N8	C41	N9	C42	3.0(11)
C29	C30	N12	C26	5.2(15)	N8	Co2	N11	C21	98.1(8)
C29	C30	N12	Co2	177.8(8)	N8	Co2	N11	C25	-90.0(8)
C31	C32	C33	C34	1.0(12)	N9	C41	N8	C014	-3.2(11)
C31	C32	C33	C47	-179.0(8)	N9	C41	N8	Co2	-168.3(6)
C31	C36	N5	C37	2.6(9)	N9	C42	C43	C44	-175.3(9)
C32	C31	C36	C35	0.8(12)	N10	C16	C17	C18	0.0(16)
C32	C31	C36	N5	179.8(7)	N10	C20	C21	C22	-176.9(13)
C32	C31	N4	C37	178.4(9)	N10	C20	C21	N11	1.7(14)
C32	C31	N4	Co1	-3.4(15)	N10	Co2	N11	C21	5.2(8)
C32	C33	C34	C35	-2.7(12)	N10	Co2	N11	C25	177.1(8)
C32	C33	C34	C48	179.0(8)	N11	C21	C22	C23	-3(2)
C33	C34	C35	C36	3.3(12)	N11	C25	C26	C27	174.4(11)
C34	C35	C36	C31	-2.4(12)	N11	C25	C26	N12	-5.4(14)
C34	C35	C36	N5	178.9(8)	N12	C26	C27	C28	2(2)
C35	C36	N5	C37	-178.6(8)	N12	Co2	N11	C21	-176.6(9)
C36	C31	C32	C33	-0.2(12)	N12	Co2	N11	C25	-4.7(8)
C36	C31	N4	C37	0.0(9)	O1	Co1	N2	C6	80.9(6)
C36	C31	N4	Co1	178.3(6)	O1	Co1	N2	C10	-87.6(6)
C37	C38	C39	C40	-179.6(11)	O1	Co1	N6	C38	-168.4(6)
C37	C38	N6	Co1	-6.3(9)	O1	Co1	N6	N7	4.5(7)
C37	C38	N6	N7	179.4(7)	O2	Co2	N11	C21	-89.0(8)
C38	C37	N4	C31	-179.0(7)	O2	Co2	N11	C25	82.9(8)
C38	C37	N4	Co1	2.2(10)					

Table S19. Hydrogen Atom Coordinates (Å×10^4) and Isotropic Displacement Parameters (Å^2×10^3) for **2**.

Atom	*x*	*y*	*z*	U(eq)
H1	1668.6	3026.09	6078.36	57
H2	238.6	2493.76	5781.87	64
H3	-213.73	2278.31	4685.78	67
H4	672.23	2613.71	3932.37	63
H7	1774.32	2919.13	3237.54	72
H8	2981.16	3323.04	2715.26	95
H9	4250.23	3818.65	3314.31	68
H12	5403.52	4294.01	4045.66	67
H13	6502.61	4681.7	4890	69
H14	6195.96	4628.48	5952.19	67
H15	4823.69	4162.7	6171.33	53
H16	2612.91	2320.21	6524.36	68
H17	1606.21	1549.97	6247.2	99
H18	2265.83	752.86	5948.47	106
H19	3899.9	703	5848.98	112
H22	5636.55	773.28	5897.57	145
H23	7152.99	964.72	5914.15	154
H24	7848.21	1785.34	6243.03	152
H27	8178.62	2770.32	6779.15	142
H28	8304.77	3665.47	7189.28	139
H29	6936.24	4129.14	7352.31	131
H30	5411.86	3686.52	7137.18	83

Atom	*x*	*y*	*z*	U(eq)
H32	2023.91	4530.61	4276.47	61
H35	777.48	5610	5842.75	61
H39	2835.82	3902.66	7628.31	60
H43	5341.72	2580.38	9646.64	66
H46	6199.48	1708.68	7780.27	67
H47A	337.63	5396.27	3580.26	113
H47B	1323.8	5109.1	3459.16	113
H47C	1318.44	5742.08	3655.58	113
H48A	47.29	6184.38	5089.91	86
H48B	-169.44	5981.5	4353.79	86
H48C	773.29	6338.95	4590.97	86
H49A	6863.22	1526.67	10056.82	108
H49B	6532.98	2104.67	10311.55	108
H49C	7498.48	2061.99	9985.95	108
H50A	7912.78	1507.27	9091.37	108
H50B	7484.3	1245.31	8406.97	108
H50C	7139.99	1021.64	9057.83	108
H5	1737.75	4774.17	6671.51	54
H9A	4061.91	3134.5	8668.37	62

Table S20. Solvent masks information for **2**.

Number	X	Y	Z	Volume	Electron count	Content
1	-0.025	0.473	0.103	2057	658	

18b. Single crystal X-ray crystallography for **3**.

Data Collection

Single crystals suitable for X-ray analysis were obtained by diffusion of diethyl ether into a saturated CH_3CN solution of **3**. A dark red crystal with approximate dimensions 0.26 × 0.16 × 0.11 mm^3 was selected under oil under ambient conditions and attached to the tip of a MiTeGen MicroMount©. The crystal was mounted in a cold nitrogen stream at 100 K and centered in the X-ray beam using a video camera. The crystal evaluation and data collection were performed on a Rigaku XtaLAB Synergy R, DW system, HyPix-Arc 150 with Cu Kα (λ = 1.54184 Å) radiation and the diffractometer.

The initial cell constants were obtained from ω scans consisting of 12 frames collected at intervals of 0.5º in a 10º range of about ω with an exposure time of 0.5 s per frame. The reflections were successfully indexed by an automated indexing routine built into the CrysAlisPro program suite. The final cell constants were calculated from a set of 21299 strong reflections from the actual data collection. The data were collected using a Laue symmetry data collection routine to survey the reciprocal space to the extent of a full sphere to a resolution of 0.84 Å. A total of 60189 data were harvested by collecting 27 sets of frames with 0.5º scans in ω and φ with exposure times of 0.34 sec per frame. These highly redundant datasets were corrected for Lorentz and polarization effects. The absorption correction was based on fitting a function to the empirical transmission surface sampled by multiple equivalent measurements.

Structure Solution and Refinement

The systematic absences in the diffraction data were consistent for the space group $P2_1/c$ and yielded chemically reasonable and computationally stable results of refinement.

A successful solution by charge-flipping provided most non-hydrogen atoms from the E-map. The remaining non-hydrogen atoms were located in an alternating series of least-squares cycles and difference Fourier maps. All non-hydrogen atoms were refined with anisotropic displacement coefficients. All hydrogen atoms were included in the structure factor calculation at idealized positions and were allowed to ride on the neighboring atoms with relative isotropic displacement coefficients.

The asymmetric unit contains one molecule of the cobalt complex and one molecule of PF_6^-, KPF_6, and DMSO, respectively. The final least-squares refinement of 943 parameters against 13331 data resulted in residuals R (based on F2 for I≥2σ) and wR (based on F2 for I≥2σ) of 0.0809 and 0.2351, respectively.

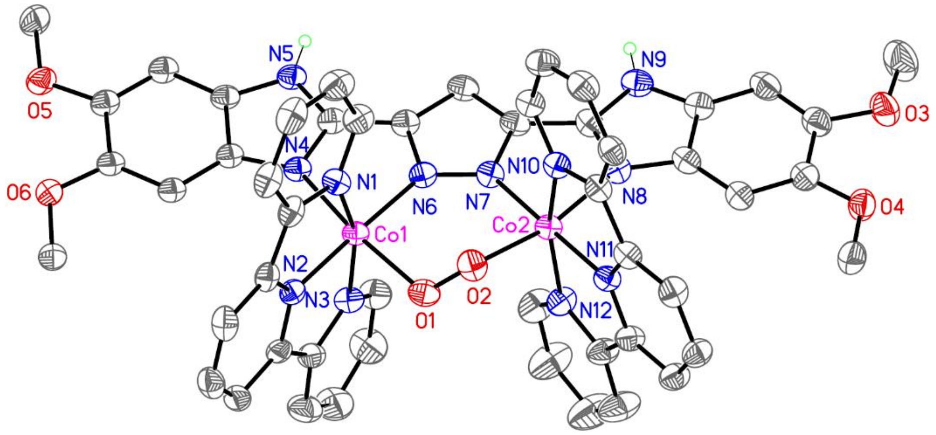

Figure S92. ORTEP drawings of **3** with thermal ellipsoids at 30% probability. Hydrogen atoms and counter ions are omitted for clarity.

Table S21. Crystal data and structure refinement for **3**.

Identification code	220654lt_auto
Empirical formula	$C_{53}H_{47}Co_2F_{24}KN_{12}O_7P_4S$
Formula weight	1732.92
Temperature/K	100.00(10)
Crystal system	monoclinic
Space group	$P2_1/c$
a/Å	18.0725(3)
b/Å	17.1834(3)
c/Å	24.5341(5)
α/°	90
β/°	100.372(2)
γ/°	90
Volume/Å^3	7494.5(2)
Z	4
ρ_{calc}g/cm^3	1.536
μ/mm^{-1}	6.065
F(000)	3480.0
Crystal size/mm^3	0.26 × 0.16 × 0.11
Radiation	Cu Kα (λ = 1.54184)
2Θ range for data collection/°	4.97 to 134.144
Index ranges	-21 ≤ h ≤ 13, -20 ≤ k ≤ 18, -29 ≤ l ≤ 29
Reflections collected	60189
Independent reflections	13331 [R_{int} = 0.0394, R_{sigma} = 0.0339]
Data/restraints/parameters	13331/0/943
Goodness-of-fit on F^2	1.068
Final R indexes [I>=2σ (I)]	R_1 = 0.0809, wR_2 = 0.2351
Final R indexes [all data]	R_1 = 0.0946, wR_2 = 0.2466
Largest diff. peak/hole / e Å^{-3}	1.26/-1.19

Table S22. Fractional Atomic Coordinates ($\times10^4$) and Equivalent Isotropic Displacement Parameters ($Å^2\times10^3$) for **3**.

Atom	*x*	*y*	*z*	U(eq)
C1	6154(4)	9534(4)	4856(3)	72.5(18)
C2	5584(5)	9804(5)	4452(4)	95(3)
C3	5395(5)	10576(5)	4424(4)	108(3)
C4	5794(5)	11083(4)	4811(3)	88(2)
C5	6371(4)	10792(3)	5200(2)	58.6(14)
C6	6810(3)	11248(3)	5642(2)	52.5(13)
C7	6753(4)	12025(3)	5752(2)	57.9(14)
C8	7205(3)	12342(3)	6217(2)	57.3(14)
C9	7719(3)	11873(3)	6556(3)	55.5(14)
C10	7768(3)	11095(3)	6427(2)	46.0(11)
C11	8244(3)	10489(3)	6738(2)	46.1(11)
C12	8743(3)	10613(4)	7225(2)	53.2(13)
C13	9128(3)	9972(4)	7490(2)	61.6(15)
C14	8992(3)	9246(4)	7258(3)	63.2(15)
C15	8492(3)	9162(3)	6764(2)	51.2(12)
C16	6575(3)	8587(3)	6297(2)	47.9(12)
C17	6179(3)	9639(3)	6647(2)	48.3(12)
C18	6021(3)	10403(3)	6791(2)	49.8(12)
C19	5514(3)	10509(4)	7141(2)	52.5(13)
C20	5165(3)	9852(4)	7346(2)	58.9(15)
C21	5325(3)	9105(4)	7217(2)	60.2(15)
C22	5840(3)	9000(3)	6859(2)	51.8(13)
C23	4225(5)	9435(6)	7851(4)	102(3)
C24	5682(4)	11887(4)	7154(3)	67.5(16)
C25	7009(3)	8155(3)	5942(2)	47.8(12)
C26	7152(3)	7395(3)	5801(2)	50.5(12)
C27	7625(3)	7491(3)	5413(2)	46.8(12)
C28	8041(3)	7033(3)	5072(2)	46.9(12)
C29	8615(3)	6140(3)	4658(2)	46.1(11)
C30	8921(3)	5473(3)	4475(2)	49.8(12)
C31	9407(3)	5553(3)	4098(2)	48.0(12)
C32	9566(3)	6312(3)	3912(2)	45.2(11)
C33	9279(3)	6975(3)	4102(2)	44.3(11)
C34	8799(3)	6884(3)	4486(2)	42.1(11)
C35	9604(4)	4189(3)	4076(3)	69.1(17)
C36	10161(4)	7054(3)	3290(3)	59.2(14)
C37	6909(3)	8343(4)	4069(3)	62.5(15)
C38	6413(4)	8429(5)	3566(3)	80(2)
C39	6654(4)	8818(5)	3141(3)	84(2)
C40	7384(4)	9085(4)	3211(3)	70.1(17)
C41	7863(3)	8977(3)	3714(2)	49.3(12)
C42	8655(3)	9170(3)	3833(2)	45.1(11)
C43	9090(4)	9515(3)	3486(2)	54.7(13)
C44	9837(4)	9634(3)	3683(3)	61.4(15)
C45	10166(3)	9426(3)	4206(3)	58.0(15)
C46	9719(3)	9082(3)	4548(2)	44.9(11)
C47	9922(3)	8832(3)	5123(2)	50.1(12)
C48	10663(3)	8818(4)	5417(3)	59.6(15)
C49	10783(4)	8519(4)	5946(3)	70.5(18)
C50	10186(4)	8248(4)	6169(3)	70.1(18)
C51	9459(4)	8278(4)	5857(2)	58.4(14)
Co1	8371.8(4)	8591.1(4)	4833.5(3)	39.9(2)
Co2	7360.1(4)	9744.2(5)	5825.5(3)	41.6(2)
N1	9336(2)	8581(2)	5344.9(17)	46.1(10)
N2	8981(2)	8960(2)	4349.9(17)	42.4(9)
N3	7603(2)	8628(3)	4144.5(19)	50.6(10)

Atom	*x*	*y*	*z*	U(eq)
N4	8434(2)	7430(2)	4753.4(16)	42.4(9)
N5	8128(2)	6264(2)	5029.8(18)	46.7(10)
N6	7753(2)	8260(2)	5342.5(18)	44.2(9)
N7	7378(2)	8663(2)	5670.2(17)	43.4(9)
N8	6633(2)	9356(3)	6295.4(18)	47.4(10)
N9	6097(3)	8341(3)	6634(2)	54.6(11)
N10	8124(2)	9768(2)	6502.8(18)	45.2(10)
N11	7319(2)	10800(2)	5979.8(17)	44.4(9)
N12	6550(3)	10022(3)	5222.9(19)	54.0(11)
O1	8093(2)	9656(2)	4920.8(17)	57.3(10)
O2	8169(2)	9924(2)	5430.2(17)	57.0(10)
O3	4658(3)	10050(3)	7683(2)	75.6(13)
O4	5314(2)	11214(3)	7328.2(18)	62.5(10)
O5	9772(2)	4960(2)	3900.4(16)	55.4(9)
O6	10033(2)	6307(2)	3525.4(15)	51.0(9)
C52	6047(6)	5457(7)	5795(5)	131(4)
C53	6398(5)	4639(7)	4950(4)	129(4)
F1	4443(3)	6837(3)	3973(2)	109.4(16)
F2	5178(3)	7813(3)	4291(2)	117.1(17)
F3	4896(5)	6972(3)	4876(2)	166(4)
F4	6088(3)	6963(4)	4647(3)	143(3)
F5	5266(3)	5998(3)	4371(3)	131(2)
F6	5585(3)	6885(4)	3763(3)	131(2)
F7	8855(2)	6437(2)	6353.6(14)	73.1(10)
F8	8064(3)	6274(3)	6927(2)	107.5(16)
F9	8592(5)	7432(2)	6863.6(19)	138(3)
F10	9715(3)	6881(4)	7062(2)	137(2)
F11	9194(3)	5727(3)	7126.9(17)	102.0(16)
F12	8875(3)	6737(2)	7638.0(16)	83.6(12)
F13	8209.6(19)	3551.4(19)	6845.3(13)	59.7(8)
F14	7727(2)	4595(2)	7203.8(16)	77.1(11)
F15	8892(2)	4160(2)	7571.7(16)	75.9(10)
F16	7254.3(19)	3399(2)	7309.5(15)	66.4(9)
F17	8416(2)	2975(2)	7676.4(15)	71.3(10)
F18	7928(2)	4042(2)	8039.3(14)	73.2(10)
F19	8917(3)	3859(3)	5313(2)	114.8(18)
F20	7860(4)	3473(3)	5526(3)	127(2)
F21	8700(4)	2598(4)	5548(3)	148(2)
F22	7768(5)	2535(4)	4891(2)	181(4)
F23	8845(6)	2930(3)	4700(4)	198(4)
F24	7963(3)	3791(3)	4656(2)	100.8(15)
K1	8735.0(7)	4713.8(7)	6285.2(5)	55.7(3)
O7	7452(2)	5268(2)	5657.0(17)	58.5(10)
P1	5246.9(12)	6902.4(14)	4343.2(13)	98.5(8)
P2	8874.0(11)	6592.4(8)	6996.8(6)	61.5(4)
P3	8066.4(9)	3785.4(9)	7445.8(6)	53.5(4)
P4	8330.9(11)	3171.2(10)	5091.7(7)	63.7(4)
S1	6739.4(13)	4786.0(14)	5673.0(11)	100.5(7)

Table S23. Anisotropic Displacement Parameters ($Å^2 \times 10^3$) for **3**.

Atom	U_{11}	U_{22}	U_{33}	U_{23}	U_{13}	U_{12}
C1	76(4)	60(4)	70(4)	-17(3)	-16(3)	-2(3)
C2	100(6)	72(5)	90(5)	-21(4)	-42(5)	8(4)
C3	124(7)	77(5)	96(6)	-12(4)	-53(5)	12(5)
C4	107(6)	62(4)	79(5)	1(4)	-29(4)	8(4)
C5	68(4)	51(3)	50(3)	1(3)	-5(3)	3(3)
C6	61(3)	45(3)	49(3)	4(2)	5(2)	0(2)
C7	70(4)	47(3)	53(3)	0(2)	2(3)	4(3)

Atom	U_{11}	U_{22}	U_{33}	U_{23}	U_{13}	U_{12}
C8	63(3)	44(3)	63(3)	-3(3)	6(3)	1(3)
C9	61(3)	45(3)	60(3)	-11(3)	11(3)	-14(3)
C10	41(3)	52(3)	44(3)	-3(2)	5(2)	-12(2)
C11	47(3)	48(3)	43(3)	-5(2)	8(2)	-10(2)
C12	51(3)	63(3)	45(3)	-5(3)	9(2)	-7(3)
C13	56(3)	78(4)	46(3)	-4(3)	-1(2)	-10(3)
C14	58(3)	69(4)	59(3)	12(3)	-1(3)	0(3)
C15	50(3)	53(3)	50(3)	1(2)	7(2)	-5(2)
C16	41(3)	55(3)	48(3)	-1(2)	8(2)	-9(2)
C17	39(3)	63(3)	43(3)	3(2)	5(2)	-8(2)
C18	39(3)	62(3)	47(3)	-1(2)	4(2)	-5(2)
C19	38(3)	68(4)	50(3)	2(3)	1(2)	1(2)
C20	46(3)	78(4)	54(3)	8(3)	10(2)	0(3)
C21	52(3)	77(4)	52(3)	12(3)	11(2)	-4(3)
C22	45(3)	55(3)	53(3)	3(3)	4(2)	-7(2)
C23	101(6)	106(7)	116(7)	3(5)	62(5)	-17(5)
C24	70(4)	61(4)	74(4)	-9(3)	19(3)	6(3)
C25	52(3)	44(3)	45(3)	4(2)	4(2)	-12(2)
C26	52(3)	45(3)	53(3)	6(2)	7(2)	-15(2)
C27	50(3)	40(3)	48(3)	-3(2)	3(2)	-7(2)
C28	54(3)	39(3)	44(3)	-3(2)	-1(2)	-6(2)
C29	53(3)	37(3)	46(3)	2(2)	2(2)	-6(2)
C30	59(3)	39(3)	48(3)	-3(2)	1(2)	-7(2)
C31	55(3)	38(3)	48(3)	-3(2)	1(2)	-4(2)
C32	50(3)	41(3)	42(3)	3(2)	2(2)	0(2)
C33	51(3)	37(3)	42(3)	-1(2)	1(2)	-4(2)
C34	46(3)	36(2)	42(3)	-1(2)	1(2)	-4(2)
C35	88(5)	41(3)	84(4)	0(3)	29(4)	3(3)
C36	71(4)	47(3)	65(4)	8(3)	25(3)	5(3)
C37	50(3)	68(4)	67(4)	7(3)	6(3)	-7(3)
C38	49(3)	110(6)	76(5)	5(4)	-5(3)	-8(4)
C39	68(4)	111(6)	62(4)	8(4)	-15(3)	9(4)
C40	82(5)	76(4)	47(3)	9(3)	0(3)	2(4)
C41	58(3)	43(3)	45(3)	3(2)	6(2)	3(2)
C42	61(3)	28(2)	47(3)	0(2)	10(2)	3(2)
C43	75(4)	40(3)	53(3)	4(2)	23(3)	2(3)
C44	75(4)	48(3)	69(4)	-5(3)	35(3)	-13(3)
C45	51(3)	48(3)	80(4)	-18(3)	27(3)	-18(3)
C46	46(3)	33(2)	56(3)	-13(2)	11(2)	-7(2)
C47	48(3)	42(3)	58(3)	-18(2)	2(2)	-2(2)
C48	45(3)	58(3)	72(4)	-23(3)	-1(3)	-1(3)
C49	54(4)	74(4)	76(4)	-25(3)	-10(3)	7(3)
C50	77(4)	67(4)	57(4)	-6(3)	-14(3)	5(3)
C51	62(3)	56(3)	53(3)	-7(3)	-2(3)	-1(3)
Co1	41.0(4)	34.5(4)	43.0(4)	1.3(3)	4.5(3)	-7.2(3)
Co2	42.8(4)	39.0(4)	41.1(4)	-1.3(3)	2.4(3)	-6.8(3)
N1	51(2)	44(2)	42(2)	-3.2(18)	4.3(19)	-9.9(19)
N2	50(2)	28.2(19)	49(2)	-4.1(17)	9.5(18)	-3.9(17)
N3	50(2)	48(3)	51(3)	6.1(19)	4(2)	-1.6(19)
N4	47(2)	35(2)	42(2)	-1.0(17)	2.1(17)	-5.5(17)
N5	55(2)	36(2)	48(2)	2.1(18)	5.8(19)	-8.0(19)
N6	45(2)	38(2)	49(2)	-0.8(18)	4.9(18)	-6.2(18)
N7	43(2)	41(2)	45(2)	-3.4(18)	3.8(18)	-8.9(18)
N8	45(2)	48(2)	48(2)	-6.0(19)	5.0(18)	-8.1(19)
N9	52(3)	52(3)	59(3)	7(2)	9(2)	-13(2)
N10	42(2)	47(2)	47(2)	1.0(19)	7.2(18)	-6.7(18)
N11	49(2)	40(2)	44(2)	-1.0(18)	7.5(18)	-7.1(18)
N12	57(3)	49(3)	50(3)	-8(2)	-5(2)	3(2)
O1	61(2)	52(2)	59(2)	7.8(18)	11.5(18)	-8.8(18)

Atom	U_{11}	U_{22}	U_{33}	U_{23}	U_{13}	U_{12}
O2	63(2)	44(2)	68(3)	-7.5(18)	20.5(19)	-19.3(18)
O3	65(3)	94(3)	75(3)	9(3)	34(2)	3(2)
O4	56(2)	69(3)	65(2)	-3(2)	16.1(19)	9(2)
O5	69(2)	36.7(18)	60(2)	-1.8(17)	10.1(19)	-2.0(17)
O6	56(2)	46(2)	52(2)	1.9(16)	12.7(17)	3.6(16)
C52	107(7)	135(9)	166(11)	-36(8)	60(7)	-28(7)
C53	80(6)	158(10)	131(8)	-62(7)	-27(5)	42(6)
F1	81(3)	115(4)	125(4)	-27(3)	0(3)	14(3)
F2	147(5)	91(3)	111(4)	2(3)	15(3)	-19(3)
F3	333(10)	63(3)	69(3)	10(2)	-57(5)	-23(4)
F4	66(3)	160(5)	180(6)	60(4)	-34(3)	-43(3)
F5	91(3)	88(3)	201(6)	36(4)	-8(4)	-16(3)
F6	94(4)	143(5)	159(6)	1(4)	36(4)	1(3)
F7	88(3)	76(2)	51.1(19)	-5.6(17)	2.3(17)	3(2)
F8	100(3)	132(4)	91(3)	-6(3)	19(3)	-32(3)
F9	294(8)	50(2)	71(3)	8(2)	33(4)	48(4)
F10	131(4)	192(6)	84(3)	-17(4)	8(3)	-79(4)
F11	150(4)	74(3)	69(2)	-6(2)	-15(3)	44(3)
F12	131(4)	55(2)	64(2)	0.7(17)	14(2)	-5(2)
F13	68(2)	57.4(19)	54.4(18)	-11.0(14)	12.4(15)	-8.9(15)
F14	100(3)	50(2)	77(2)	-3.0(17)	5(2)	10.0(19)
F15	67(2)	75(2)	84(3)	-31(2)	7.0(18)	-10.5(18)
F16	63(2)	72(2)	65(2)	-7.0(17)	12.2(16)	-2.0(17)
F17	85(2)	55(2)	67(2)	-5.2(16)	-2.4(18)	14.7(18)
F18	88(3)	79(2)	50.5(19)	-14.6(17)	8.5(17)	11(2)
F19	126(4)	116(4)	90(3)	13(3)	-13(3)	-52(3)
F20	197(6)	75(3)	135(5)	2(3)	102(4)	-10(3)
F21	166(6)	128(5)	154(5)	71(4)	38(5)	36(4)
F22	284(9)	159(6)	101(4)	-31(4)	35(5)	-150(6)
F23	319(10)	74(3)	266(9)	29(4)	228(9)	43(5)
F24	97(3)	103(3)	92(3)	31(3)	-9(2)	-22(3)
K1	57.1(7)	48.6(7)	57.9(7)	2.9(5)	0.6(5)	-0.7(5)
O7	60(2)	52(2)	63(2)	4.1(18)	8.3(19)	-10.9(18)
P1	65.1(12)	78.3(14)	145(2)	15.4(14)	0.3(12)	-15.0(10)
P2	92.6(12)	37.9(7)	50.2(8)	-0.8(6)	2.6(8)	-5.4(7)
P3	63.7(9)	47.1(8)	47.4(7)	-7.3(6)	4.1(6)	2.6(6)
P4	83.9(11)	57.1(9)	51.4(8)	-2.9(7)	15.8(8)	-2.9(8)
S1	87.3(14)	87.5(15)	120.5(18)	-4.9(12)	2.4(12)	-11.8(11)

Table S24. Bond Lengths (Å) for **3**.

Atom	Atom	Length/Å	Atom	Atom	Length/Å
C1	C2	1.375(9)	C44	C45	1.360(9)
C1	N12	1.339(7)	C45	C46	1.396(8)
C2	C3	1.369(11)	C46	C47	1.457(8)
C3	C4	1.391(10)	C46	N2	1.352(6)
C4	C5	1.374(9)	C47	C48	1.402(8)
C5	C6	1.451(8)	C47	N1	1.347(7)
C5	N12	1.362(7)	C48	C49	1.375(10)
C6	C7	1.371(8)	C49	C50	1.376(10)
C6	N11	1.361(7)	C50	C51	1.397(9)
C7	C8	1.389(8)	C51	N1	1.342(7)
C8	C9	1.388(8)	Co1	N1	1.955(4)
C9	C10	1.381(8)	Co1	N2	1.868(4)
C10	C11	1.473(8)	Co1	N3	1.987(5)
C10	N11	1.340(6)	Co1	N4	2.010(4)
C11	C12	1.377(7)	Co1	N6	1.907(4)
C11	N10	1.367(7)	Co1	O1	1.921(4)
C12	C13	1.400(9)	Co2	N7	1.899(4)

Atom	Atom	Length/Å	Atom	Atom	Length/Å
C13	C14	1.374(9)	Co2	N8	2.012(4)
C14	C15	1.383(8)	Co2	N10	1.960(4)
C15	N10	1.337(7)	Co2	N11	1.857(4)
C16	C25	1.474(8)	Co2	N12	1.943(4)
C16	N8	1.325(7)	Co2	O2	1.920(4)
C16	N9	1.366(7)	N6	N7	1.335(6)
C17	C18	1.401(8)	O1	O2	1.316(5)
C17	C22	1.402(8)	O5	K1[1]	2.872(4)
C17	N8	1.382(7)	O6	K1[1]	2.807(4)
C18	C19	1.378(8)	C52	S1	1.767(11)
C19	C20	1.427(8)	C53	S1	1.788(10)
C19	O4	1.367(7)	F1	P1	1.574(5)
C20	C21	1.366(9)	F2	P1	1.573(6)
C20	O3	1.382(7)	F3	P1	1.557(8)
C21	C22	1.402(8)	F4	P1	1.572(5)
C22	N9	1.379(8)	F5	P1	1.556(6)
C23	O3	1.421(9)	F6	P1	1.648(7)
C24	O4	1.436(8)	F7	K1	2.971(4)
C25	C26	1.386(8)	F7	P2	1.595(4)
C25	N7	1.346(6)	F8	P2	1.543(5)
C26	C27	1.399(8)	F9	P2	1.545(4)
C27	C28	1.453(8)	F10	P2	1.579(6)
C27	N6	1.358(6)	F11	K1	2.714(4)
C28	N4	1.334(7)	F11	P2	1.607(4)
C28	N5	1.337(6)	F12	P2	1.592(4)
C29	C30	1.382(8)	F13	K1	2.692(4)
C29	C34	1.405(7)	F13	P3	1.593(3)
C29	N5	1.393(7)	F14	K1	3.149(5)
C30	C31	1.391(8)	F14	P3	1.591(4)
C31	C32	1.428(7)	F15	K1	3.260(4)
C31	O5	1.350(7)	F15	P3	1.604(4)
C32	C33	1.369(7)	F16	P3	1.591(4)
C32	O6	1.379(6)	F17	P3	1.591(4)
C33	C34	1.399(7)	F18	P3	1.583(4)
C34	N4	1.380(6)	F19	K1	2.869(6)
C35	O5	1.442(7)	F19	P4	1.615(5)
C36	O6	1.442(6)	F20	K1	3.075(6)
C37	C38	1.398(9)	F20	P4	1.567(5)
C37	N3	1.327(7)	F21	P4	1.549(6)
C38	C39	1.373(11)	F22	P4	1.514(5)
C39	C40	1.377(10)	F23	P4	1.510(6)
C40	C41	1.385(8)	F24	P4	1.569(5)
C41	C42	1.448(8)	K1	O7	2.715(4)
C41	N3	1.369(7)	K1	P3	3.654(2)
C42	C43	1.391(7)	K1	S1	3.651(3)
C42	N2	1.347(6)	O7	S1	1.538(4)
C43	C44	1.365(9)			

Table S25. Bond Angles (˚) for **3**.

Atom	Atom	Atom	Angle/˚	Atom	Atom	Atom	Angle/˚
N12	C1	C2	121.0(6)	C32	O6	C36	115.6(4)
C3	C2	C1	120.6(7)	C32	O6	K1[1]	117.0(3)
C2	C3	C4	118.6(7)	C36	O6	K1[1]	116.3(3)
C5	C4	C3	118.8(7)	P2	F7	K1	102.20(18)
C4	C5	C6	124.5(6)	P2	F11	K1	113.1(2)
N12	C5	C4	121.8(6)	P3	F13	K1	114.57(17)
N12	C5	C6	113.5(5)	P3	F14	K1	95.18(18)
C7	C6	C5	128.5(5)	P3	F15	K1	90.85(17)

Atom	Atom	Atom	Angle/˚	Atom	Atom	Atom	Angle/˚
N11	C6	C5	111.7(5)	P4	F19	K1	119.2(3)
N11	C6	C7	119.8(5)	P4	F20	K1	111.0(3)
C6	C7	C8	119.4(5)	O5[1]	K1	F7	75.72(11)
C9	C8	C7	119.7(5)	O5[1]	K1	F14	143.82(11)
C10	C9	C8	119.2(5)	O5[1]	K1	F15	106.85(11)
C9	C10	C11	128.3(5)	O5[1]	K1	F20	115.78(13)
N11	C10	C9	120.0(5)	O5[1]	K1	P3	131.36(9)
N11	C10	C11	111.6(5)	O5[1]	K1	S1	144.60(10)
C12	C11	C10	124.7(5)	O6[1]	K1	O5[1]	53.71(11)
N10	C11	C10	112.8(4)	O6[1]	K1	F7	124.46(12)
N10	C11	C12	122.4(5)	O6[1]	K1	F14	112.62(11)
C11	C12	C13	118.3(5)	O6[1]	K1	F15	74.28(10)
C14	C13	C12	119.0(5)	O6[1]	K1	F19	67.14(14)
C13	C14	C15	119.8(6)	O6[1]	K1	F20	88.46(15)
N10	C15	C14	121.9(6)	O6[1]	K1	P3	87.81(8)
N8	C16	C25	116.8(5)	O6[1]	K1	S1	141.70(9)
N8	C16	N9	111.7(5)	F7	K1	F14	94.01(11)
N9	C16	C25	131.5(5)	F7	K1	F15	104.08(10)
C18	C17	C22	121.2(5)	F7	K1	F20	138.54(14)
N8	C17	C18	131.1(5)	F7	K1	P3	114.97(9)
N8	C17	C22	107.7(5)	F7	K1	S1	92.64(9)
C19	C18	C17	117.9(5)	F11	K1	O5[1]	80.12(15)
C18	C19	C20	120.1(6)	F11	K1	O6[1]	98.64(13)
O4	C19	C18	124.8(5)	F11	K1	F7	46.27(12)
O4	C19	C20	115.0(5)	F11	K1	F14	68.52(15)
C21	C20	C19	122.4(5)	F11	K1	F15	59.26(12)
C21	C20	O3	124.1(6)	F11	K1	F19	153.58(17)
O3	C20	C19	113.5(6)	F11	K1	F20	163.53(17)
C20	C21	C22	117.3(6)	F11	K1	O7	108.63(13)
C21	C22	C17	121.0(6)	F11	K1	P3	77.62(12)
N9	C22	C17	107.1(5)	F11	K1	S1	115.79(12)
N9	C22	C21	131.9(5)	F13	K1	O5[1]	130.81(11)
C26	C25	C16	140.0(5)	F13	K1	O6[1]	78.11(11)
N7	C25	C16	109.2(5)	F13	K1	F7	137.67(11)
N7	C25	C26	110.8(5)	F13	K1	F11	100.07(14)
C25	C26	C27	102.9(4)	F13	K1	F14	44.28(10)
C26	C27	C28	140.4(5)	F13	K1	F15	42.83(9)
N6	C27	C26	109.8(5)	F13	K1	F19	98.53(12)
N6	C27	C28	109.7(5)	F13	K1	F20	66.71(12)
N4	C28	C27	116.4(4)	F13	K1	O7	101.92(12)
N4	C28	N5	112.3(5)	F13	K1	P3	23.35(7)
N5	C28	C27	131.3(5)	F13	K1	S1	79.76(9)
C30	C29	C34	121.8(5)	F14	K1	F15	41.30(10)
C30	C29	N5	132.6(5)	F14	K1	P3	25.70(7)
N5	C29	C34	105.6(4)	F14	K1	S1	68.96(9)
C29	C30	C31	118.1(5)	F15	K1	P3	26.03(7)
C30	C31	C32	119.4(5)	F15	K1	S1	108.40(9)
O5	C31	C30	124.9(5)	F19	K1	O5[1]	73.53(13)
O5	C31	C32	115.7(5)	F19	K1	F7	122.59(13)
C33	C32	C31	122.8(5)	F19	K1	F14	136.83(13)
C33	C32	O6	123.7(5)	F19	K1	F15	130.73(13)
O6	C32	C31	113.5(4)	F19	K1	F20	42.72(14)
C32	C33	C34	117.0(5)	F19	K1	P3	121.85(11)
C33	C34	C29	120.9(5)	F19	K1	S1	85.87(12)
N4	C34	C29	108.5(4)	F20	K1	F14	95.04(12)
N4	C34	C33	130.6(5)	F20	K1	F15	109.27(12)
N3	C37	C38	121.6(6)	F20	K1	P3	87.89(10)
C39	C38	C37	118.7(6)	F20	K1	S1	53.99(13)
C38	C39	C40	119.7(6)	O7	K1	O5[1]	124.91(12)

Atom	Atom	Atom	Angle/˚	Atom	Atom	Atom	Angle/˚
C39	C40	C41	119.8(6)	O7	K1	O6[1]	152.15(13)
C40	C41	C42	125.9(5)	O7	K1	F7	74.25(11)
N3	C41	C40	119.9(5)	O7	K1	F14	83.36(12)
N3	C41	C42	114.1(4)	O7	K1	F15	124.66(12)
C43	C42	C41	128.4(5)	O7	K1	F19	85.51(15)
N2	C42	C41	112.2(4)	O7	K1	F20	66.79(15)
N2	C42	C43	119.4(5)	O7	K1	P3	103.13(10)
C44	C43	C42	118.6(6)	O7	K1	S1	22.36(9)
C45	C44	C43	122.1(5)	S1	K1	P3	83.95(6)
C44	C45	C46	118.4(5)	S1	O7	K1	115.5(2)
C45	C46	C47	129.5(5)	F1	P1	F6	86.9(3)
N2	C46	C45	119.3(5)	F2	P1	F1	88.5(3)
N2	C46	C47	111.2(4)	F2	P1	F6	89.1(3)
C48	C47	C46	123.7(5)	F3	P1	F1	91.0(4)
N1	C47	C46	114.0(4)	F3	P1	F2	87.3(3)
N1	C47	C48	122.3(6)	F3	P1	F4	95.8(4)
C49	C48	C47	117.8(6)	F3	P1	F6	175.9(4)
C48	C49	C50	120.1(6)	F4	P1	F1	173.2(4)
C49	C50	C51	119.7(6)	F4	P1	F2	91.6(3)
N1	C51	C50	120.6(6)	F4	P1	F6	86.2(4)
N1	Co1	N3	162.24(19)	F5	P1	F1	88.0(3)
N1	Co1	N4	89.71(17)	F5	P1	F2	176.4(3)
N2	Co1	N1	81.47(18)	F5	P1	F3	92.8(3)
N2	Co1	N3	81.42(18)	F5	P1	F4	91.9(3)
N2	Co1	N4	102.99(17)	F5	P1	F6	90.6(4)
N2	Co1	N6	177.42(17)	F7	P2	F11	89.1(2)
N2	Co1	O1	86.81(17)	F7	P2	K1	52.57(15)
N3	Co1	N4	89.48(18)	F8	P2	F7	88.8(3)
N6	Co1	N1	97.87(18)	F8	P2	F9	92.2(4)
N6	Co1	N3	99.42(18)	F8	P2	F10	177.4(4)
N6	Co1	N4	79.48(17)	F8	P2	F11	89.6(3)
N6	Co1	O1	90.85(17)	F8	P2	F12	89.9(3)
O1	Co1	N1	99.12(18)	F8	P2	K1	69.6(2)
O1	Co1	N3	84.67(18)	F9	P2	F7	90.0(2)
O1	Co1	N4	167.75(17)	F9	P2	F10	90.2(4)
N7	Co2	N8	80.04(18)	F9	P2	F11	178.0(4)
N7	Co2	N10	98.99(18)	F9	P2	F12	90.3(2)
N7	Co2	N12	97.20(19)	F9	P2	K1	137.0(2)
N7	Co2	O2	90.83(17)	F10	P2	F7	90.1(3)
N10	Co2	N8	87.40(17)	F10	P2	F11	88.0(4)
N11	Co2	N7	178.61(19)	F10	P2	F12	91.2(3)
N11	Co2	N8	98.77(18)	F10	P2	K1	107.9(3)
N11	Co2	N10	81.63(18)	F11	P2	K1	43.03(16)
N11	Co2	N12	82.11(19)	F12	P2	F7	178.7(3)
N11	Co2	O2	90.44(17)	F12	P2	F11	90.7(2)
N12	Co2	N8	92.1(2)	F12	P2	K1	126.69(15)
N12	Co2	N10	163.47(19)	F13	P3	F15	88.8(2)
O2	Co2	N8	168.15(18)	F13	P3	K1	42.08(13)
O2	Co2	N10	86.59(18)	F14	P3	F13	89.5(2)
O2	Co2	N12	96.5(2)	F14	P3	F15	90.2(2)
C47	N1	Co1	114.4(4)	F14	P3	K1	59.11(16)
C51	N1	C47	119.5(5)	F15	P3	K1	63.12(16)
C51	N1	Co1	125.7(4)	F16	P3	F13	89.86(19)
C42	N2	C46	122.2(4)	F16	P3	F14	90.5(2)
C42	N2	Co1	118.7(4)	F16	P3	F15	178.5(2)
C46	N2	Co1	118.8(3)	F16	P3	K1	116.14(15)
C37	N3	C41	120.0(5)	F17	P3	F13	89.3(2)
C37	N3	Co1	127.3(4)	F17	P3	F14	178.7(2)
C41	N3	Co1	112.7(4)	F17	P3	F15	89.2(2)

Atom	Atom	Atom	Angle/˚	Atom	Atom	Atom	Angle/˚
C28	N4	C34	106.3(4)	F17	P3	F16	90.1(2)
C28	N4	Co1	113.9(3)	F17	P3	K1	119.55(16)
C34	N4	Co1	139.6(3)	F18	P3	F13	178.5(2)
C28	N5	C29	107.3(4)	F18	P3	F14	89.2(2)
C27	N6	Co1	120.5(4)	F18	P3	F15	90.4(2)
N7	N6	C27	108.2(4)	F18	P3	F16	91.0(2)
N7	N6	Co1	131.4(3)	F18	P3	F17	92.0(2)
C25	N7	Co2	120.5(4)	F18	P3	K1	136.40(17)
N6	N7	C25	108.2(4)	F20	P4	F19	86.1(3)
N6	N7	Co2	131.1(3)	F20	P4	F24	91.3(3)
C16	N8	C17	107.1(4)	F21	P4	F19	93.0(4)
C16	N8	Co2	113.0(4)	F21	P4	F20	86.2(4)
C17	N8	Co2	139.8(4)	F21	P4	F24	176.6(4)
C16	N9	C22	106.5(5)	F22	P4	F19	178.6(5)
C11	N10	Co2	114.3(3)	F22	P4	F20	92.6(4)
C15	N10	C11	118.5(5)	F22	P4	F21	87.3(4)
C15	N10	Co2	127.1(4)	F22	P4	F24	95.2(4)
C6	N11	Co2	118.4(4)	F23	P4	F19	88.5(4)
C10	N11	C6	121.9(5)	F23	P4	F20	174.5(5)
C10	N11	Co2	119.7(4)	F23	P4	F21	93.0(4)
C1	N12	C5	119.1(5)	F23	P4	F22	92.8(5)
C1	N12	Co2	126.6(4)	F23	P4	F24	89.3(4)
C5	N12	Co2	114.3(4)	F24	P4	F19	84.5(3)
O2	O1	Co1	117.0(3)	C52	S1	C53	97.6(5)
O1	O2	Co2	117.7(3)	C52	S1	K1	129.1(4)
C20	O3	C23	116.8(6)	C53	S1	K1	122.8(3)
C19	O4	C24	116.7(4)	O7	S1	C52	106.0(4)
C31	O5	C35	116.3(5)	O7	S1	C53	101.0(5)
C31	O5	K1[1]	116.3(3)	O7	S1	K1	42.17(17)
C35	O5	K1[1]	118.7(4)				

Table S26. Torsion Angles (˚) for **3**.

A	B	C	D	Angle/˚	A	B	C	D	Angle/˚
C1	C2	C3	C4	0.5(16)	C43	C42	N2	C46	0.9(7)
C2	C1	N12	C5	1.7(11)	C43	C42	N2	Co1	173.8(4)
C2	C1	N12	Co2	179.1(7)	C43	C44	C45	C46	-0.2(9)
C2	C3	C4	C5	1.0(16)	C44	C45	C46	C47	-177.8(5)
C3	C4	C5	C6	-177.2(8)	C44	C45	C46	N2	0.6(8)
C3	C4	C5	N12	-1.3(13)	C45	C46	C47	C48	-8.0(9)
C4	C5	C6	C7	-1.1(12)	C45	C46	C47	N1	174.6(5)
C4	C5	C6	N11	177.1(7)	C45	C46	N2	C42	-1.0(7)
C4	C5	N12	C1	-0.1(10)	C45	C46	N2	Co1	-174.0(4)
C4	C5	N12	Co2	-177.8(6)	C46	C47	C48	C49	-175.7(5)
C5	C6	C7	C8	176.5(6)	C46	C47	N1	C51	174.8(5)
C5	C6	N11	C10	-177.7(5)	C46	C47	N1	Co1	1.6(5)
C5	C6	N11	Co2	0.1(6)	C47	C46	N2	C42	177.7(4)
C6	C5	N12	C1	176.2(6)	C47	C46	N2	Co1	4.7(5)
C6	C5	N12	Co2	-1.5(7)	C47	C48	C49	C50	-0.1(9)
C6	C7	C8	C9	1.5(9)	C48	C47	N1	C51	-2.7(8)
C7	C6	N11	C10	0.7(8)	C48	C47	N1	Co1	-175.9(4)
C7	C6	N11	Co2	178.5(4)	C48	C49	C50	C51	-0.1(10)
C7	C8	C9	C10	-0.5(9)	C49	C50	C51	N1	-1.1(10)
C8	C9	C10	C11	-177.1(5)	C50	C51	N1	C47	2.4(8)
C8	C9	C10	N11	-0.4(8)	C50	C51	N1	Co1	174.8(4)
C9	C10	C11	C12	-0.8(9)	Co1	N6	N7	C25	178.6(4)
C9	C10	C11	N10	177.1(5)	Co1	N6	N7	Co2	-6.3(7)
C9	C10	N11	C6	0.3(8)	Co1	O1	O2	Co2	-85.0(4)
C9	C10	N11	Co2	-177.5(4)	N1	C47	C48	C49	1.5(8)

A	B	C	D	Angle/˚	A	B	C	D	Angle/˚
C10	C11	C12	C13	176.6(5)	N1	Co1	N2	C42	-176.3(4)
C10	C11	N10	C15	-176.3(4)	N1	Co1	N2	C46	-3.1(3)
C10	C11	N10	Co2	-0.1(5)	N2	C42	C43	C44	-0.4(8)
C11	C10	N11	C6	177.5(5)	N2	C46	C47	C48	173.5(5)
C11	C10	N11	Co2	-0.2(6)	N2	C46	C47	N1	-3.9(6)
C11	C12	C13	C14	-0.4(9)	N3	C37	C38	C39	0.0(12)
C12	C11	N10	C15	1.7(8)	N3	C41	C42	C43	179.1(5)
C12	C11	N10	Co2	177.9(4)	N3	C41	C42	N2	0.3(6)
C12	C13	C14	C15	1.2(9)	N3	Co1	N2	C42	8.5(4)
C13	C14	C15	N10	-0.7(9)	N3	Co1	N2	C46	-178.3(4)
C14	C15	N10	C11	-0.8(8)	N4	C28	N5	C29	0.1(6)
C14	C15	N10	Co2	-176.4(4)	N4	Co1	N2	C42	95.9(4)
C16	C25	C26	C27	178.4(7)	N4	Co1	N2	C46	-90.9(4)
C16	C25	N7	Co2	5.6(6)	N5	C28	N4	C34	0.2(6)
C16	C25	N7	N6	-178.6(4)	N5	C28	N4	Co1	-176.6(3)
C17	C18	C19	C20	-0.2(8)	N5	C29	C30	C31	-179.2(5)
C17	C18	C19	O4	178.6(5)	N5	C29	C34	C33	-179.4(4)
C17	C22	N9	C16	-0.1(6)	N5	C29	C34	N4	0.5(5)
C18	C17	C22	C21	0.7(8)	N6	C27	C28	N4	-2.0(6)
C18	C17	C22	N9	178.9(5)	N6	C27	C28	N5	175.2(5)
C18	C17	N8	C16	-178.8(5)	N7	C25	C26	C27	-1.1(6)
C18	C17	N8	Co2	4.6(9)	N8	C16	C25	C26	179.1(6)
C18	C19	C20	C21	1.4(9)	N8	C16	C25	N7	-1.4(7)
C18	C19	C20	O3	-178.7(5)	N8	C16	N9	C22	-0.1(6)
C18	C19	O4	C24	-1.9(8)	N8	C17	C18	C19	177.4(5)
C19	C20	C21	C22	-1.5(9)	N8	C17	C22	C21	-177.9(5)
C19	C20	O3	C23	173.0(6)	N8	C17	C22	N9	0.3(6)
C20	C19	O4	C24	176.9(5)	N8	Co2	N7	C25	-5.9(4)
C20	C21	C22	C17	0.5(8)	N8	Co2	N7	N6	179.5(5)
C20	C21	C22	N9	-177.2(6)	N8	Co2	N11	C6	-91.6(4)
C21	C20	O3	C23	-7.1(10)	N8	Co2	N11	C10	86.2(4)
C21	C22	N9	C16	177.8(6)	N9	C16	C25	C26	-2.0(11)
C22	C17	C18	C19	-0.9(8)	N9	C16	C25	N7	177.5(5)
C22	C17	N8	C16	-0.3(6)	N9	C16	N8	C17	0.3(6)
C22	C17	N8	Co2	-177.0(4)	N9	C16	N8	Co2	177.9(3)
C25	C16	N8	C17	179.4(4)	N10	C11	C12	C13	-1.1(8)
C25	C16	N8	Co2	-3.0(6)	N10	Co2	N7	C25	79.9(4)
C25	C16	N9	C22	-179.0(5)	N10	Co2	N7	N6	-94.8(4)
C25	C26	C27	C28	178.5(7)	N10	Co2	N11	C6	-177.7(4)
C25	C26	C27	N6	0.8(6)	N10	Co2	N11	C10	0.2(4)
C26	C25	N7	Co2	-174.6(4)	N11	C6	C7	C8	-1.6(9)
C26	C25	N7	N6	1.1(6)	N11	C10	C11	C12	-177.7(5)
C26	C27	C28	N4	-179.7(6)	N11	C10	C11	N10	0.2(6)
C26	C27	C28	N5	-2.6(11)	N12	C1	C2	C3	-2.0(15)
C26	C27	N6	Co1	-179.4(3)	N12	C5	C6	C7	-177.3(6)
C26	C27	N6	N7	-0.1(6)	N12	C5	C6	N11	0.9(8)
C27	C28	N4	C34	177.9(4)	N12	Co2	N7	C25	-96.8(4)
C27	C28	N4	Co1	1.1(6)	N12	Co2	N7	N6	88.6(4)
C27	C28	N5	C29	-177.1(5)	N12	Co2	N11	C6	-0.7(4)
C27	N6	N7	C25	-0.6(5)	N12	Co2	N11	C10	177.2(4)
C27	N6	N7	Co2	174.6(4)	O1	Co1	N2	C42	-76.6(4)
C28	C27	N6	Co1	2.1(6)	O1	Co1	N2	C46	96.6(4)
C28	C27	N6	N7	-178.6(4)	O2	Co2	N7	C25	166.5(4)
C29	C30	C31	C32	-0.6(8)	O2	Co2	N7	N6	-8.1(4)
C29	C30	C31	O5	177.2(5)	O2	Co2	N11	C6	95.8(4)
C29	C34	N4	C28	-0.4(5)	O2	Co2	N11	C10	-86.3(4)
C29	C34	N4	Co1	175.1(4)	O3	C20	C21	C22	178.6(5)
C30	C29	C34	C33	2.6(8)	O4	C19	C20	C21	-177.4(5)
C30	C29	C34	N4	-177.5(5)	O4	C19	C20	O3	2.4(7)

A	B	C	D	Angle/˚	A	B	C	D	Angle/˚
C30	C29	N5	C28	177.3(6)	O5	C31	C32	C33	-175.7(5)
C30	C31	C32	C33	2.2(8)	O5	C31	C32	O6	4.3(7)
C30	C31	C32	O6	-177.7(5)	O6	C32	C33	C34	178.5(4)
C30	C31	O5	C35	3.5(8)	K1	F7	P2	F8	-64.9(3)
C30	C31	O5	$K1^1$	-144.0(4)	K1	F7	P2	F9	-157.1(3)
C31	C32	C33	C34	-1.4(7)	K1	F7	P2	F10	112.7(3)
C31	C32	O6	C36	175.1(5)	K1	F7	P2	F11	24.7(2)
C31	C32	O6	$K1^1$	-42.2(5)	K1	F11	P2	F7	-29.1(3)
C32	C31	O5	C35	-178.7(5)	K1	F11	P2	F8	59.7(3)
C32	C31	O5	$K1^1$	33.8(5)	K1	F11	P2	F10	-119.2(3)
C32	C33	C34	C29	-0.9(7)	K1	F11	P2	F12	149.6(3)
C32	C33	C34	N4	179.2(5)	K1	F13	P3	F14	40.8(2)
C33	C32	O6	C36	-4.9(7)	K1	F13	P3	F15	-49.3(2)
C33	C32	O6	$K1^1$	137.9(4)	K1	F13	P3	F16	131.3(2)
C33	C34	N4	C28	179.5(5)	K1	F13	P3	F17	-138.6(2)
C33	C34	N4	Co1	-5.1(9)	K1	F14	P3	F13	-30.71(16)
C34	C29	C30	C31	-1.8(8)	K1	F14	P3	F15	58.09(17)
C34	C29	N5	C28	-0.4(5)	K1	F14	P3	F16	-120.57(16)
C37	C38	C39	C40	2.7(13)	K1	F14	P3	F18	148.45(18)
C38	C37	N3	C41	-3.8(10)	K1	F15	P3	F13	34.74(15)
C38	C37	N3	Co1	178.0(5)	K1	F15	P3	F14	-54.76(17)
C38	C39	C40	C41	-1.7(12)	K1	F15	P3	F17	124.04(16)
C39	C40	C41	C42	174.8(6)	K1	F15	P3	F18	-143.97(17)
C39	C40	C41	N3	-2.1(10)	K1	F19	P4	F20	9.4(3)
C40	C41	C42	C43	2.0(9)	K1	F19	P4	F21	-76.6(4)
C40	C41	C42	N2	-176.7(6)	K1	F19	P4	F23	-169.5(4)
C40	C41	N3	C37	4.8(8)	K1	F19	P4	F24	101.1(3)
C40	C41	N3	Co1	-176.7(5)	K1	F20	P4	F19	-8.2(3)
C41	C42	C43	C44	-179.0(5)	K1	F20	P4	F21	85.0(4)
C41	C42	N2	C46	179.7(4)	K1	F20	P4	F22	172.2(4)
C41	C42	N2	Co1	-7.3(5)	K1	F20	P4	F24	-92.6(3)
C42	C41	N3	C37	-172.4(5)	K1	O7	S1	C52	131.3(5)
C42	C41	N3	Co1	6.0(6)	K1	O7	S1	C53	-127.4(4)
C42	C43	C44	C45	0.0(9)					

Table S27. Hydrogen Atom Coordinates (Å×10^4) and Isotropic Displacement Parameters (Å^2×10^3) for **3**.

Atom	*x*	*y*	*z*	U(eq)
H1	6267.1	8994.29	4876.21	87
H2	5320.17	9450.22	4188.5	114
H3	4998.58	10761.51	4146.59	129
H4	5670.45	11620.67	4806.48	106
H7	6408.58	12344.26	5512.55	70
H8	7162.28	12877.36	6302.7	69
H9	8033.7	12084.99	6873.32	67
H12	8822.85	11121.42	7376.72	64
H13	9477.92	10037.43	7825.34	74
H14	9241.71	8803.49	7436.72	76
H15	8407.1	8657.69	6605.75	61
H18	6257.07	10834.96	6650.37	60
H21	5096.09	8673.95	7363.89	72
H23A	3958.59	9161.17	7523.52	154
H23B	3858.28	9649.34	8060.94	154
H23C	4557.4	9070.68	8085.84	154
H24A	5565.32	11928.74	6749.75	101
H24B	6227.21	11835.96	7274.85	101
H24C	5505.05	12354.11	7320.39	101

Atom	*x*	*y*	*z*	U(eq)
H26	6972.65	6925.55	5934.51	61
H30	8802.49	4973.96	4602.31	60
H33	9401.26	7475.63	3979.67	53
H35A	9852.94	3802.59	3877.09	104
H35B	9058.73	4104.24	3994.64	104
H35C	9785.51	4135.78	4475.3	104
H36A	10454.23	7383.57	3576.22	89
H36B	9676.76	7303.3	3149.14	89
H36C	10438.75	6981.56	2985.89	89
H37	6745.85	8074.66	4364.62	75
H38	5917.66	8222.43	3517.99	97
H39	6319.57	8902.84	2800.94	100
H40	7558.07	9341.88	2916.01	84
H43	8871.34	9664.12	3119.93	66
H44	10135.86	9870.17	3448.18	74
H45	10686.9	9513.45	4334.62	70
H48	11068.08	9008.9	5257.11	72
H49	11277.58	8500.1	6156.53	85
H50	10268.34	8040.29	6534.09	84
H51	9048.5	8082.1	6009.36	70
H5	7916.24	5902.37	5204.77	56
H9A	5977.86	7855.89	6694.79	66
H52A	6148.81	5616.34	6185.01	197
H52B	5549.72	5211.98	5709.54	197
H52C	6057.56	5914.97	5558.61	197
H53A	6491.34	5107.8	4744.56	193
H53B	5856.75	4533.52	4889.02	193
H53C	6659.72	4195.97	4819.24	193

Table S28. Solvent masks information for **3**.

Number	X	Y	Z	Volume	Electron count	Content
1	0.500	0.861	0.750	726.7	167.7	?
2	0.500	-0.065	0.250	726.7	168.0	?

18c. Single crystal X-ray crystallography for **1-I**.

Data Collection

Single crystals suitable for X-ray analysis were obtained by diffusion of diethyl ether into a saturated anhydrous CH_3CN solution of **1** with 2.5 equiv NaH. A dark red crystal with approximate dimensions 0.13 × 0.11 × 0.1 mm^3 was selected under oil under ambient conditions and attached to the tip of a MiTeGen MicroMount©. The crystal was mounted in a cold nitrogen stream at 100 K and centered in the X-ray beam using a video camera. The crystal evaluation and data collection were performed on a Rigaku XtaLAB Synergy R, DW system, HyPix-Arc 150 with Cu Kα (λ = 1.54184 Å) radiation and the diffractometer.

The initial cell constants were obtained from ω scans consisting of 12 frames collected at intervals of 0.5º in a 10º range of about ω with an exposure time of 0.6 s per frame. The reflections were successfully indexed by an automated indexing routine built into the CrysAlisPro program suite. The final cell constants were calculated from a set of 16819 strong reflections from the actual data collection. The data were collected using a Laue symmetry data collection routine to survey the reciprocal space to the extent of a full sphere to a resolution of 0.84 Å. A total of 37259 data were harvested by collecting 29 sets of frames with 0.5º scans in ω and φ with exposure times of 0.59 sec per frame. These highly redundant datasets were corrected for Lorentz and polarization effects. The absorption correction was based on fitting a function to the empirical transmission surface sampled by multiple equivalent measurements.

II. Structure Solution and Refinement

The systematic absences in the diffraction data were consistent for the space group $P2_1/n$ and yielded chemically reasonable and computationally stable refinement results.

A successful solution by charge-flipping provided most non-hydrogen atoms from the E-map. The remaining non-hydrogen atoms were located in an alternating series of least-squares cycles and difference Fourier maps. All non-hydrogen atoms were refined with anisotropic displacement coefficients. All hydrogen atoms were included in the structure factor calculation at idealized positions and were allowed to ride on the neighboring atoms with relative isotropic displacement coefficients.

The asymmetric unit consists of one molecule of the cobalt complex and one molecule of PF_6^- and MeCN, respectively. The final least-squares refinement of 659 parameters against 8440 data resulted in residuals R (based on F2 for I≥2σ) and wR (based on F2 for I≥2σ) of 0.0422 and 0.1036, respectively.

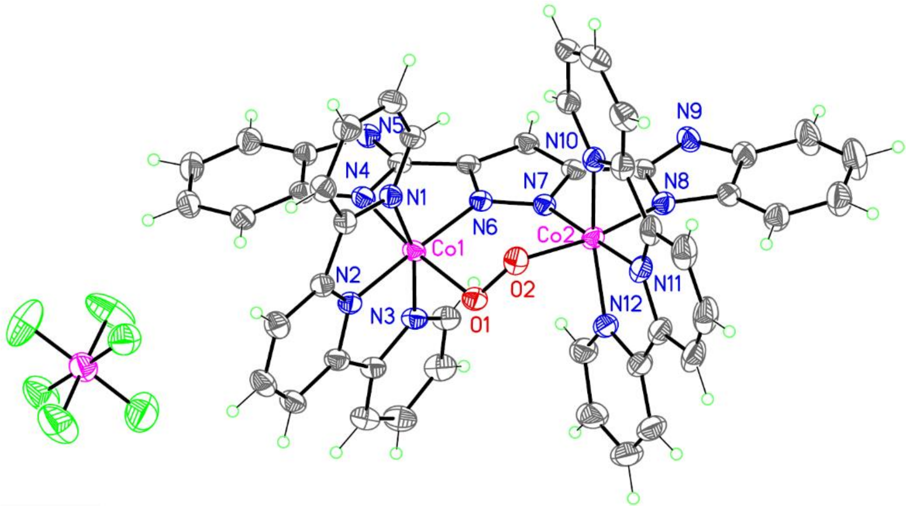

Figure S93. ORTEP drawings of **1-I** with thermal ellipsoids at 50% probability.

Table S29. Crystal data and structure refinement for **1-I**.

Identification code	220925lt2_auto
Empirical formula	$C_{49}H_{34}Co_2F_6N_{13}O_2P$
Formula weight	1099.72
Temperature/K	101(2)
Crystal system	monoclinic
Space group	$P2_1/n$
a/Å	12.3081(2)
b/Å	14.4481(2)
c/Å	26.8183(4)
α/°	90
β/°	97.1120(10)
γ/°	90
Volume/Å^3	4732.37(12)
Z	4
ρ_{calc}g/cm^3	1.544
μ/mm^{-1}	6.504
F(000)	2232.0
Crystal size/mm^3	0.13 × 0.11 × 0.1
Radiation	Cu Kα (λ = 1.54184)
2Θ range for data collection/°	6.642 to 134.146
Index ranges	-10 ≤ h ≤ 14, -17 ≤ k ≤ 17, -31 ≤ l ≤ 32
Reflections collected	37259
Independent reflections	8440 [R_{int} = 0.0248, R_{sigma} = 0.0241]
Data/restraints/parameters	8440/0/659
Goodness-of-fit on F^2	1.028
Final R indexes [I>=2σ (I)]	R_1 = 0.0422, wR_2 = 0.1036
Final R indexes [all data]	R_1 = 0.0501, wR_2 = 0.1077
Largest diff. peak/hole / e Å^{-3}	0.46/-0.35

Table S30. Fractional Atomic Coordinates (×10^4) and Equivalent Isotropic Displacement Parameters (Å^2×10^3) for **1-I**.

Atom	*x*	*y*	*z*	U(eq)
C1	5119(2)	5852(2)	7086.1(10)	28.7(6)
C2	5212(2)	4918(2)	7198.0(11)	32.7(6)
C3	4278(2)	4391(2)	7189.8(11)	32.8(6)
C4	3267(2)	4816(2)	7076.0(10)	29.8(6)
C5	3222(2)	5744.4(19)	6964.9(9)	24.6(6)
C6	2217(2)	6293.9(19)	6855.0(9)	24.7(6)
C7	1135(2)	5994(2)	6819.0(10)	30.2(6)
C8	317(2)	6644(2)	6710.1(11)	32.5(6)
C9	551(2)	7564(2)	6639.0(11)	30.0(6)
C10	1646(2)	7829(2)	6685.2(10)	26.3(6)
C11	2093(2)	8756.4(19)	6638.6(10)	26.3(6)
C12	1475(2)	9557(2)	6575.9(11)	31.1(6)
C13	2007(2)	10399(2)	6564.1(12)	36.4(7)
C14	3138(2)	10421(2)	6629.3(11)	35.7(7)
C15	3711(2)	9597.9(19)	6691.8(11)	30.3(6)
C16	5219(2)	8386.0(18)	7615.3(9)	23.0(5)
C17	4476(2)	8546.9(19)	8282.3(10)	25.3(6)
C18	4182(2)	8787(2)	8752.9(11)	31.7(6)
C19	3183(2)	8481(2)	8875.3(11)	33.5(6)
C20	2489(2)	7929(2)	8543.3(11)	31.4(6)
C21	2759(2)	7679.1(19)	8075.1(11)	27.1(6)
C22	3761(2)	8002.4(18)	7944.9(10)	24.3(5)
C23	5865(2)	8409.5(18)	7194.5(9)	22.8(5)
C24	6820(2)	8809.0(19)	7061.8(10)	24.6(6)
C25	6834(2)	8555.6(18)	6560.0(10)	23.0(5)
C26	7528(2)	8637.2(19)	6159.4(9)	23.4(5)
C27	8755(2)	8851(2)	5681.1(10)	32.0(6)
C28	9680(3)	9090(3)	5456.1(12)	49.1(9)
C29	9741(3)	8790(3)	4971.3(13)	57.5(11)
C30	8900(3)	8278(2)	4705.2(12)	42.9(8)
C31	7977(2)	8033(2)	4917.2(10)	33.0(6)
C32	7911(2)	8319.2(19)	5411.3(10)	26.6(6)
C33	7203(2)	6196(2)	6432.9(10)	26.7(6)
C34	7742(2)	5367(2)	6543.3(11)	30.5(6)
C35	7737(2)	4700(2)	6173.4(12)	35.5(7)
C36	7213(2)	4887(2)	5695.8(11)	35.2(7)
C37	6685(2)	5726(2)	5607.7(10)	28.9(6)
C38	5989(3)	5521(3)	4671.0(11)	42.0(8)
C39	6101(2)	6015(2)	5122.0(10)	31.2(6)
C40	5379(3)	5928(3)	4259.1(11)	46.9(9)
C41	4882(3)	6781(3)	4296.4(11)	44.7(9)
C42	5023(2)	7244(2)	4756.6(10)	34.4(7)
C43	4560(2)	8127(2)	4891.9(10)	34.8(7)
C44	3880(2)	8681(3)	4568.6(12)	43.5(8)
C45	3455(3)	9476(3)	4753.3(13)	49.4(9)
C46	3741(3)	9718(2)	5252.9(13)	44.4(8)
C47	4454(2)	9141(2)	5553.5(12)	35.1(7)
Co1	3889.7(3)	7547.9(3)	6801.7(2)	21.83(11)
Co2	5771.6(3)	7456.8(3)	5769.9(2)	23.29(11)
N1	4149.2(17)	6259.7(15)	6962.3(8)	24.0(5)
N2	2435.1(17)	7190.4(15)	6794.9(8)	23.7(5)
N3	3208.1(17)	8771.0(16)	6686.1(8)	26.2(5)
N4	4255.0(17)	7903.9(15)	7514.4(8)	23.9(5)
N5	5400.4(17)	8783.0(16)	8062.5(8)	26.1(5)
N6	5348.9(16)	7951.5(15)	6792.2(8)	22.5(4)
N7	5925.5(16)	8048.6(15)	6405.2(8)	22.6(5)

Atom	*x*	*y*	*z*	U(eq)
N8	7125.1(17)	8190.5(16)	5725.7(8)	25.0(5)
N9	8495.5(17)	9053.0(17)	6160.2(8)	28.9(5)
N10	6657.2(17)	6371.8(16)	5976.4(8)	25.5(5)
N11	5641.7(18)	6853.9(17)	5150.1(8)	29.5(5)
N12	4844.0(18)	8361.7(17)	5384.4(8)	29.7(5)
O1	3729.8(15)	7334.1(14)	6095.7(7)	29.1(4)
O2	4543.1(15)	6774.8(13)	5938.8(7)	29.6(4)
C48	1361(3)	7947(3)	3932.2(16)	53.1(10)
C49	962(3)	8529(3)	3510.2(16)	54.9(10)
N13	1684(4)	7484(3)	4271.4(15)	75.2(11)
F1	-10.5(16)	7128.0(16)	8182.2(10)	70.8(7)
F2	-1242(2)	6071.2(17)	8382.7(8)	75.5(7)
F3	-1761.6(16)	7534.4(15)	8207.4(8)	57.7(6)
F4	-2303.5(16)	6395.0(19)	7654.4(8)	72.0(7)
F5	-1086(2)	7433.1(16)	7462.8(9)	72.1(7)
F6	-548.0(15)	5957.1(13)	7651.9(7)	45.6(4)
P1	-1156.7(6)	6751.8(6)	7925.8(3)	38.2(2)

Table S31. Anisotropic Displacement Parameters ($Å^2 \times 10^3$) for **1-I**.

Atom	U_{11}	U_{22}	U_{33}	U_{23}	U_{13}	U_{12}
C1	23.4(13)	31.6(15)	30.5(14)	3.6(12)	0.7(11)	0.0(11)
C2	26.0(14)	35.2(16)	35.8(15)	3.1(13)	-0.3(11)	3.5(12)
C3	35.4(15)	25.5(15)	37.1(16)	3.3(12)	3.0(12)	0.3(12)
C4	28.3(14)	27.3(15)	33.9(15)	-0.4(12)	4.0(11)	-4.3(12)
C5	25.0(13)	26.4(14)	22.6(13)	-2.2(11)	3.3(10)	-3.6(11)
C6	23.3(13)	28.0(14)	22.3(13)	-2.1(11)	0.6(10)	-2.0(11)
C7	25.8(14)	30.3(15)	34.5(15)	-1.7(12)	3.2(11)	-6.3(12)
C8	20.5(13)	38.8(17)	38.0(16)	-3.5(13)	2.6(11)	-4.8(12)
C9	20.2(13)	37.2(17)	31.6(15)	-1.0(12)	-0.4(11)	0.9(11)
C10	22.2(13)	31.0(15)	24.5(13)	-1.8(11)	-1.4(10)	1.9(11)
C11	23.6(13)	29.8(15)	24.4(13)	0.7(11)	-1.8(10)	0.8(11)
C12	26.7(14)	31.0(15)	34.0(15)	-2.1(12)	-2.4(11)	4.4(12)
C13	35.1(16)	28.2(16)	44.1(17)	-1.5(13)	-2.8(13)	5.5(13)
C14	36.2(16)	27.1(15)	41.7(17)	0.9(13)	-4.0(13)	-3.9(13)
C15	28.5(14)	26.5(15)	34.3(15)	2.6(12)	-2.2(11)	-1.2(12)
C16	19.0(12)	25.5(14)	23.9(13)	1.6(11)	0.3(10)	2.1(10)
C17	24.6(13)	24.3(14)	27.0(13)	1.2(11)	3.7(10)	1.5(11)
C18	31.5(15)	33.6(16)	30.2(15)	-1.2(12)	4.8(12)	-0.3(12)
C19	36.6(15)	34.0(16)	32.0(15)	1.3(13)	12.5(12)	1.2(13)
C20	28.8(14)	29.1(15)	38.2(16)	4.7(13)	11.4(12)	2.2(12)
C21	24.8(14)	22.3(14)	34.5(15)	2.6(11)	4.2(11)	0.6(11)
C22	23.0(13)	22.2(13)	27.7(13)	1.8(11)	3.0(10)	4.0(11)
C23	19.4(12)	26.0(14)	21.9(12)	0.3(10)	-2.1(10)	2.8(10)
C24	19.1(12)	27.1(14)	26.3(13)	0.3(11)	-1.9(10)	-2.3(11)
C25	17.9(12)	22.6(13)	27.6(13)	2.1(11)	-1.0(10)	-0.4(10)
C26	20.0(12)	25.3(14)	23.9(13)	1.2(11)	-1.6(10)	1.0(10)
C27	26.2(14)	40.4(17)	29.1(14)	0.5(13)	3.0(11)	-5.0(12)
C28	31.9(16)	79(3)	37.8(17)	-11.9(17)	7.8(13)	-21.9(17)
C29	40.6(19)	92(3)	42.9(19)	-17(2)	18.1(15)	-26(2)
C30	39.8(17)	59(2)	31.0(16)	-6.9(15)	11.3(13)	-11.0(16)
C31	32.6(15)	38.6(17)	27.6(14)	-1.3(13)	3.3(11)	-5.7(13)
C32	23.5(13)	29.2(15)	26.8(13)	2.8(11)	1.9(10)	-2.7(11)
C33	22.7(13)	31.2(15)	25.7(13)	-0.8(11)	1.1(10)	-2.9(11)
C34	21.7(13)	35.2(16)	34.0(15)	1.8(12)	1.6(11)	-0.6(12)
C35	26.9(14)	30.0(16)	48.6(18)	-4.9(13)	0.9(13)	2.0(12)
C36	30.1(15)	36.5(17)	39.1(16)	-9.7(13)	4.6(12)	-3.6(13)
C37	28.3(14)	31.3(15)	27.4(14)	-5.2(12)	4.9(11)	-8.1(12)
C38	41.3(17)	55(2)	30.5(16)	-10.7(15)	9.3(13)	-17.0(16)

Atom	U_{11}	U_{22}	U_{33}	U_{23}	U_{13}	U_{12}
C39	31.1(14)	36.8(17)	27.1(14)	-3.3(12)	8.7(11)	-11.7(13)
C40	46.7(19)	70(3)	24.7(15)	-11.7(16)	8.8(13)	-24.2(18)
C41	41.8(18)	71(3)	20.9(14)	4.6(15)	0.7(12)	-25.4(17)
C42	29.4(15)	49.2(19)	23.5(14)	6.3(13)	-1.0(11)	-15.7(13)
C43	26.6(14)	48.2(19)	27.7(14)	10.7(13)	-3.7(11)	-13.6(13)
C44	33.8(16)	59(2)	34.9(17)	19.9(16)	-8.2(13)	-12.2(16)
C45	33.6(17)	59(2)	52(2)	29.0(18)	-7.2(15)	-5.5(16)
C46	34.6(16)	40.9(19)	56(2)	19.2(16)	1.4(15)	2.4(14)
C47	30.2(15)	34.3(17)	39.7(17)	9.7(13)	0.7(12)	-2.7(13)
Co1	17.4(2)	23.1(2)	24.2(2)	0.71(17)	-0.65(16)	-2.04(17)
Co2	22.5(2)	27.2(2)	19.2(2)	1.88(17)	-0.98(16)	-4.60(18)
N1	21.8(11)	27.4(12)	22.6(11)	-1.4(9)	2.1(8)	-1.9(9)
N2	22.0(11)	26.5(12)	21.9(11)	-0.6(9)	-0.4(8)	-1.7(9)
N3	22.5(11)	27.7(12)	27.1(11)	1.3(10)	-2.7(9)	0.5(9)
N4	20.1(10)	24.5(12)	26.7(11)	1.2(9)	1.2(9)	-0.1(9)
N5	23.6(11)	30.3(13)	24.0(11)	-0.8(9)	1.9(9)	-0.9(9)
N6	19.4(10)	25.2(12)	22.1(11)	1.3(9)	0.0(8)	-0.4(9)
N7	19.4(10)	24.9(12)	23.0(11)	2.4(9)	0.4(8)	-1.8(9)
N8	23.7(11)	28.7(12)	22.1(11)	1.8(9)	0.8(9)	-3.1(9)
N9	20.6(11)	37.8(14)	28.1(12)	-1.4(10)	2.8(9)	-4.8(10)
N10	23.6(11)	29.0(12)	24.1(11)	-0.7(9)	3.5(9)	-5.5(9)
N11	28.3(12)	38.9(14)	20.7(11)	1.5(10)	0.8(9)	-12.9(10)
N12	24.0(11)	35.5(14)	28.4(12)	8.9(10)	-1.5(9)	-7.2(10)
O1	22.8(9)	36.6(11)	26.6(10)	2.6(8)	-1.4(8)	-3.2(8)
O2	31.8(10)	30.4(11)	26.7(10)	-2.2(8)	4.5(8)	-5.8(8)
C48	43(2)	58(2)	62(2)	-27(2)	17.9(18)	-16.8(18)
C49	30.2(17)	51(2)	84(3)	-9(2)	7.7(17)	-1.5(16)
N13	85(3)	82(3)	58(2)	-17(2)	7(2)	-34(2)
F1	32.0(10)	60.9(14)	115(2)	-34.2(14)	-10.3(11)	0.3(10)
F2	121(2)	75.2(16)	32.9(11)	2.6(11)	18.2(12)	-8.4(15)
F3	40.6(11)	79.0(16)	53.7(12)	-26.7(11)	6.0(9)	5.7(10)
F4	39.0(11)	116(2)	58.5(13)	-40.1(14)	-3.0(9)	-8.2(12)
F5	86.1(17)	68.9(16)	68.2(15)	21.9(12)	36.8(13)	37.0(13)
F6	49.4(11)	46.8(11)	40.3(10)	-3.4(8)	4.4(8)	5.7(9)
P1	31.3(4)	50.4(5)	32.4(4)	-7.0(4)	2.4(3)	-3.0(4)

Table S32. Bond Lengths for **1-I**.

Atom	Atom	Length/Å	Atom	Atom	Length/Å
C1	C2	1.384(4)	C31	C32	1.400(4)
C1	N1	1.336(3)	C32	N8	1.373(3)
C2	C3	1.377(4)	C33	C34	1.383(4)
C3	C4	1.387(4)	C33	N10	1.346(3)
C4	C5	1.374(4)	C34	C35	1.382(4)
C5	C6	1.469(4)	C35	C36	1.387(4)
C5	N1	1.363(3)	C36	C37	1.382(4)
C6	C7	1.392(4)	C37	C39	1.469(4)
C6	N2	1.337(3)	C37	N10	1.363(3)
C7	C8	1.380(4)	C38	C39	1.396(4)
C8	C9	1.379(4)	C38	C40	1.387(5)
C9	C10	1.391(4)	C39	N11	1.343(4)
C10	C11	1.460(4)	C40	C41	1.385(5)
C10	N2	1.346(3)	C41	C42	1.396(4)
C11	C12	1.383(4)	C42	C43	1.462(5)
C11	N3	1.363(3)	C42	N11	1.346(4)
C12	C13	1.383(4)	C43	C44	1.384(4)
C13	C14	1.383(4)	C43	N12	1.366(4)
C14	C15	1.382(4)	C44	C45	1.378(5)
C15	N3	1.345(4)	C45	C46	1.387(5)

Atom	Atom	Length/Å	Atom	Atom	Length/Å
C16	C23	1.459(4)	C46	C47	1.392(4)
C16	N4	1.373(3)	C47	N12	1.326(4)
C16	N5	1.323(3)	Co1	N1	1.928(2)
C17	C18	1.399(4)	Co1	N2	1.861(2)
C17	C22	1.419(4)	Co1	N3	1.964(2)
C17	N5	1.387(3)	Co1	N4	1.977(2)
C18	C19	1.384(4)	Co1	N6	1.891(2)
C19	C20	1.403(4)	Co1	O1	1.9044(19)
C20	C21	1.386(4)	Co2	N7	1.895(2)
C21	C22	1.402(4)	Co2	N8	1.990(2)
C22	N4	1.377(3)	Co2	N10	1.950(2)
C23	C24	1.395(4)	Co2	N11	1.866(2)
C23	N6	1.355(3)	Co2	N12	1.947(2)
C24	C25	1.397(4)	Co2	O2	1.9058(19)
C25	C26	1.458(4)	N6	N7	1.335(3)
C25	N7	1.358(3)	O1	O2	1.392(3)
C26	N8	1.369(3)	C48	C49	1.446(6)
C26	N9	1.333(3)	C48	N13	1.159(6)
C27	C28	1.395(4)	F1	P1	1.587(2)
C27	C32	1.417(4)	F2	P1	1.585(2)
C27	N9	1.393(4)	F3	P1	1.595(2)
C28	C29	1.382(5)	F4	P1	1.592(2)
C29	C30	1.395(5)	F5	P1	1.595(2)
C30	C31	1.378(4)	F6	P1	1.599(2)

Table S33. Bond Angles for **1-I**.

Atom	Atom	Atom	Angle/˚	Atom	Atom	Atom	Angle/˚
N1	C1	C2	122.1(3)	N1	Co1	N4	91.35(9)
C3	C2	C1	119.3(3)	N2	Co1	N1	82.34(9)
C2	C3	C4	118.9(3)	N2	Co1	N3	81.76(9)
C5	C4	C3	119.3(3)	N2	Co1	N4	100.66(9)
C4	C5	C6	125.5(2)	N2	Co1	N6	177.74(10)
N1	C5	C4	121.6(2)	N2	Co1	O1	87.96(9)
N1	C5	C6	112.9(2)	N3	Co1	N4	87.71(9)
C7	C6	C5	128.4(3)	N6	Co1	N1	99.75(9)
N2	C6	C5	111.8(2)	N6	Co1	N3	96.21(9)
N2	C6	C7	119.9(2)	N6	Co1	N4	80.21(9)
C8	C7	C6	118.0(3)	N6	Co1	O1	91.04(9)
C9	C8	C7	121.7(3)	O1	Co1	N1	93.41(9)
C8	C9	C10	118.0(3)	O1	Co1	N3	89.97(9)
C9	C10	C11	128.0(3)	O1	Co1	N4	170.65(9)
N2	C10	C9	119.7(3)	N7	Co2	N8	79.75(9)
N2	C10	C11	112.2(2)	N7	Co2	N10	96.74(9)
C12	C11	C10	124.8(2)	N7	Co2	N12	99.28(10)
N3	C11	C10	113.1(2)	N7	Co2	O2	90.78(8)
N3	C11	C12	122.0(3)	N10	Co2	N8	90.15(9)
C11	C12	C13	119.0(3)	N11	Co2	N7	178.74(10)
C14	C13	C12	119.3(3)	N11	Co2	N8	100.11(9)
C15	C14	C13	119.1(3)	N11	Co2	N10	82.01(10)
N3	C15	C14	122.4(3)	N11	Co2	N12	81.97(11)
N4	C16	C23	113.6(2)	N11	Co2	O2	89.28(9)
N5	C16	C23	130.1(2)	N12	Co2	N8	92.91(9)
N5	C16	N4	116.3(2)	N12	Co2	N10	163.98(10)
C18	C17	C22	120.2(2)	O2	Co2	N8	169.75(8)
N5	C17	C18	129.8(2)	O2	Co2	N10	87.07(9)
N5	C17	C22	110.0(2)	O2	Co2	N12	92.47(9)
C19	C18	C17	118.1(3)	C1	N1	C5	118.8(2)
C18	C19	C20	121.3(3)	C1	N1	Co1	126.81(19)

Atom	Atom	Atom	Angle/˚	Atom	Atom	Atom	Angle/˚
C21	C20	C19	121.8(3)	C5	N1	Co1	114.38(17)
C20	C21	C22	117.1(3)	C6	N2	C10	122.7(2)
C21	C22	C17	121.4(2)	C6	N2	Co1	118.39(18)
N4	C22	C17	106.8(2)	C10	N2	Co1	118.65(19)
N4	C22	C21	131.9(2)	C11	N3	Co1	113.98(18)
C24	C23	C16	139.8(2)	C15	N3	C11	118.1(2)
N6	C23	C16	111.0(2)	C15	N3	Co1	127.63(18)
N6	C23	C24	109.0(2)	C16	N4	C22	104.0(2)
C23	C24	C25	104.4(2)	C16	N4	Co1	114.21(17)
C24	C25	C26	140.1(2)	C22	N4	Co1	140.02(17)
N7	C25	C24	109.2(2)	C16	N5	C17	102.9(2)
N7	C25	C26	110.7(2)	C23	N6	Co1	119.50(17)
N8	C26	C25	113.8(2)	N7	N6	C23	109.0(2)
N9	C26	C25	129.8(2)	N7	N6	Co1	129.77(16)
N9	C26	N8	116.3(2)	C25	N7	Co2	120.34(17)
C28	C27	C32	119.9(3)	N6	N7	C25	108.4(2)
N9	C27	C28	129.8(3)	N6	N7	Co2	130.39(17)
N9	C27	C32	110.3(2)	C26	N8	C32	104.4(2)
C29	C28	C27	118.2(3)	C26	N8	Co2	114.76(17)
C28	C29	C30	121.6(3)	C32	N8	Co2	140.14(18)
C31	C30	C29	121.5(3)	C26	N9	C27	102.2(2)
C30	C31	C32	117.5(3)	C33	N10	C37	118.4(2)
C31	C32	C27	121.3(2)	C33	N10	Co2	127.49(19)
N8	C32	C27	106.7(2)	C37	N10	Co2	114.06(18)
N8	C32	C31	132.0(2)	C39	N11	C42	122.3(3)
N10	C33	C34	122.0(3)	C39	N11	Co2	118.74(18)
C35	C34	C33	119.4(3)	C42	N11	Co2	118.7(2)
C34	C35	C36	119.2(3)	C43	N12	Co2	114.2(2)
C37	C36	C35	118.9(3)	C47	N12	C43	118.8(3)
C36	C37	C39	124.7(3)	C47	N12	Co2	127.0(2)
N10	C37	C36	122.0(3)	O2	O1	Co1	113.89(13)
N10	C37	C39	113.3(3)	O1	O2	Co2	113.16(14)
C40	C38	C39	117.3(3)	N13	C48	C49	179.6(5)
C38	C39	C37	127.4(3)	F1	P1	F3	89.66(11)
N11	C39	C37	111.7(2)	F1	P1	F4	178.25(16)
N11	C39	C38	120.8(3)	F1	P1	F5	89.68(15)
C41	C40	C38	121.3(3)	F1	P1	F6	90.18(11)
C40	C41	C42	118.8(3)	F2	P1	F1	91.02(15)
C41	C42	C43	128.9(3)	F2	P1	F3	89.67(13)
N11	C42	C41	119.3(3)	F2	P1	F4	90.73(15)
N11	C42	C43	111.8(3)	F2	P1	F5	179.29(16)
C44	C43	C42	125.0(3)	F2	P1	F6	89.39(12)
N12	C43	C42	113.3(2)	F3	P1	F5	90.28(12)
N12	C43	C44	121.7(3)	F3	P1	F6	179.04(12)
C45	C44	C43	118.9(3)	F4	P1	F3	90.38(12)
C44	C45	C46	119.7(3)	F4	P1	F5	88.56(15)
C45	C46	C47	118.3(3)	F4	P1	F6	89.82(11)
N12	C47	C46	122.6(3)	F5	P1	F6	90.66(11)
N1	Co1	N3	163.62(9)				

Table S34. Torsion Angles for **1-I**.

A	B	C	D	Angle/˚	A	B	C	D	Angle/˚
C1	C2	C3	C4	-0.9(4)	C37	C39	N11	C42	-177.0(2)
C2	C1	N1	C5	2.5(4)	C37	C39	N11	Co2	-2.1(3)
C2	C1	N1	Co1	179.6(2)	C38	C39	N11	C42	2.6(4)
C2	C3	C4	C5	1.3(4)	C38	C39	N11	Co2	177.5(2)
C3	C4	C5	C6	-177.7(3)	C38	C40	C41	C42	1.3(5)
C3	C4	C5	N1	0.2(4)	C39	C37	N10	C33	-177.5(2)

A	B	C	D	Angle/˚	A	B	C	D	Angle/˚
C4	C5	C6	C7	-3.1(4)	C39	C37	N10	Co2	3.9(3)
C4	C5	C6	N2	175.7(3)	C39	C38	C40	C41	-1.2(5)
C4	C5	N1	C1	-2.1(4)	C40	C38	C39	C37	178.8(3)
C4	C5	N1	Co1	-179.5(2)	C40	C38	C39	N11	-0.7(4)
C5	C6	C7	C8	-179.9(3)	C40	C41	C42	C43	-177.9(3)
C5	C6	N2	C10	179.2(2)	C40	C41	C42	N11	0.5(4)
C5	C6	N2	Co1	5.3(3)	C41	C42	C43	C44	-0.6(5)
C6	C5	N1	C1	176.1(2)	C41	C42	C43	N12	178.0(3)
C6	C5	N1	Co1	-1.3(3)	C41	C42	N11	C39	-2.5(4)
C6	C7	C8	C9	-0.1(4)	C41	C42	N11	Co2	-177.4(2)
C7	C6	N2	C10	-1.9(4)	C42	C43	C44	C45	176.7(3)
C7	C6	N2	Co1	-175.79(19)	C42	C43	N12	C47	-178.6(2)
C7	C8	C9	C10	-0.6(4)	C42	C43	N12	Co2	-0.5(3)
C8	C9	C10	C11	-178.5(3)	C43	C42	N11	C39	176.2(2)
C8	C9	C10	N2	0.2(4)	C43	C42	N11	Co2	1.3(3)
C9	C10	C11	C12	5.3(5)	C43	C44	C45	C46	1.9(5)
C9	C10	C11	N3	-177.6(3)	C44	C43	N12	C47	0.1(4)
C9	C10	N2	C6	1.1(4)	C44	C43	N12	Co2	178.2(2)
C9	C10	N2	Co1	175.0(2)	C44	C45	C46	C47	-0.2(5)
C10	C11	C12	C13	176.5(3)	C45	C46	C47	N12	-1.7(5)
C10	C11	N3	C15	-174.5(2)	C46	C47	N12	C43	1.8(4)
C10	C11	N3	Co1	0.1(3)	C46	C47	N12	Co2	-176.1(2)
C11	C10	N2	C6	180.0(2)	Co1	N6	N7	C25	165.94(18)
C11	C10	N2	Co1	-6.1(3)	Co1	N6	N7	Co2	-24.9(3)
C11	C12	C13	C14	-1.8(4)	Co1	O1	O2	Co2	-90.39(14)
C12	C11	N3	C15	2.7(4)	N1	C1	C2	C3	-1.0(4)
C12	C11	N3	Co1	177.4(2)	N1	C5	C6	C7	178.8(3)
C12	C13	C14	C15	1.7(5)	N1	C5	C6	N2	-2.4(3)
C13	C14	C15	N3	0.7(5)	N1	Co1	N2	C6	-4.88(19)
C14	C15	N3	C11	-2.9(4)	N1	Co1	N2	C10	-179.0(2)
C14	C15	N3	Co1	-176.7(2)	N1	Co1	N6	C23	-100.9(2)
C16	C23	C24	C25	-174.5(3)	N1	Co1	N6	N7	95.9(2)
C16	C23	N6	Co1	8.9(3)	N2	C6	C7	C8	1.4(4)
C16	C23	N6	N7	175.4(2)	N2	C10	C11	C12	-173.5(3)
C17	C18	C19	C20	1.2(4)	N2	C10	C11	N3	3.7(3)
C17	C22	N4	C16	-0.1(3)	N3	C11	C12	C13	-0.4(4)
C17	C22	N4	Co1	-163.0(2)	N3	Co1	N2	C6	179.1(2)
C18	C17	C22	C21	-1.1(4)	N3	Co1	N2	C10	4.98(19)
C18	C17	C22	N4	178.4(2)	N3	Co1	N6	C23	75.4(2)
C18	C17	N5	C16	-178.2(3)	N3	Co1	N6	N7	-87.8(2)
C18	C19	C20	C21	-1.1(4)	N4	C16	C23	C24	175.0(3)
C19	C20	C21	C22	-0.1(4)	N4	C16	C23	N6	0.8(3)
C20	C21	C22	C17	1.2(4)	N4	C16	N5	C17	0.0(3)
C20	C21	C22	N4	-178.2(3)	N4	Co1	N2	C6	-94.8(2)
C21	C22	N4	C16	179.4(3)	N4	Co1	N2	C10	91.1(2)
C21	C22	N4	Co1	16.4(5)	N4	Co1	N6	C23	-11.18(19)
C22	C17	C18	C19	-0.1(4)	N4	Co1	N6	N7	-174.4(2)
C22	C17	N5	C16	0.0(3)	N5	C16	C23	C24	-2.0(5)
C23	C16	N4	C22	-177.4(2)	N5	C16	C23	N6	-176.3(3)
C23	C16	N4	Co1	-9.3(3)	N5	C16	N4	C22	0.0(3)
C23	C16	N5	C17	177.0(3)	N5	C16	N4	Co1	168.14(18)
C23	C24	C25	C26	-176.2(3)	N5	C17	C18	C19	177.8(3)
C23	C24	C25	N7	1.0(3)	N5	C17	C22	C21	-179.5(2)
C23	N6	N7	C25	1.3(3)	N5	C17	C22	N4	0.1(3)
C23	N6	N7	Co2	170.48(18)	N6	C23	C24	C25	-0.2(3)
C24	C23	N6	Co1	-167.15(17)	N7	C25	C26	N8	-0.4(3)
C24	C23	N6	N7	-0.7(3)	N7	C25	C26	N9	-178.2(3)
C24	C25	C26	N8	176.8(3)	N8	C26	N9	C27	-0.5(3)
C24	C25	C26	N9	-1.0(6)	N8	Co2	N7	C25	-7.14(19)

A	B	C	D	Angle/˚	A	B	C	D	Angle/˚
C24	C25	N7	Co2	-171.89(17)	N8	Co2	N7	N6	-175.2(2)
C24	C25	N7	N6	-1.4(3)	N8	Co2	N11	C39	92.1(2)
C25	C26	N8	C32	-177.6(2)	N8	Co2	N11	C42	-92.8(2)
C25	C26	N8	Co2	-5.1(3)	N9	C26	N8	C32	0.5(3)
C25	C26	N9	C27	177.2(3)	N9	C26	N8	Co2	173.04(19)
C26	C25	N7	Co2	6.2(3)	N9	C27	C28	C29	179.3(4)
C26	C25	N7	N6	176.7(2)	N9	C27	C32	C31	179.6(3)
C27	C28	C29	C30	1.3(6)	N9	C27	C32	N8	0.0(3)
C27	C32	N8	C26	-0.3(3)	N10	C33	C34	C35	0.9(4)
C27	C32	N8	Co2	-169.6(2)	N10	C37	C39	C38	179.1(3)
C28	C27	C32	C31	-0.8(5)	N10	C37	C39	N11	-1.3(3)
C28	C27	C32	N8	179.6(3)	N10	Co2	N7	C25	81.8(2)
C28	C27	N9	C26	-179.3(4)	N10	Co2	N7	N6	-86.3(2)
C28	C29	C30	C31	-1.2(6)	N10	Co2	N11	C39	3.4(2)
C29	C30	C31	C32	0.1(5)	N10	Co2	N11	C42	178.4(2)
C30	C31	C32	C27	0.9(4)	N11	C42	C43	C44	-179.2(3)
C30	C31	C32	N8	-179.6(3)	N11	C42	C43	N12	-0.5(3)
C31	C32	N8	C26	-179.8(3)	N12	C43	C44	C45	-1.9(4)
C31	C32	N8	Co2	10.8(5)	N12	Co2	N7	C25	-98.4(2)
C32	C27	C28	C29	-0.3(5)	N12	Co2	N7	N6	93.5(2)
C32	C27	N9	C26	0.3(3)	N12	Co2	N11	C39	-176.3(2)
C33	C34	C35	C36	1.6(4)	N12	Co2	N11	C42	-1.3(2)
C34	C33	N10	C37	-3.2(4)	O1	Co1	N2	C6	88.8(2)
C34	C33	N10	Co2	175.29(19)	O1	Co1	N2	C10	-85.3(2)
C34	C35	C36	C37	-1.8(4)	O1	Co1	N6	C23	165.5(2)
C35	C36	C37	C39	179.9(3)	O1	Co1	N6	N7	2.2(2)
C35	C36	C37	N10	-0.5(4)	O2	Co2	N7	C25	168.9(2)
C36	C37	C39	C38	-1.3(5)	O2	Co2	N7	N6	0.9(2)
C36	C37	C39	N11	178.3(3)	O2	Co2	N11	C39	-83.7(2)
C36	C37	N10	C33	3.0(4)	O2	Co2	N11	C42	91.3(2)
C36	C37	N10	Co2	-175.7(2)					

Table S35. Hydrogen Atom Coordinates (Å×10^4) and Isotropic Displacement Parameters (Å^2×10^3) for **1-I**.

Atom	*x*	*y*	*z*	U(eq)
H1	5765.57	6215.15	7097.65	34
H2	5913.18	4643.65	7279.64	39
H3	4325.16	3747.31	7260.85	39
H4	2611.4	4467.68	7075	36
H7	964.08	5362.22	6867.85	36
H8	-426.32	6451.39	6683.44	39
H9	-18.21	8003.97	6560.88	36
H12	698.6	9529.5	6541.45	37
H13	1598.26	10956.35	6511.69	44
H14	3517.16	10994.21	6631.24	43
H15	4488.42	9617	6740.54	36
H18	4654.85	9150.08	8981.92	38
H19	2963.26	8646.78	9190.6	40
H20	1814.86	7722.28	8642.03	38
H21	2286.64	7304.67	7852.18	33
H24	7343.58	9171.44	7266.39	29
H28	10250.66	9449.37	5631.42	59
H29	10372.14	8935.78	4815.32	69
H30	8965.17	8092.87	4370.22	51
H31	7405.81	7683.52	4734.89	40
H33	7218.95	6655.31	6687.16	32
H34	8112.79	5257.19	6870.14	37

Atom	*x*	*y*	*z*	U(eq)
H35	8087.75	4121.36	6245.35	43
H36	7217.43	4445.16	5433.65	42
H38	6318.07	4930.52	4647.13	50
H40	5299.32	5614.65	3945.21	56
H41	4452.93	7046.88	4013.47	54
H44	3707.81	8516.93	4224.89	52
H45	2969.29	9855.7	4539.58	59
H46	3458.54	10263.84	5386.49	53
H47	4668.43	9312.15	5893.4	42
H49A	1373.72	8397.8	3228.16	82
H49B	1057.65	9180.61	3607.07	82
H49C	182.93	8402.94	3409.48	82

Table S36. Solvent masks information for **1-I**.

Number	X	Y	Z	Volume	Electron count	Content
1	0.000	0.500	0.500	242.6	59.6	?
2	0.500	0.000	1.000	242.6	59.6	?

18d. Single crystal X-ray crystallography for **3-I**.

Data Collection

Single crystals suitable for X-ray analysis were obtained by diffusion of diethyl ether into a saturated anhydrous CH_3CN solution of **3** with 2.5 equiv NaH. A blackish violet crystal with approximate dimensions 0.11 × 0.08 × 0.07 mm^3 was selected under oil under ambient conditions and attached to the tip of a MiTeGen MicroMount©. The crystal was mounted in a cold nitrogen stream at 100 K and centered in the X-ray beam using a video camera. The crystal evaluation and data collection were performed on a Rigaku XtaLAB Synergy R, DW system, HyPix-Arc 150 with Cu Kα (λ = 1.54184 Å) radiation, and the diffractometer.

The initial cell constants were obtained from ω scans consisting of 12 frames collected at intervals of 0.5º in a 10º range of about ω with an exposure time of 5 s per frame. The reflections were successfully indexed by an automated indexing routine built into the CrysAlisPro program suite. The final cell constants were calculated from a set of 45851 strong reflections from the actual data collection. The data were collected using a Laue symmetry data collection routine to survey the reciprocal space to the extent of a full sphere to a resolution of 0.84 Å. A total of 72219 data were harvested by collecting 21 sets of frames with 0.5º scans in ω and φ with exposure times of 4.87 sec per frame. These highly redundant datasets were corrected for Lorentz and polarization effects. The absorption correction was based on fitting a function to the empirical transmission surface sampled by multiple equivalent measurements.

Structure Solution and Refinement

The systematic absences in the diffraction data were consistent for the space group $I4_1cd$, which yielded chemically reasonable and computationally stable refinement results.

A successful solution by charge-flipping provided most non-hydrogen atoms from the E-map. The remaining non-hydrogen atoms were located in an alternating series of least-squares cycles and difference Fourier maps. All non-hydrogen atoms were refined with anisotropic displacement coefficients. All hydrogen atoms were included in the structure factor calculation at idealized positions and were allowed to ride on the neighboring atoms with relative isotropic displacement coefficients.

The asymmetric unit consists of one molecule of the cobalt complex, one molecule of PF_6^-, one of KPF_6, one of diethyl ether, and six molecules of H_2O. The final least-squares refinement of 833 parameters against 8800 data resulted in residuals R (based on F2 for I≥2σ) and wR (based on F2 for I≥2σ) of 0.0371 and 0.1012, respectively.

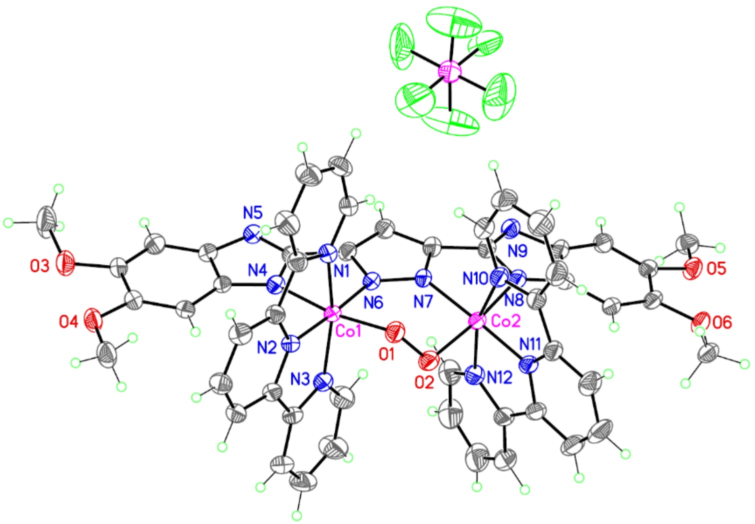

Figure S94. ORTEP drawings of **3-I** with thermal ellipsoids at 50% probability.

Table S37. Crystal data and structure refinement for **3-I**.

Identification code	230155lt2_auto
Empirical formula	$C_{55}H_{56}Co_2F_9K_{0.5}N_{12}O_{10.5}P_{1.5}$
Formula weight	1407.98
Temperature/K	99.99(11)
Crystal system	tetragonal
Space group	$I4_1cd$
a/Å	23.42250(10)
b/Å	23.42250(10)
c/Å	42.4296(3)
α/°	90
β/°	90
γ/°	90
Volume/Å^3	23277.5(3)
Z	16
ρ_{calc}g/cm^3	1.607
μ/mm^{-1}	6.052
F(000)	11536.0
Crystal size/mm^3	0.11 × 0.08 × 0.07
Radiation	Cu Kα (λ = 1.54184)
2Θ range for data collection/°	6.77 to 134.146
Index ranges	-27 ≤ h ≤ 27, -27 ≤ k ≤ 27, -50 ≤ l ≤ 28
Reflections collected	72219
Independent reflections	8800 [R_{int} = 0.0301, R_{sigma} = 0.0178]
Data/restraints/parameters	8800/2/833
Goodness-of-fit on F^2	1.038
Final R indexes [I>=2σ (I)]	R_1 = 0.0371, wR_2 = 0.1012
Final R indexes [all data]	R_1 = 0.0387, wR_2 = 0.1024
Largest diff. peak/hole / e Å^{-3}	0.68/-0.73
Flack parameter	-0.0318(17)

Table S38. Fractional Atomic Coordinates (×10^4) and Equivalent Isotropic Displacement Parameters (Å^2×10^3) for **3-I**.

Atom	*x*	*y*	*z*	U(eq)
C1	7585.7(19)	2820(2)	4796.8(12)	29.1(10)
C2	7266(2)	2737(2)	4531.7(14)	37.8(12)
C3	7092(2)	2188(2)	4450.9(15)	41.3(13)
C4	7245(2)	1744(2)	4645.8(15)	39.1(12)
C5	7566.5(19)	1856.7(19)	4913.0(12)	27.7(10)
C6	7773.3(18)	1419.1(19)	5132.1(12)	26.0(9)
C7	7698(2)	830(2)	5108.9(13)	32.5(11)
C8	7975(2)	487.5(19)	5327.7(13)	34.2(11)
C9	8303.5(19)	724.5(19)	5565.8(12)	29.6(10)
C10	8344.7(18)	1313.9(19)	5582.1(12)	26.8(9)
C11	8680(2)	1653.8(19)	5805.0(12)	28.6(10)
C12	8983(2)	1441(2)	6055.4(13)	35.6(11)
C13	9293(3)	1809(2)	6243.8(14)	45.9(14)
C14	9297(3)	2383(2)	6168.7(16)	50.9(16)
C15	8988(2)	2574(2)	5910.0(14)	41.1(13)
C16	9136.2(18)	2930.1(17)	5014.3(11)	23.5(9)
C17	9276.5(18)	2029.4(18)	4952.7(11)	23.1(9)
C18	9271.5(18)	1429.7(18)	4957.8(11)	24.8(9)
C19	9673.1(19)	1152.7(19)	4777.7(12)	28.0(10)
C20	10086(2)	1460(2)	4602.0(12)	32.1(11)
C21	10104(2)	2050(2)	4602.8(12)	32.9(11)
C22	9688.3(19)	2338.7(18)	4783.4(11)	25.9(9)
C23	10785(3)	1366(3)	4197.5(19)	61(2)
C24	9312(2)	243(2)	4933.3(16)	43.2(14)
C25	8832.2(18)	3413.3(18)	5156.4(11)	24.1(9)
C26	8850.5(19)	4009.3(19)	5165.5(12)	27.2(9)
C27	8414.1(19)	4156.7(18)	5372.7(12)	27.9(9)
C28	8201.1(19)	4655.1(18)	5541.9(12)	26.5(9)
C29	8084.6(19)	5444.1(19)	5782.0(11)	26.0(9)
C30	8107.2(19)	6010.4(18)	5893.6(11)	26.3(9)
C31	7757.5(19)	6150.6(18)	6140.9(11)	25.7(9)
C32	7376.0(18)	5745.4(19)	6278.6(11)	25.6(9)
C33	7353.5(18)	5194.7(18)	6170.4(11)	24.5(9)
C34	7720.7(19)	5047.6(18)	5919.2(11)	25.3(9)
C35	8188(2)	7069.1(19)	6189.5(13)	32.2(11)
C36	6647(2)	5571(2)	6663.2(12)	31.0(10)
C37	6858(2)	3795(2)	5291.5(13)	34.3(11)
C38	6321(2)	3829(3)	5157.6(15)	44.5(13)
C39	5844(2)	3789(3)	5350.5(16)	50.7(14)
C40	5916(2)	3726(2)	5670.0(14)	41.4(12)
C41	6465(2)	3711.7(19)	5792.0(13)	30.4(10)
C42	6603(2)	3696.0(18)	6129.0(12)	29.7(10)
C43	6236(2)	3679(2)	6384.1(14)	38.5(12)
C44	6469(3)	3672(2)	6687.7(14)	45.0(14)
C45	7051(3)	3674(2)	6728.9(13)	39.4(12)
C46	7404(2)	3688.3(19)	6465.6(12)	30.9(10)
C47	8027(2)	3713.7(19)	6451.6(12)	32.2(11)
C48	8389(3)	3722(3)	6714.1(14)	45.8(14)
C49	8963(3)	3807(3)	6669.3(16)	53.5(16)
C50	9171(2)	3899(3)	6373.8(16)	47.7(14)
C51	8796(2)	3876(2)	6120.3(14)	37.3(12)
Co1	8229.2(3)	2429.7(3)	5365.8(2)	21.68(16)
Co2	7652.0(3)	3710.2(3)	5827.3(2)	23.11(17)
N1	7742.2(16)	2395.3(15)	4989.1(10)	25.5(8)
N2	8087.5(15)	1646.2(14)	5366.4(9)	23.1(7)
N3	8678.5(17)	2222.5(16)	5732.8(10)	28.3(8)

Atom	x	y	z	U(eq)
N4	8919.9(16)	2413.7(15)	5096.4(10)	24.5(8)
N5	9594.1(16)	2924.4(16)	4827.5(10)	26.3(8)
N6	8408.7(16)	3222.8(14)	5346.6(9)	24.1(7)
N7	8149.1(16)	3675.5(15)	5477.1(9)	24.0(8)
N8	7788.2(15)	4538.7(15)	5759.6(9)	25.2(8)
N9	8389.4(16)	5189.0(15)	5538.3(10)	27.4(8)
N10	6931.6(16)	3728.9(15)	5602.0(10)	26.9(8)
N11	7168.7(17)	3716.5(14)	6176.1(10)	26.5(8)
N12	8237.3(17)	3763.8(16)	6158.1(10)	30.5(9)
O1	7562.6(14)	2594.6(13)	5602.0(8)	29.8(7)
O2	7673.0(14)	2904.8(13)	5874.2(8)	30.5(7)
O3	10458.7(16)	1114.4(15)	4439.3(10)	43.4(9)
O4	9708.5(15)	569.8(13)	4750.5(9)	36.2(8)
O5	7751.4(14)	6674.1(13)	6287.4(8)	30.9(7)
O6	7055.0(14)	5948.8(13)	6524.6(8)	30.3(7)
C52	9313(4)	7184(3)	5452(2)	81(3)
C53	8820(5)	7277(4)	5226(3)	99(3)
C54	9790(3)	6409(4)	5704(2)	79(2)
C55	9788(5)	5755(5)	5715(3)	115(4)
O11	9332(2)	6594(3)	5498.7(16)	79.8(16)
F1	6893(3)	4708(2)	4129.3(15)	112(2)
F2	7417(2)	5325.1(16)	4394.9(9)	68.5(11)
F3	6827(3)	4750(2)	4657.0(17)	125(3)
F4	7715(3)	4642(3)	4711.8(17)	129(3)
F5	7203(3)	3993.5(17)	4433.7(14)	121(2)
F6	7810(3)	4588(3)	4173.4(19)	152(4)
P1	7308.0(6)	4652.1(6)	4411.9(4)	40.9(3)
F7	9516.7(14)	5074.1(16)	3784.1(9)	54.3(9)
F8	10101.6(13)	5673.9(13)	3523.2(9)	50.0(8)
F9	9522.4(14)	5083.4(14)	3257.2(9)	51.7(9)
K1	10000	5000	4395.0(5)	46.2(4)
O9	9962.0(18)	5939.3(16)	4746.3(11)	48.2(10)
O10	8883(2)	5206(3)	4501.7(13)	71.9(14)
P2	10000	5000	3521.3(5)	36.8(4)
O7	10000	0	4110.0(18)	64.9(18)
O8	8840(2)	5829(2)	5052.5(13)	67.3(14)

Table S39. Anisotropic Displacement Parameters ($Å^2 \times 10^3$) for **3-I**.

Atom	U_{11}	U_{22}	U_{33}	U_{23}	U_{13}	U_{12}
C1	34(2)	27(2)	26(3)	-0.8(19)	0(2)	5.8(18)
C2	44(3)	34(2)	36(3)	5(2)	-10(2)	9(2)
C3	43(3)	42(3)	39(3)	-1(2)	-19(2)	2(2)
C4	38(3)	33(2)	46(4)	-3(2)	-12(2)	-1(2)
C5	26(2)	29(2)	28(3)	0.7(19)	-0.2(19)	0.6(17)
C6	26(2)	25(2)	27(3)	2.4(18)	1.4(18)	0.8(16)
C7	36(2)	29(2)	33(3)	1(2)	0(2)	-6.5(18)
C8	41(3)	25(2)	37(3)	6(2)	5(2)	-2.5(18)
C9	32(2)	28(2)	29(3)	5(2)	2(2)	4.2(18)
C10	28(2)	29(2)	23(3)	3.0(18)	2.7(19)	2.5(17)
C11	36(2)	29(2)	22(3)	4.8(19)	4.4(19)	1.8(18)
C12	45(3)	38(3)	24(3)	6(2)	-1(2)	5(2)
C13	60(3)	48(3)	30(3)	2(2)	-17(3)	9(3)
C14	72(4)	44(3)	37(3)	-8(3)	-29(3)	4(3)
C15	57(3)	31(2)	36(3)	-6(2)	-14(3)	2(2)
C16	28(2)	22(2)	20(2)	-1.9(17)	-2.4(18)	2.8(16)
C17	25(2)	27(2)	17(2)	-1.4(17)	-2.4(17)	4.3(16)
C18	25(2)	26(2)	23(2)	2.6(18)	-1.4(18)	2.5(16)
C19	32(2)	25(2)	27(3)	1.0(19)	1.6(19)	6.0(17)

Atom	U_{11}	U_{22}	U_{33}	U_{23}	U_{13}	U_{12}
C20	37(2)	31(2)	28(3)	3(2)	7(2)	12.6(19)
C21	35(2)	33(2)	31(3)	4(2)	6(2)	3.7(19)
C22	28(2)	28(2)	21(2)	0.8(18)	0.7(18)	3.2(17)
C23	71(4)	38(3)	74(5)	8(3)	47(4)	14(3)
C24	50(3)	25(2)	54(4)	6(2)	20(3)	7(2)
C25	26(2)	27(2)	20(2)	-3.3(18)	0.3(18)	0.9(16)
C26	31(2)	24(2)	26(3)	0.4(18)	1.6(19)	-0.4(17)
C27	32(2)	25(2)	26(3)	-1.7(19)	2(2)	2.4(17)
C28	33(2)	24(2)	22(2)	-2.7(18)	2.3(19)	2.2(17)
C29	31(2)	24(2)	23(2)	-2.8(18)	3.5(19)	2.5(16)
C30	32(2)	21.5(19)	25(3)	-0.5(18)	0.4(19)	-1.8(17)
C31	34(2)	23(2)	21(2)	-1.3(18)	-2.1(19)	1.7(17)
C32	28(2)	31(2)	18(2)	-3.3(18)	0.9(18)	0.3(17)
C33	29(2)	24(2)	20(2)	0.9(18)	1.8(18)	1.3(16)
C34	29(2)	25(2)	22(2)	1.4(18)	-1.5(18)	1.6(16)
C35	42(3)	25(2)	30(3)	2(2)	-1(2)	-4.4(19)
C36	36(2)	35(2)	22(3)	0(2)	6(2)	-0.2(19)
C37	40(3)	37(2)	26(3)	-4(2)	3(2)	5(2)
C38	45(3)	61(3)	28(3)	-1(3)	-7(2)	8(3)
C39	38(3)	70(4)	44(4)	-2(3)	-7(3)	9(3)
C40	35(3)	52(3)	37(3)	-5(2)	4(2)	8(2)
C41	33(2)	29(2)	29(3)	0(2)	6(2)	2.9(18)
C42	41(2)	23(2)	25(3)	-1.2(18)	6(2)	1.0(18)
C43	41(3)	38(3)	37(3)	0(2)	13(2)	-5(2)
C44	59(3)	49(3)	27(3)	-1(2)	18(3)	-7(3)
C45	59(3)	37(3)	22(3)	-1(2)	7(2)	-2(2)
C46	49(3)	23(2)	20(3)	0.7(18)	2(2)	2.1(18)
C47	48(3)	28(2)	21(3)	-3.0(19)	-5(2)	7(2)
C48	62(4)	50(3)	25(3)	-8(2)	-8(3)	17(3)
C49	55(4)	62(4)	44(4)	-21(3)	-16(3)	16(3)
C50	39(3)	53(3)	52(4)	-18(3)	-12(3)	4(2)
C51	34(2)	36(2)	42(3)	-6(2)	-3(2)	0(2)
Co1	26.4(3)	21.6(3)	17.1(4)	-0.3(3)	0.1(3)	2.2(2)
Co2	29.8(4)	21.2(3)	18.3(4)	-0.3(3)	2.2(3)	1.1(3)
N1	28.0(18)	24.3(18)	24(2)	-0.1(15)	0.0(16)	3.3(14)
N2	26.2(17)	23.1(16)	20(2)	2.0(15)	0.7(16)	2.0(13)
N3	36(2)	27.9(18)	21(2)	-3.6(16)	-3.3(16)	4.1(15)
N4	28.1(18)	24.3(17)	21(2)	-2.0(15)	-0.4(15)	1.1(14)
N5	29.9(18)	26.6(18)	22(2)	-1.0(15)	-0.3(16)	2.6(15)
N6	29.4(18)	24.2(17)	18.7(19)	-1.3(15)	0.9(16)	3.7(14)
N7	31.3(18)	20.2(17)	21(2)	-1.7(15)	2.9(15)	3.9(14)
N8	32.0(19)	21.8(17)	22(2)	-1.0(15)	3.2(16)	0.9(14)
N9	34(2)	23.6(17)	25(2)	0.5(15)	6.5(17)	0.5(15)
N10	32.2(19)	25.3(18)	23(2)	-2.4(15)	3.8(17)	0.9(15)
N11	38(2)	19.3(16)	22(2)	2.8(15)	6.0(17)	-0.1(14)
N12	41(2)	25.6(18)	25(2)	-3.2(16)	-3.6(17)	3.9(16)
O1	34.4(17)	27.6(16)	27(2)	-3.2(14)	3.0(14)	0.5(12)
O2	40.0(17)	27.8(15)	23.8(19)	-1.7(14)	1.3(14)	0.5(13)
O3	51(2)	33.0(17)	46(2)	6.7(17)	24.2(19)	13.6(15)
O4	43.4(19)	23.1(15)	42(2)	2.5(15)	12.4(16)	8.3(13)
O5	42.0(18)	24.2(15)	27(2)	-6.8(13)	5.5(15)	-1.9(13)
O6	37.2(17)	29.1(15)	24.7(19)	-5.3(13)	9.2(14)	-1.6(13)
C52	91(6)	63(4)	90(7)	-8(4)	25(5)	1(4)
C53	111(8)	79(6)	106(9)	8(5)	-33(6)	10(5)
C54	60(4)	90(6)	88(7)	-1(5)	-13(4)	-11(4)
C55	89(7)	95(7)	160(13)	21(7)	-47(7)	-1(5)
O11	75(3)	79(4)	85(4)	-3(3)	-3(3)	-8(3)
F1	121(4)	100(4)	115(5)	45(3)	-65(4)	-53(3)
F2	120(3)	48(2)	37(2)	0.7(17)	4(2)	-19(2)

Atom	U_{11}	U_{22}	U_{33}	U_{23}	U_{13}	U_{12}
F3	148(5)	87(3)	139(6)	5(3)	104(5)	-10(3)
F4	130(5)	130(5)	127(6)	75(4)	-65(4)	-30(4)
F5	243(8)	29(2)	89(4)	6(2)	19(4)	-7(3)
F6	191(7)	98(4)	167(7)	40(4)	136(6)	72(4)
P1	51.3(8)	34.5(7)	36.9(8)	6.3(6)	8.4(6)	5.2(6)
F7	42.9(17)	73(2)	47(2)	10.4(18)	11.8(15)	19.5(16)
F8	44.9(17)	42.3(17)	63(2)	-7.3(16)	-1.7(16)	2.2(13)
F9	50.9(18)	52.9(19)	51(2)	7.6(16)	-15.5(16)	-4.7(15)
K1	47.0(8)	44.3(8)	47.2(11)	0	0	-5.2(7)
O9	55(2)	35.8(18)	54(3)	0.1(18)	-16(2)	-1.4(17)
O10	64(3)	100(4)	52(3)	-4(3)	7(2)	-18(3)
P2	31.5(8)	41.6(9)	37.2(11)	0	0	3.7(7)
O7	89(5)	52(4)	53(4)	0	0	13(3)
O8	85(4)	60(3)	57(3)	4(2)	26(3)	-12(3)

Table S40. Bond Lengths for **3-I**.

Atom	Atom	Length/Å	Atom	Atom	Length/Å
C1	C2	1.365(7)	C37	C38	1.382(7)
C1	N1	1.338(6)	C37	N10	1.338(7)
C2	C3	1.392(7)	C38	C39	1.389(9)
C3	C4	1.376(8)	C39	C40	1.374(9)
C4	C5	1.386(7)	C40	C41	1.387(7)
C5	C6	1.466(7)	C41	C42	1.467(7)
C5	N1	1.366(6)	C41	N10	1.358(6)
C6	C7	1.395(6)	C42	C43	1.383(7)
C6	N2	1.347(6)	C42	N11	1.341(6)
C7	C8	1.387(7)	C43	C44	1.399(9)
C8	C9	1.386(7)	C44	C45	1.374(9)
C9	C10	1.386(6)	C45	C46	1.390(7)
C10	C11	1.465(7)	C46	C47	1.461(7)
C10	N2	1.344(6)	C46	N11	1.348(7)
C11	C12	1.371(7)	C47	C48	1.400(8)
C11	N3	1.367(6)	C47	N12	1.345(7)
C12	C13	1.381(8)	C48	C49	1.373(9)
C13	C14	1.382(8)	C49	C50	1.363(10)
C14	C15	1.388(8)	C50	C51	1.390(8)
C15	N3	1.330(7)	C51	N12	1.344(7)
C16	C25	1.467(6)	Co1	N1	1.965(4)
C16	N4	1.357(6)	Co1	N2	1.865(3)
C16	N5	1.334(6)	Co1	N3	1.941(4)
C17	C18	1.405(6)	Co1	N4	1.981(4)
C17	C22	1.404(6)	Co1	N6	1.906(3)
C17	N4	1.371(6)	Co1	O1	1.895(3)
C18	C19	1.375(6)	Co2	N7	1.890(4)
C19	C20	1.419(7)	Co2	N8	1.987(4)
C19	O4	1.373(5)	Co2	N10	1.940(4)
C20	C21	1.382(7)	Co2	N11	1.863(4)
C20	O3	1.376(6)	Co2	N12	1.966(4)
C21	C22	1.411(7)	Co2	O2	1.898(3)
C22	N5	1.402(6)	N6	N7	1.342(5)
C23	O3	1.408(7)	O1	O2	1.389(5)
C24	O4	1.432(6)	C52	C53	1.515(13)
C25	C26	1.397(6)	C52	O11	1.397(10)
C25	N6	1.354(6)	C54	C55	1.533(13)
C26	C27	1.392(7)	C54	O11	1.447(10)
C27	C28	1.458(6)	F1	P1	1.549(5)
C27	N7	1.361(6)	F2	P1	1.598(4)
C28	N8	1.365(6)	F3	P1	1.551(5)

Atom	Atom	Length/Å	Atom	Atom	Length/Å
C28	N9	1.326(6)	F4	P1	1.590(6)
C29	C30	1.409(6)	F5	P1	1.565(4)
C29	C34	1.389(6)	F6	P1	1.558(5)
C29	N9	1.391(6)	F7	K1	2.834(4)
C30	C31	1.371(7)	F7	P2	1.599(3)
C31	C32	1.429(6)	F8	P2	1.596(3)
C31	O5	1.375(5)	F9	P2	1.595(4)
C32	C33	1.370(6)	K1	O9	2.659(4)
C32	O6	1.372(6)	K1	O9[1]	2.659(4)
C33	C34	1.412(7)	K1	O10	2.698(5)
C34	N8	1.380(6)	K1	O10[1]	2.698(5)
C35	O5	1.441(6)	K1	P2	3.707(3)
C36	O6	1.429(6)			

Table S41. Bond Angles for **3-I**.

Atom	Atom	Atom	Angle/˚	Atom	Atom	Atom	Angle/˚
N1	C1	C2	123.1(4)	N11	Co2	N7	177.88(15)
C1	C2	C3	119.7(5)	N11	Co2	N8	101.80(15)
C4	C3	C2	118.3(5)	N11	Co2	N10	82.11(18)
C3	C4	C5	119.3(5)	N11	Co2	N12	81.73(18)
C4	C5	C6	124.4(4)	N11	Co2	O2	86.58(15)
N1	C5	C4	122.2(4)	N12	Co2	N8	85.92(16)
N1	C5	C6	113.4(4)	O2	Co2	N8	169.01(15)
C7	C6	C5	127.3(5)	O2	Co2	N10	95.55(15)
N2	C6	C5	111.9(4)	O2	Co2	N12	88.30(15)
N2	C6	C7	120.8(4)	C1	N1	C5	117.4(4)
C8	C7	C6	117.8(5)	C1	N1	Co1	128.6(3)
C9	C8	C7	121.0(4)	C5	N1	Co1	113.9(3)
C10	C9	C8	118.3(4)	C6	N2	Co1	119.0(3)
C9	C10	C11	127.7(4)	C10	N2	C6	121.3(4)
N2	C10	C9	120.8(4)	C10	N2	Co1	119.4(3)
N2	C10	C11	111.4(4)	C11	N3	Co1	115.2(3)
C12	C11	C10	125.5(4)	C15	N3	C11	118.3(4)
N3	C11	C10	112.5(4)	C15	N3	Co1	126.5(3)
N3	C11	C12	122.0(5)	C16	N4	C17	104.1(4)
C11	C12	C13	119.6(5)	C16	N4	Co1	115.9(3)
C12	C13	C14	118.5(5)	C17	N4	Co1	140.0(3)
C13	C14	C15	119.4(5)	C16	N5	C22	102.5(4)
N3	C15	C14	122.2(5)	C25	N6	Co1	120.5(3)
N4	C16	C25	113.7(4)	N7	N6	C25	108.5(3)
N5	C16	C25	130.0(4)	N7	N6	Co1	130.7(3)
N5	C16	N4	116.3(4)	C27	N7	Co2	120.1(3)
C22	C17	C18	122.0(4)	N6	N7	C27	108.3(4)
N4	C17	C18	130.1(4)	N6	N7	Co2	129.6(3)
N4	C17	C22	107.9(4)	C28	N8	C34	103.9(4)
C19	C18	C17	117.2(4)	C28	N8	Co2	114.0(3)
C18	C19	C20	121.3(4)	C34	N8	Co2	138.9(3)
O4	C19	C18	123.9(4)	C28	N9	C29	103.1(4)
O4	C19	C20	114.8(4)	C37	N10	C41	118.9(4)
C21	C20	C19	121.8(4)	C37	N10	Co2	126.9(3)
O3	C20	C19	113.4(4)	C41	N10	Co2	114.0(3)
O3	C20	C21	124.8(5)	C42	N11	C46	122.5(4)
C20	C21	C22	117.3(4)	C42	N11	Co2	118.8(3)
C17	C22	C21	120.3(4)	C46	N11	Co2	118.4(3)
N5	C22	C17	109.2(4)	C47	N12	Co2	113.6(3)
N5	C22	C21	130.5(4)	C51	N12	C47	119.0(5)
C26	C25	C16	140.1(4)	C51	N12	Co2	127.3(4)
N6	C25	C16	110.3(4)	O2	O1	Co1	113.1(2)

Atom	Atom	Atom	Angle/˚	Atom	Atom	Atom	Angle/˚
N6	C25	C26	109.6(4)	O1	O2	Co2	115.3(2)
C27	C26	C25	104.1(4)	C20	O3	C23	117.5(4)
C26	C27	C28	139.5(4)	C19	O4	C24	116.5(4)
N7	C27	C26	109.6(4)	C31	O5	C35	115.8(4)
N7	C27	C28	110.3(4)	C32	O6	C36	117.7(3)
N8	C28	C27	114.6(4)	O11	C52	C53	104.9(7)
N9	C28	C27	129.4(4)	O11	C54	C55	108.5(7)
N9	C28	N8	115.6(4)	C52	O11	C54	114.0(7)
C34	C29	C30	120.8(4)	F1	P1	F2	88.9(3)
C34	C29	N9	109.8(4)	F1	P1	F3	92.9(4)
N9	C29	C30	129.4(4)	F1	P1	F4	175.5(4)
C31	C30	C29	117.4(4)	F1	P1	F5	91.8(3)
C30	C31	C32	121.9(4)	F1	P1	F6	88.8(5)
C30	C31	O5	124.5(4)	F3	P1	F2	90.0(3)
O5	C31	C32	113.6(4)	F3	P1	F4	84.4(4)
C33	C32	C31	120.8(4)	F3	P1	F5	89.5(3)
C33	C32	O6	124.1(4)	F3	P1	F6	176.5(4)
O6	C32	C31	115.0(4)	F4	P1	F2	87.5(3)
C32	C33	C34	117.3(4)	F5	P1	F2	179.2(3)
C29	C34	C33	121.8(4)	F5	P1	F4	91.8(3)
N8	C34	C29	107.6(4)	F6	P1	F2	86.9(3)
N8	C34	C33	130.6(4)	F6	P1	F4	93.8(5)
N10	C37	C38	122.0(5)	F6	P1	F5	93.5(4)
C37	C38	C39	119.1(6)	P2	F7	K1	110.40(17)
C40	C39	C38	119.3(5)	F7	K1	F7[1]	47.68(13)
C39	C40	C41	119.0(5)	F7	K1	P2	23.84(7)
C40	C41	C42	124.7(5)	F7[1]	K1	P2	23.84(7)
N10	C41	C40	121.6(5)	O9	K1	F7[1]	125.23(13)
N10	C41	C42	113.7(4)	O9[1]	K1	F7	125.23(13)
C43	C42	C41	128.8(5)	O9[1]	K1	F7[1]	116.66(12)
N11	C42	C41	111.2(4)	O9	K1	F7	116.66(12)
N11	C42	C43	119.9(5)	O9	K1	O9[1]	111.8(2)
C42	C43	C44	118.5(5)	O9[1]	K1	O10[1]	74.09(15)
C45	C44	C43	120.3(5)	O9	K1	O10	74.09(15)
C44	C45	C46	119.2(5)	O9[1]	K1	O10	94.93(15)
C45	C46	C47	128.9(5)	O9	K1	O10[1]	94.93(15)
N11	C46	C45	119.4(5)	O9[1]	K1	P2	124.10(10)
N11	C46	C47	111.7(4)	O9	K1	P2	124.10(10)
C48	C47	C46	124.9(5)	O10[1]	K1	F7	123.49(15)
N12	C47	C46	114.0(4)	O10	K1	F7[1]	123.49(15)
N12	C47	C48	120.9(5)	O10[1]	K1	F7[1]	75.83(13)
C49	C48	C47	119.0(6)	O10	K1	F7	75.83(13)
C50	C49	C48	120.1(6)	O10	K1	O10[1]	160.7(2)
C49	C50	C51	118.6(6)	O10[1]	K1	P2	99.66(12)
N12	C51	C50	122.1(6)	O10	K1	P2	99.66(12)
N1	Co1	N4	90.23(16)	F7	P2	F7[1]	91.5(3)
N2	Co1	N1	81.81(16)	F7[1]	P2	K1	45.76(14)
N2	Co1	N3	81.33(16)	F7	P2	K1	45.76(14)
N2	Co1	N4	97.32(15)	F8	P2	F7[1]	89.9(2)
N2	Co1	N6	176.56(17)	F8[1]	P2	F7[1]	89.7(2)
N2	Co1	O1	93.05(15)	F8	P2	F7	89.7(2)
N3	Co1	N1	163.11(16)	F8[1]	P2	F7	89.9(2)
N3	Co1	N4	90.88(17)	F8	P2	F8[1]	179.4(3)
N6	Co1	N1	97.66(16)	F8	P2	K1	89.70(16)
N6	Co1	N3	99.11(16)	F8[1]	P2	K1	89.71(16)
N6	Co1	N4	79.26(15)	F9[1]	P2	F7[1]	88.86(19)
O1	Co1	N1	87.72(16)	F9	P2	F7[1]	179.1(2)
O1	Co1	N3	94.21(17)	F9[1]	P2	F7	179.1(2)
O1	Co1	N4	169.03(15)	F9	P2	F7	88.86(19)

Atom	Atom	Atom	Angle/˚	Atom	Atom	Atom	Angle/˚
O1	Co1	N6	90.33(15)	F9	P2	F8	89.26(19)
N7	Co2	N8	80.18(15)	F9	P2	F8[1]	91.16(19)
N7	Co2	N10	98.59(17)	F9[1]	P2	F8	91.16(19)
N7	Co2	N12	97.72(17)	F9[1]	P2	F8[1]	89.26(19)
N7	Co2	O2	91.36(15)	F9[1]	P2	F9	90.8(3)
N10	Co2	N8	92.67(16)	F9[1]	P2	K1	134.62(15)
N10	Co2	N12	163.13(17)	F9	P2	K1	134.62(15)

Table S42. Torsion Angles for **3-I**.

A	B	C	D	Angle/˚	A	B	C	D	Angle/˚
C1	C2	C3	C4	0.7(9)	C41	C42	N11	C46	-177.7(4)
C2	C1	N1	C5	-0.4(7)	C41	C42	N11	Co2	-4.0(5)
C2	C1	N1	Co1	176.6(4)	C42	C41	N10	C37	-174.2(4)
C2	C3	C4	C5	-0.2(9)	C42	C41	N10	Co2	1.3(5)
C3	C4	C5	C6	-177.7(5)	C42	C43	C44	C45	-1.1(8)
C3	C4	C5	N1	-0.6(8)	C43	C42	N11	C46	3.9(6)
C4	C5	C6	C7	1.5(8)	C43	C42	N11	Co2	177.6(3)
C4	C5	C6	N2	178.3(5)	C43	C44	C45	C46	0.8(8)
C4	C5	N1	C1	0.9(7)	C44	C45	C46	C47	177.5(5)
C4	C5	N1	Co1	-176.6(4)	C44	C45	C46	N11	1.8(7)
C5	C6	C7	C8	174.2(5)	C45	C46	C47	C48	0.8(8)
C5	C6	N2	C10	-176.1(4)	C45	C46	C47	N12	-175.5(5)
C5	C6	N2	Co1	-2.6(5)	C45	C46	N11	C42	-4.2(6)
C6	C5	N1	C1	178.2(4)	C45	C46	N11	Co2	-177.9(4)
C6	C5	N1	Co1	0.8(5)	C46	C47	C48	C49	-173.2(5)
C6	C7	C8	C9	1.5(7)	C46	C47	N12	C51	170.2(4)
C7	C6	N2	C10	0.9(7)	C46	C47	N12	Co2	-5.9(5)
C7	C6	N2	Co1	174.5(3)	C47	C46	N11	C42	179.4(4)
C7	C8	C9	C10	0.6(7)	C47	C46	N11	Co2	5.7(5)
C8	C9	C10	C11	-177.8(5)	C47	C48	C49	C50	2.0(9)
C8	C9	C10	N2	-2.1(7)	C48	C47	N12	C51	-6.3(7)
C9	C10	C11	C12	-5.3(8)	C48	C47	N12	Co2	177.7(4)
C9	C10	C11	N3	172.8(5)	C48	C49	C50	C51	-3.2(9)
C9	C10	N2	C6	1.3(7)	C49	C50	C51	N12	-0.3(8)
C9	C10	N2	Co1	-172.2(3)	C50	C51	N12	C47	5.0(7)
C10	C11	C12	C13	178.6(5)	C50	C51	N12	Co2	-179.5(4)
C10	C11	N3	C15	-177.2(5)	Co1	N6	N7	C27	-175.1(3)
C10	C11	N3	Co1	1.1(5)	Co1	N6	N7	Co2	21.4(6)
C11	C10	N2	C6	177.6(4)	Co1	O1	O2	Co2	90.0(3)
C11	C10	N2	Co1	4.1(5)	N1	C1	C2	C3	-0.4(8)
C11	C12	C13	C14	-1.4(9)	N1	C5	C6	C7	-175.8(4)
C12	C11	N3	C15	1.0(7)	N1	C5	C6	N2	1.0(6)
C12	C11	N3	Co1	179.3(4)	N1	Co1	N2	C6	2.4(3)
C12	C13	C14	C15	0.6(10)	N1	Co1	N2	C10	176.1(4)
C13	C14	C15	N3	1.0(10)	N1	Co1	O1	O2	-153.4(3)
C14	C15	N3	C11	-1.8(9)	N2	C6	C7	C8	-2.3(7)
C14	C15	N3	Co1	-179.9(5)	N2	C10	C11	C12	178.7(5)
C16	C25	C26	C27	-177.3(6)	N2	C10	C11	N3	-3.2(6)
C16	C25	N6	Co1	-6.3(5)	N2	Co1	O1	O2	124.9(3)
C16	C25	N6	N7	178.7(4)	N3	C11	C12	C13	0.6(8)
C17	C18	C19	C20	2.0(7)	N3	Co1	N2	C6	-176.5(4)
C17	C18	C19	O4	-177.7(4)	N3	Co1	N2	C10	-2.9(3)
C17	C22	N5	C16	-0.6(5)	N3	Co1	O1	O2	43.4(3)
C18	C17	C22	C21	2.0(7)	N4	C16	C25	C26	179.2(6)
C18	C17	C22	N5	-178.7(4)	N4	C16	C25	N6	1.7(5)
C18	C17	N4	C16	178.7(5)	N4	C16	N5	C22	-0.2(5)
C18	C17	N4	Co1	-1.9(8)	N4	C17	C18	C19	177.2(5)
C18	C19	C20	C21	-0.2(8)	N4	C17	C22	C21	-178.0(4)

A	B	C	D	Angle/˚	A	B	C	D	Angle/˚
C18	C19	C20	O3	179.1(5)	N4	C17	C22	N5	1.2(5)
C18	C19	O4	C24	-2.5(7)	N4	Co1	N2	C6	-86.8(3)
C19	C20	C21	C22	-0.8(8)	N4	Co1	N2	C10	86.9(4)
C19	C20	O3	C23	163.9(6)	N4	Co1	O1	O2	-74.0(9)
C20	C19	O4	C24	177.8(5)	N5	C16	C25	C26	1.3(10)
C20	C21	C22	C17	-0.2(7)	N5	C16	C25	N6	-176.2(5)
C20	C21	C22	N5	-179.3(5)	N5	C16	N4	C17	0.9(5)
C21	C20	O3	C23	-16.8(8)	N5	C16	N4	Co1	-178.6(3)
C21	C22	N5	C16	178.5(5)	N6	C25	C26	C27	0.2(5)
C22	C17	C18	C19	-2.9(7)	N6	Co1	O1	O2	-55.7(3)
C22	C17	N4	C16	-1.3(5)	N7	C27	C28	N8	-0.8(6)
C22	C17	N4	Co1	178.1(4)	N7	C27	C28	N9	171.4(5)
C25	C16	N4	C17	-177.3(4)	N7	Co2	O2	O1	-50.7(3)
C25	C16	N4	Co1	3.2(5)	N8	C28	N9	C29	1.8(5)
C25	C16	N5	C22	177.7(5)	N8	Co2	N7	C27	8.4(4)
C25	C26	C27	C28	169.0(6)	N8	Co2	N7	N6	170.2(4)
C25	C26	C27	N7	-0.7(5)	N8	Co2	N11	C42	94.9(3)
C25	N6	N7	C27	-0.8(5)	N8	Co2	N11	C46	-91.1(3)
C25	N6	N7	Co2	-164.3(3)	N8	Co2	O2	O1	-90.1(9)
C26	C25	N6	Co1	175.4(3)	N9	C28	N8	C34	-2.3(5)
C26	C25	N6	N7	0.4(5)	N9	C28	N8	Co2	-166.2(3)
C26	C27	C28	N8	-170.5(6)	N9	C29	C30	C31	-179.8(5)
C26	C27	C28	N9	1.8(10)	N9	C29	C34	C33	-179.1(4)
C26	C27	N7	Co2	166.3(3)	N9	C29	C34	N8	-0.8(5)
C26	C27	N7	N6	1.0(5)	N10	C37	C38	C39	-0.5(8)
C27	C28	N8	C34	171.1(4)	N10	C41	C42	C43	179.8(5)
C27	C28	N8	Co2	7.2(5)	N10	C41	C42	N11	1.5(5)
C27	C28	N9	C29	-170.4(5)	N10	Co2	N7	C27	99.6(4)
C28	C27	N7	Co2	-6.6(5)	N10	Co2	N7	N6	-98.6(4)
C28	C27	N7	N6	-171.9(4)	N10	Co2	N11	C42	3.8(3)
C29	C30	C31	C32	-1.0(7)	N10	Co2	N11	C46	177.8(3)
C29	C30	C31	O5	176.3(4)	N10	Co2	O2	O1	48.0(3)
C29	C34	N8	C28	1.7(5)	N11	C42	C43	C44	-1.2(7)
C29	C34	N8	Co2	159.0(4)	N11	C46	C47	C48	176.7(4)
C30	C29	C34	C33	1.1(7)	N11	C46	C47	N12	0.5(6)
C30	C29	C34	N8	179.4(4)	N11	Co2	O2	O1	129.8(3)
C30	C29	N9	C28	179.2(5)	N12	C47	C48	C49	2.9(8)
C30	C31	C32	C33	1.1(7)	N12	Co2	N7	C27	-76.1(4)
C30	C31	C32	O6	179.8(4)	N12	Co2	N7	N6	85.7(4)
C30	C31	O5	C35	-6.7(7)	N12	Co2	N11	C42	179.0(3)
C31	C32	C33	C34	0.0(7)	N12	Co2	N11	C46	-7.1(3)
C31	C32	O6	C36	177.3(4)	N12	Co2	O2	O1	-148.4(3)
C32	C31	O5	C35	170.9(4)	O1	Co1	N2	C6	89.7(3)
C32	C33	C34	C29	-1.1(7)	O1	Co1	N2	C10	-96.7(4)
C32	C33	C34	N8	-178.9(4)	O2	Co2	N7	C27	-164.6(4)
C33	C32	O6	C36	-4.0(7)	O2	Co2	N7	N6	-2.7(4)
C33	C34	N8	C28	179.8(5)	O2	Co2	N11	C42	-92.3(3)
C33	C34	N8	Co2	-22.9(8)	O2	Co2	N11	C46	81.7(3)
C34	C29	C30	C31	0.0(7)	O3	C20	C21	C22	-180.0(5)
C34	C29	N9	C28	-0.6(5)	O4	C19	C20	C21	179.5(5)
C37	C38	C39	C40	1.0(9)	O4	C19	C20	O3	-1.2(6)
C38	C37	N10	C41	-2.0(7)	O5	C31	C32	C33	-176.5(4)
C38	C37	N10	Co2	-176.9(4)	O5	C31	C32	O6	2.2(6)
C38	C39	C40	C41	0.8(9)	O6	C32	C33	C34	-178.6(4)
C39	C40	C41	C42	174.5(5)	C53	C52	O11	C54	178.3(8)
C39	C40	C41	N10	-3.4(8)	C55	C54	O11	C52	-175.9(8)
C40	C41	C42	C43	1.7(8)	K1	F7	P2	F7[1]	-0.002(0)
C40	C41	C42	N11	-176.6(5)	K1	F7	P2	F8[1]	89.7(2)
C40	C41	N10	C37	4.0(7)	K1	F7	P2	F8	-89.89(19)

A	B	C	D	Angle/˚	A	B	C	D	Angle/˚
C40	C41	N10	Co2	179.5(4)	K1	F7	P2	F9	-179.16(18)
C41	C42	C43	C44	-179.3(5)					

Table S43. Hydrogen Atom Coordinates (Å×10^4) and Isotropic Displacement Parameters (Å^2×10^3) for **3-I**.

Atom	*x*	*y*	*z*	U(eq)
H1	7702.7	3197.78	4847.13	35
H2	7162.9	3052.79	4402.95	45
H3	6873.1	2121.66	4266.05	50
H4	7132.22	1364.76	4597.63	47
H7	7465.61	668.36	4948.7	39
H8	7938.41	84.28	5314.11	41
H9	8495.53	488.87	5714.04	36
H12	8979.62	1043.34	6099.06	43
H13	9497.85	1670.5	6421.13	55
H14	9509.75	2645.17	6293.03	61
H15	8998.81	2968.14	5857.83	49
H18	9001.79	1224.12	5080.7	30
H21	10385.07	2253.54	4486.58	40
H23A	10528.6	1533.57	4039.78	92
H23B	11024.22	1075.09	4097.63	92
H23C	11027.58	1666.16	4286.82	92
H24A	9339.83	-160.39	4874.41	65
H24B	8923.09	380	4892.92	65
H24C	9400.49	286.46	5157.76	65
H26	9102.64	4256.85	5055.64	33
H30	8355.2	6284.24	5801.17	32
H33	7101.15	4922.66	6260.82	29
H35A	8136.87	7161.41	5965.98	48
H35B	8564.74	6895.33	6220.72	48
H35C	8160.63	7419.08	6315.05	48
H36A	6393.94	5421.56	6498.87	47
H36B	6421.22	5778.54	6820.12	47
H36C	6847.04	5253.46	6765.49	47
H37	7183.83	3819.43	5159.1	41
H38	6279.1	3878.27	4936.56	53
H39	5470.91	3804.68	5262.49	61
H40	5594.34	3692.88	5805.3	50
H43	5833.91	3671.29	6353.97	46
H44	6224.42	3667.31	6866.14	54
H45	7210.14	3664.92	6934.8	47
H48	8239.23	3668.88	6920.19	55
H49	9215.56	3801.3	6844.48	64
H50	9564.93	3977.78	6341.6	57
H51	8938.57	3942.29	5913.86	45
H52A	9674.81	7323.35	5359.19	98
H52B	9244.03	7386.17	5652.96	98
H53A	8913.81	7107.06	5020.88	149
H53B	8753.54	7686.94	5199.65	149
H53C	8475.72	7095.75	5310.25	149
H54A	9733.38	6566.08	5918.02	95
H54B	10160.6	6547.68	5622.27	95
H55A	9736.77	5602.69	5502.02	172
H55B	9474.34	5623.74	5850.07	172
H55C	10151.87	5619.2	5801.47	172
H9A	10096.11	6269.49	4688.52	72
H9B	9684.18	6020.16	4874.49	72

Atom	*x*	*y*	*z*	U(eq)
H10A	8839.52	5355.56	4689.7	108
H10B	8695.52	4881.51	4511.66	108
H7A	9890.28	-191.19	4274.77	97
H7B	10163.77	303.12	4186.38	97
H8A	8873.93	6182.49	5112.15	101
H8B	9006.63	5633.47	5200.71	101

Table S44. Atomic Occupancy for **3-I**.

Atom	*Occupancy*	Atom	*Occupancy*	Atom	*Occupancy*
H7A	0.5	H7B	0.5		

19. References

(1) Chou, J.-L.; Chyn, J.-P.; Urbach, F.; Gervasio, D. Dinuclear copper (II) complexes incorporating a novel pyrazolo-based ligand with S-and N-rich coordination spheres. *Polyhedron* **2000**, *19*, 2215-2223.

(2) Laine, T. M.; Kärkäs, M. D.; Liao, R.-Z.; Åkermark, T.; Lee, B.-L.; Karlsson, E. A.; Siegbahn, P. E.; Åkermark, B. Efficient photochemical water oxidation by a dinuclear molecular ruthenium complex. *Chem. Commun.* **2015**, *51*, 1862-1865.

(3) Schenck, T. G.; Downes, J.; Milne, C.; Mackenzie, P. B.; Boucher, T. G.; Whelan, J.; Bosnich, B. Bimetallic reactivity. Synthesis of bimetallic complexes containing a bis (phosphino) pyrazole ligand. *Inorg. Chem.* **1985**, *24*, 2334-2337.

(4) Aran, V. J.; Kumar, M.; Molina, J.; Lamarque, L.; Navarro, P.; García-España, E.; Ramirez, J. A.; Luis, S. V.; Escuder, B. Synthesis and Protonation Behavior of 26-Membered Oxaaza and Polyaza Macrocycles Containing Two Heteroaromatic Units of 3, 5-Disubstituted Pyrazole or 1-Benzylpyrazole. A Potentiometric and ^{1}H and ^{13}C NMR study. *J. Org. Chem.* **1999**, *64*, 6135-6146.

(5) McNab, H. Synthesis of pyrrolo[1,2-*c*]imidazol-5-one, pyrrolo[1,2-*a*]imidazol-5-one and pyrrolo[1,2-*b*]pyrazol-6-one (three isomeric azapyrrolizinones), by pyrolysis of Meldrum's acid derivatives. *J. Chem. Soc., Perkin Trans. 1* **1987**, 653-656.

(6) Stuart, J. G.; Khora, S.; McKenney Jr, J. D.; Castle, R. N. The synthesis of dimethoxy- and trimethoxy[1]benzothieno[2,3-*c*]quinolines. *J. Heterocycl. Chem.* **1987**, *24*, 1589-1594.

(7) Cmheea, S. S.; Langford, S.; Cheung, N. S.; Beart, P. M.; Macfarlane, K. J.; Mulcair, M. Agents and methods for the treatment of disorders associated with oxidative stress. WO Pat. WO2003099762A1, 2003.

(8) Jinkerson, D. L.; Weinschenk III I, J.; Dean, D. W. UV-absorbers for ophthalmic lens materials. United States US Pat. US7803359B1, 2010.

(9) Hogg, R.; Wilkins, R. Exchange studies of certain chelate compounds of the transitional metals. Part VIII. 2,2′,2″-terpyridine complexes. *J. Chem. Soc.* **1962**, 341-350.

(10) Niemczak, M.; Biedziak, A.; Czerniak, K.; Marcinkowska, K. Preparation and characterization of new ionic liquid forms of 2,4-DP herbicide. *Tetrahedron* **2017**, *73*, 7315-7325.

(11) Inomata, K.; Nakayama, Y.; Kotake, H. Quaternary Ammonium Periodate as a New Oxidizing Agent. *Bull. Chem. Soc. Jpn.* **1980**, *53*, 565-566.

(12) Baitalik, S.; Flörke, U.; Nag, K. Mononuclear and Binuclear Ruthenium(II) Complexes Containing 2,2′-Bipyridine or 1,10-Phenanthroline and Pyrazole-3,5-Bis(benzimidazole). Synthesis, Structure, Isomerism, Spectroscopy, and Proton-Coupled Redox Activity. *Inorg. Chem.* **1999**, *38*, 3296-3308.

(13) Hsu, W. C.; Wang, Y. H. Homogeneous Water Oxidation Catalyzed by First-Row Transition Metal Complexes: Unveiling the Relationship between Turnover Frequency and Reaction Overpotential. *ChemSusChem* **2022**, *15*, e202102378.
(14) Franco, C.; Olmsted III, J. Photochemical determination of the solubility of oxygen in various media. *Talanta* **1990**, *37*, 905-909.
(15) Eisenberg, G. Colorimetric Determination of Hydrogen Peroxide. *Industrial & Engineering Chemistry Analytical Edition* **1943**, *15*, 327-328.
(16) Lee, Y.; Park, G. Y.; Lucas, H. R.; Vajda, P. L.; Kamaraj, K.; Vance, M. A.; Milligan, A. E.; Woertink, J. S.; Siegler, M. A.; Narducci Sarjeant, A. A.; et al. Copper (I)/O_2 Chemistry with Imidazole Containing Tripodal Tetradentate Ligands Leading to μ-1,2-Peroxo−Dicopper(II) Species. *Inorg. Chem.* **2009**, *48*, 11297-11309.
(17) Roberts, J. A.; Bullock, R. M. Direct Determination of Equilibrium Potentials for Hydrogen Oxidation/Production by Open Circuit Potential Measurements in Acetonitrile. *Inorg. Chem.* **2013**, *52*, 3823-3835.
(18) Stoll, S.; Schweiger, A. EasySpin, a comprehensive software package for spectral simulation and analysis in EPR. *J. Magn. Reson.* **2006**, *178*, 42-55.
(19) Caldeweyher, E.; Bannwarth, C.; Grimme, S. Extension of the D3 dispersion coefficient model. *J. Chem. Phys.* **2017**, *147*, 034112.
(20) Caldeweyher, E.; Ehlert, S.; Hansen, A.; Neugebauer, H.; Spicher, S.; Bannwarth, C.; Grimme, S. A generally applicable atomic-charge dependent London dispersion correction. *J. Chem. Phys.* **2019**, *150*, 154122.
(21) Neese, F. An improvement of the resolution of the identity approximation for the formation of the Coulomb matrix. *J. Comput. Chem.* **2003**, *24*, 1740-1747.
(22) Weigend, F. Accurate Coulomb-fitting basis sets for H to Rn. *Phys. Chem. Chem. Phys.* **2006**, *8*, 1057-1065.
(23) Helmich-Paris, B.; de Souza, B.; Neese, F.; Izsák, R. An improved chain of spheres for exchange algorithm. *J. Chem. Phys.* **2021**, *155*, 104109.
(24) Tao, J.; Perdew, J. P.; Staroverov, V. N.; Scuseria, G. E. Climbing the Density Functional Ladder: Nonempirical Meta–Generalized Gradient Approximation Designed for Molecules and Solids. *Phys. Rev. Lett.* **2003**, *91*, 146401.
(25) Hehre, W. J.; Ditchfield, R.; Pople, J. A. Self—Consistent Molecular Orbital Methods. XII. Further Extensions of Gaussian—Type Basis Sets for Use in Molecular Orbital Studies of Organic Molecules. *J. Chem. Phys.* **1972**, *56*, 2257-2261.
(26) Hay, P. J.; Wadt, W. R. *Ab initio* effective core potentials for molecular calculations. Potentials for the transition metal atoms Sc to Hg. *J. Chem. Phys.* **1985**, *82*, 270-283.

(27) Stephens, P. J.; Devlin, F. J.; Chabalowski, C. F.; Frisch, M. J. *Ab initio* Calculation of Vibrational Absorption and Circular Dichroism Spectra Using Density Functional Force Fields. *J. Phys. Chem.* **1994**, *98*, 11623-11627.
(28) Chai, J.-D.; Head-Gordon, M. Long-range corrected hybrid density functionals with damped atom–atom dispersion corrections. *Phys. Chem. Chem. Phys.* **2008**, *10*, 6615-6620.
(29) Weigend, F.; Ahlrichs, R. Balanced basis sets of split valence, triple zeta valence and quadruple zeta valence quality for H to Rn: Design and assessment of accuracy. *Phys. Chem. Chem. Phys.* **2005**, *7*, 3297-3305.
(30) Marenich, A. V.; Cramer, C. J.; Truhlar, D. G. Universal solvation model based on solute electron density and on a continuum model of the solvent defined by the bulk dielectric constant and atomic surface tensions. *J. Phys. Chem. B* **2009**, *113*, 6378-6396.
(31) Kelly, C. P.; Cramer, C. J.; Truhlar, D. G. Single-Ion Solvation Free Energies and the Normal Hydrogen Electrode Potential in Methanol, Acetonitrile, and Dimethyl Sulfoxide. *J. Phys. Chem. B* **2007**, *111*, 408-422.
(32) Cramer, C. Essentials Of Computational Chemistry: Theories And Models. 2010; pp 385-427.
(33) Kang, R.; Yao, J.; Chen, H. Are DFT Methods Accurate in Mononuclear Ruthenium-Catalyzed Water Oxidation? An ab Initio Assessment. *J. Chem. Theory Comput.* **2013**, *9*, 1872-1879.
(34) Bao, J. L.; Yu, H. S.; Duanmu, K.; Makeev, M. A.; Xu, X.; Truhlar, D. G. Density Functional Theory of the Water Splitting Reaction on Fe(0): Comparison of Local and Nonlocal Correlation Functionals. *ACS Catal.* **2015**, *5*, 2070-2080.
(35) Geer, A. M.; Musgrave Iii, C.; Webber, C.; Nielsen, R. J.; McKeown, B. A.; Liu, C.; Schleker, P. P. M.; Jakes, P.; Jia, X.; Dickie, D. A.; et al. Electrocatalytic Water Oxidation by a Trinuclear Copper(II) Complex. *ACS Catal.* **2021**, *11*, 7223-7240.
